# The Notch pathway in the annelid *Platynereis*: insights into chaetogenesis and neurogenesis processes

**DOI:** 10.1098/rsob.160242

**Published:** 2017-02-01

**Authors:** Eve Gazave, Quentin I. B. Lemaître, Guillaume Balavoine

**Affiliations:** Institut Jacques Monod, CNRS, UMR 7592, Univ Paris Diderot, Sorbonne Paris Cité, 75205 Paris, France

**Keywords:** Notch, *Platynereis*, neurogenesis, chaetogenesis, lateral inhibition

## Abstract

Notch is a key signalling pathway playing multiple and varied functions during development. Notch regulates the selection of cells with a neurogenic fate and maintains a pool of yet uncommitted precursors through lateral inhibition, both in insects and in vertebrates. Here, we explore the functions of Notch in the annelid *Platynereis dumerilii* (Lophotrochozoa). Conserved components of the pathway are identified and a scenario for their evolution in metazoans is proposed. Unexpectedly, neither *Notch* nor its ligands are expressed in the neurogenic epithelia of the larva at the time when massive neurogenesis begins. Using chemical inhibitors and neural markers, we demonstrate that Notch plays no major role in the general neurogenesis of larvae. Instead, we find Notch components expressed in nascent chaetal sacs, the organs that produce the annelid bristles. Impairing Notch signalling induces defects in chaetal sac formation, abnormalities in chaetae producing cells and a change of identity of chaeta growth accessory cells. This is the first bilaterian species in which the early neurogenesis processes appear to occur without a major involvement of the Notch pathway. Instead, Notch is co-opted to pattern annelid-specific organs, likely through a lateral inhibition process. These features reinforce the view that Notch signalling has been recruited multiple times in evolution due to its remarkable ‘toolkit’ nature.

## 1. Background

Since developmental biology met molecular and cellular biology, considerable efforts have been deployed in understanding signalling pathways function, modularity, architecture and later evolution. Among them, the Notch pathway has been especially investigated in these frameworks. As a direct juxtacrine signalling system, it provides a mechanism for short-range, localized signalling between directly apposing cells in a process called ‘trans-activation’ [1–5]. Upon binding of a ligand (Delta or Jagged) displayed by a neighbouring cell, the Notch transmembrane receptor is cleaved by the γ-secretase complex (presenilin-nicastrin-APH1-PEN2) resulting in the release of the Notch intracellular domain (NICD) into the cytoplasm (additional cleavage of Delta by the γ-secretase complex has been also described in vertebrates [6]). After its translocation to the nucleus of the receiving cell, NICD interacts with the CSL (CBF1, suppressor of hairless (Su(H)), Lag-1)/Ncor/SMRT/histone deacetylase transcriptional complex and activates the transcription of target genes. The same ligands can also interact with the receptor Notch within the same cell (in *cis*). The Notch pathway functions with a number of ‘core component’ proteins, whose evolutionary emergence has been studied in detail [7]: it was probably already functional as early as in the last common ancestor of all metazoans, but has its roots anchored deep in the eukaryote tree as some non-metazoan unikonts display members of these components.

Expression and functional studies of Notch pathway components during development have been performed in few metazoan species, mostly in deuterostomes and ecdysozoans. In these two lineages, the remarkably pleiotropic Notch pathway affects cell fate choices associated with lineage segregation, cell differentiation, cell proliferation or apoptosis in many tissues and developmental stages [8–11]. The extreme versatility and pleiotropy of Notch signalling seems at first glance to make the task of reconstituting an evolutionary history of its involvement in metazoan development rather daunting. In many ways, the Notch pathway could be described as a developmental ‘Swiss army knife’, a generic tool that has been co-opted multiple times in evolution, in various animal lineages, for unrelated or sometimes convergent functions in development. However, comparisons of the roles of Notch between insect and vertebrate have brought to light interesting similarities that have led to speculations about an ancestral involvement of Notch signalling in neurogenesis [12].

Notch is crucial in the early patterning of neurogenesis both in the fruit fly and in vertebrates. The study of *Drosophila* neurogenesis in particular has led to uncovering a mechanism called ‘lateral inhibition’. In the fly embryo, the central nervous system originates from delaminating neural progenitors [13,14], called neuroblasts. These divide asymmetrically to give birth to further dividing neuron precursors (the ganglion mother cells). Neuroblasts are active during both embryogenesis and larval development, playing the role of neural stem cells. Delaminating neuroblasts are initially specified in clusters of ectodermal epithelial cells, called ‘proneural clusters’. These cells are initially bipotent, giving either a neuroblast or, for the majority of them, epidermal cells. The cell that will eventually become a neuroblast starts to express higher levels of the ligand transmembrane protein Delta, which binds to the Notch transmembrane protein present at the surface of neighbouring cells. In these neighbouring cells, the activation of the Notch pathway results in the downregulation of both *Delta* and neurogenic gene expressions, thus inhibiting the neural fate. In vertebrate embryos, early neurogenesis processes take place mostly in the neural tube after it has completed its closure. In the thick pseudostratified epithelium of the neural tube, a gradient of *Notch1* expression, maximal apically, keeps neurogenic specification on the apical/ventricular side [15], while differentiating neuron bodies migrate to the basal side. On the apical side, where both symmetrical and asymmetrical cell divisions take place, lateral inhibition by Notch/Delta regulates the pace of neuronal specification, maintaining a pool of neural progenitors [16,17].

This mechanism of lateral inhibition by Notch/Delta has been evidenced in a number of contexts, such as hair cell formation in the inner ear [18]. Interestingly, the inner ear development is also mediated by another mechanism of the Notch pathway called lateral induction, through the alternate ligand Jagged1. Such mechanisms, finely modulated by *cis*-interactions, exemplify the notion of the Notch pathway as a general and versatile developmental tool that could possibly have been co-opted for playing similar functions. However, there is now considerable evidence that both neurogenesis [19] and nervous system centralization [20] in protostomes and vertebrates share a common origin. In this context, Notch is often seen as a pathway ancestrally involved in neurogenesis patterning in bilaterians. This hypothesis receives support also from cnidarians that have a dispersed nervous system organization [21]. In the sea anemone *Nematostella*, the Notch pathway appears to play a role in regulating the abundance of differentiating nerve cells, even if the exact roles of specific pathway components remain disputed [22–24]. This pushes the involvement of Notch in nervous system formation back to ancestors of eumetazoans (cnidarians + bilaterians), at the very evolutionary origin of the nervous system.

While plenty of studies focused on the various aspects of Notch pathway mechanisms and regulations among deuterostomes and ecdysozoans, the third large group of bilaterians, i.e. the lophotrochozoans, has been barely studied. The Notch pathway architecture and functions have been investigated so far in two annelid species only. In the leech *Helobdella robusta*, the Notch pathway has been proposed to be involved in posterior elongation and segment formation [25,26]. In *Capitella teleta*, the expression patterns of the Notch pathway components (discussed later in the article) in brain, foregut, chaetal sacs and posterior growth zone suggest a possible involvement of this pathway in brain development, chaetogenesis and segmentation processes [27].

In this study, we analysed the functions of the Notch pathway in the marine annelid *Platynereis dumerilii*, a leading lophotrochozoan model species for evolution and developmental biology. *Platynereis dumerilii* is considered to be a ‘short branch’ organism that has kept a number of ancestral-looking characters at the genomic [28], anatomical and developmental levels [20,29,30], making it a highly attractive model system for understanding the origin and diversification of these traits. *P. dumerilii* displays an indirect form of development (for a review, see [31]): embryogenesis first gives rise to a minute spherical trochophore larva with a small number of differentiated larval structures, including a precise arrangement of a few neuronal and sensory cells, such as the apical organ or the posterior pioneer neurons [20,32,33]. Later than this larval nervous system, a wave of massive ‘adult’ neurogenesis takes place in the mid-trochophore stage, at two different locations: the episphere at the animal pole, which will give rise to the anterior brain of the worm, and the ventral neural ectoderm, which will give rise to the ganglia of the ventral nerve cord (VNC) [20]. Ventral neurogenesis takes place in thickening stratified or pseudo-stratified epithelia where mitoses happen on the apical side and neuron differentiations occur on the basal side, similar to what has been described for the vertebrate neuroepithelium [20,34]. In addition, *Platynereis* neurogenesis involves a number of the same transcription factors that have already been identified in insect and vertebrate models such as the bHLH *NeuroD, neurogenin* and *achaete-scute*-related genes [33].

The aim of this study is to explore the roles of the Notch pathway in *Platynereis* and to shed light on the ancestral role(s) of Notch in lophotrochozoans and bilaterians (we do not describe here a potential role in posterior elongation and segment formation that require further study in the juvenile worm). Taking advantage of small molecule inhibitors, we show here that the Notch signalling pathway does not seem to play a major generic role in the early processes of larval or ‘adult’ neurogenesis in the annelid, even though roles in the formation of a few specific head and trunk neurons cannot be excluded. Instead, at the time when neurogenesis happens, we find a role of Notch in the correct patterning of the cells of chaetal sacs, likely through a lateral inhibition mechanism. Chaetal sacs are the larval and adult organs that are responsible for the formation of chaetae, i.e. locomotory and mechanosensory bristles of the worm. Our results thus challenge the view that the Notch pathway is necessarily involved in nervous system patterning in the same way at a eumetazoan scale. Our data rather support the view of co-options in regulating cell fate specification in multiple contexts.

## 2. Results

### 2.1. Notch pathway core components identification: a lophotrochozoan focus

**Figure 1.**
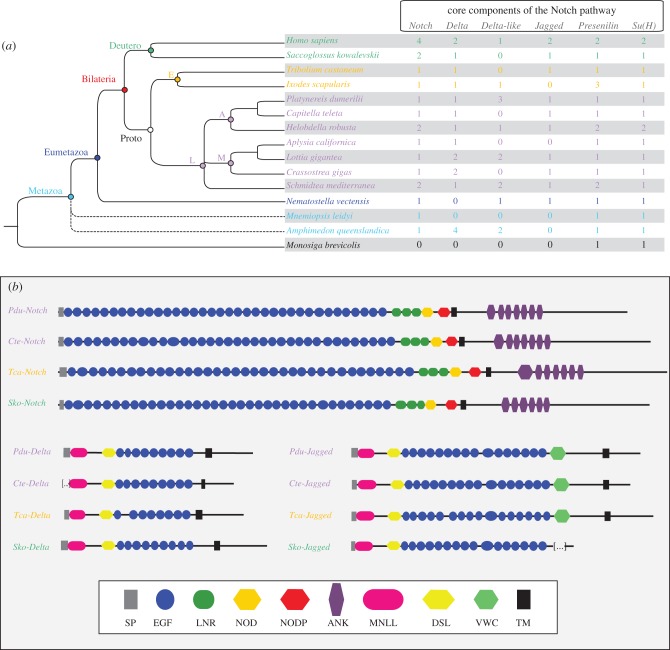
Core components of the Notch pathway in metazoans. (*a*) The presence/absence and number of gene copies at a metazoan scale for the Notch pathway main components. Fifteen species representatives of the metazoans, with a lophotrochozoan focus, are included. Dashed lines show the unresolved phylogenetic positions for sponges and ctenophores. (*b*) Domain arrangement of Notch, Delta and Jagged proteins in four bilaterian species are schematized. See figure inset for the domain legends. *Pdu-Notch* presents 36 EGF repeats, three LNR, one NOD domain, one NODP domain and seven ANK repeats. For *Pdu-Delta* and *Pdu-Jagged,* we detected the MNLL region, a Delta/Serrate/Lag (DSL) domain (which mediates binding to Notch receptors in bilaterians) and a series of EGF repeats (nine for *Pdu-Delta* and 16 for *Pdu-Jagged*). In addition to these domains, *Pdu-Jagged* also contains a Von Willebrand factor C domain (VWC) characteristic of Serrate/Jagged proteins. The choanoflagellate is in black, the sponge and ctenophore are in light blue, the cnidarian is in blue, lophotrochozoans are in purple, ecdysozoans are in orange and deuterostomians are in green. Deutero, deuterostomes; Proto, protostomes; E, ecdysozoans; L, lophotrochozoans; A, annelids; M, molluscs.

Exhaustive searches on the genome and several transcriptomes of *P. dumerilii* led us to identify core components of the Notch pathway i.e. the receptor Notch (*Pdu-Notch*, already identified [28]), the two ‘classical’ ligands *Pdu-Delta* and *Pdu-Jagged*, *Pdu-Presenilin* (component of the γ-secretase complex) and the transcription factor suppressor of hairless (*Pdu-SuH*; figure 1*a*; electronic supplementary material, figure S1A). In addition, we found three *Delta-like* genes (i.e. lacking the minimal domain arrangement defined below). We also identified the intracellular regulator *Nrarp* (*Pdu-Nrarp*), a gene encoding a small ankyrin (ANK)-repeat protein that is part of a negative feedback loop that attenuates Notch activity in vertebrates [35], the antagonist *Numb* (*Pdu-Numb*) and the post-translational modifier *Fringe* (*Pdu-Fringe*) (electronic supplementary material, figure S1A). *Platynereis* Delta, Jagged and Notch possess conserved domain arrangements that are very similar to other bilaterian species and likely to be ancestral in the bilaterian lineage (figure 1*b*). Interestingly, we identified two splice variants for *Pdu-Delta* (*Pdu-Delta^tv1^* and *Pdu-Delta^tv2^*; electronic supplementary material, figure S1B). In *Pdu-Delta^tv2^*, retention of the last intron led to a shorter sequence lacking the final short ATEV peptide. Despite extensive and specific searches, no other typical Notch, Jagged or Delta proteins have been evidenced.

The origin and evolution of the components and auxiliary factors of the Notch pathway have previously been investigated at a large scale [7]. We performed here a detailed search of Notch pathway core components in metazoan species representative of all main metazoan lineages (Deuterostomia, Ecdysozoa, Lophotrochozoa, Ctenophora, Cnidaria and Porifera), but surveyed more extensively lophotrochozoan species as they have been undersampled in earlier studies due to a lack of available genomes. Data comparisons among lophotrochozoan, ecdysozoan and deuterostomian species are of special interest in order to reconstruct bilaterian ancestral states (figure 1*a*). We identified a single unambiguous Notch orthologue in all metazoans species investigated, except in vertebrates, in the enteropneust *Saccoglossus kowalevskii*, in the annelid *H. robusta* and in the planarian *Schmidtea mediterranea.* A more complicated situation emerged for the evolution of Delta and Jagged proteins. In all species but the ctenophore, several Delta-related proteins with various domain compositions are identified. One particular domain composition (MNLL-DSL-(9)xEGF-TM-ATEV), in which not only epidermal growth factor (EGF) motif numbers but also their specific spacings are conserved, presumably corresponds to an ancestral Delta protein that was already present in the bilaterian ancestor [36]. Our study supports this interpretation; indeed genes corresponding to this proposed ancestral organization are present in several bilaterian species we sampled. In addition, the EGF domains generally follow the pattern of repeat spacings and cysteine residue spacings noticed previously [36] (electronic supplementary material, figure S1C). As noted above, a conserved alternative splicing upstream of the ATEV peptide is found in both *Platynereis* and vertebrate proteins, which suggests that this particular splicing is as ancient as the bilaterian ancestor. In addition to these ancestral Delta proteins, a number of bilaterian genomes code for ‘Delta-like’ proteins (three in *Platynereis*, two in *H. robusta*, *S. mediterranea* and *L. gigantea*, one in *I. scapularis*, and the human Dll3), displaying domain variations, the most common being the loss of the ATEV motif and of a number of EGF repeats. There is no indication that orthology relationships can be traced back to the bilaterian ancestors for any of these Delta-like proteins. Jagged proteins are absent outside eumetazoans (alternative scenarios for their emergence have been already discussed [7]) and have been lost or diverged beyond recognition in some bilaterians, such *I. scapularis* and *Aplysia californica* (figure 1*a*).

Phylogenetic analyses of metazoan *Delta* and *Jagged* ligands (electronic supplementary material, figure S1A) are based on a very limited length of sequences: only the domains corresponding to the MNLL and Delta/Serrate/Lag (DSL). Yet, the limited resolution these trees show is compatible with the interpretations given above. Briefly, phylogenetic analyses show that *Pdu-Delta* and *Pdu-Jagged* are included in robust Delta and Jagged clades, respectively. Sponge *Deltas* are clustered and form the sister group of the eumetazoan Delta + Jagged groups (approximate likelihood-ratio test (aLRT) > 0.90). Inside the Delta and Jagged clades, relationships are not fully resolved but *Platynereis* and *Capitella* sequences always group together. We thus tried unsuccessfully to infer the evolutionary origin of the three *Delta-like* genes found in *Platynereis* (electronic supplementary material, figure S1A). The phylogenetic relationships between *Platynereis* Notch receptor and other metazoan receptors are provided in the electronic supplementary material, figure S1A. As previously noticed [7], the relationships among Notch receptors are not perfectly well resolved and do not follow the metazoan species tree. However the orthology of the *Platynereis* Notch is not questionable (electronic supplementary material, figure S1A).

To sum up, core components of the Notch pathway in the lophotrochozoan *Platynereis* appear to be very similar to the ancestral situation of bilaterians. Therefore, investigating its functions in *Platynereis* is of special relevance to draw evolutionary conclusions.

### 2.2. The components of the *Platynereis* Notch pathway are not generally expressed in neurogenic tissues but they are expressed in forming chaetal sacs

**Figure 2.**
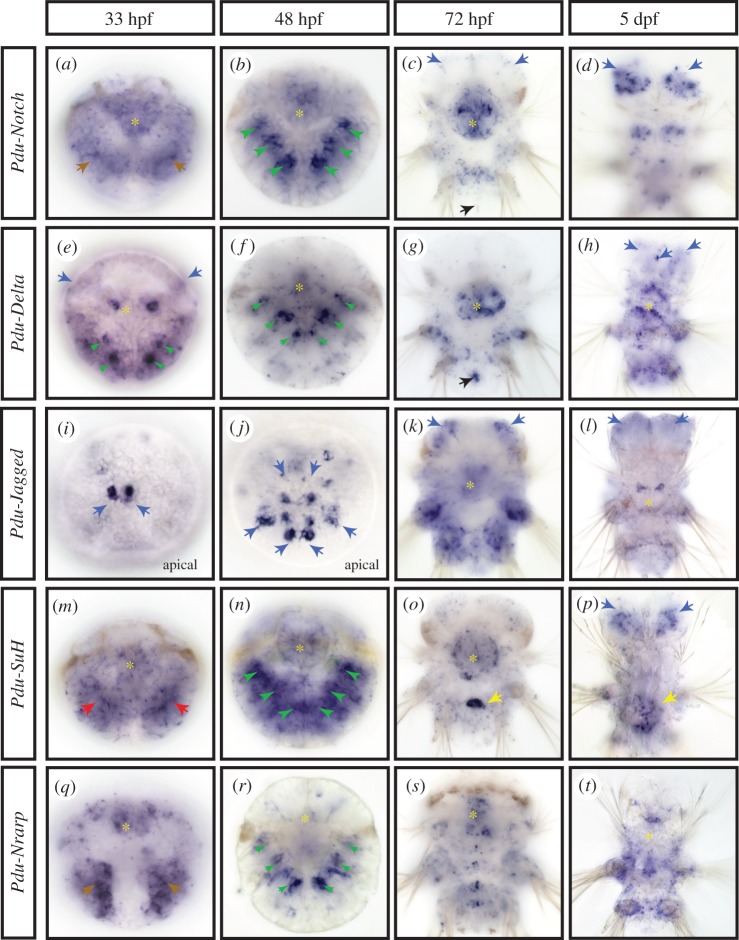
Expression patterns of the main components of the Notch pathway during *Platynereis* larval development. WMISH for *Pdu-Notch* (*a–d*)*, Pdu-Delta* (*e–h*)*, Pdu-Jagged* (*i–l*), *Pdu-Su(H)* (*m–p*) and *Pdu-Nrarp* (*q–t*)*,* are shown at four larval stages (33, 48, 72 hpf and 5 dpf). All panels are ventral views (anterior is up) except (*i* and *j*) that are apical (dorsal is up). Yellow asterisks mark expression in the stomodeum/forming pharynx. Brown arrows indicate an expression in the ectoderm, green arrowheads in the chaetal sacs, blue arrows in brain cells, red arrows in the mesoderm, black arrows in the putative mesoteloblasts and yellow arrows in a cluster of midgut cells.

We analysed by whole-mount *in situ* hybridization (WMISH) the expression patterns of five genes, *Pdu-Notch, Pdu-Delta, Pdu-Jagged, Pdu-SuH* and *Pdu-Nrarp* (figure 2). We targeted developmental stages when mass adult neurogenesis is taking place: at 33 hpf (hours post-fertilization; mid trochophore), the anterior episphere (mostly the future brain of the worm) and the ventral ectoderm (the future nerve cord) start to thicken and numerous mitoses occur superficially; at 48 hpf (late trochophore), neuronal differentiation has started and cell proliferation becomes more localized along the midline; at 72 hpf (three segment nectochaete larva), many brain and nerve cord neurons have differentiated but cell proliferation and thus neurogenesis continue locally and superficially; at 5 dpf (days post-fertilization; late nectochaete larva), cell proliferation and differentiation continue in the brain [20,34,37,38]. The Notch core component expression patterns can be categorized in three main structures of the larva: the chaetal sacs, some brain cells including the apical organ and the stomodeum.

All genes but *Pdu-Jagged* are expressed in specific structures called chaetal sacs. The chaetal sacs are responsible for producing chaetae, the retractile chitinous bristles displayed by the annelid leg-like appendages (parapodia) that help in locomotion. Chaetal sacs appear by invagination of ectodermal pockets [39]. In the late trochophore stage (48 hpf), when the chaetal sacs are fully formed and producing the first chaetae, *Pdu-Notch, Pdu-Delta* and *Pdu-Nrarp* are found in two bilateral ensembles of six patches of expression (figure 2*b,f,n,r*; green arrowheads). These lateral patches coincide with the 12 chaetal sacs of the larvae: two per future hemi-segment, six in the future neuropodia and six in the future notopodia (the ventral and dorsal moieties of the parapodia). All three gene expressions appear a little stronger in the ventral sacs (figure 2*b,f,n,r*; green arrowheads). *Pdu-Delta* expression is more restricted compared with *Pdu-Notch* and *Pdu-Nrarp* (figure 2*f*, green arrowheads) in a few deep cells of the chaetal sac and is observable early (at 33 hpf) in four to six bilateral and internalized patches of cells (figure 2*e*, green arrowheads). *Pdu-Su(H)* expression while being widespread is more intense in the chaetal sac areas at 48 hpf (figure 2*n*, green arrowheads).

Most of the genes studied (all but *Pdu-Nrarp*) are also expressed in different populations of brain cells and/or apical organ cells (a sensory structure composed of neurons and ciliary tuft [40] and present in many marine invertebrate larvae [32]), at several stages. Indeed, at 33 hpf, *Pdu-Delta* is expressed in few lateral brains cells (figure 2*e*, blue arrows), while *Pdu-Jagged* is found in two small groups of different brain cells (figure 2*i*, blue arrows, apical view). At 48 hpf, the *Pdu-Jagged* expression pattern expands in the brain, and forms several rows of cells inside and surrounding the apical organ (figure 2*j*, blue arrows, apical view; electronic supplementary material, figure S2A, internal dotted circle). Co-localization experiments reveal that some of those *Pdu-Jagged* *+* cells in the apical organ are serotoninergic, FMRFAmidergic and RYa peptidergic neurons (electronic supplementary material, figure S2A (v to x), internal dotted circle, purple arrow). Other brain cells expressing *Pdu-Jagged* are cholinergic neurons (electronic supplementary material, figure S2A (y), orange arrows). Later, at 72 hpf, the Notch receptor and the two ligands are expressed in several populations of brain cells (figure 2*c,k*, blue arrows; electronic supplementary material, figure S2A (b to d)). Co-localization with the c-amidated dipeptide RYa neuropeptide antibody [41] allows us to identify a small number of peptidergic neurons among the *Pdu-Notch*+ brain cells (electronic supplementary material, figure S2A (g to h), orange arrow). Similarly, *Pdu-Delta* *+* cells are most probably but not exclusively FMRFAmidergic, FLAmidergic and cholinergic neurons (electronic supplementary material, figure S2A (i to o), internal dotted circle, purple arrows). *Pdu-Delta* and *Pdu-Notch* are also co-expressed in two bilateral patches of brain cells of unknown identity (electronic supplementary material, figure S2A (e), orange arrows). At this stage, *Pdu-Jagged* expression is wider in the brain, where it is broadly co-expressed with *Pdu-Notch* but not *Pdu-Delta* (figure 2*k*, blue arrows; electronic supplementary material, figure S2A (d and f)). In addition, *Pdu-Delta* is found in pyramidal cells of the apical organ also expressing *Pdu-Notch* (electronic supplementary material, figure S2A (e), internal dotted circle, purple arrow). At 5 dpf, a long time after the main wave of neuronal differentiation occurred, widespread expression of *Pdu-Notch* appears in the brain (figure 2*d*, blue arrows). At this stage, *Pdu-Delta, Pdu-Jagged* and *Pdu-Su(H)* have also an expression in a few brain cells (figure 2*h,l,p* blue arrows).

Remarkably, all genes are expressed in the stomodeum and/or pharynx at several stages, either broadly (*Pdu-Notch*) or in few specific cells (*Pdu-Delta*; figure 2, yellow asterisks). Finally, at 72 hpf, *Pdu-Notch* and *Pdu-Delta* expressions are found in posterior internal cells that correspond to the mesoteloblasts (figure 2*c,g* black arrows), as evidenced by the coexpression with *Vasa* and *Smb*, two stem cell markers [42] (electronic supplementary material, figure S2B (b to j, yellow arrows)).

We thus find no general expression of Notch core components in tissues that are undergoing the main wave of neurogenesis between 33 and 55 hpf. Strikingly, we find no evidence of expression patterns resembling the grid-like expression of *Delta* genes in the ventral neural ectoderm of arthropods [43–46] or of the ‘salt and pepper’ expression in the neural tube of vertebrates [47]. Instead, *Delta* expression is limited at these stages and later ones to a few putative neuronal precursor cells, indicating a restricted role in the specification of a limited number of specific neurons. This surprisingly suggests that the Notch pathway may not be generally involved in *Platynereis* neurogenesis. Conversely, the prominent expression of Notch core components in chaetal sacs suggests a function in *Platynereis* chaetal sac patterning.

### 2.3. Notch pathway chemical disruption in *Platynereis*: initial characterization of effects

Although the core components of the Notch pathway apparently do not show general expression patterns associated with *Platynereis* larval neurogenesis, we cannot exclude that their expression levels are below the detection level of our WMISH experiments. To test the role of Notch in central nervous system (CNS) formation and chaetogenesis, therefore, we used three pharmacological agents: DAPT, LY-411575 and RO-4929097, all of which are inhibitors of the γ-secretase complex [48]. These drugs act by preventing the third cleavage of the Notch receptor, thereby blocking the nuclear relocalization and the activity of NICD as a transcriptional activator in the receiving cell. A large number of larvae (150 at least) were treated in each experiment. As adult neurogenesis and chaetogenesis overlap broadly in time, we applied drugs during a time window encompassing both processes: 24–48 hpf (Time window 1, table 1). During this time period, massive neural ectoderm proliferation occurs, the neural epithelium thickens considerably and neuronal differentiation starts, with the first elements of the adult neurite scaffold being put in place [34]. The chaetal sacs also start to form from 32 hpf onwards and the production of chaetae starts around 40 hpf [39].

**Table 1.** Types of treatment performed to study specifically chaetogenesis and neurogenesis processes. Four time windows were chosen. Time window 1 encompasses both processes and gives a general view of the potential functions of the Notch pathway in the annelid *Platynereis*. Time window 2 allows targeting the cell fates of chaetal sacs. Time window 3 encompasses the larval neuron formation. Time window 4 encompasses ‘adult’ neurogenesis. For each time window and drug concentration, the phenotype and results observed at several stages are mentioned. VNC, ventral nerve cord; conc., concentration.

drug conc.	Time window 1: 24–48 hpf		Time window 2: 20–48 hpf		Time window 3: 12/16–24 hpf		Time window 4: 30–48 hpf	
	stages	phenotypes	stages	phenotypes	stages	phenotypes	stages	phenotypes
LY-411575 1 µM	48 hpf	follicle cells markers expression decreased; neurogenesis markers expression maintained	48 hpf	overexpression of chaetoblast marker; presence of abortive chaetoblasts	24 hpf	*Elav* expression in maintained in 9 neurons	72 hpf	normal number of brain cells/ number of VNC neurons slightly enhanced
	72 hpf	absence of chaetae/normal chaetoblasts; normal VNC	72 hpf	absence of chaetae; development slightly delayed	72 hpf	absence of chaetae		
	6 dpf	absence of chaetae/normal VNC						
RO-4929097 2 µM	48 hpf	follicle cells markers expression decreased; neurogenesis markers expression maintained	48 hpf	absence of chaetae/very few abortive chaetoblasts				
	72 hpf	absence of chaetae/normal chaetoblasts; normal VNC	72 hpf	absence of chaetae				
	6 dpf	absence of chaetae/normal VNC						
RO-4929097 30 µM	48 hpf	absence of chaetae/development delayed	48 hpf	abnormal expression of chaetoblast marker; presence of abortive supernumerary chaetoblasts	24 hpf	*Elav* expression is maintained in 9 neurons	72 hpf	normal number of brain cells; development delayed
	72 hpf	absence of chaetae/development delayed	72 hpf	absence of chaetae; development delayed	72 hpf	absence of chaetae; development delayed		
	later	dead						
DAPT 40 µM	48 hpf	follicle cells markers expression decreased; neurogenesis markers expression maintained						
	72 hpf	absence of chaetae/normal chaetoblasts; normal VNC						
	6 dpf	absence of chaetae/normal VNC						

**Figure 3.**
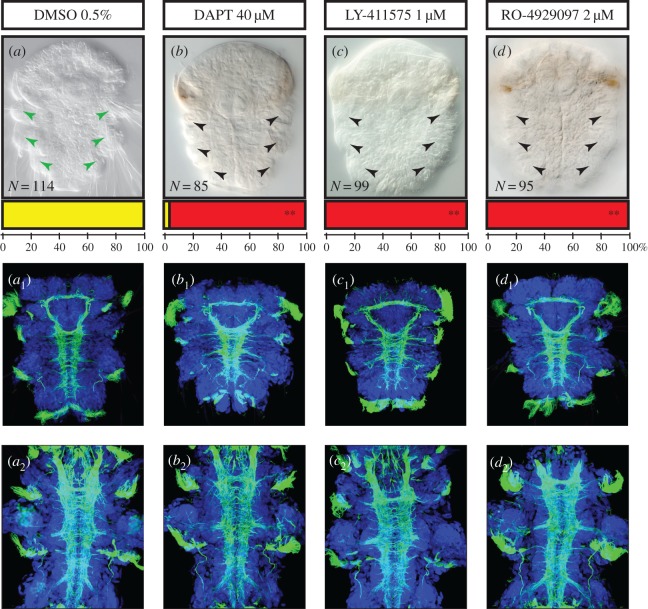
Inhibition of γ-secretase induces defects in bristle formation but no major nervous system phenotype. Ventral views of whole nectochaete larvae (72 hpf) and 6 dpf worms are shown (anterior is up). Larvae were incubated with DAPT (40 µM, *b* to *b*_2_), or LY-411575 (1 µM, *c* to *c*_2_), or RO-4929097 (2 µM, *d* to *d*_2_) in DMSO or in DMSO only (control group, *a* to *a*_2_) from 24 to 48 hpf. Larvae treated with the three drugs targeting the Notch pathway display a clear reduction of the bristles (*a–d*). Phenotypic classes were scored as ‘presence of chaetae’ (yellow) and ‘absence or abnormal chaetae pattern’ (red). Bars at the base of each image represent the percentages of larvae in each phenotypic class. Double asterisks indicate the highly significant differences (Student's test *p* < 0.01) between the mean numbers of affected versus unaffected larvae in the control and treated groups (sample sizes are indicated on the figure). Antibody labelling against acetylated tubulin (green) show the axon scaffold of the VNC at 72 hpf (*a*_1_ to *d*_1_) and 6 dpf (*a*_2_ to *d*_2_). Hoechst nuclear staining is in blue. Larvae treated with the three drugs display no gross defect in axon guidance, commissural projections or connectives in both stages.

We first tested different concentrations of drugs to determine whether they cause characteristic defects. We considered the lowest concentrations (40 µM DAPT, 2 µM RO-4929097 and 1 µM LY-411575) causing these defects to minimize the potential off-target effects and toxicity of the drugs. Importantly, LY-411575 precipitates above 2 µM, RO-4929097 and DAPT above 40–50 µM in seawater (as already noticed for DAPT [49]). Only RO-4929097 thus causes defects at a concentration well below solubility problems. Morphological defects were then assessed at 72 hpf, when the three segments, the brain and the VNC are fully formed and all chaetae are externally visible [31], and at 6 days (figure 3).

Treated larvae showed limited but completely similar defects as far as gross morphology and behaviour are concerned. At 3 and 6 days, they are well elongated and segmented (figure 3) and show normal muscular contractions, swimming behaviour and phototactism. The swimming speed is faster than normal because treated larvae display very few or completely absent protruding chaetae (in a highly significant way, Student's test *p* < 0.01; figure 3*a–d*). Labellings of the neurite scaffold with an anti-acetylated alpha-tubulin antibody show no gross defects in the head and trunk nervous systems as connectives and commissures appear to form normally at 72 hpf and 6 days (figure 3*a*_1_–*d*_2_). Consistent results for treated embryos obtained with all three drugs (with different molecular structures but targeting the same molecule) clearly suggest that their action is specific and that limited off-target effects appear at the chosen concentrations. Nevertheless, to determine whether drug-induced cell death occurred, we used the terminal deoxynucleotidyl transferase dUTP nick end labelling (TUNEL) method and found just a few apoptotic cells in the treated embryos, as for the control (electronic supplementary material, figure S2C (*a* to *a*4)).

As *Notch* plays a role in vertebrates in maintaining stocks of undifferentiated progenitors, it could have also a general role in *Platynereis* in regulating cell proliferation. To test that, we assessed proliferation at 48 hpf using 5-ethynyl-2′-deoxyuridine (EdU) labelling [34] and quantified EdU-labelled cells in structures related to the nervous system (i.e. in the ventral neurectoderm and the episphere), but also in the whole embryo, stomodeum and chaetal sacs (*n* = 8 for each condition; electronic supplementary material, figure S2C (b to d)). We found few significantly different percentages of EdU + cells (Student's test *p* < 0.05), in treated embryos compared to the control, when looking at the whole embryo, the ventral neurectoderm or the episphere (electronic supplementary material, figure S2C (d)). Only three comparisons (between dimethyl sulfoxide (DMSO) and DAPT and LY-411575 for the ventral neurectoderm and between DMSO and LY-411575 for the episphere) appeared to give slightly more mitoses in the treated larvae, in a significant way. However, we observed that the total numbers of cells in the whole embryo, the ventral neurectoderm and the episphere (defined by quantifying DAPI+ cells) are similar in treated embryos and controls (electronic supplementary material, figure S2C (d)).

Together those results tend to indicate that the Notch pathway disruption does not affect considerably either the general VNC or brain formation, nor does it affect in a major way cell proliferation profiles in the ventral and anterior neurectoderm. On the contrary, the disappearance of chaetae rather supports a role in chaetal sac patterning. Those two aspects are extensively studied using specific time windows for treatments (table 1) depending on the process in the following sections.

### 2.4. Chaetoblast addition is progressive and their number is finely regulated

To unravel the role of the Notch signalling pathway in the chaetogenesis process, we decided to investigate in depth the development of the chaetal sacs of *Platynereis*. Chaetal sacs are composed of several pockets called follicles, each of which produces a single bristle [50]. Each follicle consists of one chaetoblast that builds the bristle by proximal addition of material, mostly chitin and four surrounding follicle cells arranged on top of each other along the chaetae [44] (electronic supplementary material, figure S3A) that possibly add more material along the bristle length. The bristle grows along the canal formed by follicle cells and finally outside of the parapodium surface [51]. Morphological and ultrastructural studies revealed that new follicles emerge by internalization of epidermal surface cells. A central cell, the future chaetoblast, sinks down into the epithelium, surrounded by several cells that will become follicle cells (electronic supplementary material, figure S3A).

**Figure 4.**
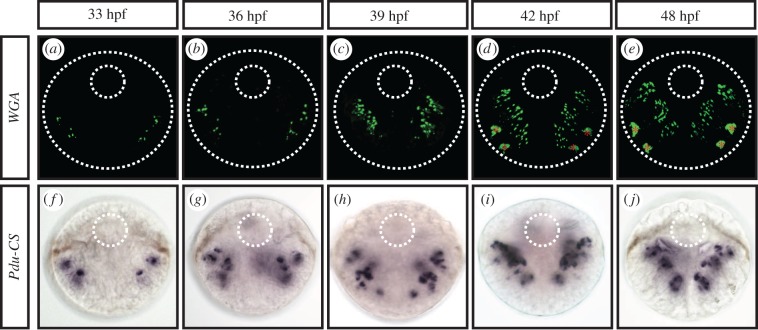
Chaetogenesis dynamics during *Platynereis* embryonic development. WGA staining reveals the arrangement and number of chaetoblasts during the course of development (33, 36, 39, 42 and 48 hpf) (*a–e*). Expression patterns of the chaetoblast marker *Pdu-CS1* show similar results (*f–j*). From 33 to 36 hpf, active chaetoblasts are present in only two segments (1 and 2) and then appears in segment 3 at 39 hpf. Around 42 hpf the chaetal sacs show a fixed number of follicles. All panels are ventral views (anterior is up). Red asterisks mark the artificial staining of glands at 42 and 48 hpf. Large dotted circles indicate the outline of the embryos, while the small dotted circles indicate the position of the stomodeum.

We used the properties of the wheat germ agglutinin (WGA), which binds strongly to the β-chitin [39], the main structural component of chitinous chaetae. We thus identified during larval development the position and number of chaetae and, at the proximal end of the bristle, chaetoblasts (figure 4*a–e*). We also investigated the expression pattern dynamic of *Chitin synthase 1* (*CS1*), a conserved gene encoding an enzyme crucial for chitin polymerization [52] and expressed specifically in *Platynereis* chaetoblasts [44] (figure 4*f–j*). Both stainings give similar results: chaetoblasts and their corresponding chaetae are present in increasing numbers from 33 to 42 hpf (figure 4; electronic supplementary material, figure S3B), first in segments 1 and 2, and then in segment 3. Chaetoblasts first appear near the surface of the larval ectoderm and then sink deeper in the larval body as the chaetal sacs themselves internalize. Chaetoblast numbers are specific for each chaetal sac, depending on segment and dorsal–ventral position (figure 4; electronic supplementary material, figure S3B).

**Figure 5.**
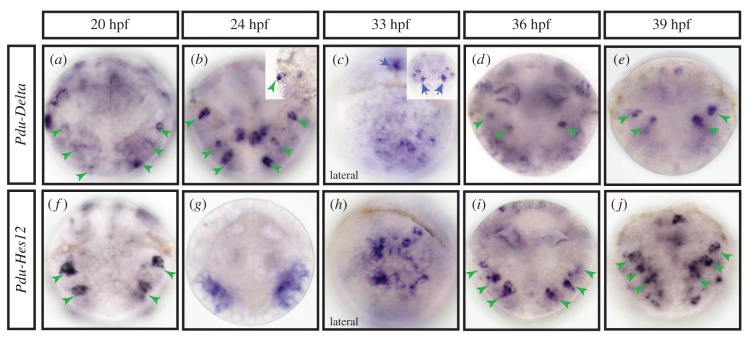
Expression patterns of chaetoblast markers during *Platynereis* early embryonic development. WMISH for *Pdu-Delta* (*a–e*) and *Pdu-Hes12* (*f–j*)*,* at five developmental stages (20, 24, 33, 36 and 39 hpf) are shown. All panels are ventral views (anterior is up) except (*c* and *h*) that are lateral. Blue arrows indicate an expression in brain cells, green arrowheads in the presumptive chaetal sacs, presumably in chaetoblast cells. From 20 to 33 hpf, scattered superficial cells are observed (*a–c*, *f–h*). Those cells start to get internalized between 33 and 36 hpf and reach their final position after 39 hpf (*d,e,i,j*). (*b*) Inset details the ectodermal nature of *Pdu-Delta+*cells and (*c*) inset shows an apical view of *Pdu-Delta* *+* brain bilateral cells.

When are chaetal sac cell identities defined? As some chaetoblasts start being functional as early as 33 hpf, their specification and initial internalization must have taken place quite early in larval development. We complemented these stainings with live imaging experiments using the plasma membrane vital dye FM-464 to observe early chaetoblast formation (electronic supplementary material, figure S3C). We detected the presence of ‘rosettes’ of ectodermal cells at the level of the future parapodia, made of one or two central bottleneck-shaped cells that appears to be in the course of internalization, surrounded by six or seven petal-shaped cells, already present at the surface of larvae at 27 hpf (electronic supplementary material, figure S3C (*a*, *a*′ and *a″*)). Later in the development deeper roundish structures appear revealing the outlines of the maturing chaetal sacs (electronic supplementary material, figure S3C (*b* to *d″*)). We investigated the very early expressions of potential chaetoblast precursor markers (i.e. *Pdu-Delta* and *Pdu-Hes12*; figure 5) and follicle cells markers (not shown). We found that both types of markers are expressed as early as 20 hpf. *Pdu-Delta* and *Pdu-Hes12* are expressed in scattered lateral superficial ectodermal cells (figure 5*a,f*, green arrowheads) in the parapodial field. Those cells are possibly the future chaetoblast cells, as suggested by their position and arrangement. Later, from 24 to 33 hpf, more cells express both genes in a ‘salt and pepper’ fashion in the whole parapodial field (figure 5*b,c,g,e* green arrowheads). While most of these cells are in a superficial position, some are located more internally, suggesting a possible mechanism of internalization. At 36–39 hpf, superficial expression decreases considerably while a few more internal cells continue to express both genes (figure 5*d,e,i,j*). As the fully formed chaetal sacs appear from 42 hpf on, *Pdu-Delta* is first restricted to a few deep cells in the chaetal sacs of segments 1 and 2 and later appears in segment 3 while *Pdu-Hes12* expression is found in all chaetal sacs.

### 2.5. Molecular signature and arrangement of the chaetal sac cell types

We then used the pattern registration technique recently developed for *Platynereis* larvae [52] to better unravel the mature follicle cell types' composition and arrangement. Pattern registration in the case of chaetal sacs does not allow a perfect one-cell resolution analysis because cells do not always display perfectly constant positions within the sacs. It is sufficient however to accurately visualize expression domains in three-dimensional views, to establish gross overlap of expression domains and to corroborate hypotheses of coexpression. Expression patterns of all Notch components expressed in chaetal sac cells (*Pdu-Notch, Pdu-Delta, Pdu-SuH* and *Pdu-Nrarp*) were thus scanned, aligned and averaged. We have shown previously [53] the expressions of two *Hes* (*Hairy/enhancer of Split*) genes (potential target genes of the Notch pathway in eumetazoans) in the chaetal sacs. *Pdu-Hes2* and *Pdu-Hes12* that are found in 12 patches corresponding to, respectively, a large proportion and a few internal cells of the chaetal sac, were also included in the database. As we wanted to compare those gene expressions with known markers of chaetal sacs cells, we also add the *Pdu-CS1* (a chaetoblast marker) and the *Pdu-Caml* (a follicle cell marker [39]) genes to this analysis. Finally, to better understand the architecture of chaetal sacs in trochophore larvae, we also used *Pdu-Twist*, expression of which spans mesodermal territories surrounding the chaetal sacs [54]. We used again the WGA labelling to identify the position of the chaetoblasts, located in the proximal-most part of each chaetal sac [39].

In the electronic supplementary material, figure S3D, we provided three different views of three-dimensional surface labelling (either expression patterns or WGA staining) rendering possible the understanding of the complex organization of the 12 chaetal sacs (six in the neuropods and six in the notopods) surrounded by mesodermal tissues (*Pdu-Twist*) in the 48 hpf larvae. *Pdu-Delta* expression is localized in the internal-most part of the sac (electronic supplementary material, figure S3D (*a*_4_, *b*_4_, *c*_4_, *d*_3_, *d*_4_)) while *Pdu-Notch* expression is more widespread in a large medial area of the sac (electronic supplementary material, figure S3D (*a*_3_, *b*_3_, *c*_3_, *d*_3_, *d*_4_)).

**Figure 6.**
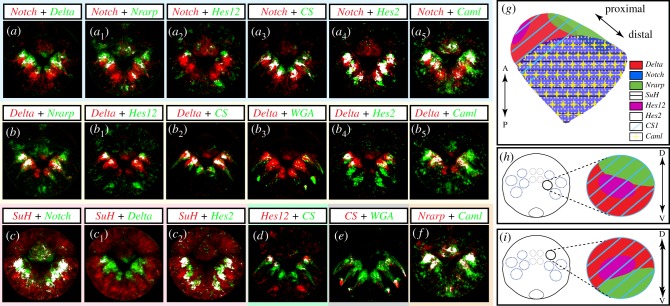
Molecular fingerprint of *Platynereis* chaetal sac cell types at 48 hpf. All images are confocal maximum z-projection of averaged expression patterns registered on an average larva. Ventral surface views of the whole larvae, anterior side up, are shown. White patterns reveal the colocalization of green and red pixels. (*a* to *a*_5_) *Pdu-Notch* expression slightly overlaps with *Pdu-Delta, Pdu-Nrarp* and *Pdu-CS1*, is mutually exclusive with *Pdu-Hes12* and is broadly co-expressed with *Pdu-Hes2* and *Pdu-Caml*. (*b* to *b*_5_) *Pdu-Delta* expression overlaps partly with *Pdu-Nrarp,* broadly with *Pdu-Hes12, Pdu-CS1* and WGA, and slightly with *Pdu-Hes2* and *Pdu-Caml*. (*c* to *c*_2_) *Pdu-Su(H)* is largely co-expressed with *Pdu-Notch* and *Pdu-Hes2* but not with *Pdu-Delta.* The chaetoblast marker, *Pdu-CS1,* is co-expressed with *Pdu-Hes12, Pdu-Delta* and WGA staining (*d*, *b*_2_ and *e*). *Pdu-Caml* and *Pdu-Nrarp* are broadly overlapping (*f*). The summary of expression patterns territories relative to a chaetal sac is provided in (*g*,*h*,*i*). (*g*) Schematic representation of a ventral chaetal sac with the gene expression territories mentioned, in a proximal–distal axis. See figure inset for the gene colour legends. The cartoons in (*h*,*i*) show apical view of a 48 hpf larva with the chaetal sacs represented by blue dashed circles. The black circles correspond to the location of the stomodeum. (*h*) Focus on an apical view of ventral chaetal sac gene expression territories. (*i*) Focus on a vegetal view of dorsal chaetal sac gene expression territories. Note the mirror-like expression patterns territories between the dorsal and chaetal sacs. A–P, antero-posterior axis; D–V, dorso-ventral axis.

We next tried to define the identity and molecular signature of the cells composing a chaetal sac by looking at the colocalization patterns of gene expression areas using registration (figure 6). With the WGA staining to localize the chaetoblasts, as well as the *Pdu-CS1* expression (figure 6*e*), we identified *Pdu-Delta* as a marker of at least some cells in the chaetoblast region (figure 6*b*_2_ to *b*_3_)*. Pdu-Hes12* + cells fall also in the *Pdu-CS1*+domain and are thus chaetoblast cells too (figure 6*b*_1_ and *d*). *Pdu-Notch* and *Pdu-Hes2* expression territories are broadly in the medial part of the sac, and probably correspond to several if not all follicle cells (figure 6*a*_4_). Their colocalization with the follicle cell marker *Pdu-Caml* supports this interpretation (figure 6*a*_5_). *Pdu-Nrarp* is co-localized partially with *Pdu-Delta, Pdu-Notch* and *Pdu-Caml*, and is expressed both in chaetoblasts and some follicle cells (figure 6*a*_1,_ *b* and *f*). Altogether those different combinations of markers allowed us to identify two domains of expression patterns among the sac: one proximal area (being *Pdu-Delta+, Pdu-Hes12+, Pdu-CS1+* and *Pdu-Nrarp*+) and one distal area (being *Pdu-Notch+, Pdu-Hes2+, Pdu-Su(H)+, Pdu-Caml+* and *Pdu-Nrarp*+) (figure 6*g*). In addition, *Pdu-Nrarp* expression pattern is polarized along the dorsal–ventral axis, but in an inverse disposition in notopodial versus neuropodial chaetal sacs (figure 6*h,i*).

### 2.6. Treatments with γ-secretase inhibitors suggest that Notch patterns *Platynereis* chaetal sac cell types

The morphological defects obtained through 24–48 hpf (time window 1, table 1) chemical disruptions (figure 3) as well as the continuous expressions of Notch pathway components, especially *Pdu-Delta* (figures 2 and 5), from the onset of chaetal sac formation to chaetae elongation strongly suggest an extensive role in chaetal sac pattern formation and chaetogenesis. We thus investigated the effects of Notch pathway chemical inhibition (Time window 1, table 1) on genes expressed in chaetal sacs to test whether Notch pathway component expressions themselves are affected (electronic supplementary material, figure S3E). Interestingly, *Pdu-Notch* is significantly downregulated (electronic supplementary material, figure S3E (*a* to *a*_3_); an expected effect, for example, see [55]), whereas *Pdu-Delta* seems not affected by any treatments (electronic supplementary material, figure S3E (*b* to *b*_3_)). Similarly, treatments also lead to drastic decreases of follicle cells marker expression (*Pdu-Nrarp, Pdu-Hes2* and *Pdu-Caml*) while chaetoblast markers (*Pdu-Hes12* and *Pdu-CS1*) are maintained (electronic supplementary material, figure S3E (*c* to *g*_3_)). As Notch pathway disruption does not affect cell proliferation profile and does not induce specific cell death in follicles and chaetal sacs (electronic supplementary material, figure S2C), the expression losses observed may be related to an abnormal differentiation of follicle cells.

**Figure 7.**
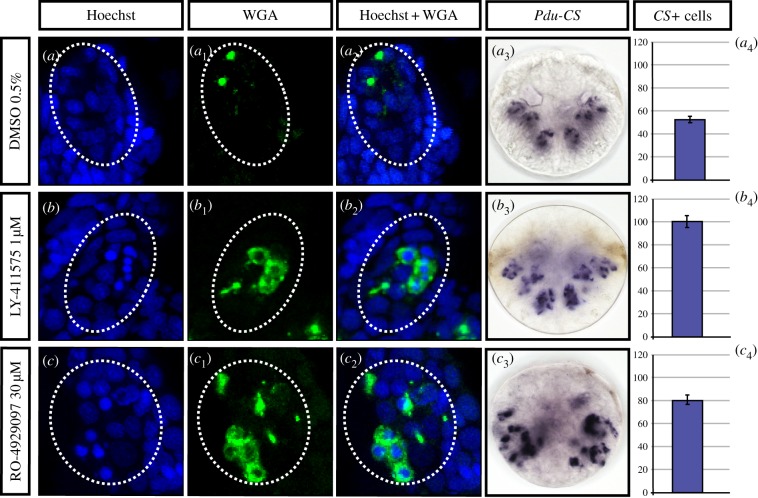
Early inhibitions of the Notch pathway induce supernumerary abnormal chaetoblasts within chaetal sacs. Embryos were incubated with LY-411575 (1 µM, *b* to *b*_4_), or RO-4929097 (30 µM, *c* to *c*_4_) in DMSO or in DMSO only (control group, *a* to *a*_4_) from 20 to 48 hpf. Treated larvae present several packed, intensely DAPI-stained nuclei surrounded by WGA-stained cytoplasm (*b* to *b*_2_, *c* to *c*_2_) instead of WGA spots neighbouring the chaetoblast nuclei (*a* to *a*_2_). The chaetoblast marker *Pdu-CS1* is overexpressed within the whole sacs in treated larvae instead of a packed internal region (*b* to *b*_2_, *c* to *c*_2_, and compare to *a*_3_ to *a*_4_). All panels are ventral views (anterior is up).

Notch pathway components (*Pdu-Delta, Pdu-Hes12, Pdu-Nrarp* and *Pdu-Hes2*) are expressed early in surface ectodermal cells, suggesting that they play a role in selecting cells that are going to differentiate into chaetoblasts or follicle cells, in a way that could involve lateral inhibition. We thus also performed early drug treatments (20–48 hpf, Time window 2, table 1) and assessed the resulting phenotypes on chaetoblast differentiation at 48 hpf, using WGA staining as well as the chaetoblast marker *Pdu-CS1* (figure 7). LY-411575 was used at 1 µM while RO-4929097 had been raised to a higher concentration (30 µM) to obtain similar results. DAPT used at the limit of solubility (40 µM), while affecting chaetae production as the two other drugs do, gave no phenotype related to chaetoblast differentiation (not shown). In both efficient treatments, our experiments revealed the presence of roundish, intensely DAPI-stained cells surrounded by cytoplasmic WGA-reactive chitin (figure 7*b* to *c*_2_). Many of these cells do not show however a WGA-stained bristle elongating from it, suggesting that although they have some characteristics of chaetoblasts, they are not capable of producing bristles. We thus call them ‘abortive chaetoblasts’. Chaetoblasts and abortive chaetoblasts are more numerous in treated embryos than in the control (figure 7*a* to *c*_4_), as highlighted by *Pdu-CS*+ expression and cell counting (figure 7*a*_4_, *b*_4_ and *c*_4_). Abortive chaetoblasts are located at dispersed, abnormal positions in the sacs, including close to the surface and presumably at the ‘normal’ locations of follicle cells in a control embryo. No recognizable chaetal sacs form in these early treated larvae, suggesting that the patterning of these structures is completely compromised, in sharp contrast with later treatments. The internalization process, linked to the chaetal sac patterning, is consequently presumably affected.

As we are not able to establish unambiguously that the early *Delta* expressions are exclusively linked to chaetoblast differentiation, we also tested the possible involvement of the Notch pathway in the formation or selection of neural progenitors that will give rise to the few pioneer neurons of the 24 hpf larvae [31]. We thus treated larvae with the drugs (same concentrations as before) from 12 and 16 to 24 hpf (to be sure to encompass the very first neuron formation, Time window 3, table 1) and assessed the numbers of neurons (revealed by the postmitotic neuron marker *Elav* [33]) in both treated and control embryos at 24 hpf. At 24 hpf, the number of *Elav*+ cells in the trunk is very precise (nine) and is not at all affected by any early treatments (electronic supplementary material, figure S4A).

In conclusion, the inhibition of the Notch pathway in an early time window (Time window 2) results in missformed chaetal sacs and supernumerary chaetoblasts, consistent with a lateral inhibition mechanism, while additional experiments should be performed in the future to firmly prove it. By contrast, early larval neurogenesis is not affected. Later treatments (window 1), or with more limited drug doses, result in abnormal gene expressions and possibly abnormal differentiation of follicle cells.

### 2.7. No major involvement of the Notch pathway in ventral neurogenesis and brain patterning is evidenced

**Figure 8.**
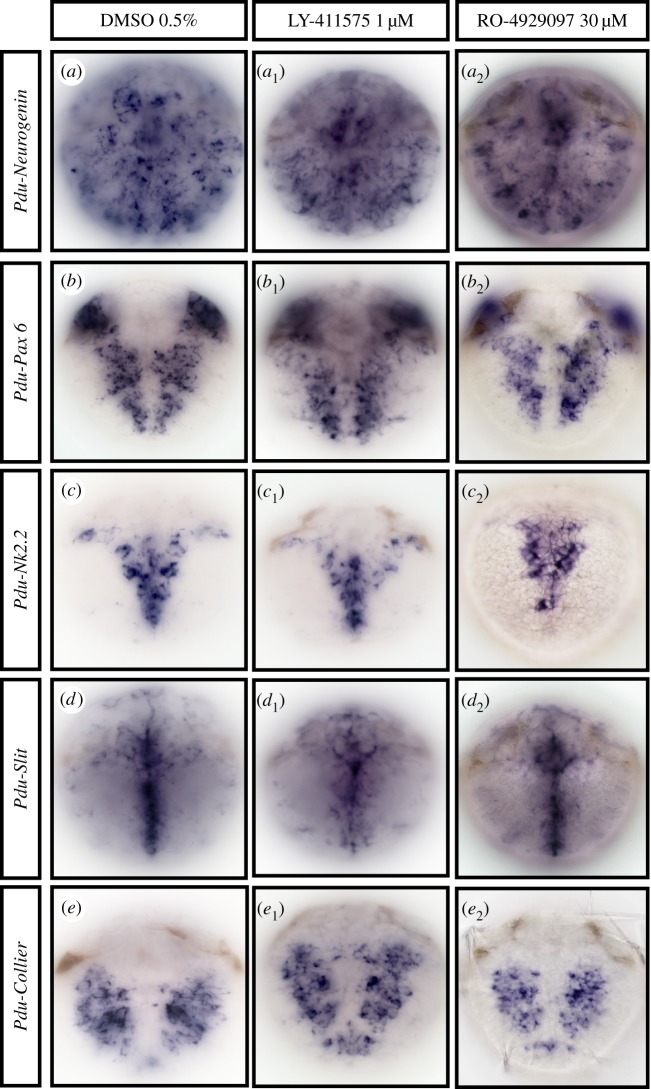
The Notch pathway does not regulate general ventral nerve chord patterning in 48 hpf *Platynereis* larvae. Ventral views of whole trochophore larvae (48 hpf) are shown (anterior is up). Larvae were incubated with LY-411575 (1 µM, *a*_1_ to *e*_1_), in DMSO or in DMSO only (control group, *a–e*) from 24 to 48 hpf, or RO-4929097 (30 µM, *a*_2_ to *e*_2_) from 30 to 48 hpf. WMISH, in control and treated larvae, reveals no gross defects in the expression of several genes involved in the process of neurogenesis after inhibition of the γ-secretase.

Although none of the components of the Notch pathway are obviously expressed in the forming VNC (figures 2 and 5) and the apparent morphology of the ventral nervous system does not appear much disturbed after γ-secretase inhibitor treatments (figure 3), we cannot exclude that Notch nevertheless could have a role in patterning and neuronal specification. We thus tested further the normal formation of the ventral nervous system by using several markers specific for different steps of neurogenesis and VNC patterning (*Neurogenin*, *Pax6*, *Nk2.2*, *Slit* and *Collier*) on 48 hpf-treated embryos for all drugs (Time window 1, table 1 and figure 8). The proneural gene *Neurogenin* (*Pdu-Ngn*) is exclusively expressed in neural progenitor cells (figure 8*a*) [33]. *Pdu-Pax6* and *Pdu-Nk2.2* are expressed in neurogenic columns and are key to medio-lateral patterning of neurons in vertebrates (figure 8*b,c*) [20]. *Pdu-Slit* is expressed in the ventral midline and is a key axon guidance factor in insects and vertebrates (figure 8*d*) [34]. *Collier* (*Pdu-Coe*), as a marker of neuronal differentiation, is expressed in differentiating and/or fully differentiated neurons (figure 8*e*) [56]. In concordance with the morphology and behaviour, we observed no expression pattern alteration in the forming CNS on treated larvae at concentrations (LY-411575 1 µM, RO-4929097, 30 µM, DAPT 40 µM; data not shown) that perturb chaetal sac patterning, either at gross levels or in the precise spatial patterns of these five genes (figure 8*a* to *e*_2_). In addition, no overlapping expression was observed between either *Pdu-Notch* or *Pdu-Delta* and *Pdu-SoxB*, *Pdu-Ngn* and *Pdu-Pax6,* central elements of the neurogenic network at eumetazoan scale [24] (electronic supplementary material, figure S4B).

**Figure 9.**
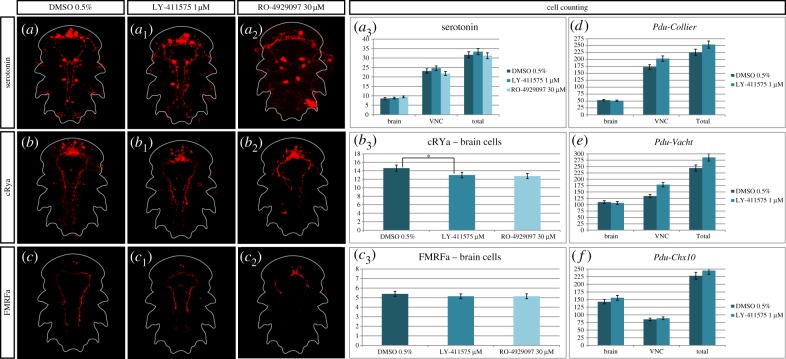
γ-Secretase inhibitor treatments, while possibly causing a slight increase in the number of neurons, do not change their overall differentiation patterns in 72 hpf *Platynereis* larvae. Ventral views of whole nectochaete larvae (72 hpf) are shown (anterior is up). Larvae were incubated with LY-411575 (1 µM, *a*_1_ to *c*_1_) or RO-4929097 (30 µM, *a*_2_ to *c*_2_) in DMSO or in DMSO only (control group, *a–c*) from 30 to 48 hpf and fixed at 72 hpf. Total number of neurons as well as specific populations of neurons (serotoninergic, FMRFAmidergic, RYa peptidergic, cholinergic and interneurons) in the VNC and the brain were counted in both control and treated larvae (*a*_3_ to *c*_3_, *d–f*). The serotoninergic, FMRFAmidergic and RYa peptidergic numbers of neurons are equivalent between control and treated larvae. The numbers of differentiated trunk neurons (*Pdu-collier*), as well as the cholinergic neurons (*Pdu-Vacht*) appear to be slightly increased after Notch inhibition (*d–f*) but a specific category of interneurons (*Pdu-Chx10*) remains stable.

While inhibiting Notch clearly does not disrupt patterning of the VNC at 48 hpf, we then tested the possibility that a Notch activity reduction may enhance neurogenesis in older embryos. We thus treated larvae (LY-411575 (1 µM) and RO-4929097 (30 µM)) from 30 to 48 hpf (Time window 4, table 1), a time window that encompasses the massive neurogenesis process in *Platynereis* [34] but prevents too drastic phenotypes. We assessed the phenotype at 72 hpf, at the cellular level (figure 9). We used several markers and antibodies of differentiated neurons or specific types of neurons (serotoninergic, FMRFAmidergic, RYa peptidergic, cholinergic and interneurons), and investigated the number of cells that are positive for each of them, in both control and treated larvae. Serotonin, Crya [41] and FMRFa antibody stainings reveal no gross defaults between the control and LY-411575 treated larvae, while the RO-4929097 treated larvae appear to be delayed in their development (*a* to *c*_2_). Manual counting of serotoninergic (*a*_3_), RYa peptidergic (*b*_3_) and FMRFAmidergic (*c*_3_) neurons shows no significant differences in both brain and VNC between the control and treated larvae (even for the delayed ones), except for one case (RYa brain cells are significantly reduced in the RO-4929097 condition compared to control). We then used three other markers of neurogenesis: *Pdu-Collier* for all the differentiated neurons, *Pdu-VAchT* for the cholinergic neurons [20] and *Pdu-Chx10* for a subset of interneurons [20]. Using registration, we obtained an averaged expression pattern for these genes in both control and LY-411575-treated larvae (not for RO-4929097 as the concentration used led to delayed larvae, preventing from a correct alignment during registration; electronic supplementary material, figure S4C). Using the Imaris software, we counted on these averages the number of positive cells for each gene in both the VNC and brain. This automatized approach for cell counting is highly linked to the *in situ* hybridization experiment efficiency and gives only a rough estimate of number of neurons. For all three genes, the number of neurons in the brain is almost equivalent between the control and treated larvae, while a few more interneurons are found after Notch inhibition (figure 9*d–f*). The number of interneurons is nevertheless constant in the VNC, while the cholinergic neurons and the overall differentiated neurons in general appear to be slightly more numerous in treated larvae. However, these numbers may be biased by the quality of the *in situ* hybridization and are in no way comparable to the drastic phenotypes observed in other models (in different contexts, see [57–60]).

## 3. Discussion

### 3.1. Evolution of the Notch pathway architecture: a lophotrochozoan focus

We investigated the evolution of the Notch pathway core components in metazoan genomes with a strong focus on lophotrochozoan species, and updated a previous analysis done at the eukaryotic level [7]. In a majority of species, a single *Notch* gene was found. The presence of supplementary copies of genes in *H. sapiens* is most probably due to the two whole-genome duplication events (2R) at the origin of vertebrates [61] while some lineage-specific duplications are also evidenced (for example, in *H. robusta* and *S. mediterranea*). The receptor Notch structure (domain composition and numbers of domain copies) is conserved in a large majority of cases, suggesting that the last common ancestor of bilaterians probably possessed a single Notch protein composed of 36 EGF repeats, three LNR repeats, NOD and NODP domains and seven ANK repeats. A more complex evolutionary history of the Delta/Delta-like proteins is suspected, but a single *Delta* gene was probably present in the urbilaterian ancestor with a structure (MNLL/DSL/9 EGF repeats/ATEV) that has been mostly conserved in extant bilaterians. The ATEV motif, which binds to PDZ domain proteins [62], is coded in most bilaterian *Delta* genes, while we identified in *Platynereis* a splice variant lacking this motif. Additional lophotrochozoan Delta-like proteins are found but their origin is uncertain. Drawing strong conclusions on the early metazoan history of the Delta-like ligand is difficult as non-bilaterian data are rather limited. At least one *Delta-like* gene was present in the last metazoan ancestor (with presumably multiple duplications events in the sponge lineages) but the structure of this ancestral gene cannot be firmly reconstructed from the available data (figure 1*a*; electronic supplementary material, figure S1D) [7]. In conclusion, the urbilaterian genome harboured well-defined *Notch*, *Delta* and *Jagged* genes. This situation is remarkably conserved in *Platynereis*, thus making it legitimate to discuss their functions in the worm in a bilaterian evolutionary context.

### 3.2. Is the Notch pathway ancestrally linked to central neurogenesis?

We think we have here gathered convergent arguments to rule out a major involvement of the Notch pathway in the early events of central neurogenesis in *Platynereis*, thereby contrasting with the situation known from insects and vertebrates:

— There is no major expression of the receptor *Pdu-Notch* or the ligand *Pdu-Delta* at the time when mass adult neurogenesis is taking place in the episphere (future brain) and the ventral neuroectoderm. Both genes are found expressed in sub-populations of cells in the brain (notably in the apical organ) but only at very late larval stages (72 hpf onward) when the bulk of brain neurons have already differentiated.
— Previously, we have shown the expression of two of the 13 *Hes* genes present in *Platynereis* (*Hes12* and *Hes13*) in the presumptive VNC during larval neurogenesis [53]. As *Hes* genes are sometimes considered as ‘canonical Notch effector genes’, their expression could indicate a limited involvement of Notch in the events of central neurogenesis. Nevertheless, none of them is found in a very broad pattern that would suggest a role in general neurogenesis [53]. Finally, larvae treated with γ-secretase inhibitors do not show any *Hes12* expression increase (electronic supplementary material, figure S3E). We thus feel confident that Notch does not act through any *Hes* gene (alone or in combination with another *Hes*) to pattern *Platynereis* VNC, while we cannot exclude that some *Hes* may play a role in more limited aspects such as the differentiation of specific neuron types. We also would like to stress the fact that the status of *Hes* genes as ‘core’ Notch pathway targets is the result of a historical over-simplification, based mostly on *Drosophila* studies. The Hes superfamily was shaped by many independent lineage-specific tandem duplication events leading to a high diversity of *Hes* members [53]. Consequently, a regulation of *Hes* by Notch should not be systematically expected.
— Treatments with three drugs preventing the normal cleavage of the Notch receptor do not result in major defects in the nervous system, when applied during the two time windows of neurogenesis (i.e. during the formation of the specific larval and pioneer neurons, and during the later mass adult neurogenesis events in the VNC and the brain). The normal patterning of the CNS is preserved as indicated by gene expressions. Near normal proliferation of both anterior and ventral neural ectoderm is maintained. Near normal numbers of neurons are differentiating and form nerve connections that seem unperturbed. We nevertheless observed a slight increase of cholinergic neurons in the VNC after drug treatments. *Pdu-VAchT* (the cholinergic marker used) is known to be also expressed in the peripheral nervous system (PNS) of *Platynereis* (J Béhague and P Kerner 2017, unpublished data). We thus cannot exclude a limited role of the Notch pathway in peripheral neuron differentiation. However, the persistence of normal peripheral nerves in treated larvae does not suggest a predominant role in PNS formation either.
— By contrast, the same three drugs are causing specific defects in organs where the core components of the Notch pathway are specifically expressed, the chaetal sacs. These defects seem to be compatible with the Notch pathway being involved in a process of lateral inhibition in the chaetal sacs.

The minor involvement of the Notch pathway in *Platynereis* generic neurogenesis comes as an important surprise and two alternative evolutionary scenarios can be proposed to interpret this striking fact:

(i) The Notch pathway may be ancestrally involved in neurogenesis and this role has been lost in ancestors of the *Platynereis* lineage. The ‘salt and pepper’ expression of proneuronal bHLH genes in *Platynereis* neurectoderm suggests that a process of selection of cells with neuronal fate occurs [33]. If the Notch pathway is not playing this role, one has to wonder how this process is performed and no alternate candidates are suspected so far.
(ii) The situation in *Platynereis* reflects the fact that the Notch pathway may not be ancestrally involved in neurogenesis. The Notch pathway could have been co-opted independently in ancestors of the arthropod and vertebrate lineages to perform analogous functions in the formation of nervous systems.

How plausible does each of these scenarios seem? The first scenario may be the most tempting as the view that the Notch pathway is probably ancestrally linked to neurogenesis is relatively widespread. However, careful examination of the available data leads to questioning the functional similarities that were initially discovered between insect and vertebrate central neurogenesis. In vertebrates, the Notch pathway plays a key role in maintaining a pool of neural stem cells during the progressive process of neurogenesis [63]. Therefore, Notch is not involved in directing neural progenitors towards one fate or another, this choice being regulated by different signalling pathways (FGF, Wnt and BMP [64]). By contrast, the Notch lateral inhibition process described in the fruit fly very precisely switches the central cell in proneural clusters towards a neural stem cell fate, while all the other cells in the cluster will become epidermal cells. Hence, in *Drosophila*, the first role of Notch is to choose between neural and non-neural fates, which is an earlier role compared to what the pathway does in vertebrates. Conversely, there does not seem to be a general role for Notch in the maintenance of the neural stem cell pools in the fruit fly although Notch plays a role locally in the maintenance of post-embryonic type II neuroblasts [65]. Consequently, the overlap between the insect and vertebrate functions of Notch in central neurogenesis could be seen as limited.

Do data coming from other model animals strategically placed in the metazoan tree support an independent recruitment of Notch in neurogenesis? The general picture is more ambiguous than usually acknowledged. In arthropods, expression data support a role of Notch similar to what is known from *Drosophila* (reviewed in [12]) but strong differences exist in neurogenetic processes between arthropod lineages. In particular, neuroblasts exist only in insects and crustaceans. In ecdysozoans, an ancestral role of Notch in neurogenesis comparable to arthropods is not warranted. In onychophorans [66,67], *Delta* does not show an expression similar to the arthropod situation. In nematodes, the derived components of the Notch machinery do not seem to have a general role in neurogenesis although they are necessary to specify the fate of a number of neural cells [68].

In lophotrochozoans, two annelid species other than *Platynereis* also do not suggest a general role of Notch in neurogenesis: in larvae of the worm *C. teleta* [27], both *Notch* and *Delta* are expressed in two longitudinal rows corresponding to chaetal sacs but show no prominent expression in the ventral neurogenic ectoderm. In the leech *H. robusta*, *Notch* inhibition does not lead to gross defect in the production of neurons *per se* [25,26]. In deuterostomes other than vertebrates, the existing data are insufficient to firmly conclude in favour of an ancestral role of Notch in neurogenesis, but in the sea urchin, a role for Notch in controlling the number of serotoninergic neurons in the larva is proposed while nothing is known on the formation of the adult nervous system [69]. In the chordates amphioxus [70] and ascidians [49,71], the expression of *Delta* homologues and the effects of Notch pathway perturbations are in good agreement with a role in the selection of neural cells.

Animals branching outside of bilaterians, namely cnidarians and sponges, are of special interest to potentially unravel the ancestral Notch role in neurogenesis. Cnidarians do not have a centralized nervous system but possess neural cells. In the evolutionarily derived *Hydra*, a proper *Delta* gene is missing and the small Notch receptor found in this species is involved in head and tentacle patterning [72]. In *Nematostella* (a sea anemone), a proper Notch protein and a short Delta ligand are present. An initial report [21] proposed an involvement of Notch in general neurogenesis. However, another article [22], while confirming a role of Notch in the production of at least a part of the anemone neurons, surprisingly showed that this role was not dependent on SuH and therefore not based on the ‘canonical’ Notch pathway. This point already pushes some authors to propose that Notch may have a more general role in cell differentiation at the metazoan scale and could have been coopted later in both cnidarians and bilaterians in a different manner in the neurogenic pathways [23]. A recent detailed study on *Nematostella* supports the idea that Notch signalling negatively regulates neurogenesis while its exact function remains controversial [24]. Last but importantly, the Notch pathway has been shown to exist in the sponge *Amphimedon* [73] with no less than six *Delta-like* ligands. The Notch pathway thus predates the appearance of a nervous system. Interestingly, some of the *Delta-like* genes are expressed in larval cells interpreted as sensory cells. Some of these cells might be derived from ancestral metazoan sensory cells that were the starting point for the first neurons [74,75].

All these data open the possibility that the roles of Notch in early neurogenesis may have evolved in parallel in several metazoan groups, while we cannot rule out now the possibility that the differing roles of this pathway in different species can also be derived from an ancestral involvement in neurogenesis, secondarily lost in some groups. Nevertheless, the sponge data support the view that the Notch pathway is a crucial ‘toolkit’ that was involved in cell type differentiation very early in metazoan history.

### 3.3. The Notch signalling pathway plays a central role in chaetal sacs morphogenesis in *Platynereis*

Annelid chaetae are extracellular locomotory structures, displayed on the worm appendages, that function in bundles alternatively deployed and retracted in epidermal pockets, the chaetal sacs. We show in this work that the number of chaetae produced in each chaetal sac is specific and strictly regulated. Chaetae are deciduous structures: they are shed progressively and new chaetae grow out of new follicle continuously during the worm's life. This process of replacement has been described as spatially polarized within the chaetal sac [50], chaetae being shed on one side and new follicles forming on the opposite side.

Before the formation and during the invagination of the chaetal sacs (20–36 hpf, figure 5), *Delta* is found in a dynamic ‘salt and pepper’ pattern in superficial or slightly more internal ectodermal cells. These cells may correspond to precursors being selected towards differentiating into chaetoblasts. *Notch* is expressed in broad lateral domains at these stages. Consistent with a role in the selection and strict control of the chaetoblast numbers, drug inhibitions of Notch signalling result in missformed chaetal sacs and the differentiation of abnormal and supernumerary chaetoblasts. We suggest that a process similar to lateral inhibition may be involved in the progressive selection of chaetoblast precursors from the surface ectoderm. We are not able, however, to demonstrate that *Delta*-expressing cells at these early stages are chaetoblast precursors, because of the absence of efficient lineage tracing in *Platynereis* embryos. But both *Delta* and *Hes12* are expressed in the chaetoblasts later and *Hes12* shows a ‘salt and pepper’ expression very similar to *Delta*. In addition, drug treatments do not affect the differentiation of larval and pioneer neurons, suggesting that *Delta* expression is not associated with differentiating neurons in embryogenesis. If *Delta* expressing cells indeed differentiate as chaetoblasts, surrounding cells that are laterally inhibited could give rise either to epidermal or to the various types of follicle cells.

After the complete formation of the chaetal sacs (42–48 hpf), *Delta* and *Notch* are expressed in specific regions of the chaetal sacs. With the help of pattern registration and WGA staining, we obtained a general picture of the gene combinations expressed by the cell populations that compose a chaetal sac (figure 6). *Notch* is found in at least part of the follicle cells, as well as *Hes2*, a potential target transcription factor, and *Nrarp* as shown by *CamL* (a follicle cell marker [39]) expression. *Delta* is found in few cells in the region where chaetoblasts are sitting, as shown by *CS1* (a chaetoblast marker [39]) expression. A sub-population of the cells in the chaetoblast domain also expresses *Pdu-Hes12*.

Drug treatments during later time windows or with a lesser dose of drug (table 1) lead to mild phenotypes. Larvae do not show any clear defects but the absence of protruding bristles. Importantly, the chaetal sacs seem properly formed in late trochophore stage (48 hpf) and a normal number of chaetoblasts, as shown by chitin-synthase and WGA labellings, is found. Additionally, drug treatment is not accompanied by a modification of cell death and cell proliferation patterns in the sacs (electronic supplementary material, figure S2C). DIC microscopy reveals that short abnormal chaetae are present internally (not shown). Gene expressions reveal that *Notch/Hes2*/*Nrarp* and *CamL* expressing cells, corresponding to at least part of the follicle cells, are affected. We propose that follicle cells play an important role in the production of chaetae. Forming the canal through which the bristle is progressively built by basal addition, they have the capacity to secrete materials that could contribute to the bristle structure and rigidity, hence the abortive bristles seen in treated larvae. The *Notch* receptor expressed at the surface of these cells would play a role in maintaining their identity and normal function. What is the significance of the *Delta* and *Hes12* expressing cells in the deep side of the chaetal sac? One speculative idea may be that the cells are new differentiating chaetoblasts that will eventually replace the larval ones, i.e. the start of the ‘treadmill’ process of replacement of chaetae. It is interesting to note in this context that *Nrarp* is expressed in a dorsoventrally polarized way in each chaetal sac and may play a role in the polarized process of chaetae replacement. Parapodia and their chaetal sacs are symmetrically organized respective to a medial longitudinal plane and *Nrarp* is expressed on the externally facing side of each sac respective to this plane (figure 6).

In summary, in late embryogenesis, *Pdu-Notch* and *Pdu-Delta* play a likely role in building chaetal sacs by selecting prospective chaetoblasts, and follicle cells, potentially through a process of lateral inhibition. Later during larval development, when chaetal sacs are in place, Notch probably plays a role in the maintenance and normal function of follicle cells.

### 3.4. Chaetogenesis in lophotrochozoans and the role of the Notch pathway

We can definitively assume that the Notch pathway plays an important role in chaetogenesis in the errantian annelid *Platynereis dumerilii*. In a previous study, Thamm & Seaver [27] proposed, based on expression patterns data, that Notch may be also involved in chaetal development in the sedentarian annelid *Capitella teleta*. As a majority of annelid species are encompassed into the errantia and the sedentaria [76], the requirement of Notch signalling in chaetae formation is probably an ancestral feature in annelids. Chaetogenesis is not restricted to annelids, but also occurs in other lophotrochozoan phyla: brachiopods and molluscs [39,50]. Interestingly, morphological and ultrastructural studies suggest that brachiopod chaetae-like structures are potentially similar to the annelid ones, but they have often been interpreted as non-homologous structures with an independent origin [39]. Recently, phylogenomic studies highlight the fact that brachiopods and annelids are much more closely related phyla than previously thought [77,78]. In this context, gaining insight into the molecular mechanisms underlying chaetogenesis in brachiopods by investigating the formation of the larval chaetae would fill a critical gap. In a comparative approach, several candidate genes that have been shown to be expressed in chaetal sac anlagen in annelids, such as *Hox2, Post1, Nk3, Tlx, GATA456* and of course the Notch pathway core components could be tested [79–81]. The ultimate aim of such an evolutionarily comparative study would be to decipher whether the Notch pathway involvement in the chaetogenesis process is a specific developmental circuit co-opted in the lineage of annelids or whether it was ancestrally present in the common ancestor of both annelids and brachiopods to regulate chaetogenesis.

## 4. Conclusion

The *Platynereis* Notch pathway molecular architecture is well conserved and similar to the urbilaterian ancestral conformation. This pathway was certainly coopted, at least in the lineage of annelids, to play a role in bristle development, more precisely to pattern chaetal sac cells probably through a lateral inhibition process. Its absence of major function in annelid early neurogenesis could be seen as surprising when considering the general idea of a systematic involvement of this pathway to specify neuronal cell fate in bilaterians. We nevertheless highlight the fact that an ancestral link between the Notch pathway and neurogenesis is not as well supported as suggested before, and argue that independent recruitments of Notch in neurogenesis cannot be ruled out. Such a scenario of co-option of genetic networks involved in cell differentiation towards a role in early embryonic patterning has indeed already been advocated with several examples by Erwin & Davidson [82]. Exploring the Notch pathway in other lophotrochozoans and non-bilaterian species will undoubtedly help us to understand whether reasoning on homologies based on the comparison of Notch functions or modes of actions at very large scale is relevant or not.

## 5. Material and methods

### 5.1. Survey of Notch signalling pathway core components in *Platynereis dumerilii*: identification and cloning

*Platynereis* Notch pathway core components were identified by sequence similarity searches against large collections of expressed sequence tags (ESTs) and genomic sequences (*Platynereis* resources, 4dx.embl.de/platy/, D. Arendt, Jékely Lab, jekely-lab.tuebingen.mpg.de/blast/) [28] using the specific conserved domains diagnostic for each gene family in *Drosophila* and/or vertebrate genes. A *Platynereis Notch* gene had already been identified from a BAC sequence ([28], Genbank: AM114766). For *Delta* and *Jagged* family genes, a combination of the specific MNLL and DSL domains found in both type of proteins was used. Complete coding sequences for all genes were assembled from EST fragments using CodonCode Aligner (CodonCode Corporation, USA). Putative exons positions, for the *Platynereis Delta* gene, were mapped on genomic DNA by comparison with ESTs using Artemis [83]. Large gene fragments (1–3 kb) were subsequently cloned by PCR using sequence-specific primers using cDNAs from mixed larval stages as templates (primer sequences and PCR conditions are available upon request). PCR products were TA cloned into the PCR2.1 vector following the manufacturer's instructions (Invitrogen) and sequenced. Partial cDNAs obtained were then used as templates to produce RNA antisense probes for WMISH using Roche reagents. Orthology relationships were defined using as criteria sequence similarities, presence of specific domains and phylogenetic analyses (see below). The newly identified *Platynereis* gene sequences were deposited in Genbank with the following accession numbers: *Pdu-Delta*, KP293866; *Pdu-Jagged*, KP293873; *Pdu-Su(H)*, KP293861; *Pdu-psen*, KP293862; *Pdu-Nrarp*, KP293864; *Pdu-fng*, KP293863; *Pdu-numb*, KP293865; *Pdu-dl-like1*, KP293867; *Pdu-dl-like2*, KP293868; *Pdu-dl-like3*, KP293869; *Pdu-DSL-like1*, KP293870; *Pdu-DSL-like2*, KP293871; *Pdu-DSL-like3*, KP293872.

### 5.2. Survey of Notch signalling pathway core components in a panel of metazoans and phylogenetic analyses

#### 5.2.1. Data sources, sequence retrieving and domains composition

Notch pathway core component searches were carried out using the tblastn or blastp algorithms implemented in ngKlast (Korilog V 4.0, Questembert, France) with *Drosophila*, vertebrate and *Nematostella* proteins as query sequences, with the default Basic Local Alignment Search Tool (BLAST) parameters and a low cut-off *E*-value threshold of 0.1, against 15 genome datasets. Lists of BLAST hits were then reciprocally BLASTed against the human proteins dataset of the NCBI database (reciprocal best hits [69]). Those genomes correspond to 15 species representatives of the main lineages of animals: Porifera, Ctenophora, Cnidaria, Lophotrochozoa, Ecdysozoa and Deuterostomia, with a lophotrochozoan focus (plus one choanoflagellate). For each species, we screened the genome assembly, the predicted protein sequence dataset and transcriptomes when available. The presence of the specific protein domains (available upon request) was systematically checked by scanning sequences with both NCBI Conserved Domain search option V3.10 [84] and InterProscan v. 42 online software [85]. Orthology relationships were defined using as criteria sequence similarities, the presence of specific domains and phylogenetic analyses. The structural diversity of DSL proteins was very broad in metazoans. We had thus to define different classes of proteins to conduct a sensible phylogenetic analysis. We defined the organizations of Delta and Jagged proteins in metazoans (modified from a previous one made by Rassmussen *et al.* [36]) based on the presence of a minimum number and specific arrangement of domains. Based on its likely presence in at least the bilaterian ancestor, we defined as a ‘Delta’ protein a sequence that comprises all of the signal peptide (SP), an MNLL domain, a DSL domain, at least nine EGF repeats, a transmembrane domain (TM) and an intra-cellular domain (ICD). A number of proteins we found that displayed all these domains but with a number of EGF repeats smaller than seven were defined as ‘Delta-like’ proteins. All DSL-containing proteins that comprised at least one additional conserved domain not mentioned above were classified as ‘DSL-like’ proteins and not included in the scope of this phylogenetic analysis. For Jagged proteins, likewise, the minimal domain arrangement is considered to be an SP, an MNLL domain, a DSL domain, at least 16 EGF, a Von Willebrand factor C (VWC) domain, a TM and an ICD. Similarly, Notch proteins that do not possess the minimal domain arrangement of six EGF, three Lin12/Notch repeats (LNR), a TM and three ANK repeats were excluded.

#### 5.2.2. Phylogenetic analyses

The predicted amino acid sequences of the identified *Platynereis* gene fragments were aligned with their presumptive orthologues from 13 metazoan species. These genes were either annotated as such in public databases, or found as predicted genes in whole-genome BLAST screening. *Monosiga brevicolis* sequences were selected as outgroup in order to root the tree, when possible. For Notch and Delta/Jagged proteins, the number of EGF domains is variable, preventing from adequate alignments; they were thus excluded. The Notch alignment includes a partial sequence that begins at the first LNR domain until the end of the protein. The Delta/Jagged alignment includes partial protein sequence from the signal peptide to the end of the DSL domain. Multiple alignments were then performed with MUSCLE 3.7 online [86,87] under default parameters and manually adjusted and improved in Bioedit [88]. Handling of the multiple alignments was also done using Bioedit. Maximum-likelihood analyses were performed using PHYML [89,90] with the Le and Gascuel amino acid substitution model [91] and six rate categories. Statistical support for the different internal branches was assessed by aLRT [92]. Trees were handled using FigTree and provided in the electronic supplementary material, figure S1A. The whole list of sequences used in this study and the multiple alignments are available in the electronic supplementary material, file S1.

### 5.3. Animal culture and collection

*Platynereis* embryos were obtained from an 18°C breeding culture established in the Institut Jacques Monod (Paris), according to the protocol of Dorresteijn *et al*. [93]. Staging of the embryos was done following Fischer *et al*. [31]. Embryos and larvae were fixed in 4% PFA, 1× phosphate-buffered saline (PBS), 0.1% Tween 20 and stored at −20°C in methanol 100% [94].

### 5.4. WMISH, immunohistochemistry, chitin and membrane labelling

NBT/BCIP whole-mount *in situ* hybridizations were performed as previously described for wild-type and treated embryos [34] with a modified protocol allowing for sharper signal as required for three-dimensional confocal imaging [52] (performed on two larval stages (48 and 72 hpf)). Cilia and neurite staining on 72 hpf treated larvae (see above) was done as previously described [34] using the mouse anti-acetylated tubulin antibody (Sigma T7451, 1 : 500), fluorescent secondary anti-mouse IgG Alexa Fluor 488 or 555 conjugate (Invitrogen, 1 : 500) and DAPI (1 : 1000). Neuronal antibodies were also used to label specific neuron populations as well as their axons and dendrites as previously described: antibodies against the neuropeptide RFamide [32], the monoamine transmitter serotonin [32] and the amidated neuropeptides RYamide, and FLamide [41]. The chitinous chaetae were stained with WGA FITC conjugate (Sigma L4895) at a concentration of 100 µg ml^−1^ for 12 h at 4°C. The staining was stopped with several washes in PBS/0.1% Tween 20. Larvae for three-dimensional imaging were counterstained with DAPI (Sigma), diluted at 1 µg ml^−1^ in PBS/0.1% Tween 20. Following WMISH or antibody labelling, larvae were transferred into 97% TDE diluted with PBS/0.1% Tween 20 [52]. Three-dimensional stacks of the nuclear DAPI labelling and of the reflected signal of NBT/BCIP labelling [95] were recorded and used for three-dimensional reconstructions and pattern registration. The FM4-64 dye (ThermoFisher Scientific T3166), as a membrane stain, was used to detect cellular outlines. 15 hpf embryos (before ciliae appearance) were embedded in 1% UltraPure™ Low Melting Point agarose containing FM4-64 (2 µl ml^−1^), in a glass bottom Petri dish covered by seawater with FM4-64 (2 µl ml^−1^) before imaging.

### 5.5. Small molecule inhibition

Embryos were incubated in the chosen time window (see main text) in natural seawater containing anti-presenilin drugs: DAPT (Tocris Bioscience), LY-411575 (A. G. Scientific) or RO-4929097 (Stemgent). For all inhibitors, stock solutions were 10 mM in 100% DMSO and different final concentrations were used (consequently the DMSO concentrations too). Control embryos originating from the same batch were incubated in natural seawater containing 0.5% DMSO. After drug incubations, embryos were either (i) rinsed in seawater for 24 h (and so, until 72 hpf) to assess their phenotypes or (ii) fixed at 48 hpf to perform WMISH with several markers. Approximately 150 embryos per treatment were examined and counted.

### 5.6. Whole-body gene expression registration

Image registration of three-dimensional gene pattern scans based on the DAPI nuclear labelling were performed using the open-source software package ITK4.0 and a collection of specific scripts developed in the Jékely Lab [52]. All patterns were registered to average anatomical reference templates (48 and 72 hpf). Both of these reference templates were calculated using a total of 40 DAPI-labelled larva three-dimensional stacks. The complete protocol for unbiased anatomical reference calculation and the reference files themselves are available upon request. Average patterns for a given gene were calculated using at least five individual larvae and gene pattern comparisons were performed using ImageJ and Imaris.

### 5.7. Edu cell proliferation and TUNEL assay

Incorporation in EdU (200 µM) was done directly in seawater, for 30 min prior to tissue fixation. Labelling was done after rehydration, using the Click-iT^®^ 488/555 EdU kit, following the manufacturer's instructions (Molecular Probes). Embryos were counterstained with Hoechst or DAPI (1 µg ml^−1^) for nuclear staining. For TUNEL assays, after embryo rehydration, cuticle digestion and post-fixation, the terminal deoxynucleotide transferase reaction was performed following the protocol of the Click-iT TUNEL kit (Molecular Probes), with previously described minor modifications [34]. Embryos used for positive control were incubated with DNAse I to generate DNA breaks (similar incubation, 1 h on ice followed by 1 h at 37°C). Labelling was then performed following the protocol of the Click-iT TUNEL kit (Molecular Probes). For cell counting of EdU experiments, confocal stacks were obtained for five control embryos and five embryos treated with each of the three different drugs. To enumerate precisely all cells in a given larval region of interest (ROI), all embryos were registered with ITK4.0 as described above to the 48 hpf anatomic reference template. ROIs corresponding to the neurectoderm, episphere, chaetal sacs and stomodeum were delimitated using the 48 hpf template and the three-dimensional surface tool of Imaris software (Bitplane). DAPI- and EdU-positive nuclei were then counted for each registered sample stack and each ROI using Imaris.

### 5.8. Imaging

Bright field images were taken on a Leica microscope. Classical confocal images were taken with a Leica SP5 confocal microscope. Adjustments of brightness, contrast and Z projections were performed using the ImageJ software. Confocal images for registration procedure were taken in a Zeiss LSM 710 confocal microscope. Three-dimensional surface of expression patterns and associated videos were performed using, respectively, the surface and animation tools of Imaris. Co-localization stacks of expression patterns were obtained with the Coloc tool of Imaris and co-localization images were then edited on ImageJ. Live imaging of the 24 hpf FM4-64 labelled embryos was performed on a Zeiss LSM 780 confocal microscope with a 63× water immersion objective. Z-stacks (*z* = 0.53 µM) of several whole embryos were performed every hour (using the multiposition option). The figure panels were compiled using Adobe Illustrator and Adobe Photoshop software.

### 5.9. Statistical analyses

To compare the number of embryos harbouring a normal versus abnormal chaetae arrangement (Y variable) in control and treated conditions (X variable), a Fisher's test (two sided) was performed to test the independency of X and Y. To compare the number of positive cells for several markers (*CS*, Crya, Fmrfa, serotonin and EdU) in control and treated embryos, a Welch's 2 sample *t*-test (two sided) was performed for each comparison, to test whether the means are equivalent between the two groups. Statistical tests were performed using the BioStaTGV online platform (http://marne.u707.jussieu.fr/biostatgv/?module=tests). All raw data are provided in the electronic supplementary material, table S1.

## Supplementary Material

Electronic supplementary material full legends - The Notch pathway in the annelid Platynereis: Insights into chaetogenesis and neurogenesis processes”; Eve Gazave, Quentin I. B. Lemaître and Guillaume Balavoine

## Supplementary Material

Figure S1: The Notch pathway in the annelid Platynereis: Insights into chaetogenesis and neurogenesis processes”; Figure S1: Eve Gazave, Quentin I. B. Lemaître and Guillaume Balavoine

## Supplementary Material

Figure S2: The Notch pathway in the annelid Platynereis: Insights into chaetogenesis and neurogenesis processes”; Figure S1: Eve Gazave, Quentin I. B. Lemaître and Guillaume Balavoine

## Supplementary Material

Figure S3: The Notch pathway in the annelid Platynereis: Insights into chaetogenesis and neurogenesis processes”; Figure S1: Eve Gazave, Quentin I. B. Lemaître and Guillaume Balavoine

## Supplementary Material

Figure S4: The Notch pathway in the annelid Platynereis: Insights into chaetogenesis and neurogenesis processes”; Figure S1: Eve Gazave, Quentin I. B. Lemaître and Guillaume Balavoine

## Supplementary Material

File S1 all sequences + ali_new

## Supplementary Material

Table S1

## Data accessibility

The data from this study are available as the electronic supplementary material.
