# Supplementary material for: The Notch pathway in the annelid Platynereis: insights into chaetogenesis and neurogenesis processes
Source: Open Biol. 2017 Feb 1;7(2):160242. doi: 10.1098/rsob.160242 (PMC5356439; doi:10.1098/rsob.160242)
Supplement: Electronic supplementary material full legends - The Notch pathway in the annelid Platynereis: Insights into chaetogenesis and neurogenesis processes”; Eve Gazave, Quentin I. B. Lemaître and Guillaume Balavoine [file rsob160242supp1.pdf]

Electronic supplementary material legends from "The Notch pathway in the annelid *Platynereis*: Insights into chaetogenesis and neurogenesis processes"; Eve Gazave, Quentin I. B. Lemaître and Guillaume Balavoine; Open biology http://dx.doi.org/10.1098/rsob.160242

Figure S1: **Phylogenetic analysis of the *Platynereis* Notch pathway core components, *Pdu-Delta* splice variants structures, alignment of bilaterian *Delta* genes and scenario for the origin and evolution of Delta and Jagged proteins in metazoans.**

A) Maximum Likelihood (ML) trees constructed with PhyML are shown. Statistical supports (aLRT) are indicated on the nodes by color circles (color code is indicated in the figure). Nodes without color circles are not statistically supported. Whenever possible, the choanoflagellates (or non-bilaterian species) sequences were used to root the phylogenetic trees. Sequences were identified either by their NCBI accession number (when available) or another database accession number. Color code for the species name are as follow: red, *Platynereis dumerilii*; green, deuterostomes (2 different species); orange, ecdysozoans (2 different species); purple:, lophotrochozoans (others than *Platynereis*: 5 different species); dark blue, cnidarians (1 species); light blue: sponge and ctenophore (2 species); black, choanoflagellates (1 species). (a) *Delta* + *Jagged* genes. *A. queenslandica* (sponge) sequences were used as outgroup to root the phylogenetic tree. Robust clades of *Delta* and *Jagged* genes are highlighted with boxes. (b) *Delta* + *Jagged* + *Pdu-Delta-like* genes. *A. queenslandica* (sponge) sequences were used as outgroup to root the phylogenetic tree. Robust clades of *Delta* and *Jagged* genes are highlighted with boxes. The *Pdu-Delta-like* genes sequences cluster together in a clade distinct from either *Delta* or *Jagged* groups. (c) *Notch* genes. *A. queenslandica* (sponge) sequence was used as outgroup to root the phylogenetic tree. *Pdu-Notch* is grouped within most of the lophotrochozoan Notch sequences. (d) *Presenilin* genes. *M. brevicolis* (choanoflagellate) sequence was used as outgroup to root the phylogenetic tree.

Pdu-psn is grouped within most of the lophotrochozoan presenilin sequences. (e) *Su(H)* genes. *M. brevicolis* (choanoflagellate) sequence was used as outgroup to root the phylogenetic tree. *Pdu-Su(H)* is grouped within most of the lophotrochozoan Su(H) sequences. (f) *Nrarp* genes. *N. vectensis* (cnidarian) sequence was used as outgroup to root the phylogenetic tree. (G) *Numb* genes. *M. leidyi* (ctenophore) sequence was used as outgroup to root the phylogenetic tree. *Pdu-numb* is grouped within most of the lophotrochozoan numb sequences. (H) *Fringe* genes. *A. queenslandica* (sponge) sequences were used as outgroup to root the phylogenetic tree.

B) The different domains are schematized by colored boxes (see inset). The intron positions, indicated by gaps, are essentially conserved between *Pdu-Delta* splice variants except for the last one, located after the TM domain, absent in the *Pdu-Delta*^{tv2}. Due to the retention of this intron, *Pdu-Delta*^{tv2} lacks the ATEV domain, present in *Pdu-Delta*^{tv1}.

C) *Pdu-Delta* gene follows the MNLL-DSL-(9)xEGF-TM-ATEV domain composition suggested to be ancestral in bilaterian [1]. In all bilaterian sequences, the EGF domains harbor a conserved pattern of repeat spacings and cysteine residue spacings. Cysteines are highlighted in red. SP: signal peptide; TM: Transmembrane Domain.

D) The different domains are schematized by colored boxes (see inset). An ancestral Delta (DSL) like protein was present in *Urmetazoa*, with an unknown number of EGF domains. Gene duplication and domains acquisitions occurred during the transition from the metazoan to the eumetazoan ancestor leading to the appearance of Jagged protein [2]. ATEV domain acquisition took place during the transition from eumetazoan to bilaterian ancestor, where two Delta splice variants were already present, with 9 EGF domains. The Jagged proteins probably harbored 16 to 18 EGF domains in *Urbilateria*. In deuterostomes, lophotrochozoans and ecdysozoans, lineage-specific gene duplications and domain losses occurred, giving rise to the present pattern of Jagged, Delta and Delta-like observed

Figure S2: ***Pdu-Notch*, *Pdu-Delta* and *Pdu-Jagged* brain cells identities and expression in mesodermal posterior stem cells; Pharmacological treatments altering Notch pathway activity induce neither cell death nor cell proliferation in *Platynereis* embryos.** A) All images are confocal maximum z-projection (40 µm) of averaged expression patterns registration. Apical views of the brain, dorsal side up are shown. White pixels reveal the colocalization patterns. External white dotted circle delineates the shape of the brain. Internal white dotted circle delineates the apical organ. a - o, 72 hpf; p - y, 48 hpf. (a and u) Acetylated tubulin staining showing the larval axonal scaffold with the main nerves at 72 and 48 hpf respectively. *Pdu-Notch*, *Pdu-Delta* and *Pdu-Jagged* brain expression pattern registration are shown in panel b, c, d and p respectively. A set of neuropeptide antibodies (RYamide (g, h, s and x), FMRFamide (j, k, r and w), FLamide (l and m), Serotoninergic (q and v)), as well as specific neuron populations markers (*Pdu-VAchT*: cholinergic (n and o), *Pdu-Chx10*: interneurons (t and y)) were used to determine the identity of *Pdu-Notch*, *Pdu-Delta* and *Pdu-Jagged* + brain cells. Purple arrow indicates colocalization patterns inside the apical organ. Orange arrow indicates colocalization in brain cells outside the apical organ.

B) All images are confocal maximum z-projection of averaged expression patterns registration. Ventral surface views of the whole larvae, anterior side up, are shown. White patterns reveal the colocalization of green and red pixels. (a) Schematic representation of a 72 hpf larvae, the mesoteloblasts are highlighted in yellow. *Pdu-Notch* and *Pdu-Delta* expression pattern registration are shown in panel b and c respectively. *Pdu-Smb* (d) and *Pdu-Vasa* (e) are markers of the posterior stem cells of the 72 hpf larvae. *Pdu-Notch* and *Pdu-Delta* are co-localized in the mesoteloblasts (F, yellow arrow). They are also both co-expressed with *Pdu-Smb* (g and i) and *Pdu-Vasa* (h and j) in *Platynereis* posterior stem cells.

C) Ventral views of trochophore larvae (48 hpf) are shown (anterior is up). (a) A positive control embryo incubated in DNAse I shows a labelling of all cells following TUNEL assay. (a-a_4) The amount of apoptotic cells observed in treated embryos was equivalent to that observed in their control counterpart, suggesting that none of the treatments induced cell death in *Platynereis* embryos. The scheme in b represents a superficial ventral view of a 48 hpf larvae, with the neuro-ectoderm (Ne) in grey and the midline represented by a white dashed line. The scheme in c shows a deeper ventral view of a 48 hpf larvae, with the 6 ventral chaetal sacs. Embryos were incubated with DAPT (40 µM, b_2 and c_2), or LY-411575 (1 µM, b_3 and c_3), or RO-492997 (2 µM, b_4 to c_4) in DMSO or in DMSO only (control group, b_1 and c_1) from 24 to 48 hpf. Thirty minutes EdU (red) incorporation and Hoechst nuclear staining (blue) at 48 hpf show a similar cell proliferation profile in both control (b_1 and c_1) and treated embryos (b_2 to c_4), in superficial and deeper ventral views. (D) Bars below each image show the mean percentage of EdU+ cells (red) for a whole embryo and for specific structures (neuro-ectoderm, episphere, chaetal sacs and stomodeum) for both control and treated embryos.

Figure S3: **Chaetogenesis detailed studies.**

A) Schematic representation of a single chaetal follicle invagination hypothesis and follicle final architecture in *Platynereis*. a: Earliest stage with a “rosette” of cells (composed of a chaetoblast surrounded by several follicle cells) in the ectodermal surface of the embryo (apical view). b: Beginning of invagination of both chaetoblast and follicle cells (transversal view). c: Mature single chaetal follicle that gives rise to one developing chaetae. One follicle is composed of a basally located cell: the chaetoblast. On top of it, several follicle cells are present (4 in total) and envelop the growing chaeta. Red: chaetoblast; white: follicle cells; blue: ectodermal cells; black: chaetae. Modified and adapted from [3, 4].

B) Detailed chaetogenesis dynamic and chaetae counting during *Platynereis* embryonic development**.** a to e: Graphics show the number of chaetae per sac for each of the 12 sacs (6 ventral and 6 dorsal) for several embryos (n=5 to 11) at 33, 36, 39, 42 and 48 hpf respectively. a: At 33 hpf, only the chaetal sacs of 1st and 2nd segments present chaetae. The inset shows the nomenclature used for identifying each sac (dorsal/ventral side, left/right side, 1st/2nd/3rd pair). The sacs of the 3rd segment start to produce chaetae at 39 hpf (c). A constant and stable number of chaetae per sac is reached at 42 hpf (d), with the sacs of the 2nd segment (either ventral or dorsal) always containing more chaetae (D-L2>D-L1, for example). In addition, the dorsal chaetal sacs have more chaetae than the ventral ones (D-L1>V-L1, for example) (f). There is no left/right asymmetry (D-L1=D-R1, for example) (f).

C) FM4-64 membrane dye reveals "rosettes" of cells invaginating throughout *Platynereis* development. a to d': Confocal snapshots of live imaged *Platynereis* embryos stained with FM4-64 dye at 27, 31, 33 and 36 hpf. Membranes are in white. All views are laterals, ventral side is left, and anterior is up. Each developmental stage snapshot is deeper than the previous one in order to follow the invaginated cells. a and a': 27 hpf, a rosette of ectodermal cells is observed. b, b', c and c': 31 and 33 hpf, later in development the six chaetal sacs outlines start to be visible, 2 per segments. At 36 hpf, the chaetal sacs or fully formed. White dotted lines indicate the segment boundaries. a'' to d'': corresponding representation of the chaetal sacs.

D) Image registration of 3D surface gene expression patterns in chaetal sacs. The schemes in a, b and c show respectively a ventral, lateral left and dorsal slightly posterior view of a 48 hpf trochophore larva. Chaetal sacs are represented by blue dashed circles/lines. The black or white circles (a to c) correspond to the location of the stomodeum in each view. The WGA staining shows the location and numbers (7 to11) of chaetoblasts sitting at the internal tip of the sac (a_1 to c_1). The *Pdu-Twist* expression spans mesodermal territories surrounding the chaetal sacs and the stomodeum, as especially visible in the lateral view (a_2 to c_2). Broad *Pdu-*

Notch and restricted *Pdu-Delta* expressions in the chaetal sacs and the stomodeum are shown (a_3, to c_4). Combination of two 3D surface labellings is shown in the merged panels (d to d_4). The dorsal posterior views show the whole set of 12 sacs and highlight the fact that dorsal sacs genes expressions are slightly less pronounced (c_3, c_4). The details of each combination of labelling and views are mentioned in each merged panels. *Pdu-Notch* 3D surface pattern corresponds to the gaps in the *Pdu-Twist* expression. *Pdu-Delta* expression is proximal in the sac in comparison to *Pdu-Notch* distal expression (d_3 and d_4).

E) Notch pathway inhibition abolishes follicle cells markers expression in *Platynereis* embryos. Ventral views of whole trochophore larvae (48 hpf) are shown (anterior is up). Embryos were incubated with DAPT (40 µM, a_1 to f_1), or LY-411575 (1 µM, a_2 to f_2), or RO-492997 (2 µM, a_3 to f_3) in DMSO or in DMSO only (control group, a to f) from 24 to 48 hpf. WMISH reveal drastic losses of follicle cells markers (*Pdu-Notch, Pdu-Nrarp, Pdu-Hes2* and *Pdu-CamL)* expression in treated embryos, while chaetoblast markers (*Pdu-CS1, Pdu-Delta* and *Pdu-Hes12*) are not affected by any drug treatment.

Figure S4: **Additional information on neurogenesis.**

A) *Elav* expression patterns in both treated and control larvae reveal no involvement of the Notch pathway in the selection of the neural progenitors that will give rise to the 9 pioneer neurons of the 24 hpf larvae. Larvae were incubated with LY-411575 (1 µM, a1) and RO-492909 (30 µM, a2) in DMSO or in DMSO only (control group, a) from 12 and 16 hpf to 24 hpf and fixed at 24 hpf. Ventral views of 24 hpf larvae treated from 12 to 24 hpf are shown (anterior is up). At 24 hpf, the postmitotic neuron marker *Elav* is expressed in the 9 pioneer neurons of the larvae (black arrowheads) and in several cells of the episphere (bleu arrows). In treated embryos, the numbers of *Elav*+ cells is strictly the same: 9 per embryos, 10 embryos per condition. B) *Pdu-SoxB, Pdu-neurogenin (Ngn)* and *Pdu-Pax6* (neurogenic

ectoderm marker) images registrations: no colocalization with Notch ligand and receptor. *Pdu-SoxB, Pdu-neurogenin (Ngn)* and *Pdu-Pax6* at 48 hpf show an expression in the VNC domain and in the brain (a to c, ventral views, anterior up). None of them are co-expressed with both *Pdu-Notch* and *Pdu-Delta* internal expression (d to I, ventral views, anterior up). C) Averaged expression patterns of neurogenesis markers at 72 hpf in both treated and control larvae. Ventral views of whole nectochaete larvae (72 hpf) are shown (anterior is up). Larvae were incubated with LY-411575 (1 µM, a_1 to c_1), in DMSO or in DMSO only (control group, a to c) from 30 to 48 hpf and fixed at 72 hpf. Averaged expression of at least 8 embryos for each gene (*Pdu-Collier, Pdu-Vacht* and *Pdu-Chx10*) was used for neurons counting. Yellow asterisks: artificial staining of chaetae.

File S1: The whole list of sequences and multiple alignments used in this study is provided.

Table S1: **Raw data used for statistical analyses.** All data used for figures (3, 7, 9) and supplementary materials (2C, 3B, 4A) as well as result of statistical tests are supplied.

1 Rasmussen, S. L., Holland, L. Z., Schubert, M., Beaster-Jones, L., Holland, N. D. 2007 Amphioxus *AmphiDelta*: evolution of Delta protein structure, segmentation, and neurogenesis. *Genesis*. **45**, 113-122. (10.1002/dvg.20278)

2 Gazave, E., Lapébie, P., Richards, G. S., Brunet, F., Ereskovsky, A. V., Degnan, B. M., Borchiellini, C., Vervoort, M., Renard, E. 2009 Origin and evolution of the Notch signalling pathway: an overview from eukaryotic genomes. *BMC evolutionary biology*. **9**, 249. (10.1186/1471-2148-9-249)

3 Zakrzewski, A. C. 2011 Molecular Characterization of Chaetae Formation in Annelida and Other Lophotrochozoa: Berlin.

4 Tilic, E., Hausen, H., Bartolomaeus, T. 2014 Chaetal arrangement and chaetogenesis of hooded hooks in *Lumbrineris (Scoletoma) fragilis* and *Lumbrineris tetraura* (Eunicida, Annelida). *Invertebrate Biology*. **133**, 354-370. (10.1111/ivb.12066)
