# Supplementary figures and images for: The Notch pathway in the annelid Platynereis: insights into chaetogenesis and neurogenesis processes
Source: Open Biol. 2017 Feb 1;7(2):160242. doi: 10.1098/rsob.160242 (PMC5356439; doi:10.1098/rsob.160242)

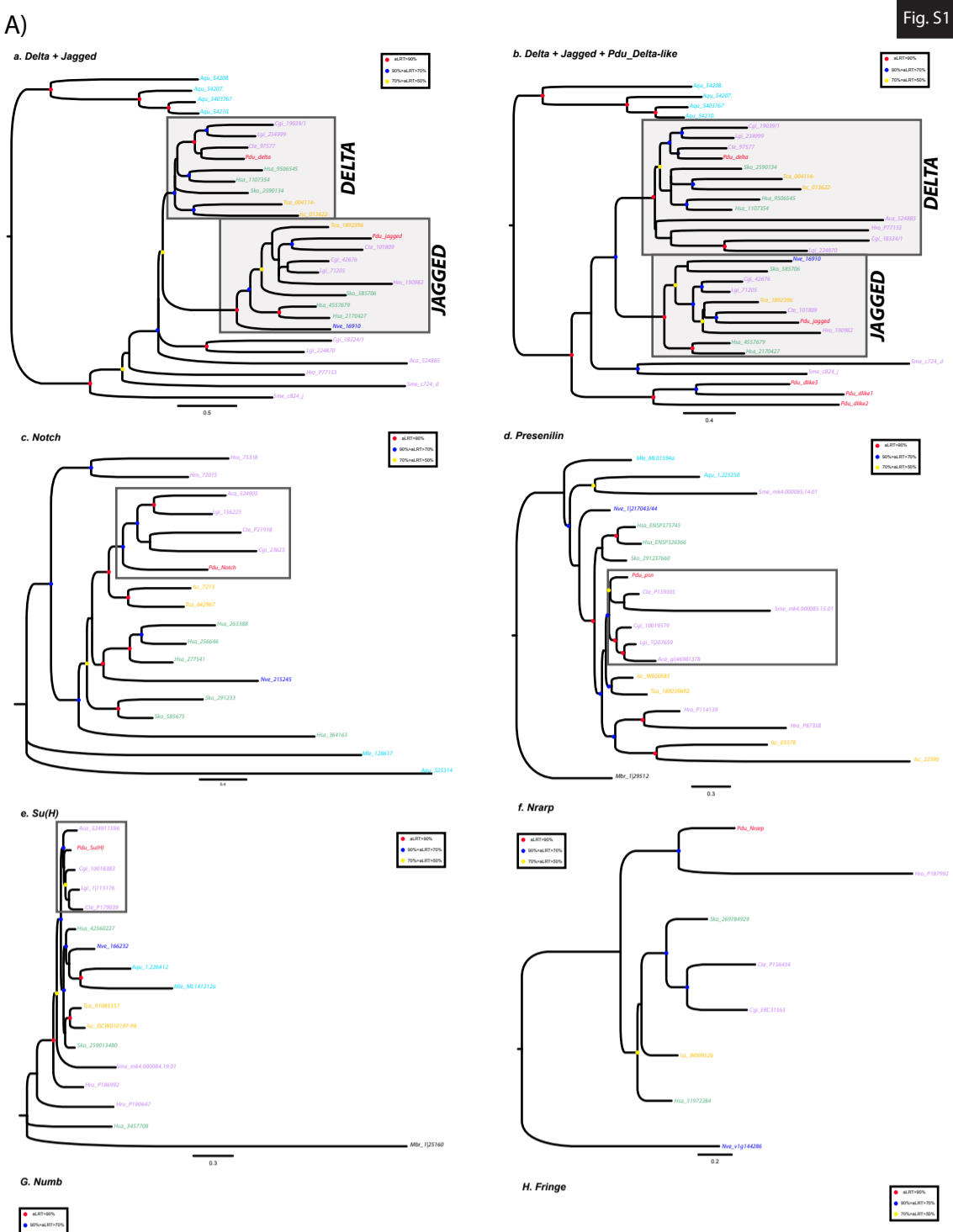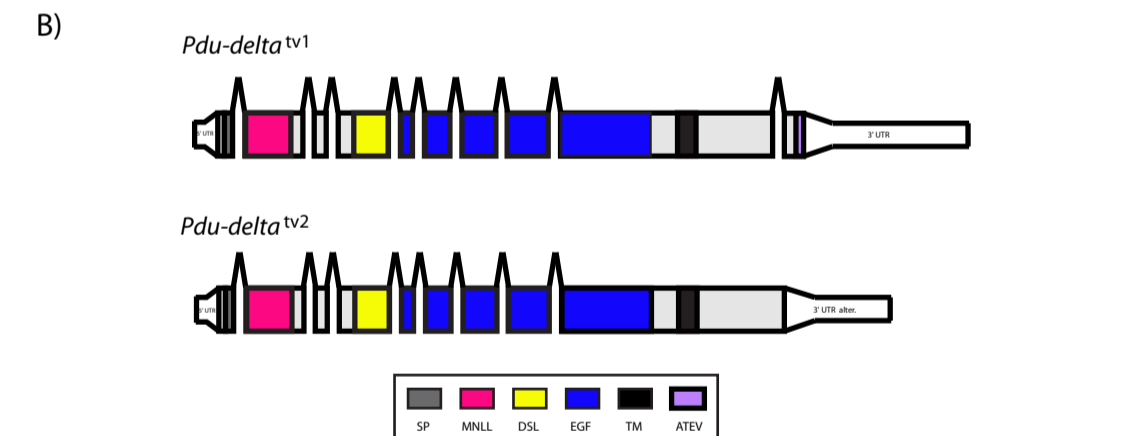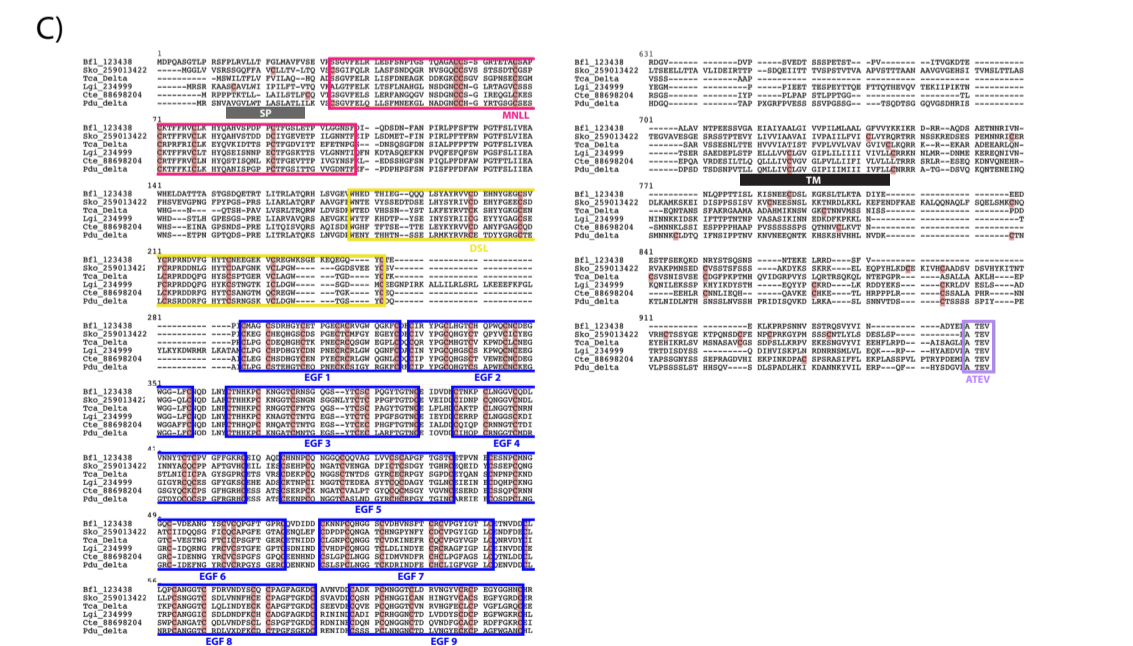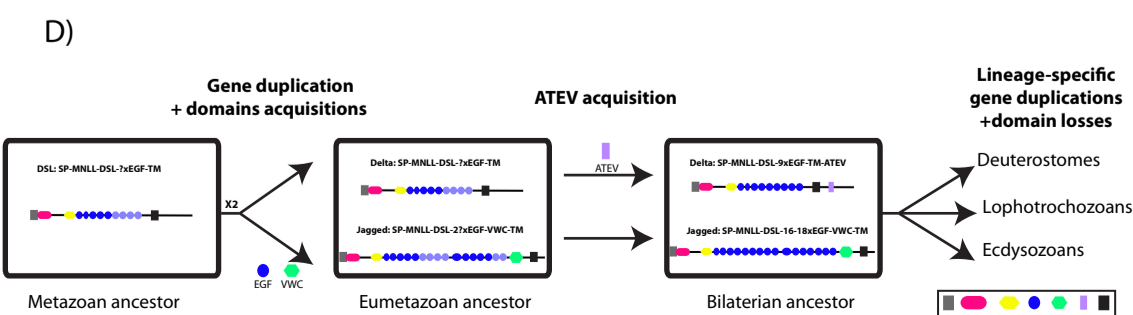

Supplement: Figure S1: The Notch pathway in the annelid Platynereis: Insights into chaetogenesis and neurogenesis processes”; Figure S1: Eve Gazave, Quentin I. B. Lemaître and Guillaume Balavoine [file rsob160242supp2.pdf]

A)

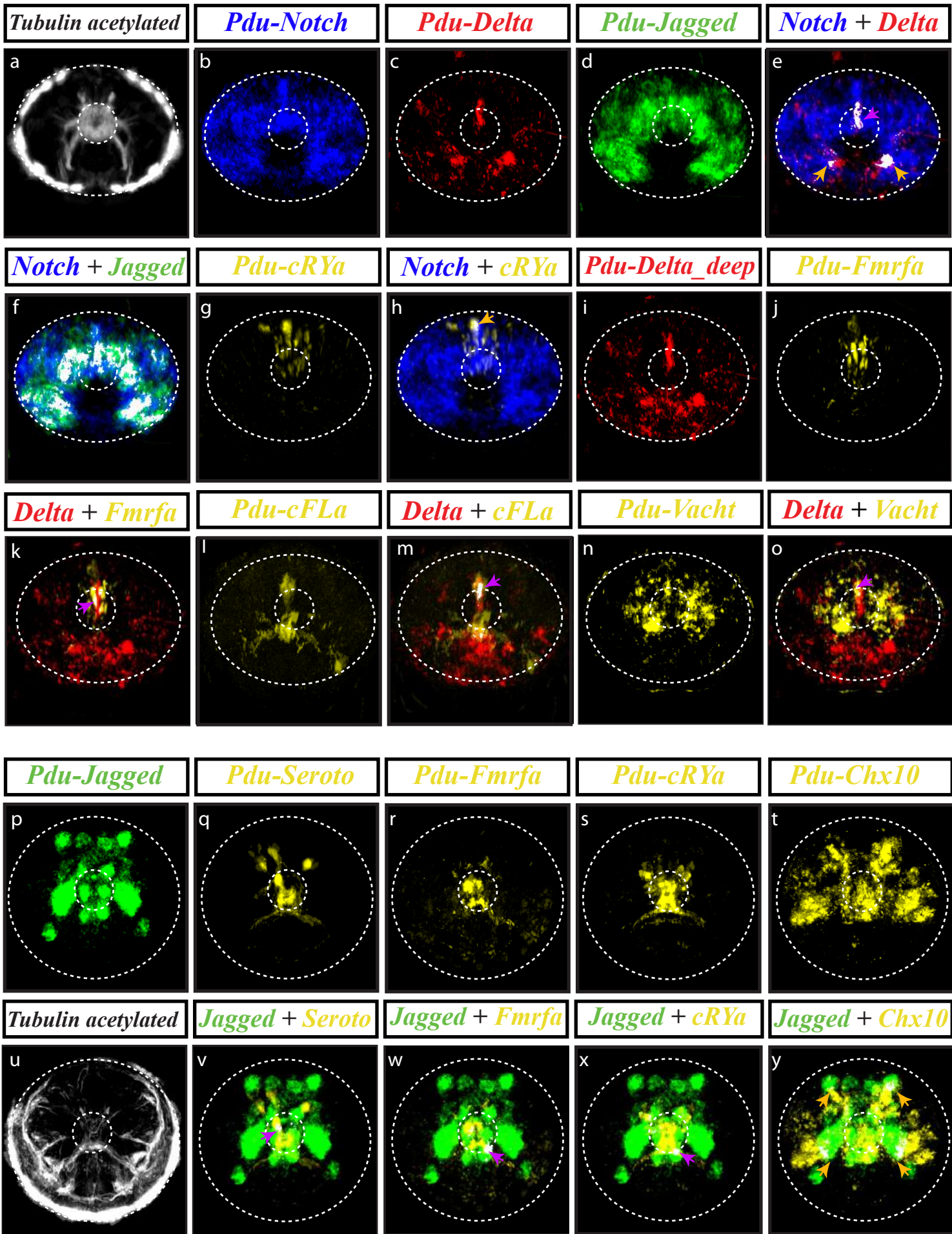

B)

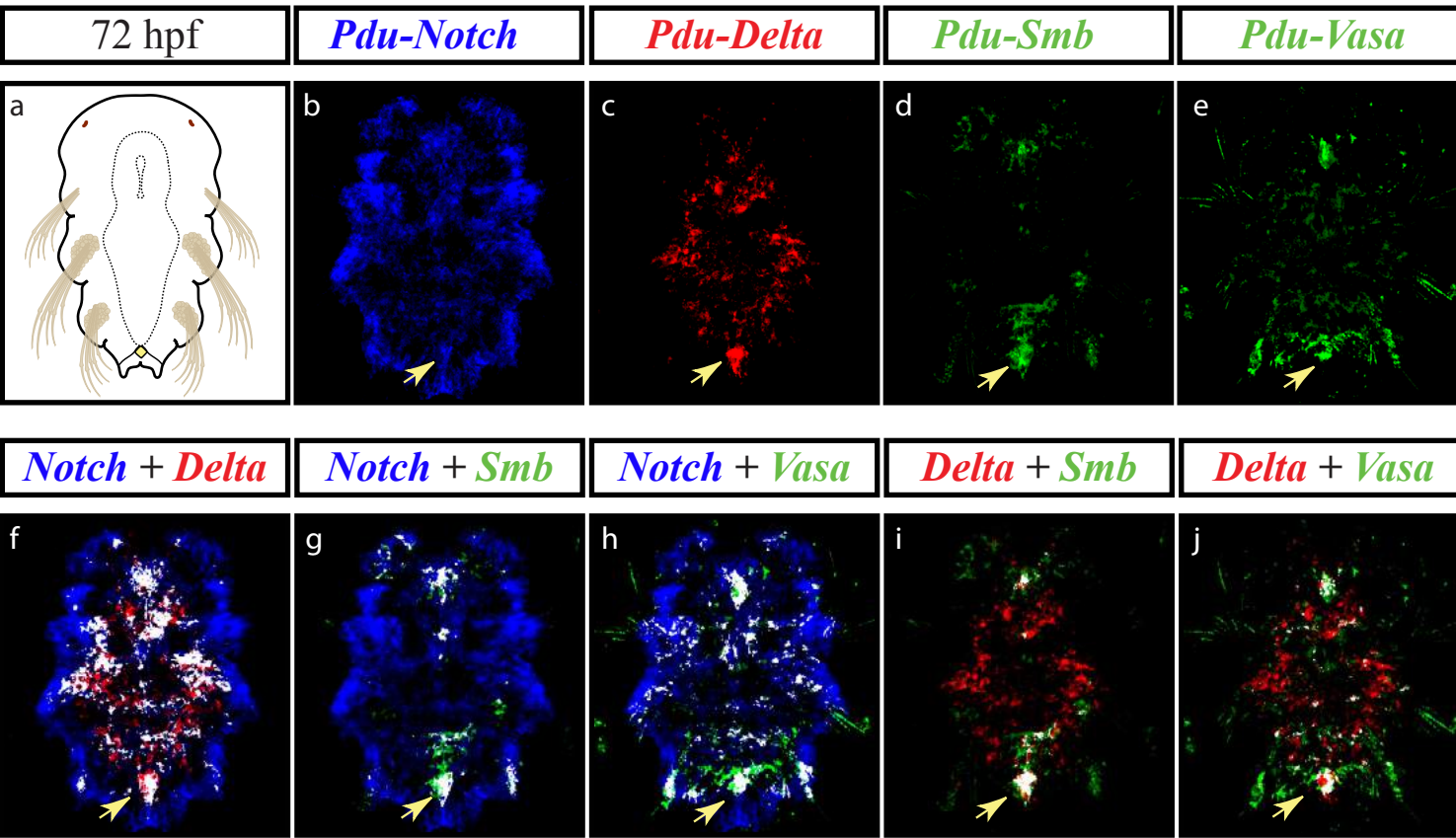

C)

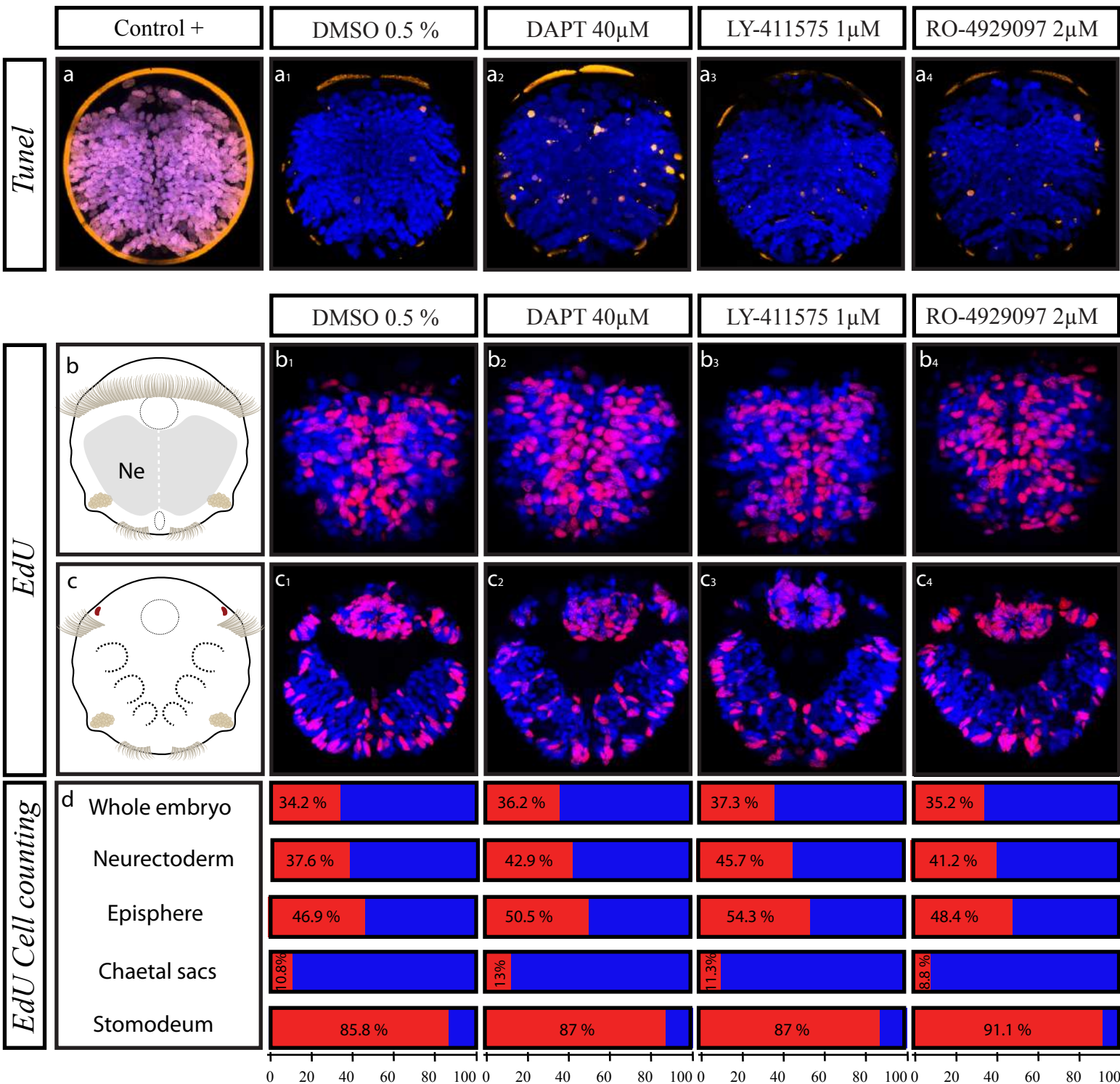

Supplement: Figure S2: The Notch pathway in the annelid Platynereis: Insights into chaetogenesis and neurogenesis processes”; Figure S1: Eve Gazave, Quentin I. B. Lemaître and Guillaume Balavoine [file rsob160242supp3.pdf]

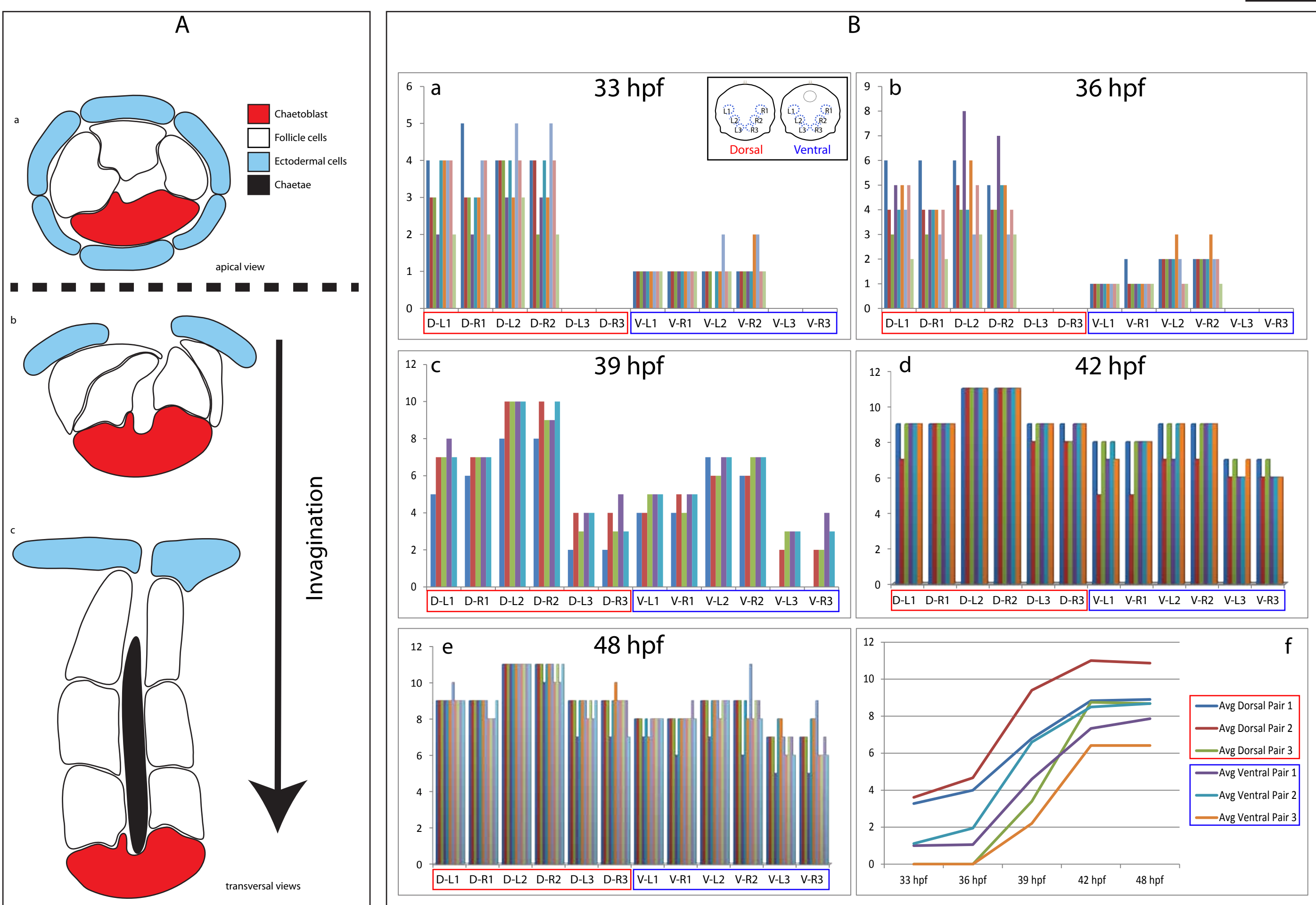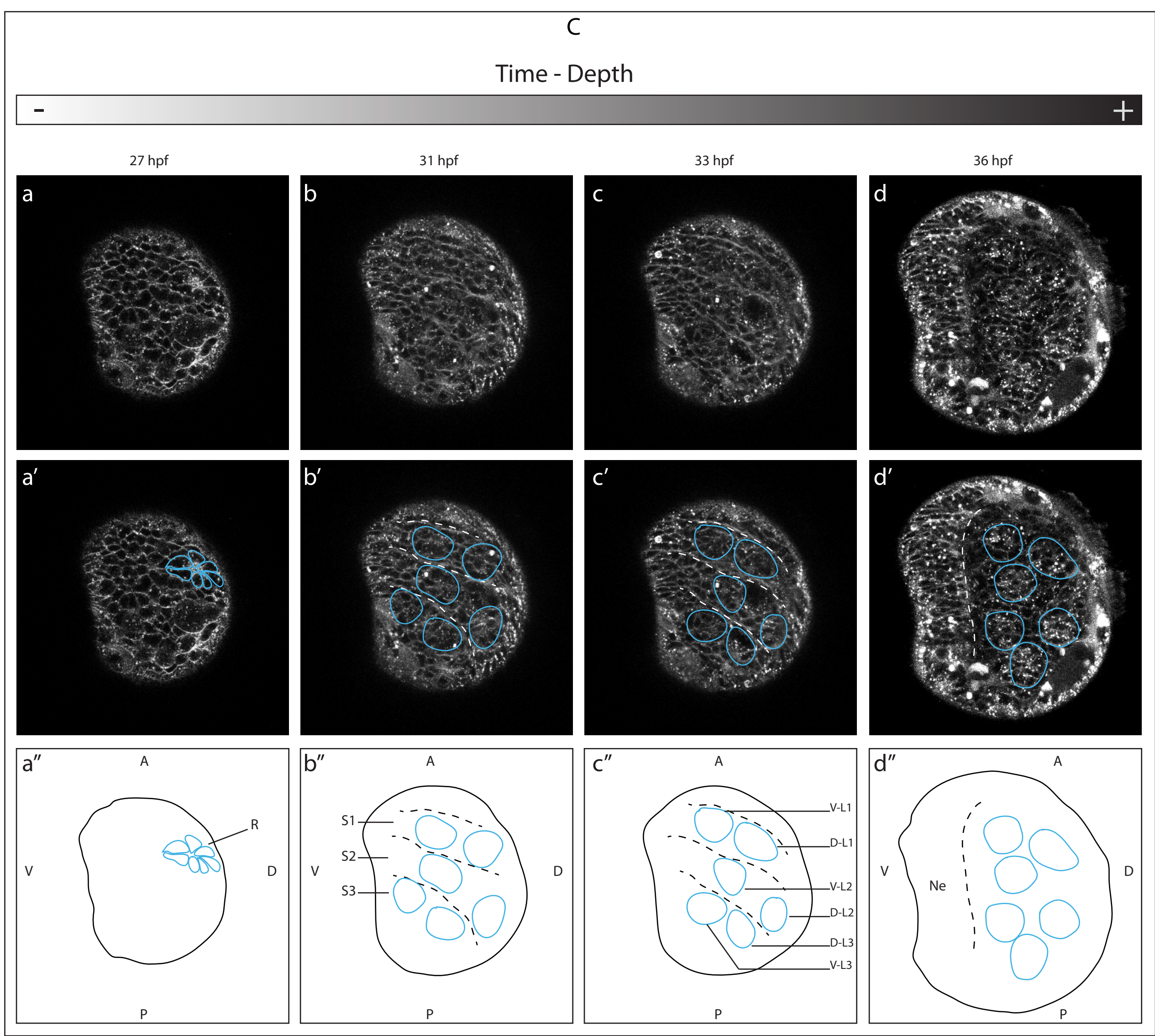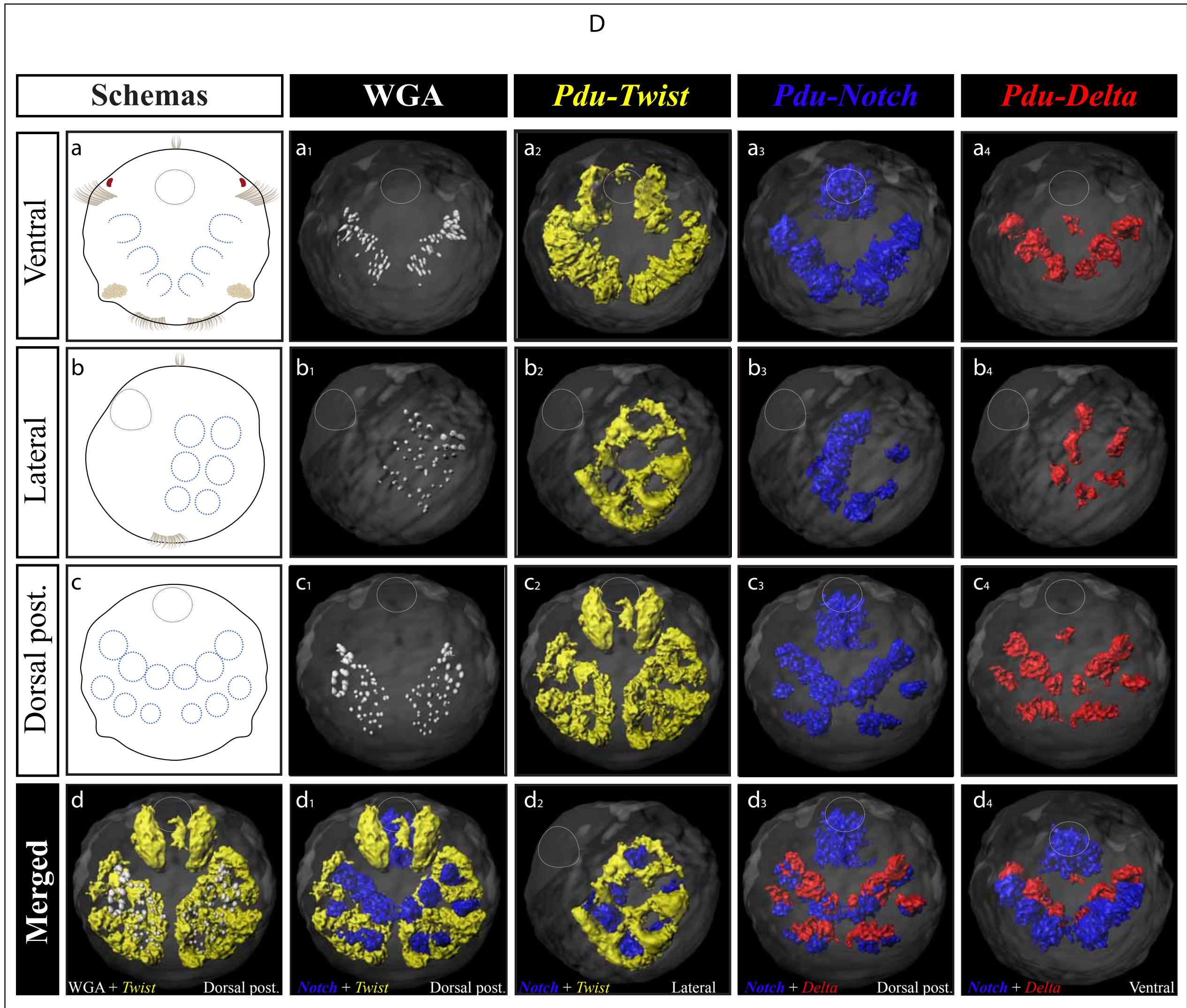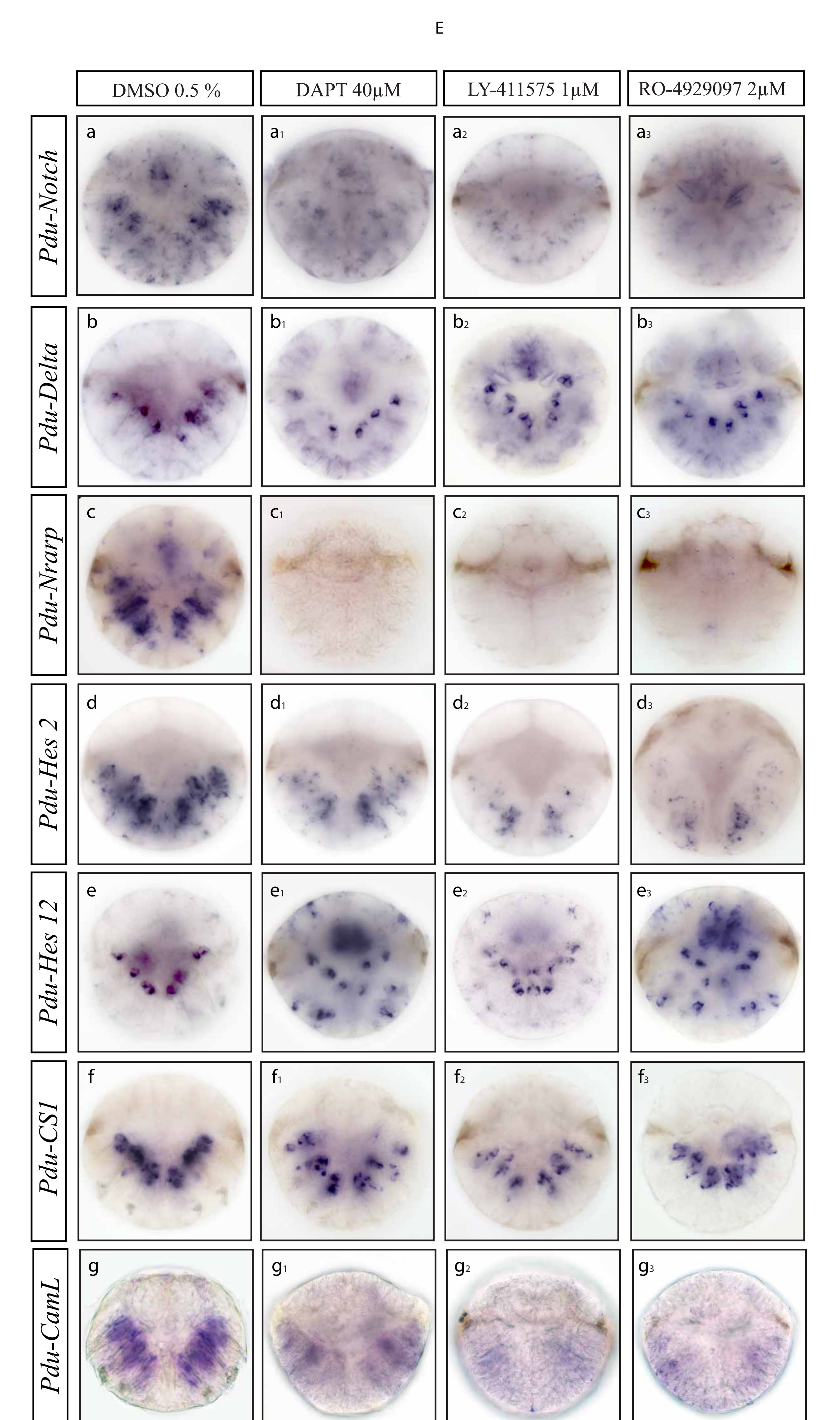

Supplement: Figure S3: The Notch pathway in the annelid Platynereis: Insights into chaetogenesis and neurogenesis processes”; Figure S1: Eve Gazave, Quentin I. B. Lemaître and Guillaume Balavoine [file rsob160242supp4.pdf]

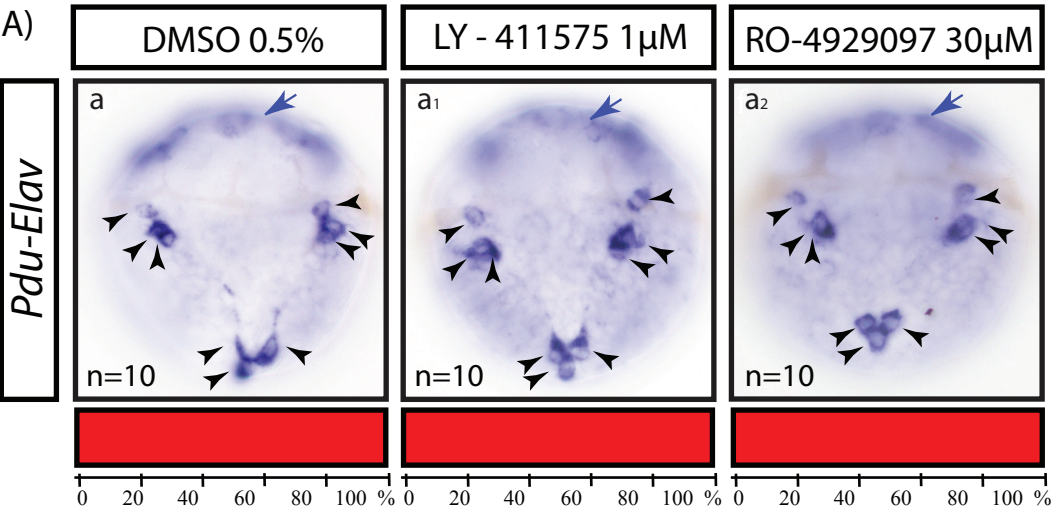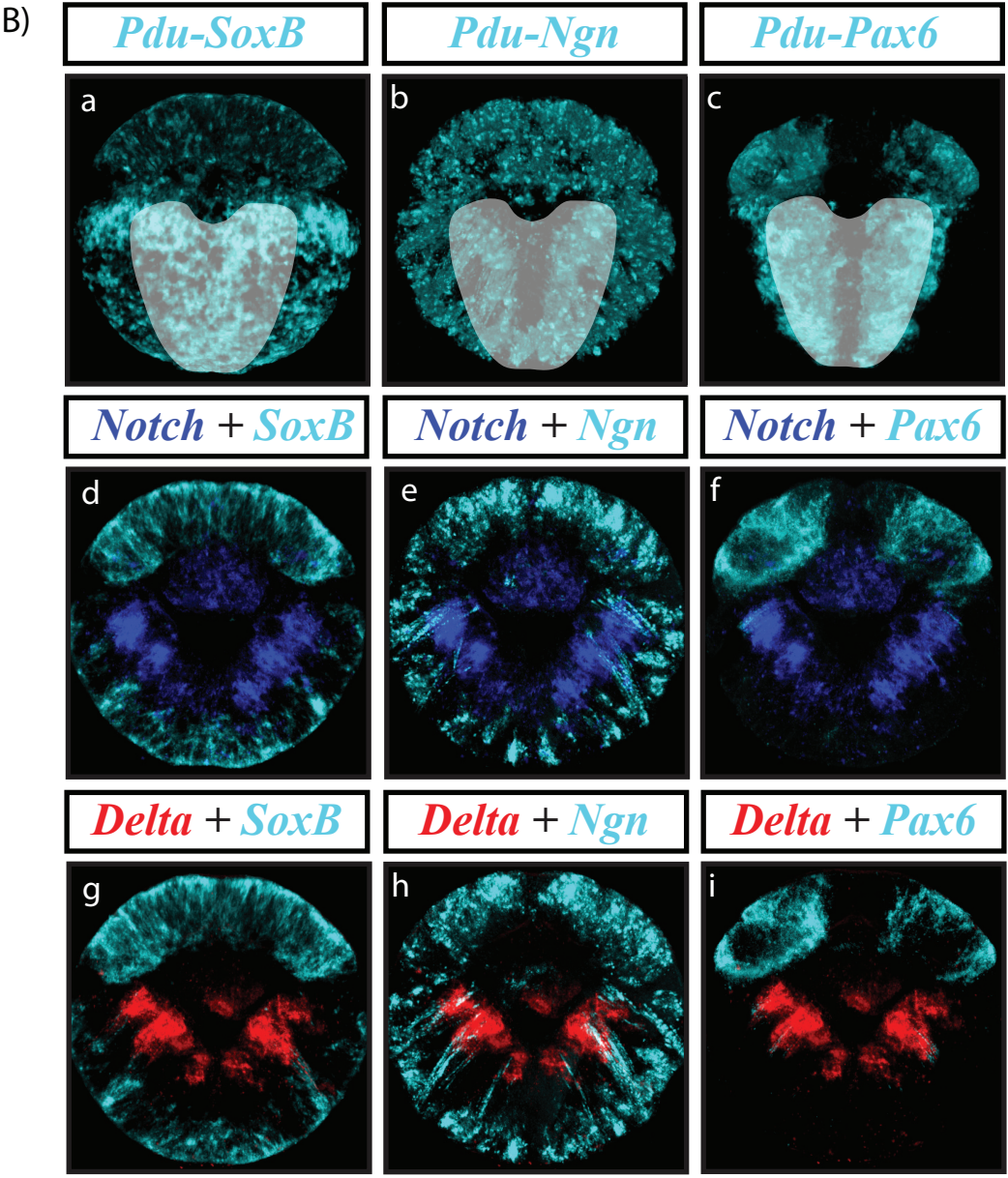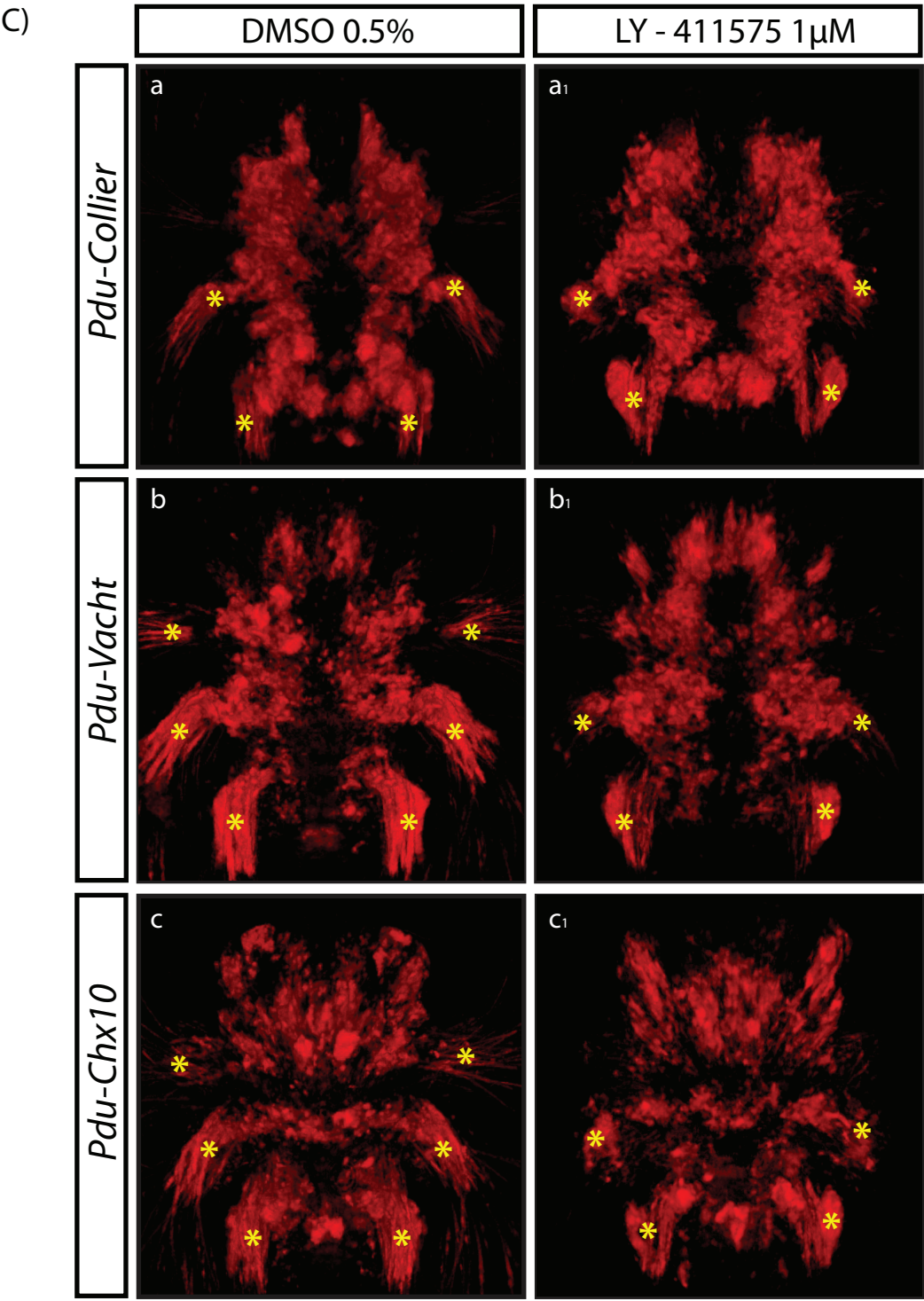

Supplement: Figure S4: The Notch pathway in the annelid Platynereis: Insights into chaetogenesis and neurogenesis processes”; Figure S1: Eve Gazave, Quentin I. B. Lemaître and Guillaume Balavoine [file rsob160242supp5.pdf]
