# Supplementary material for: The Notch pathway in the annelid Platynereis: insights into chaetogenesis and neurogenesis processes
Source: Open Biol. 2017 Feb 1;7(2):160242. doi: 10.1098/rsob.160242 (PMC5356439; doi:10.1098/rsob.160242)
Supplement: File S1 all sequences + ali_new [file rsob160242supp6.docx]

**Fringe sequences**

>Aca_524872

MRLSVRKVVKYSTFLTIILLLNVWISYNVNIWTFNNLNDRGKVAESLDTRRDVGNGGAWP

SPPNGAEGAVPGQEFFGVGGLGEVFRRQRSAKSVSVSYEGNNWRNAVGYKRGTDRDESLV

ASQQNKNLMFQKNGSLQVSVNRQAKPGAVSNKSGARARLPNQLRETELSDVFVSVKTTLK

YHKSRIQLILKTWYLLAREQIYFFTDTEDKEMKEALRDHVVNTNCSTDHSRQALSCKMAV

EFDYYMASRKRWFCHMDDDIYLNVPRLLKLLQQYDHRSDWYLGKPSLKHPLEIEDRKHPG

MKLAFWFATGGAGFCISRSLALKMAPHASGGRFMTTAETIRLPDDCTVGYIIEHLLQKQL

TVVPQFHSHLEALRLLKPSQLSEQITVSFSEYPGKANVVEVPGFSSAEDPTRFRSLHCFL

FPTFRECRQLS

>Hsa_L93140

MLKRCGRRLLLALAGALLACLLVLTADPPPPPLPAERGRRALRSLAGPAGAAPAPGLGAA

AAAPGALVRDVHSLSEYFSLLTRARRDAGPPPGAAPRPADGHPRPLAEPLAPRDVFIAVK

TTKKFHRARLDLLLETWISRHKEMTFIFTDGEDEALARHTGNVVITNCSAAHSRQALSCK

MAVEYDRFIESGRKWFCHVDDDNYVNLRALLRLLASYPHTRDVYVGKPSLDRPIQAMERV

SENKVRPVHFWFATGGAGFCISRGLALKMSPWASGGHFMNTAERIRLPDDCTIGYIVEAL

LGVPLIRSGLFHSHLENLQQVPTSELHEQVTLSYGMFENKRNAVHVKGPFSVEADPSRFR

SIHCHLYPDTPWCPRTAIF

>Hsa_M21536

MQCRLPRGLAGALLTLLCMGLLCLRYHLNLSPQRVQGTPELSQPNPGPPKLQLHDVFIAV

KTTRAFHRLRLELLLDTWVSRTREQTFVFTDSPDKGLQERLGSHLVVTNCSAEHSHPALS

CKMAAEFDTFLASGLRWFCHVDDDNYVNPRALLQLLRAFPLARDVYVGRPSLNRPIHASE

PQPHNRTRLVQFWFATGGAGFCINRKLALKMAPWASGSRFMDTSALIRLPDDCTMGYIIE

CKLGGRLQPSPLFHSHLETLQLLRTAQLPEQVTLSYGVFEGKLNVIKLQGPFSPEEDPSR

FRSLHCLLYPDTPWCPQLGAR

>Hsa_R74271

MSRARGALCRACLALAAALAALLLLPLPLPRAPAPARTPAPAPRAPPSRPAAPSLRPDDV

FIAVKTTRKNHGPRLRLLLRTWISRARQQTFIFTDGDDPELELQGGDRVINTNCSAVRTR

QALCCKMSVEYDKFIESGRKWFCHVDDDNYVNARSLLHLLSSFSPSQDVYLGRPSLDHPI

EATERVQGGRTVTTVKFWFATGGAGFCLSRGLALKMSPWASLGSFMSTAEQVRLPDDCTV

GYIVEGLLGARLLHSPLFHSHLENLQRLPPDTLLQQVTLSHGGPENPHNVVNVAGGFSLH

QDPTRFKSIHCLLYPDTDWCPRQKQGAPTSR

>Sko_585715

MRLRAKRTLQGLVLTAILCLIALQLSIPSNGSEITAEVERGGGHGDGVFDALPFMSEKVD

GAGISKALFHDRRHIQGLPDAQIDSVRRKAVDKFQQLNSSDEASLFSQDEGDIVDQRPAA

HKPKHGSLAQIHSNVYNRNRTQSNNSSNVQLGGYAKLSMGLLDMSQMVTSPVNSPRRTEL

SDIFIGVKTTKRYHAERLDLLLDTWVDMSLEQTYLFTDQEDYEYNKKLHGHLINTNCSSL

HTRQALCCKMSAMYDMFLESHKRWFCHVDDDNYLNVAQLVKLLQQYRHTDDVYLGKPSLS

HPIEAVDRTNNMRRVSFWFATGGAGFCLSRAMALHMSPYASGGSFSSMCNRIRLPDDVTV

GFIVEVLLKKKLTKVSEFNSHLEALWQIKKLDLPKQVTLSYSKGTKKYKNVITLDNRVFS

DEQDPTRLKSLHCMMFPQVGECALMARPPSLLSGLDDGWPHLGKGNVV

>Tca_189241

MVFHVGTNLRAPFAHAPISPLPLKGTRGTITNNNQHTTWCPSQSHIVYFSESLVSIVAVV

HVRQHLCPFPLENYVVDLLFSPMSCSGDQVIFEMNARHRRFLQTLSLAALLAYTALFAYQ

TLSRGGVADEENVLAEKRHQRAPLEEASVTSESSSATATLRPPSTVLDDVFISVKTTKNY

HRQRLPIILKTWFQLAKAQTWFFTDTDDPEFQHKTNGHMINTNCSSSHNRKALCCKMSVE

FDTFIDTDKKWFCHFDDDNYVNVPRLVRFLGDYNPREDWYLGKPSIQAPLEIINKEKKPT

VNQKVKFWFATGGAGFCLSRALALKMMPVASGGKFISTGEKIRLPDDVTMGYIIEHLLKK

PLTVVDQFHSHLEPMKFIRREILEEQISFSYSRNKDEWNVVKIEGFDTKYDTNRFLSLHC

FLFPHFNFCPR

>Isc_009494

MSVELDFFLDSNKKWMCHFDDDNYVNVPRLVRLLQGYDPREDWYLGKPSIRQPLEILARD

SGSPPQKISFWFATGGAGFCISRSLALKMLPIAGGGKFISIGEHIRLPDDVTMGYIVEHL

LKKKLTVVENFHSHLEPMKFLKKEALSDQVTFSYSRFGKEMNVLSIDGFPYRVDPTRFLS

LHCHLFPNFSFCPR

>Pdu_fng

MRIPVKKGVQAFLLACCVLGVNVLYVFLQQEIKQHSSAAEFFQQDGGKQSQEDVPKLGRS

LFERSRRSMXNESYNLKENVTDQGEGVNGKNKAFLSHKADSKSSKTVSQVFQDPSKKSSA

FHHTNNNNNNNKILKNNAQFTNGYNHNNAVYKNEVKLSEVFVSVKTTGKFHASRIPILLE

TWVVMGRDAVHFFTDVEDEELKSKILPGRLINTNCSARHTRAALCCKMSLEFDMFLASKR

KWFCHVDDDTYLNIPALLRLLKQYNSTGDWYLGKPSLNHPLEIKDIEYPKQKTAFWFATG

AGFCVSRGLALKMMPYAGGGRLRSVCEKVRLPDDCSIGFIIYYYMKKELTVVPGFHSHLE

GLWLIKPRDLENHITFSYGQSGHATNVVRVGGFSQGSDPTRFRSIHCHLYPNLPQCANIE

KDFR

>Cte_224315

MTTYFFTDEEDEEFSKRTKGHLINTNCTAGHTRRCVVKWRWSTIRLLPQRKGHPLQMGDK

ENPGQKIAFWFATGGAGFCISRGLALKMMPHTSGGRLKTVCEHIRLPDDCSIGYIISFKL

KKELTIVKDFHSHLEGLWKINHRNIEDQITMSYMCMSSGASSKPSPSRNGCNSVDISSGF

PPHVDPTRFLSIHCLLYPNLAMCQEIS

>Lgi_142624

MTNILLCYFIPENVPSRRSERQEILANEDNGRYGGVQLPKRIDNKHHFGRLEWKSQVAKD

KSIATKNDLGNANINSSSRETDISDVFISVKTTARNHQLRLRILLNTWILLAREQTHIFT

DTDDPALDKERENGVEIINTKCPPNHSRRALCCKMAIEYDTFLASKKRWFCHVDDDNYVN

IPQLVKLLRQYNHTQDWYLGKPSLRKPLEIMDRKHSGQKIAFWFGTGGAGLCISRSLALK

MMPYASGGRLMTIGESIRLPDDCTMGYIISHLMKKQLTVIEQFHSHLESLNLMSKRDLVN

QITYSYKYQDKTKNILNIDGFSIKQDPTRLWSLHCFLFPTFRECNRLGS

>Cgi_100084

MDMAPMGHLLNRSGTYIFTDASDFSLIEKMPGNPYALLQKYNHTENWYLGKPSLSHPLEI

QSRSREGQKVAFWFATGGAGFCISKALGIAMVPEAGGGKLIKVGDSIRLPDDCTIGYIIN

HILKVDLTRVDLFHSHLESQFRIQDPKKHITLSYNEQNVASVPGFSLKDDPTSDPEEGDM

KLIWAVSTPTLCVSDDRLPTQKMKCKVHPDQDSGVLCCQQCEAPVCMKCISTERHHGHSF

VAASEMVVSMRDKCHEEIAKIKDTIIPNCRLVVQELGKNQDTSKHLLSQVRTTMTSKIRK

MKDILDDILSNNMKELTDIEHVLMHEYQDEVHKMNEHLARLQEVLDEFEKKGQHMESGDL

MSIYNRIQKLKEIPSLKLHHDHPTFQEGSGQMDAETLEALFGKLTVPKYLKNSMQKIHPN

KPKLAMRIQKVKAIHVPDIDRCRHISCMKSNKTWVGDELGNLVEIDPKGNELHKIKTHHF

RQQPTGFHAVTLEDELLFIDQYDDSISKLLSGRKKKTLIKFKEWTPISIHVSHSSDDLLV

GMVDDTQQKAKVVRYDASGKELKTYEFHEDSKKRLYNRPHYLSENINGDIVVSDWEKQVV

VAVGMTGNHRFDYDAEGGHPPGTIFEPSGICTDILGNILVCDNNDHLDDSVHMLDKDGRF

LCLLLRLPSSSLERRIRALGLDDSCNLWIGDVDTNEIRVYKYLE

>Nve_198546

MFFPRRKLRIFLTWILLAALLQILIYAVLFVDVSNVPRTISRSKNRHLVQASQLQRPERP

GITLQLKDIGSLGMDLDRAQAIPDSRTLNFAKDSLNAMSTSAPELKKTAQVLMTEEIYDL

LTTAIKKTGPMSTLAEPDLKNVLTLEDGRPTGPSDVFLAIKTTRNYHQSRMQVLMKTWIS

LAKEQVYVFSDGDDPDLNKLLAALCCKMQAELNYFWEKSNERWFCHFDDDNYVNFPALIK

LLREHNHTYPHYIGKPSINHKMQGYDREKNYKMVSFWFATGGAGLCLSRVLVARMAEYFR

NHAFVQTCDRIGLPDDCVLGYIAEYALGVKLKKSNLFHSHLEGLRFIRQEELRDQVTFSH

GYMGSFYNRLKISGPFSEAIDPSRYVVGGNVFVLSDQGSCRCIVCCTLEQFGAPRIYEHI

S

>1Aqu_34037

MSRAKVYIVVGIGLALLLLVIFYETGTNNVVKRNITYLRVPHIMTLATDRPPAANLTTTV

QPTVNTNDNENPSSVSSPITTPAGSTPTSHTSSVQSPSDIPYIDPRTLNESGFIEYSLGV

SPLSDMKLVGHRVPEKILIPVDISDIFISVKTSSLNAYRQQTVFLTWAQTVPIDQISFTT

DKATNWTDAFAAHGYKINTAPHCGLGHTDYSLCCKSGVEYDQFYKAIEAGRNFSWMCHID

DDEYMNVWKLKRMLIKYDPNKPWYIGKSHHEYWKLNPSNSGNFPEAKRKIYKFNTGNVYC

LSKQIMKETEKYFRGRNFIKTCEKAHWIDDVTIAIVIVAVLGYEPTEEKQMWSHYEVLDQ

LPKSESLKMINFGYGKLEFGFHGRWNTSLNLPNPRFSYNADPTRHGISLLSLYDVS

>3Aqu_34037

MIVFGARRKLILAIVFLFLLVIGVVNKLRQLPNSIVNQLEIEHRSHQPKRSYFLPETGSQ

TNPHQNPSTLFQNEYWLLGEEAILEKMQRPSRKLSDFESAGNNIMIAIRTTKKFHQKRLP

YLYDTWLNKVNGSNVFLVTDAEDEEYQERSKQLGIHYVVSSLCGESLLWPIPSRWYLCCR

TGEALTLIWFCYLDDDIYLIMENLIKLIAKFPKDELSYIGRPGTPWEKPHKNSTRKHYHF

ASGGFYCLSRTILDKIKPWIVGGHNLGDTCRQLLEPDDLTIGCAVELLGGGKLSRTLLFH

HHGMNLAKAVNANTLKDQIAIPYGCGQMCKYGGIVDNAIEVPNAAFSFDEDPSRFRSLHC

HLYPQATICK

>4Aqu_34037

MITIRTTRKFHQKRLPYMYDTWLNKVNGSNVFLVTDAEDEEYQEKSRQLGIHYKIISCGK

DYSRWSLCCKSGEEMALMHRPENKQYSWFCHLDDDIYIILKNLVNLLSKFDPLKEPIYMG

RAGTHWKKPFKLSKKQKMLEPQNVHPFHFAVGGMYCLSRAMLDKVKPWLGNGETMGDTCN

KLLQPEDVAVGATVELLAKERLSRTKLFHPHGLILSRFVNPRTLKDQIGFAYGCGQLCAY

KGYKNNAIKVPNARFPFSEDPSRFKSLHCHLFPDSALCK

>5Aqu_34037

MSRRYLVVASSVVILIVVVLALSYQKDTLSSTGLSRLHKYDPTHPRPGTTGIISLIANGS

SSPPPVSIYHTQTPSDTTHSTPPAVSKASTQATSVSLINPKGLAEDGFIEYSLGVSPLSD

MKLVGHRVPEKILIPVDISDIYFSVKTASIYPDRLNTVMLTWAQTVSIEQISFTTDKATN

WTDAFAARGYKINLAPHCGLGHEHYFSLCCKSGVEYDHFYKSIESGKNYNWLCHIDDDQY

YNVWKLKRMLIKYDPNKPWYIGKSHHGFTSYNSFPFPEAKRHDYKFNTGNVYCLSKQTMK

EIEKYLRAKNFPKSCDKTRQPDDVTIAIIIVGVLGHTPTEEKQMWSHLELLDQLPKEESM

KMISFGYGHLEGAYHGRWNTSLNLPNPRFSYNADPTRFLSYHCMVYPKLKWCQ

>6Aqu_34037

MKHKIGMALLCLIFSLLMLLFYLSKITNTSHQSISTTVLVSSRHRNLIIIKPNTTLDEAS

TDPTSVTTQPARASFMNPNDILESGFIEYSLGVSPLSDMKLVGHRVPEKILIPVDISDIY

FSVKTASIYPDRLNTVMLTWAQTVSIEQISIITDATTNWTDAFSSRGYSISAATQCGLGH

SVQSLCCKSGVEYDRFYKSIESGKKYNWFCHIDDDQYYNVWKLKRMLIKYDPNKPWYIGK

SHHGPTPVSNLSTDPTIYLFIYMFVLAVFLFETEFSGSVGVLGHKQTEEKQMWSHLELLD

QLPKEESMKMISFGYGHLEGAYHGRWNTSLNLPNPRFSYNADPTRFLSYHCMVYPKLKWC

Q

**Fringe alignment**

>1Aqu_34037

KILIPVDISDIFISVKTSS-LNAYRQQTVFLTWAQTVPIDQISFTTDKATNWTDAFAAHG

YKINTAPHCGLGHTDYSLCCKSGVEYDQFYKAIEAGRNFSWMCHIDDDEYMNVWKLKRML

IKYDPNKPWYIGKSHHEYWKLNPSNSGNFPEAKRKIYKFNTGNV-YCLSKQIMKETEKYF

RGRNFIKTCEKAHWIDDVTIAIVIVAVLGYEPTEEKQMWSHYEVLDQLPKSESLKMINFG

YFGFHGRWNTSLNLPNPRFSYNADPTR----HGISLLSLYDVS

>5Aqu_34037

KILIPVDISDIYFSVKTASIY-PDRLNTVMLTWAQTVSIEQISFTTDKATNWTDAFAARG

YKINLAPHCGLGHHYFSLCCKSGVEYDHFYKSIESGKNYNWLCHIDDDQYYNVWKLKRML

IKYDPNKPWYIGKSH--HGFTSYN-SFPFPEAKRHDYKFNTGNV-YCLSKQTMKEIEKYL

RAKNFPKSCDKTRQPDDVTIAIIIVGVLGHTPTEEKQMWSHLELLDQLPKEESMKMISFG

YGAYHGRWNTSLNLPNPRFSYNADPTRFLSYHCMVYPKLKWCQ

>6Aqu_34037

KILIPVDISDIYFSVKTASIY-PDRLNTVMLTWAQTVSIEQISIITDATTNWTDAFSSRG

YSISAATQCGLGHSVQSLCCKSGVEYDRFYKSIESGKKYNWFCHIDDDQYYNVWKLKRML

IKYDPNKPWYIGKSH-HGPTPVSNLS-----TDPTIYLFI-----YMFVLAVFLFETEFS

GS----------------------VGVLGHKQTEEKQMWSHLELLDQLPKEESMKMISFG

YGAYHGRWNTSLNLPNPRFSYNADPTRFLSYHCMVYPKLKWCQ

>3Aqu_34037

LSDFESAGNNIMIAIRTTKKFHQKRLPYLYDTWLNKVNGSNVFLVTDAEDEEYQERSKQL

GIHYVVSSCGESLSRWYLCCRTGEALTLI-----------WFCYLDDDIYLIMENLIKLI

AKFPKDELSYIGRPGWEKPHKNSTRKHY---------HFASGGF-YCLSRTILDKIKPWI

VGGHNLDTCRQLLEPDDLTIGCAVELLGGGKLSRTLLFHHHMNLAKAVNANTLKDQIAIP

YCKYGGIVDNAIEVPNAAFSFDEDPSRFRSLHCHLYPQATICK

>4Aqu_34037

-----------MITIRTTRKFHQKRLPYMYDTWLNKVNGSNVFLVTDAEDEEYQEKSRQL

GIHYKIISCGKDYSRWSLCCKSGEEMALMHR--PENKQYSWFCHLDDDIYIILKNLVNLL

SKFDPKEPIYMGRAGWKKPFKLSKKQKMLEPQNVHPFHFAVGGM-YCLSRAMLDKVKPWL

GNGETMDTCNKLLQPEDVAVGATVELLAKERLSRTKLFHPHLILSRFVNPRTLKDQIGFA

YCAYKGYKNNAIKVPNARFPFSEDPSRFKSLHCHLFPDSALCK

>Nve_198546

EDGRPTGPSDVFLAIKTTRNYHQSRMQVLMKTWIS-LAKEQVYVFSDGDDPDL-NKLLAA

-----------------LCCKMQAELNYFWE--KSNER--WFCHFDDDNYVNFPALIKLL

REHNHTYPHYIGKPSINHKMQGYDREKN---YKMVSFWFATGGAGLCLSRVLVARMAEYF

RNHAFVQTCDRIGLPDDCVLGYIAEYALGVKLKKSNLFHSHLEGLRFIRQEELRDQVTFS

H-GYMGSFYNRLKIGP--FSEAIDPSRYVVGGVFVLSDQGSCR

>Hsa_M21536

PGPPKLQLHDVFIAVKTTRAFHRLRLELLLDTWVS-RTREQTFVFTDSPDKGL---QERL

GSHLVVTNCSAEHSHPALSCKMAAEFDTFLA---SGLR--WFCHVDDDNYVNPRALLQLL

RAFPLARDVYVGRPSLNRPIHASEPQPHNR-TRLVQFWFATGGAGFCINRKLALKMAPWA

SGSRFMDTSALIRLPDDCTMGYIIECKLGGRLQPSPLFHSHLETLQLLRTAQLPEQVTLS

YGVFEGK-LNVIKLGP--FSPEEDPSRFRSLHCLLYPDTPWC-

>Hsa_L93140

PLAEPLAPRDVFIAVKTTKKFHRARLDLLLETWIS-RHKEMTFIFTDGEDEAL--ARHTG

--NVVITNCSAAHSRQALSCKMAVEYDRFIE---SGRK--WFCHVDDDNYVNLRALLRLL

ASYPHTRDVYVGKPSLDRPIQAMERVSENK-VRPVHFWFATGGAGFCISRGLALKMSPWA

SGGHFMNTAERIRLPDDCTIGYIVEALLGVPLIRSGLFHSHLENLQQVPTSELHEQVTLS

YGMFENK-RNAVHVGP--FSVEADPSRFRSIHCHLYPDTPWC-

>Hsa_R74271

PAAPSLRPDDVFIAVKTTRKNHGPRLRLLLRTWIS-RARQQTFIFTDGDDPEL---ELQG

GDRVINTNCSAVRTRQALCCKMSVEYDKFIE---SGRK--WFCHVDDDNYVNARSLLHLL

SSFSPSQDVYLGRPSLDHPIEATERVQGGRTVTTVKFWFATGGAGFCLSRGLALKMSPWA

SLGSFMSTAEQVRLPDDCTVGYIVEGLLGARLLHSPLFHSHLENLQRLPPDTLLQQVTLS

HGGPENP-HNVVNVGG--FSLHQDPTRFKSIHCLLYPDTDWC-

>Cgi_100084

------------------------------------LNRSGTYIFTDASDFSL-IEKMPG

------------------------------------------------------NPYALL

QKYNHTENWYLGKPSLSHPLEIQSRSRE---GQKVAFWFATGGAGFCISKALGIAMVPEA

GGGKLIKVGDSIRLPDDCTIGYIINHILKVDLTRVDLFHSHLESQFRIQ--DPKKHITLS

Y----NE-QNVASVPG--FSLKDDPTRIQKVKAIHVPDIDRCR

>Pdu_fng

VYKNEVKLSEVFVSVKTTGKFHASRIPILLETWVV-MGRDAVHFFTDVEDEELKSKILPG

--RLINTNCSARHTRAALCCKMSLEFDMFLA---SKRK--WFCHVDDDTYLNIPALLRLL

KQYNSTGDWYLGKPSLNHPLEIKDIEYP---KQKTAFWFATG-AGFCVSRGLALKMMPYA

GGGRLRSVCEKVRLPDDCSIGFIIYYYMKKELTVVPGFHSHLEGLWLIKPRDLENHITFS

YGQSGHA-TNVVRVGG--FSQGSDPTRFRSIHCHLYPNLPQC-

>Cte_224315

---------------------------------------MTTYFFTDEEDEEF-SKRTKG

--HLINTNCTAGHTRRCVVK--------------------W-----------RWSTIRLL

PQRKG------------HPLQMGDKENP---GQKIAFWFATGGAGFCISRGLALKMMPHT

SGGRLKTVCEHIRLPDDCSIGYIISFKLKKELTIVKDFHSHLEGLWKINHRNIEDQITMS

YGASSKPSPNSVDISG--FPPHVDPTRFLSIHCLLYPNLAMC-

>Sko_585715

NSPRRTELSDIFIGVKTTKRYHAERLDLLLDTWVD-MSLEQTYLFTDQEDYEY-NKKLHG

--HLINTNCSSLHTRQALCCKMSAMYDMFLE---SHKR--WFCHVDDDNYLNVAQLVKLL

QQYRHTDDVYLGKPSLSHPIEAVDRTNN---MRRVSFWFATGGAGFCLSRAMALHMSPYA

SGGSFSSMCNRIRLPDDVTVGFIVEVLLKKKLTKVSEFNSHLEALWQIKKLDLPKQVTLS

YSKGTKKYKNVITLDNRVFSDEQDPTRLKSLHCMMFPQVGECA

>Aca_524872

NQLRETELSDVFVSVKTTLKYHKSRIQLILKTWYL-LAREQIYFFTDTEDKEM-KEALRD

--HVVNTNCSTDHSRQALSCKMAVEFDYYMA---SRKR--WFCHMDDDIYLNVPRLLKLL

QQYDHRSDWYLGKPSLKHPLEIEDRKHP---GMKLAFWFATGGAGFCISRSLALKMAPHA

SGGRFMTTAETIRLPDDCTVGYIIEHLLQKQLTVVPQFHSHLEALRLLKPSQLSEQITVS

FSEYPGK-ANVVEVPG--FSSAEDPTRFRSLHCFLFPTFREC-

>Lgi_142624

SSSRETDISDVFISVKTTARNHQLRLRILLNTWIL-LAREQTHIFTDTDDPALDKERENG

-VEIINTKCPPNHSRRALCCKMAIEYDTFLA---SKKR--WFCHVDDDNYVNIPQLVKLL

RQYNHTQDWYLGKPSLRKPLEIMDRKHS---GQKIAFWFGTGGAGLCISRSLALKMMPYA

SGGRLMTIGESIRLPDDCTMGYIISHLMKKQLTVIEQFHSHLESLNLMSKRDLVNQITYS

Y-KYQDKTKNILNIDG--FSIKQDPTRLWSLHCFLFPTFREC-

>Tca_189241

LRPPSTVLDDVFISVKTTKNYHRQRLPIILKTWFQ-LAKAQTWFFTDTDDPEF-QHKTNG

--HMINTNCSSSHNRKALCCKMSVEFDTFID---TDKK--WFCHFDDDNYVNVPRLVRFL

GDYNPREDWYLGKPSIQAPLEIINKEKKPTVNQKVKFWFATGGAGFCLSRALALKMMPVA

SGGKFISTGEKIRLPDDVTMGYIIEHLLKKPLTVVDQFHSHLEPMKFIRREILEEQISFS

YSRNKDEW-NVVKIEG--FDTKYDTNRFLSLHCFLFPHFNFC-

>Isc_009494

------------------------------------------------------------

---------------------MSVELDFFLD---SNKK--WMCHFDDDNYVNVPRLVRLL

QGYDPREDWYLGKPSIRQPLEILARDSGSP-PQKISFWFATGGAGFCISRSLALKMLPIA

GGGKFISIGEHIRLPDDVTMGYIVEHLLKKKLTVVENFHSHLEPMKFLKKEALSDQVTFS

YSRFGKE-MNVLSIDG--FPYRVDPTRFLSLHCHLFPNFSFC-

**Numb sequences**

>Hsa_347169

MNKLRQSFRRKKDVYVPEASRPHQWQTDEEGVRTGKCSFPVKYLGHVEVDESRGMHICED

AVKRLKAERKFFKGFFGKTGKKAVKAVLWVSADGLRVVDEKTKDLIVDQTIEKVSFCAPD

RNFDRAFSYICRDGTTRRWICHCFMAVKDTGERLSHAVGCAFAACLERKQKREKECGVTA

TFDASRTTFTREGSFRVTTATEQAEREEIMKQMQDAKKAETDKIVVGSSVAPGNTAPSPS

SPTSPTSDATTSLEMNNPHAIPRRHAPIEQLARQGSFRGFPALSQKMSPFKRQLSLRINE

LPSTMQRKTDFPIKNAVPEVEGEAESISSLCSQITNAFSTPEDPFSSAPMTKPVTVVAPQ

SPTFQANGTDSAFHVLAKPAHTALAPVAMPVRETNPWAHAPDAANKEIAATCSGTEWGQS

SGAASPGLFQAGHRRTPSEADRWLEEVSKSVRAQQPQASAAPLQPVLQPPPPTAISQPAS

PFQGNAFLTSQPVPVGVVPALQPAFVPAQSYPVANGMPYPAPNVPVVGITPSQMVANVFG

TAGHPQAAHPHQSPSLVRQQTFPHYEASSATTSPFFKPPAQHLNGSAAFNGVDDGRLASA

DRHTEVPTGTCPVDPFEAQWAALENKSKQRTNPSPTNPFSSDLQKTFEIEL

>Hsa_442759

MNKLRQSLRRRKPAYVPEASRPHQWQADEDAVRKGTCSFPVRYLGHVEVEESRGMHVCED

AVKKLKAMGRKSVKSVLWVSADGLRVVDDKTKDLLVDQTIEKVSFCAPDRNLDKAFSYIC

RDGTTRRWICHCFLALKDSGERLSHAVGCAFAACLERKQRREKECGVTAAFDASRTSFAR

EGSFRLSGGGRPAEREAPDKKKAEAAAAPTVAPGPAQPGHVSPTPATTSPGEKGEAGTPV

AAGTTAAAIPRRHAPLEQLVRQGSFRGFPALSQKNSPFKRQLSLRLNELPSTLQRRTDFQ

VKGTVPEMEPPGAGDSDSINALCTQISSSFASAGAPAPGPPPATTGTSAWGEPSVPPAAA

FQPGHKRTPSEAERWLEEVSQVAKAQQQQQQQQQQQQQQQQQQQQAASVAPVPTMPPALQ

PFPAPVGPFDAAPAQVAVFLPPPHMQPPFVPAYPGLGYPPMPRVPVVGITPSQMVANAFC

SAAQLQPQPATLLGKAGAFPPPAIPSAPGSQARPRPNGAPWPPEPAPAPAPELDPFEAQW

AALEGKATVEKPSNPFSGDLQKTFEIEL

>Sko_259013462

MKKLRRSLSKRETYVPECSKPHQWQEDEKLVKAGTCNFPVKYLGSVEVNESRGMPICEDA

VRKLRDKKKVRAVLWVSSDGLRVVDEDSKGLIVDQTIEKVSFCAPDRRHERAFSYICRDG

TTRRWLCHAFIAVRDSGERLSHAVGCAFAACLERKQKRDKECGVKVEFDVNKTSFARQGS

FREPTMTEKLEQQKREAEERSRTDGAQLPLEHVPPRAVNLPYAVPRRHATPNMLERQGSF

RGFPGLAKDSPFKRQLSLRLNELPSTLARQKDLNILPEENGSPNSPLSPSGQNQSQQIPT

NRPTQVSSSSSAAAGSADISSLCQQLTQGLSELSNQDPFADFTPTSSTSMTPVSPVDMFS

PTRQVPSRSPITSPIAASNTALPVRQTNPWENSTPKGVQAQPSEPTWATAQIPNQQTKPA

WTPGHRRQLSDADKWLETASETVAVVNSPGNPFASPDSPQMATTVVKSRSNPYNLSTQKT

TPTSERPPLVKSHTIGDPFTPLPPQPQPQQWPTGSTNQHNGFQSSNYMNTKPPPVVPQRT

YNGGLNSSWTNNTHQISAPFQSAQQHQVHQSDFDAQWNALSNAQPSHTENPFANAVKTFE

INL

>Tca_189240899

MGNHPSNHQPLERTNVGKFGDPHTRRSLRSPRKMDRLRRSFRDSFRRRKDRVPESSKPHQ

WQADESAVRSGTCTFAVKYLGCVEVFESRGMQVCEEALKVLRGSRRRPVRAVLHVSGDGL

RVVEDESKGLIVDQTIEKVSFCAPDRNHERGFSYICRDGTTRRWMCHGFLALRESGERLS

HAVGCAFAVCLERKQKRDKECGVTMMFDTKNATFTRSGSFRQQGLTERLERGVDVGPPRP

APVNPFAIERPHATPSMLQRQGSCRGFSTLGQNSPFKRQMSLRVNELPSNTARLNAYKSP

TSPTNPKPQVSPIPEISPGDSVTALCQQLSQGLSQLTHSGSDDFNFNNQTSINQNTVNLS

VTTINNSFSPNLSQSQVTTSITSNASSLPKPDQWLGQIVNSTSPVPAKMDGVSPRRGPLL

GAHSRAMSLDSSGAFQRTADPFDAEWADIAAKNNPTNPFLSSAAPQPFQIQL

>Isc_021614-PA

MDRLRRSLRDSLRRGRREHVPECSKPHQWQADEAAVRAGSCTFPVKYLGCVEVFESRGMQ

VMLLPLSELPALFRPIIFRNALIIRHVLVRFPSSDRPLSDFIVRQVFVRLGKASYYETSH

RWACLLRPIIRLIFISLRRRPMPCPQGERLSHAVGCAFAVCLERKQKRDKESVLMNFDPK

TSSFTRTGSFRQGSITDSLPQDPQEAKPSGESPRKSFLALAEQRGRCDAHVRVVSCRAEA

PPVKSVVNPYAIERPHATVSMLQRQGSFRGFGSLSQTSPFKRQLSLRLNELPSNIERQRA

MSLDASGDLPSR

>Pdu_numb

MEKLKRSLSFRKKKDHVPESSKPHQWQEDERKVREGTCSFQVRYLGCIEVFESRGMQVCE

EAVKALKARQNRSVWSRMSLRRKKKKPEEQCKGKYQRAILYVSGDALRVVDEISKGLIVD

QTIEKVSFCAPDRNHEKGFAYICRDGTTRRWMCHGFLALKESGERLSHAVGCAFAICLEK

KQKREKDAVQVQYNEKGTSFSRTGSFRQATLTERLCDPQSTILSESVPVKAVDNPHAVSR

PHATADMLQRQSSFRGFTKLGDQSPFKRQLSLRLSDLPSTLQRQQDMNNPANGISNGLSA

SPIPEASPTKENPDTIAEMCQQLTQGLTALQTDDPFANVPNVTQRNITSPTSTVSHTPSP

PATAPHQHTVTVQGRSIPLPDPIRRQMPWAPSGGPPGRRPPGGSVSXAEAWLSNTASEVG

RPSEVGRPEPYAAAGQAPGYVANGNSMNQYNVGPGYNTGAAYPGQVASVAQHRAPHLTHL

RSHSIDTAQIWDQQQQQQRAPTLRQLAHSGYPIGQFTSQQNGAGGTWSPPPHAPQQQAFD

PFDAAWAAKKVGVANPFNGQGDTVSKSFEVNL

>Cte_P228321

MTVCEEACKALRSQSKGKYQRAILYVSGEALRVVDEINKGLILDQTIEKVSFCAPDPHNL

KGFSYICRDGTSRRWMCHGFMAVKDSGERLSHAVGVAFTVCLEKKQEREKEAVSVAFNDK

GTSFTRTGSFRQATLTERLEDPQSAIVAEPVPQKQIFNPYAVQRPHAKDAMLERQGSFRG

FTKLAESSPFKRQLSLRLNELPSTLDRQKQVLNQQSSVDNGFSPIPEASPSKEDVDSIAA

LCQEVSQGLNALGGPKPNNNMQNFQHTSSIPAAEKGLQNNNPWATSSSVAESWLASSATV

AASPFAPPPPPPQGASPSFNPASRAPHLAEVRSHSLDSTQRNGSTAAWSATPQATTFDPF

DAAWAASGRPSSTNPFQTPDNVTTAFKVNL

>Hro_P63899d

MDKFKRTLSLKKKKESSGGESIKPHQWQEDERKVREGSCSFQVKYLGCIEVFESRGIQVC

EDAVKALKNSGHHMAVLYVTGDALRVVDEISKGLIVDQTIEKVSFCAPDRNHEKGFAYIC

RDGTTRRWMCHGFLAVKESGERLSHAVGCAFAICLEKKQQREKEKVTVTYNDNGTSFSRL

GSFRQATMTERLTDPQSAILAGRSLIHL

>Hro_P126290d

QQSIPQLWLEDEVKVKNATCNYPVEYLGYTEVFESRGVVVCEKAIRALKSQTRRRCKKAV

IHISGDAIRVVDEINKVLLVDQTLEKVSFCAPDRNLNEGFAYICRDASTKRWLCHGFLAI

KDSGDRISHALGTAFAVCLERKVNREKLLAS

>Aca_524887937

MERKGSKRFGRYRNIMQSIRRRFSLRKKKDHASESARVDQWEEDTNKVRNGTCSFQVRYL

GYIEVFDSRGMHICEEAVKALKAQCKGKYQRAVLYVSGDALRVVDEVSKSMIVDQTIEKV

SFCAPDRHNEKAFAYICRDGTTRRWMCHGFLAVKESVSGERLSHAVGCAFAICLEKKQKR

GSETGVTVTYNQSRTSFERTGSFRQTTLTERIVDPQSAILAEPVPVKSANNPYAVQRPHA

SANLLERQGSFRGFEKLNEISSPFKRTTSLRLNELPSTLQRQNAVLESPTHNGNGALQTI

NQSVNPWGPVAVNPPSTWGAAAAAVPAPPQMATINSSSNSNPFVSSGPAFVPQPGRLNNG

GAPTTMLQQQQQQQNMFAHNRSHSIDTGEMSSTHWQRNHHQVQKAQPSATTMPHQVQQNG

GVASVPSSASSNAAAMGSSSPWPTSAPPVPSSQGPSVDPFDVAWAAKSVNQSSANPFASK

SVKKFEVQL

>Lgi_164463

METGKSIRRFYVNNIMQSLKRRLSFRKKKDHVPECSKPHQWQEDEKKVREGTCSFQVRYL

GCIEVFDSRGMQVCEEAVKALKNQAKGKVQRAVLYVSGDALRVVDEISKSMIVDQTIEKV

SFCAPDRNHEKGFAYICRDGTTRRWMCHGFMAVKESGERLSHAVGCAFAVCLEKKQKRDK

ETGVTVTFSEDRTSFTRTGSFRQTSMTERLADPQSAILAEPVPVRRVDNPFAVQRPHATP

ELFNRQGSFRGFEKLQEASSPFKRTLSLRYSDLPSTLQRQNAILESPSANGAVGGTIHEV

SPEHDDSIAQMCQQLTQGLSALTTDETFNNAPVQRGIQHHGLALHHQHSAPSFQPPQSTT

NTALPRQNTLPSSSLLSQHAPATNPWAQSSSNGTKQNLLWNKPTSPTNPFVSTQPPTGAT

SSVNGVPFTGDQFPRPPPAHQRSNAFQNLRSHSIDTGELSGSGWSHAQRNKQQPTLLELS

HQRSFQENGTDSSNWAASAVSTTSPTHSKTGVDPFDVAWAAKSVNKNIASNPYGGKTVKK

FEVQL

>Cgi_10003959

GKTSIILRFLDRDEAPKPTVALEYTFGRRAKGHNIAKDVGHIWELGGGTWLSKLMEVPLN

PDTLQHTTLAIVVDLSKPSEIWFTLETLLAAAKARVENILTEMKQEYPGLKEKLCKKAWE

RVGEEHSDKAMIDPFLIPLIIIGGKYDVFQAKDVGHIWELGGGTWLSKLMEVPLNPDTLQ

HTTLAIVVDLSKPSEIWFTLETLLAAAKARVENILTEMKQEYPGLKEKLHKKAWERVGEE

HSDKAMIDPFLIPLIIIGGKYDVFQDFDSEKRKVICKTLRFIAHTNGATLQFCGLKQEQL

VMRMRGLISHHLFGTTMGKSLQVDHNKPLMVAAGHDSLQQIGTPPLSEQDIGRVHAKNPM

QLWKHAFTGFFPQDDISNPALVDDPSKDPQYQEAAIDSLRSQKDEAGMESLKRRLSFRKK

KNHVPECSKPHQWQEDEKKVRDGTCSFQVRYLGCVEVFESRGMQVCEEAVKTLKSQCKGK

YQRAVLYVSGDALRVVDEISKSMIVDQTIEKVSFCAPDRNHEKGFAYICRDGTTRRWMCH

GFLAVKESGERLSHAVGCAFAICLERKQKRDKDSSTGVEVTFSQDKTSFTRMGSFRQTTL

TERITDPQSAILAEPVPVRKVDNPFAVERPKATPSMLVRQGSFRGFENLQKDSSPFKRSV

SLRLSDLPSTLQRQGAITESSPPKQTDVSAPIQEMSPSKEQEDSISHMCQQLTMGLSQLS

SDDAFGTTQRVETTPPHNLTSPAFQAPSPRHTNETNIPSHPAAPVQQTNPWASSSASPAH

TATSATNWPSPKATNPFANAPPISGASVSNGGAAVSNGGAFGQFPQQPQPPLRSQPPHLQ

HVRSHSIDTGELSTNQWQSHSRQQARPTLMDMAQQRSFQVNGSAWGESASGVSTKPTGPN

VDPFDVAWAAKSANKPTASNNNPFNSARGAQKTFEGERLSHAVGCAFAICLERKQKRDKD

SSTGVEVTFSQDKTSFTRMGSFRQTTLTERITDPQSAILAEPVPVRKVDNPFAVERPKAT

PSMLVRQGSFRGFENLQKDSSPFKRSVSLRLSDLPSTLQRQGAITESSPPKQTDVSAPIQ

EMSPSKEQEDSISHMCQQLTMGLSQLSSDDAFGTTQRVETTPPHNLTSPAFQAPSPRHTN

ETNIPSHPAAPVQQTNPWASSSASPAHTATSATNWPSPKATNPFANAPPISGASVSNGGA

SVSNGGAFGQFPQQPQPPLRSQPPHLQHVRSHSIDTGELSTNQWQSHSRQQARPTLMDMA

QQRSFQVNGSAWGESASGVSSKPTGPNVDPFDVAWAAKSANKPTASNNNPFNSARGAQKT

FEVKL

>Sme_5843.00.01

LQTSPPLHPIIVGRDARSVPDVDQYNPQVYPINSRMLCVFVDLNTIVASIMERFKRTFTL

RKKKPTSKALLDSNTKPSQWNDDEKKVREGCCSFQVKYLGSIEVFESRGMPVCEEAMKQL

LKYKSKKKSKRAILYVSGDALRVTEEISKGLIVDQVIEKVSFCAPDRNHKRGFAYICRDA

TTRRWLCHGFKAVKES

>Sme_2653a

MSFSSPRIFIHCPICIHYLSVKFQKKTIRAILYVSGDTIRVSDEISKGLIVDQTIEKVSF

CSPDRNHKKGFAYICRDGTTRRWMCHAFLAVKET

>Sme_2653b

MEKFKKSISFRKKKEKCQDEDPNNKPVSWQEDERKVREGCCSYIEVFDSRGMQVCEEALK

ALRRPKLENVYF

>Nve_241883d

MTHLITHARVRSSTSETIEPYNSLSNSQPSRLRVAESRILPSLVGMKRLKRSLSLRSSRR

RIPESHRPQVWENDSYKVRNGGVSFPVKYVGAIEVTESRGTQVCAEAFRKMREAGVHKKK

RMNLLVTSDCIRVVDEETKSLTIDQTIEKVSFCTPDPSDDRVFSYICREGTTRRWMCHCF

IAIRDTGERLSHAVGCAFTACLQRKQKAQALQQQAKAAKEGEKTDGTQATSTAPSTAANQ

QQSSRAFVSTPSSVTSASSVVNGPTETSGMTSSPSSTAPVVTTTQQVPPALKPPPAAARS

RPQPFAPSPFTRHMSLRYRSTPMSLTPLKDSGKAGYSTLLEDPGMAPKLPPAVDALGLQS

SPAVVPESHPVMQSNASLAYPQMQAAPLSSATVFNGMHPTSMAVNSTGSLPQAATPLQPN

TMQYTSPQTNQWPAAQPQYQPHGRPLERKVSDAEKWLASAEGSTKESNSNQGLLATHNPF

AETAAQISALSSAWTQDMNNSLPAASQSNGPWGSTGMHQVREDDEFASLATRRTGSPLSS

PGATANPFNKGSNVYWV

>Mle_00718ad

MKKIKKNIGQQKNGVSQQHYEADKDLVAKSGATFCVKYLGYEEVKEARGVKVCSVAVAKL

IKDKKNKKKMSLSISMDGVRVVDDISRVLVLDQPIEKISFCAPDSLNPKLFSYIARDGPC

RRWLCYGFYACDVPGERLSHALGCAFTACLQRKQKASKVTESNNSTGSVSSKGSSIESSP

ATQPAKGKPAATKPPITKANTTASITTITRPPPKEITVPIPDFSRDRSATTIGISTPPSQ

QAGQPRTQTNPFGSDFNPGDNFTADLADILLFDPNDEPDPQQEQRRRAATELPGSGGLDE

FDPLHPTFKSMQFEDNFKPPTVLAQNHVAGYPGNNFGYHGHQTLPQFSHTNPFSAAVSSE

SSGQMDDFDIFASGRLTLR

**Numb alignment**

>Hsa_347169

MNKLRQ----SFRRKKDVYVPEASRPHQWQTDEEGVRTGKCSFPVKYLGHVEVDESRGMH

ICEDAVKRLKGKKAVKAVLWVSADGLRVVDEKTKDLIVDQTIEKVSFCAPDRNFDRAFSY

ICRDGTTRRWICHCFMAVKDTGERLSHAVGCAFAACLERKQKREKECGVTATFDASRTTF

TREGSFRVTTATEQAEREEITSPTSDATTSLEMNNPHAIPRRHAPIEQLARQGSFRGFPA

LSQKMSPFKRQLSLRINELPSTMQRKTDFPIKNAVPEPTGTCPVDPFEAQWAALENKS

>Hsa_442759

MNKLRQ----SLRRRKPAYVPEASRPHQWQADEDAVRKGTCSFPVRYLGHVEVEESRGMH

VCEDAVKKLKGRKSVKSVLWVSADGLRVVDDKTKDLLVDQTIEKVSFCAPDRNLDKAFSY

ICRDGTTRRWICHCFLALKDSGERLSHAVGCAFAACLERKQRREKECGVTAAFDASRTSF

AREGSFRLSGGGRPAEREAPTPATTSPGEKGEAGTPAAIPRRHAPLEQLVRQGSFRGFPA

LSQKNSPFKRQLSLRLNELPSTLQRRTDFQVKGTVPEPAPAPELDPFEAQWAALEGKA

>Sko_259013

MKKLRRSL-----SKRETYVPECSKPHQWQEDEKLVKAGTCNFPVKYLGSVEVNESRGMP

ICEDAVRKLRDKKKVRAVLWVSSDGLRVVDEDSKGLIVDQTIEKVSFCAPDRRHERAFSY

ICRDGTTRRWLCHAFIAVRDSGERLSHAVGCAFAACLERKQKRDKECGVKVEFDVNKTSF

ARQGSFREPTMTEKLEQQKR--LPLEHVPPRAVNLPYAVPRRHATPNMLERQGSFRGFPG

LAK-DSPFKRQLSLRLNELPSTLARQKDLNILPEENGQQHQVHQSDFDAQWNALSNAQ

>Tca_189240

MDRLRRSFRDSFRRRKDRV-PESSKPHQWQADESAVRSGTCTFAVKYLGCVEVFESRGMQ

VCEEALKVLRRRRPVRAVLHVSGDGLRVVEDESKGLIVDQTIEKVSFCAPDRNHERGFSY

ICRDGTTRRWMCHGFLALRESGERLSHAVGCAFAVCLERKQKRDKECGVTMMFDTKNATF

TRSGSFRQQGLTERLERGV-DVGPPRPAPV----NPFAIERPHATPSMLQRQGSCRGFST

LGQ-NSPFKRQMSLRVNELPSNTARLNAYKSPTSPTNGAFQRTADPFDAEWADIAAKN

>Isc_021614

MDRLRRSLRDSLRRGRREHVPECSKPHQWQADEAAVRAGSCTFPVKYLGCVEVFESRGMQ

VMLLPLSELPRPIIFRNALIIRHVLVRFSDRPLSDFIVRQVFVRLGKAS---YYETSHRW

AC---LLRPIIRLIFISLRPQGERLSHAVGCAFAVCLERKQKRDKE-SVLMNFDPKTSSF

TRTGSFRQGSITDSLPQDPQVSCRAEAPPVKSVVNPYAIERPHATVSMLQRQGSFRGFGS

LSQ-TSPFKRQLSLRLNELPSNIERQRAMSLDASGDLPSR------------------

>Pdu_numb

MEKLKRSL--SFRKKKDHV-PESSKPHQWQEDERKVREGTCSFQVRYLGCIEVFESRGMQ

VCEEAVKALKKGKYQRAILYVSGDALRVVDEISKGLIVDQTIEKVSFCAPDRNHEKGFAY

ICRDGTTRRWMCHGFLALKESGERLSHAVGCAFAICLEKKQKREKDA-VQVQYNEKGTSF

SRTGSFRQATLTERLCDPQ-STILSESVPVKAVDNPHAVSRPHATADMLQRQSSFRGFTK

LGD-QSPFKRQLSLRLSDLPSTLQRQQDMNNPANGISAPQQQAFDPFDAAWAAKKVGV

>Cte_P22832

----------------------------------------------------------MT

VCEEACKALRKGKYQRAILYVSGEALRVVDEINKGLILDQTIEKVSFCAPDPHNLKGFSY

ICRDGTSRRWMCHGFMAVKDSGERLSHAVGVAFTVCLEKKQEREKEA-VSVAFNDKGTSF

TRTGSFRQATLTERLEDPQ-SAIVAEPVPQKQIFNPYAVQRPHAKDAMLERQGSFRGFTK

LAE-SSPFKRQLSLRLNELPSTLDRQKQVLNQQSSVDTPQATTFDPFDAAWAASGRPS

>Hro_P12629

---------------------QQSIPQLWLEDEVKVKNATCNYPVEYLGYTEVFESRGVV

VCEKAIRALKRRRCKKAVIHISGDAIRVVDEINKVLLVDQTLEKVSFCAPDRNLNEGFAY

ICRDASTKRWLCHGFLAIKDSGDRISHALGTAFAVCLERKVNREKL--------------

------------------------------------------------------------

LAS-------------------------------------------------------

>Hro_P63899

MDKFKRTL--SLKKKKESSGGESIKPHQWQEDERKVREGSCSFQVKYLGCIEVFESRGIQ

VCEDAVKALKNSGHHMAVLYVTGDALRVVDEISKGLIVDQTIEKVSFCAPDRNHEKGFAY

ICRDGTTRRWMCHGFLAVKESGERLSHAVGCAFAICLEKKQQREKE-KVTVTYNDNGTSF

SRLGSFRQATMTERLTDPQ-----------------------------------------

----------------------------------------------------------

>Aca_524887

MQSIRRRF--SLRKKKDHA-SESARVDQWEEDTNKVRNGTCSFQVRYLGYIEVFDSRGMH

ICEEAVKALKKGKYQRAVLYVSGDALRVVDEVSKSMIVDQTIEKVSFCAPDRHNEKAFAY

ICRDGTTRRWMCHGFLAVKESGERLSHAVGCAFAICLEKKQKRGSETGVTVTYNQSRTSF

ERTGSFRQTTLTERIVDPQ-SAILAEPVPVKSANNPYAVQRPHASANLLERQGSFRGFEK

LNEISSPFKRTTSLRLNELPSTLQRQNAVLESPTHNGSSQGPSVDPFDVAWAAKSVNQ

>Lgi_164463

MQSLKRRL--SFRKKKDHV-PECSKPHQWQEDEKKVREGTCSFQVRYLGCIEVFDSRGMQ

VCEEAVKALKKGKVQRAVLYVSGDALRVVDEISKSMIVDQTIEKVSFCAPDRNHEKGFAY

ICRDGTTRRWMCHGFMAVKESGERLSHAVGCAFAVCLEKKQKRDKETGVTVTFSEDRTSF

TRTGSFRQTSMTERLADPQ-SAILAEPVPVRRVDNPFAVQRPHATPELFNRQGSFRGFEK

LQEASSPFKRTLSLRYSDLPSTLQRQNAILESPSANGTHSKTGVDPFDVAWAAKSVNK

>Cgi_100039

MESLKRRL--SFRKKKNHV-PECSKPHQWQEDEKKVRDGTCSFQVRYLGCVEVFESRGMQ

VCEEAVKTLKKGKYQRAVLYVSGDALRVVDEISKSMIVDQTIEKVSFCAPDRNHEKGFAY

ICRDGTTRRWMCHGFLAVKESGERLSHAVGCAFAICLERKQKRDKDTGVEVTFSQDKTSF

TRMGSFRQTTLTERITDPQ-SAILAEPVPVRKVDNPFAVERPKATPSMLVRQGSFRGFEN

LQKDSSPFKRSVSLRLSDLPSTLQRQGAITESSPPKQKPTGPNVDPFDVAWAAKSANK

>Sme_2653b

MEKFKKSI--SFRKKKEKCQDEDPKPVSWQEDERKVREGCCSY-------IEVFDSRGMQ

VCEEALKALR----------------------------RPKLENVYF-------------

------------------------------------------------------------

------------------------------------------------------------

----------------------------------------------------------

>Sme_5843.0

MERFKRTF--TLRKKKPTSKASNTKPSQWNDDEKKVREGCCSFQVKYLGSIEVFESRGMP

VCEEAMKQLLKKKSKRAILYVSGDALRVTEEISKGLIVDQVIEKVSFCAPDRNHKRGFAY

ICRDATTRRWLCHGFKAVKES---------------------------------------

------------------------------------------------------------

----------------------------------------------------------

>Sme_2653a

---------------------------------------------------MSFSSPRIF

ICPICIHYLSQKKTIRAILYVSGDTIRVSDEISKGLIVDQTIEKVSFCSPDRNHKKGFAY

ICRDGTTRRWMCHAFLAVKET---------------------------------------

------------------------------------------------------------

----------------------------------------------------------

>Nve_241883

MKRLKRSL--SLRSSRRRI-PESHRPQVWENDSYKVRNGGVSFPVKYVGAIEVTESRGTQ

VCAEAFRKMRVHKKKRMNLLVTSDCIRVVDEETKSLTIDQTIEKVSFCTPDPSDDRVFSY

ICREGTTRRWMCHCFIAIRDTGERLSHAVGCAFTACLQRKQKA---QALQQQAKAAKEGE

KTDGTQATSTAPSTAANQQQSASSVVNGPTETSGMTPVVTTTQQVPPALKPPPAAARSRP

QPFAPSPFTRHMSLRYRSTPMSLTPLKDSGKAGYSTLGSTGMHQVREDDEFASLATRR

>Mle_00718a

MKKIKK----NIGQQKNGV-----SQQHYEADKDLVAKSGATFCVKYLGYEEVKEARGVK

VCSVAVAKLIKKNKKKMSLSISMDGVRVVDDISRVLVLDQPIEKISFCAPDSLNPKLFSY

IARDGPCRRWLCYGFYACDVPGERLSHALGCAFTACLQRKQKA---SKVTESNNSTGSVS

SKGSSIESSPATQPAKG-----------------------KPAATKPPITKANTTASITT

ITR---PPPKEITVPIPDFSRDRSATTIGISTPPSQQLPQFSHTNPFSAAVSSESSGQ

**Nrarp sequences**

>HOMSA|gi|51972284|ref|NP_001004354.1| notch-regulated ankyrin repeat-containing protein [Homo sapiens]

MSQAELSTCSAPQTQRIFQEAVRKGNTQELQSLLQNMTNCEFNVNSFGPEGQTALHQSVI

DGNLELVKLLVKFGADIRLANRDGWSALHIAAFGGHQDIVLYLITKAKYAASGR

>Skow_269784929|ref|NP_001161616.1

MTAQIQSPHQKVFHEAVKNGDTTELARVLETARQDVNVNMFDCEGQTALHQSVIDGNFEL

VKLLVQFGADVKLANRDGWNALHIAAYGGHEDIALYLITNVNSCKR

>IXOSC|ISCW009526-PA pep:novel scaffold:IscaW1:DS834657:5562:5876:1 gene:ISCW009526 transcript:ISCW009526-RA description:"notch-regulated ankyrin repeat protein, putative"

MSQTEIMSQEVFQRAVKKGDASELQRLLQRWEGSLNVNFYDHEGQTALHKSVMDGNLELV

KLLVKFGADTRLANRDGWSAIHIAAYGGHQDIALYLISNSGTRR

>Pdu_Nrarp

MTQELFQEAVGAGDTKQVHKLLTDSRLAKLLDLDSFDREGMTALHHFCLSGDLELVKLLV

KSGANARLANRDGWGPVHLAAYGGHSEIALFLMKATRRHAT

>CAPTE|CapteP156434 pep:novel supercontig:GCA_000328365.1:CAPTEscaffold_749:78648:79616:1 gene:CapteG156434 transcript:CapteT156434 description:""

MSLHSDVTTGAQRPSPRSSSSATSTSSSSYQRAFQDAVRNGDARQLLSILEERLEKVNIN

FFDKEGQTALHQSCLDGNLELVKTLVRYGADVRLANRDGWNPIHIAFQGGHTAIALYLVN

AHRR

>HELRO|HelroP187992 pep:novel supercontig:GCA_000326865.1:HELROscaffold_1:9773481:9775268:1 gene:HelroG187992 transcript:HelroT187992 description:""

MTPIYKTFSDALLMQDFPRIQSILTERHTFLDLNRYCRDGQTPLHHCSSSGNLKLVRLLV

EHGANPRRKNKEGWTPLHLSVFRGHRKITAYLLKILIGKREKVHHSITQDVKPSIFEVTC

KEEKIDFEEDDKKCLLQLQ

>CRAGI|EKC31563 pep:novel supercontig:GCA_000297895.1:scaffold962:983812:984159:-1 gene:CGI_10027675 transcript:EKC31563 description:"Notch-regulated ankyrin repeat-containing protein "

MNGTGVKCNQIQKYFLDVVRNGDCDELQKILESRDEKININLYDNEGQTALHQSCLIGSL

KKVQILVKFGADIKLANRDGWNALHIASFGGHQDIALYLISTKSRTKTMSTSSDS

>NEMVE|NEMVEDRAFT_v1g144286-PA pep:known scaffold:ASM20922v1:scaffold_988:11461:11661:-1 gene:NEMVEDRAFT_v1g144286 transcript:NEMVEDRAFT_v1g144286-RA description:""

NINTQNKAGDSPLHQCIKDGKLDSVKLLVDFGADITLPNRDGWSPMHVATFLGYRDMMLY

LLMNGR

**Nrarp alignment**

>HELRO|HelroP187992

PIYKTFSDALLMQDFPRIQSILTERHTFLDLNRYCRDGQTPLHHCSSSGNLKLVRLLVEH

GANPRRKNKEGWTPLHLSVFRGHRKITAYLL

>NEMVE|NEMVEDRAFT_v1g144286-PA

-----------------------------NINTQNKAGDSPLHQCIKDGKLDSVKLLVDF

GADITLPNRDGWSPMHVATFLGYRDMMLYLL

>Pdu_Nrarp

MTQELFQEAVGAGDTKQVHKLLTDSRLALDLDSFDREGMTALHHFCLSGDLELVKLLVKS

GANARLANRDGWGPVHLAAYGGHSEIALFLM

>CRAGI|EKC31563

QIQKYFLDVVRNGDCDELQKILESRDEKININLYDNEGQTALHQSCLIGSLKKVQILVKF

GADIKLANRDGWNALHIASFGGHQDIALYLI

>CAPTE|CapteP156434

SYQRAFQDAVRNGDARQLLSILEERLEKVNINFFDKEGQTALHQSCLDGNLELVKTLVRY

GADVRLANRDGWNPIHIAFQGGHTAIALYLV

>Skow_269784929|ref|NP_001161616.

PHQKVFHEAVKNGDTTELARVLETARQDVNVNMFDCEGQTALHQSVIDGNFELVKLLVQF

GADVKLANRDGWNALHIAAYGGHEDIALYLI

>HOMSA|gi|51972284|ref|NP_0010043

QTQRIFQEAVRKGNTQELQSLLQNMTNCFNVNSFGPEGQTALHQSVIDGNLELVKLLVKF

GADIRLANRDGWSALHIAAFGGHQDIVLYLI

>IXOSC|ISCW009526-PA

MSQEVFQRAVKKGDASELQRLLQRWEGSLNVNFYDHEGQTALHKSVMDGNLELVKLLVKF

GADTRLANRDGWSAIHIAAYGGHQDIALYLI

**Su(H) sequences**

>HOMSA|gi|34577081|ref|NP_055091.2| recombining binding protein suppressor of hairless-like protein isoform 1 [Homo sapiens]

MDPAGAADPSVPPNPLTHLSLQDRSEMQLQSEADRRSLPGTWTRSSPEHTTILRGGVRRC

LQQQCEQTVRILHAKVAQKSYGNEKRFFCPPPCVYLSGPGWRVKPGQDQAHQAGETGPTV

CGYMGLDSASGSATETQKLNFEQQPDSREFGCAKTLYISDADKRKHFRLVLRLVLRGGRE

LGTFHSRLIKVISKPSQKKQSLKNTDLCISSGSKVSLFNRLRSQTVSTRYLSVEDGAFVA

SARQWAAFTLHLADGHSAQGDFPPREGYVRYGSLVQLVCTVTGITLPPMIIRKVAKQCAL

LDVDEPISQLHKCAFQFPGSPPGGGGTYLCLATEKVVQFQASPCPKEANRALLNDSSCWT

IIGTESVEFSFSTSLACTLEPVTPVPLISTLELSGGGDVATLELHGENFHAGLKVWFGDV

EAETMYRSPRSLVCVVPDVAAFCSDWRWLRAPITIPMSLVRADGLFYPSAFSFTYTPEYS

VRPGHPGVPEPATDADALLESIHQEFTRTNFHLFIQT

>HOMSA|gi|42560227|ref|NP_005340.2| recombining binding protein suppressor of hairless isoform 1 [Homo sapiens]

MDHTEGSPAEEPPAHAPSPGKFGERPPPKRLTREAMRNYLKERGDQTVLILHAKVAQKSY

GNEKRFFCPPPCVYLMGSGWKKKKEQMERDGCSEQESQPCAFIGIGNSDQEMQQLNLEGK

NYCTAKTLYISDSDKRKHFMLSVKMFYGNSDDIGVFLSKRIKVISKPSKKKQSLKNADLC

IASGTKVALFNRLRSQTVSTRYLHVEGGNFHASSQQWGAFFIHLLDDDESEGEEFTVRDG

YIHYGQTVKLVCSVTGMALPRLIIRKVDKQTALLDADDPVSQLHKCAFYLKDTERMYLCL

SQERIIQFQATPCPKEPNKEMINDGASWTIISTDKAEYTFYEGMGPVLAPVTPVPVVESL

QLNGGGDVAMLELTGQNFTPNLRVWFGDVEAETMYRCGESMLCVVPDISAFREGWRWVRQ

PVQVPVTLVRNDGIIYSTSLTFTYTPEPGPRPHCSAAGAILRANSSQVPPNESNTNSEGS

YTNASTNSTSVTSSTATVVS

>Skow_259013480|ref|NP_001158483.1

MATTSPLSINTMATSVHTPPSPVPTPSPPTGAISPPRPDENGLIHTRHHGDKRLSKDAMR

HYLRDRQDMTLVILHAKVAQKSYGNEKRFFCPPPCIYLLGPGWKAKKEQMEKDGENDAGS

QVCAFMGIGNSDQDMQQLNLEGKNYCAAKTLYISDSDKRKHFFLSVKMFYGNGEDIGVFN

SKRIKVISKPSKKKQSLKNADLCIQSGSTVALFNRLRSQTVSTRYLHVENGNFHASSQQW

GAFNIHLLDDDESESEEFTVRDGYIHYGSTVKLVCSVTGMALPRLIIRKVDKQTALLDAD

DPVSQLHKCAFYMKDTERMYLCLSQERIIQFQATPCPKEPVKEMINDGASWTIISTDKAE

YTFYEGMGPVSSPLTPVPVVHSLQLNGGGDVAMLELTGDNFFPNLRVWFGDVEAETMYRC

AESIICVVPDISAFRGGWRWVRQPTQVPVSLVRNDGIIYPTGLTFTYTPEPGPRQHCTAT

ESILRGGHLSSPSSDTLHYISPMH

>Pdu_SuH

MQTPPSPLPTPSPPHRPGDIENYHHRSINSQYPGQRLTREGMRNYLRDRGDQVLVILHAK

VAQKSYGNEKRFFCPPPCIYLFGNGWKRKREQMERDGASDQESTVCAFMGIGNSDQEMVQ

LNLEGKHYCAAKTLYISDSDKRKHFMLTVKMFYGNGQDIGVFNSKRIKVISKPSKKKQSL

KNADLCIASGTKVALFNRLRSQTVSTRYLHVEGGNFHASSTQWGAFTIHLLDDDEGESEE

FTVRXGYIHYGMTVKLVCSVTGMALPRLIVRKVDKQTVLLDADEPVSQLHKCAFYMKDTE

RMYLCLSQERIIQFQATPCPKEPNKEMINDGAAWTIISTDKAEYTFFEGMGPVKAPVTPV

PVVSSLQLNGGGDVAMLELSGENFMPSLKVWFGDVEAETMFRCEVSMLCVVPDISAFRSS

WRWVRQPLQVPVTLVRNDGIIYATGLTFTYTPEPGPNHQSAAAACVMGHPTPNDHQDSQH

ALETLT

>CAPTE|CapteP179039 pep:novel supercontig:GCA_000328365.1:CAPTEscaffold_459:58445:61224:1 gene:CapteG179039 transcript:CapteT179039 description:""

MREYLKDRHDQILVVLHAKVAQKSYGNEKRFFCPPPCIYLFGSGWKRKKEAMEREGVTGQ

ESDVCAFMGIGNSEQDMQQLQLDGRNYCAAKTLYISDSDKRKHFMLSVKLFYGNGDEIGL

FNSQRIKVISKPSKKKQSLKNADLCIASGSKVALFNRLRSQTVSTRYLHVENGNFHASST

QWGAFTIHLLDDNEGESEEFTVRDGYVHYGSTVKLVCAVTGMALPRLVMRKVDKQTVLLD

ADEPVSQLHKCAFYLKDTDRMYLCLSQEKIIQFQATACPKESNKQMINDGASWTIISTDK

AEYTFYEGMGPVKCSVTPVPVVNSLHLNGGGDVAMLELAGENFSPSLKVWFGEVEAETMY

RCAESMLCVVPDISAFRSGWKWVRQPLQVPVSLVRHDGVIYATGLTFTYTPEPGPRPSRN

PAQDLMCGDPAKGPPPALPPGGHMGGMSMHSMHTPL

>HELRO|HelroP186992 pep:novel supercontig:GCA_000326865.1:HELROscaffold_22:2788619:2793315:1 gene:HelroG186992 transcript:HelroT186992 description:""

MAEYLRCRTDKHLKILHAKVAQKSYGSEKRFFCPPPCIYLSGQGWERPLKPSTDDVQVCA

YMGIGNSDQDMVPLNLDGKDYCAAKTLFISDSDKRKHFMLSVRMFYSNAQPIGVFQSKRI

KVISKPSKKKQSIKNADLCIGSGSKVSLFNRLRSQTVSTRYLHVEKGNFHASATQWGAFT

VHLLDDDESESEEFSVKDGFIHYGSTIKLVCVETGMALPRLVIRKVDKQTVILDTDEPVS

QLHKCAFYLKDTDHMYLCLAHEKIIQFQATPCPKEPNKEMINDGALWTIISTDCAEYAFY

EGMGPVAHPITPVPTVQSLQLNGGGDVAMLELTGENFTANLKVWFGEVEAETMHRCCEIM

LCVVPDISAFRSQWKYVREPLQVPVMLVRNDGIIYATGLTFTYTPEPGENPSPTPSDNNN

NNNNTNNNNNRLTFHAV

>HELRO|HelroP190647 pep:novel supercontig:GCA_000326865.1:HELROscaffold_14:1178087:1181747:-1 gene:HelroG190647 transcript:HelroT190647 description:""

MIENSQCSANLKELVRKYLQKPLHQTVLILHAKVAQKSYGSEKRFFCPPPCLYLSGDGWK

CNKEGAEDNGDRSEPTNHICAYMGIGTSSKEMVHMPLEARNFCAAKTLYISDSDKRKHFR

LCVNLFYSDGQSIGSFEGNNIKVISKPSKKKQSLKNADLCIESGTKIALFNRLRSQTVST

RYLFVDDGNFHASSMHWGAFEINLVDDNASESNEFTMESGFIHYGMTVKLVCVVTGIALP

LMVVHKVDKQTILLDAEEPVSQLHKCAFRFKNPHSTERMYLCLSQEKIIQYLAAPCPKDP

TKEMISDGASWTIISTESVRYSFFDGYSLPNIPVTPVPEIGIISVNGTSKMGFLEIKGRN

FVPTMQVWFGDVPAETTYRSDEIILCIVPDVNLFATTPAYVFISDHDPDPDPDSSNNDNN

NNTNNINNNNFNDNNSNNNNNNSNNNNSRANGRSLKKLEV

>Aca_524911596|ref|XP_005110659.1

MTPKYCAAKTLYISDSDKRKHFMLTVKMFYGNGQDIGVFQSKRIKVISKPSKKKQSLKNA

DLCIASGTKVALFNRLRSQTVSTRYLHVENGNFHASSTQWGAFTIHLLDDDEGESEEFTV

KDGYIHYGSTVKLVCSVTGMALPRLIIRKVDKQTSLLDADDPVSQLHKCAFYLKDTERMY

LCLSQERIIQFQATPCPKEAKREMINDGASWTIISTDKAEYTFYEGMGPVNKPVTPVPVV

NSLNLNGGGDVAMLELNSENFTAALKVWFGEVEAETMYRCDDSMLCVVPDISAFRTGGGR

WVRQPLQVPVSLVRCDGVIYSTGLIFTYTPEPGPQQHCREVDRILGRAPASPDSTSNTLH

AAL

>Lotgi1|115176|e_gw1.20.102.1

REAMRKYLRDRSDQILVILHAKVAQKSYGNEKRFFCPPPCIYLFGKGWKRKHDQMEEEGS

TKDEAQVCAFMGIGNSDQEMVQLHLEDKDYCAAKTLYISDSDKRKHFMLSVKMFYGNGQD

IGLFLGKRIKVISKPSKKKQSLKNAELCIASGTKVALFNRLRSQTVSTRYLHVENYNGKC

NFHASSTQWGAFTIHLLDDNEGESEEFTVRDGYIHYGSTVKLVCSVTGMALPRLVIRKVD

KQTALLDADDPVSQLHKCAFFLKDTERMYLCLSQERIIQFQATPCPKEPNKEMINDGASW

TIISTDKAEYTFYEGMGPVKNSLTPVPVVNSLHLNGGGDVAMLELNGENFNPSLKVWFGD

VEAETMFRAEDSMLCVVPDIAAFRPGWKWVRQPLQAPVSLVRLDGVIYATGLTFTYTPEP

GPRQHCKDMDRIVGRTSTASPDSTSHTTL

>CRAGI|oyster|CGI_10018383

MNEQHLYVSQNGYTYSVGPTQQSSHLMSCSQPQQNGSHTGHGFMHHNAGYTLHPPDRQYG

GRADPGPSSRADLTGHMAHGGYENPMDLSSNKPGNPGRLVKEEGHHHGYLAAMGTTPVSV

GIPDHVHNPSHIVAGSLTPPEKINGDPGAMATSSPLSITTMTQAIPAPPSPISTPSPLYA

SNSYVDRGYQDQRLTKEAMRNYLKDRGDQVLVILHAKVAQKSYGNEKRFFCPPPCIYLFG

SGWKRKKEAIEAEGGTEQDSTTCAFMGIGNSDQEMVQLNLEGKNYCAAKTLYISDSDKRK

HFMLTVKMFFGNGQDIGVFNSKRIKVISKPSKKKQSLKNADLCIASGTKVALFNRLRSQT

VSTRYLHVENGNFHASSTQWGAFTIHLLDDNESESEEFTVRDGYIHYGSTIKLVCSVTGM

ALPRLIIRKVDKQTAILDADDPVSQLHKCAFYMKDSERMYLCLSQERIIQFQATPCPKEP

NKEMINDGASWTIISTDKAEYTFFEGMGPVKSPVSPVPVVNSLHLNGGGDVAMLELSGEF

LAPNLKVWFGEVEAETMFRCEESMFCVVPDISAFRAGWRWVRQPLQVPVLLVRSDGIIYS

TGLTFTYTPEPGPRAHSREVDRIIQPGVSSPDSTSTANFSNPL

>Smed_mk4.000084.19.01

MNNNLNGYNSMTYLGNISNDLMLPNQSDFMSCTDQSLFQYSNPINKGFDSNWSYNPSNGL

LVDRKRITMNDDYSFAPRTKIMHTTTHHPDFESYKLSVESFPQKSNVTNHNNDYHNNIST

NHTTSANCCTFNYKYSDHLDRTFSVNKEPTFEPLDLRSPIGSTDLKMNDLLNDNSDSNHI

NPFKLNNSSQEQDPNLTQNGGIPYTAALPLSHHTNVNNNNNSNNNNINNNNNNNNNNNNN

NNTHQVKPSIDLITFILISSPENNNTIRRLTKEVMRKYLQDPRDHILIILHAKVAQKSYG

NEKRFFCPPPCVYLHGKGWEDDQNRLSEQSKSPESRLGTDSTQLKAFMGIGSTNPEQEMV

ELNLDSHKDYSAAKTLYISDSDKRKHFMLHVKIFHPNGQDVGQFYSKRIKVISKPSKKKQ

SLKNTDLCIASGTKVALFNRLRSQTVSTRYLHVEDKNFHASSTKWGAFSMHLLADDEVEA

EEFSVQEGYIHYGHTVKLVCSVTGMALPRLIVRKVDKTTVILDADDPISQLHKCAFYLKD

TDRMYLCLSQDKIIQYQAVSRPDHENGGNSMREAINDSACWTIISTDKAEYRWFQALGPA

MGPVSPVPLVSDLKLNGGGDVAMVELNGENFSTNLEVWFGDVPAQTYYRCSNVLLCVVPD

INEFRTAWDHVQEPVEVPVNLVRSDGVVYSTGMTFTYRPEPAPRKKCVEAIQIICNGVPN

SDNITGPTSGSDAACSINS

>Tca_91083357|ref|XP_975102.1

MPHQYGLPGIAHAQSPPTPPPQHGAMYSRFSGAVVPGGYRGEDRRLTREAMERYLRDRSD

MVIVILHAKVAQKSYGNEKRFFCPPPCIYLFGDGWRLRQEQMLREGESEQASQLCAFIGI

GNSDQDMQQLDLNNGKQYCAAKTLYISDSDKRKHFMLSVKMFYGSGHDIGVFHSKRIKVI

SKPSKKKQSLKNADLCIASGTKVALFNRLRSQTVSTRYLHVENGHFHASSTQWGAFTIHL

LDDNESESEEFQVRDGYIHYGSTVKLVCSVTGMALPRLIIRKVDKQQALLEADDPVSQLH

KCAFYMKDTERMYLCLSQERIIQFQATPCPKEPNKEMINDGACWTIISTDKAEYQFYEGM

GPVRSPVTPVPIVHSLHLNGGGEVAMLELTGDNFSPSLQVWFGDVEAETMYRCQESMLCV

VPDISQFRGEWLWVRQPTQVPVSLVRNDGIIYATGLTFTYTPEPGPRPQCLPADEIMRHG

QRSAAAAQGQSQALTQEAAWNSHGGMH

>IXOSC|ISCW010197-PA pep:known scaffold:IscaW1:DS829427:102938:106662:-1 gene:ISCW010197 transcript:ISCW010197-RA description:"recombining binding protein suppressor of hairless, putative"

MSDVYLPDDQYAGYHPVQNNYSSPHDDGTFAVSGSAYSGDYGRDLLLDLGQQAAGAPVDM

SSHPARAHPYFNSGGVPFKNGLADGEPGALLGSAAKGAGEGPPQGSPHLGVQAPRLPPSP

LPSPPSEERYRRGEPRLTRDAMDRYLRDRGDMVLVILHAKVAQKSYGNEKRFFCPPPCVY

LLGDGWQRKRDQLLRDGEADQAAQLCAFIGIGNSDQDMQQLDFAGKSYCAAKTLFISDSD

KRKHFMLSVKLFYGNGEDVGVFQSKRIKVISKPSKKKQSLKNADLCIASGTRVALFNRLR

SQTVSTRYLHVDGGNFHASSSQWGAFTIHLLDDNESEAEEFTVRDGYIHYGSTVKLVCSV

TGMALPRLVIRKVDKQNAFLDADDPVSQLHKCAFYMKDTERMYLCLSQEKIIQFQATPCP

KEPNREMINDGASWTIISTDKAEYTFHEGAGPVRVPVTPVPVVNSLHLNGGGDIAMLELT

GENFAPNLRVWFGNVEAETMYRCAECLLCVVPDISAFREGWQWVRQPTQVPVSLVRSDGV

IYATGLTFTYTPEPGPRQPGPYPALVHDILRPANARSHPPAPEDHGAPTANFGHHMHFGS

HHQNMS

>NEMVE|jgi|Nemve1|166232|estExt_gwp.C_710036

MIRSANLVASLSSSSRKSDRIHIRDDTLEEISGFHPYNGPMRRAASSLTSPQPPIKRFHF

DSYSPNELAPSNGSLDSALYQAQISSNVQGNYVPVSESHRDRRDPYHRVGVYSSTPKHGH

DALASPETRGLAPINMTAGQPSPPTTPSPLPDDEECLHNESSRIPFCPYIPTKLTKAAMD

RYLTDVVHRNGLQTVVIFHAKVAQKSYGNEKRFFCPPPCVYLFGEGWRNRQRLLQQAGED

DGNNQQPVAFIGIGNSSEQEMQQLIIEEKGYGAAKTLFISDSDKRKHFELSVKLLYPNGD

HVGIFNSKRIKVISKPSKKKQSLKNADLCIQSGSTVALFNRLRSQTVSTRYLHVENGNFH

ASSQQWGAFTIHLLDDEESESEEFTVREGYIHYGSTVKLVCSVTGMALPRLIVRKVDKQT

SLLDADDPVSQLHKVAFYMKDTERMYLCLSQERIIQFQATPCPKESNKEMINDGASWTII

STDKAECNFYEGMGPVRSPVTPVPFVSHLQLNGGGDVAMLELTGENFTANLKVWFGDVEA

ETMYRCGESLLCVVPDISAIRGGWRFVRQPTEVAVSLVRCDGVIYPTGLTFTYTPEPVER

PALSVADSVLRGEL

>MNELE|comb jellyfish [Mnemiopsis leidyi] ML141212a

MEAEDTPPMVTEIKKEADEYWVTVVKAMLHCQQVMQQQQQQQQQQQRQQQQQQQQQQQQQ

QQQHQHRLARHCLTDFSQLGYISPKSEMFSHTEPVMSTLFTDRLRSHTPPASLYKPNIII

PQYDHLIRQASPPISPPQPETSAAVAPVEFTKEIMNKYLANRKRLDKEVVIRHARVAQKS

YKNEKRFFCPPPCVYLRGTAWGVGEHRRGILEQSGLTPNAFIGLSRNHDDMQQLHLEEKG

FAAAKTLYISDSVDKRKQFDLYLKMLYSNGQNIGTLKSKRIKVISKPSKKKLSIKNIALS

IQSGTHIALFNRLRSQTVSTRFLHVEDENFHASSHQWGSFAIHLVDDDEEDSEVFNVQPG

HVHYGKTVKIVCSETKVSLPKVVIRKVEKQVAFLDAEDPVSQLHKVAFYLKDTERMYLCL

SQDKIIQFQATPCPTDPKKEMINDGACWTVISTDEAKYRFCDALTTVKKPIAPVPVVEQM

TAEGYGNGSGESYLEISGEGFSPNLKVWLDNHECETVYRCAETMLAIIPPIQVFKPNWTY

VQQPVTCLLVLVRTDGVVYSTDKEYIYEVQSPPSPESPQDFQASTSQSTS

>Monbr1|25160|fgenesh2_pg.scaffold_9000094

MSAPDAPVSSVSSFLFSLFALSLHYSLSLSLTSLFCLTTGHDSIKERERERERERERGVS

GDDKRWLPFAACLLPGGGEGGKRCFVASVLRRDLIWWGRFNFVTMAKRSTASDYHVPATS

SNYFDPGAPLAKLPKPGDHSMYQDPSMYAPPTMTGVSAVDTLDFASNNPIVSQALPSLAD

GNFASLLDRGDVSGYPPTLASQMSMGTSASAQMYSDLPPTSDTSYNMATTTMSHVTSMQS

MASSMPHVSSMQSMASSMPHVSSMQSVASSMQPMVSSMQPMVSSMQHVASNMQPMVSNMQ

PLVSDISSTISTMPHYSQAASVSQAAYFGNTDLTTHGQDDISQPSATPGSSNVVDIKSCV

KAYLESTHTGAQDMKLTILHPRVVQKSYGNEKRYFCPPPQIHLSGRNWNHPQLGEPRVFV

SMHLNYEHTHQTEIPLSRLADSEFHGTASQLFIPDTDKRKSFCLQLKVFFQNDESNEVRD

VGTFPSKPLRVISKPSKKKQSNKSHSDMYMESGCEALFFHRSRSTNAATYYFRLNEEGQP

DCSDEGWHALKLVNDHTNVRETKLDRFLPAPHLPVPNPPPPSLLAPLTLTCTSQHNEANL

ASRNDRYIVYDRQVRMKCAQTNKEMVCTIHRCDRNAVVPVDNECVSQLQKVAISRVNPET

KETEYLKFQSSNGSASVFFERARSKADKGSGVQLSEQSIWTVCTGARLEYSFFDHTIDRF

VQHIEGEMVKPITPVPLVDEVKSLGPMVELYGEHFSTMLTVFFGPAPACTYYRCEELLFC

KPPSYAEAVESSEAVCTASYTVPLLLVRNDGVIYNTFKTYKYQPQPNGSMRAASNASEST

FSLSLSLKLSNSLKLSLKLSLSLKLSLSLSNSLSQTLSLSIRLSLSNSLKLSLSLFRWRE

SQCCLRLYDKSRFCLAVSAVLNDTNFDEIERVYIKSLESRFDYTCTRKNNMYCAGVYSEE

RFYSLLPRVQNLSLSVAARESLLGNMCTPCAYKFLSLMAAESELPTEYMHLEALCVRDEE

RFCYPRLANVLALSDENTSLARFGQALCHETEMGRCASKMLVRQQLYPAAVAFSMPVPDK

DLVYQEQACIRSTDQELCSKVAEDVAGLMTVGLQATGTNTGPDMCDRVPADDRCTWGCQR

RWTPVRNEYGCCYATYRQFAHDLGASSSDLATTFGHLDEVAAKCNRSFVDACPAIAKSTA

QQLSLVLTDIDADYARHNKSVILKAVGPDVQRILGLSADAFNITDLTTLDGVATLIITVY

GDHAATVTQLEAALQADITNNAVIFMNLQKVYESAKRQRGRQPCGLAMEAESEVRARLER

ANAPCIALLKPKRENQQGQYMSLDSPDVVKAEPQRGLYPCLDDSKIQALISGTPCDCPGF

QLKASPADLRWPLIVKCICARLQQLQCSHCQHDLSAHPGSDDVQDETAVQLRRLRAFYMP

TGHARVNLSPADAIVSLRAHPDPAGQQVSLCVFDGGIKTLTRYTHAFKAGSTVSNLTDAD

GRSKGRPVIPASSAPSVTQLKSEGSTLPPASVDLSVPTSTPKTVRDPLAVSTKKPRQLTL

FGTRATKACPLPSVTNDATATGADPEPETKKLPDLPSDPVAPHQRIDAMFKRTSETASGG

RNASKTKTKRSKVTPTPNITAPTDSKPGAICFAVELPKTKPPPPTAPPPPMLSTTSSSTH

TNQENVPSGTGLQLRGSKPSVKPPPPAMQSPPPAPPKSTQQTLLNFGRPSVKSKPKNEAH

RNAVTCPSSQSLAEELVQSKNSCTFSFSRRASKTLRFLAAPDGACFSWRATSNDLVRLGA

NGDGNIFVTGNTELPDTIIDVTFGGAALSLLLVLTARGNIEAAVIPPHPHSDVERQSLAV

VPGIATPTLTANEDWLAVGTFEDGPVQRLKPHYFFSSTSQ

>Aqu1.226412_PAC:15724940

MSLVPAVYSSNGALLPIPQMTTVTSGGYIGVDGHGLQLIGQPLVTAPSQQIHLLQQQQHP

SSSSSPSSNDKISNQPLSEKYELKLNKLTREAMRQYLKDREDCTVVVLHAKVAQKSYGNE

KRFFCPPPSLYLMGKGWKSRKKENEKKKAEGQRNGSTSSTSSSSQANSSSVGVDAFDKPC

CFVGIGNHDQEMQHLSLEEKNFCIAKTLYISDSDKRKHFQLQCKVYLGDGRDLGTFLSQR

IKVISKPSKKKQSLKNPELCIPSGSSVALFNRLRSQTVSTRYLHVEGNNFHASSQQWGSF

SIHLVDEDAAESEEFTVQEGYIKYGSTVKLVCSTSGMALPRLVIRKVDKQTVLLDADDPV

SQLHKVAFYMKDTERMYLCLSQDRIIQFQATQCPGEPHREMINDGASWTIISTDKAEYTF

SEGMGPVTAPVTPIPIAKDIRLIGGDDLQMLEIQGEGFTPDLRVWFADVEAETMYRCAEC

LMAVVPPIDQFQKDSKKDDSIKVPLLIVRIDGIIYNTHMKFQYISSDVPLPPILEPRHIP

ALPPPLMDHMNMIPNHTPYSIGGPNCTTSSLTLLQRPHLLSTDPTSSHLGVQPGFIHHSP

YSNGGGAEMQLHSYRGSIVFLTASFYTFDIKKSPGRCFS

**Su(H) alignment**

>Monbr1|251

IKSCVKAYLESAQDMKLTILHPRVVQKSYGNEKRYFCPPPQIHLSGRNWNHPQLG-----

--PRVFVSMEHTQTEIPLSRLDSEFHGTASQLFIPDTDKRKSFCLQLKVFFQNDEDVGTF

PSKPLRVISKPSKKKQSNKSHDMYMESGCEALFFHRSRSTNAATYYFRLNEGQPDCSDEG

WHAL--KLVNDHTNETDRFLPNDRYIVYDRQVRMKCAQTNKEM-VCTIHRCDRNAVVPVD

NECVSQLQKVAISRVNKETEYLKFQSSNSVFFERARSKADKGGVQLSEQSIWTVCTGARL

EYSFFDHTGEMVKPITPVPLVDEVKSLG----PMVELYGEHFSTMLTVFFGPAPACTYYR

CEELLFCKPPSYAEAVESEAVCTASYTVPLLLVRNDGVIYNTFKTYKYQPQPN

>HelroP1906

LKELVRKYLQKPLHQTVLILHAKVAQKSYGSEKRFFCPPPCLYLSGDGWKCNKEGAEDNG

DRICAYMGIGTSSKEMVHMPLEARNFCAAKTLYISDSDKRKHFRLCVNLFYSDGQSIGSF

EGNNIKVISKPSKKKQSLKNADLCIESGTKIALFNRLRSQTVSTRYLFVDDGNFHASSMH

WGAFEINLVDDNASESNEFTMESGFIHYGMTVKLVCVVTGIALPLMVVHKVDKQTILLDA

EEPVSQLHKCAFRFKNTERMYLCLSQEKIIQYLAAPCPKDPTKEMISDGASWTIISTESV

RYSFFDGYSLPNIPVTPVPEIGIISVNGTSKMGFLEIKGRNFVPTMQVWFGDVPAETTYR

SDEIILCIVPDVNLFAT-----------------------TPAYVFISDHDPD

>HelroP1869

----MAEYLRCRTDKHLKILHAKVAQKSYGSEKRFFCPPPCIYLSGQGWERPLKPSTDDV

--VCAYMGIGNSDQDMVPLNLDGKDYCAAKTLFISDSDKRKHFMLSVRMFYSNAQPIGVF

QSKRIKVISKPSKKKQSIKNADLCIGSGSKVSLFNRLRSQTVSTRYLHVEKGNFHASATQ

WGAFTVHLLDDDESESEEFSVKDGFIHYGSTIKLVCVETGMALPRLVIRKVDKQTVILDT

DEPVSQLHKCAFYLKDTDHMYLCLAHEKIIQFQATPCPKEPNKEMINDGALWTIISTDCA

EYAFYEGMGPVAHPITPVPTVQSLQLNGGGDVAMLELTGENFTANLKVWFGEVEAETMHR

CCEIMLCVVPDISAFRSQWKYVREPLQVPVMLVRNDGIIYATGLTFTYTPEPG

>Hsa3457708

LRGGVRRCLQQQCEQTVRILHAKVAQKSYGNEKRFFCPPPCVYLSGPGWKPGQDQAHQAG

ETVCGYMGLGS-ATETQKLNFDSREFGCAKTLYISDADKRKHFRLVLRLVLRGGRELGTF

HSRLIKVISKPSQKKQSLKNTDLCISSGSKVSLFNRLRSQTVSTRYLSVEDGAFVASARQ

WAAFTLHLADGHSAQGD-FPPREGYVRYGSLVQLVCTVTGITLPPMIIRKVAKQCALLDV

DEPISQLHKCAFQFPGSPPTYLCLATEKVVQFQASPCPKEANRALLNDSSCWTIIGTESV

EFSFSTSLACTLEPVTPVPLISTLELSGGGDVATLELHGENFHAGLKVWFGDVEAETMYR

SPRSLVCVVPDVAAFCSDWRWLRAPITIPMSLVRADGLFYPSAFSFTYTPEYS

>Hsa4256022

TREAMRNYLKERGDQTVLILHAKVAQKSYGNEKRFFCPPPCVYLMGSGWKKKKEQMERDG

CSPCAFIGIGNSDQEMQQLNLEGKNYCTAKTLYISDSDKRKHFMLSVKMFYGNSDDIGVF

LSKRIKVISKPSKKKQSLKNADLCIASGTKVALFNRLRSQTVSTRYLHVEGGNFHASSQQ

WGAFFIHLLDDDESEGEEFTVRDGYIHYGQTVKLVCSVTGMALPRLIIRKVDKQTALLDA

DDPVSQLHKCAFYLKDTERMYLCLSQERIIQFQATPCPKEPNKEMINDGASWTIISTDKA

EYTFYEGMGPVLAPVTPVPVVESLQLNGGGDVAMLELTGQNFTPNLRVWFGDVEAETMYR

CGESMLCVVPDISAFREGWRWVRQPVQVPVTLVRNDGIIYSTSLTFTYTPEPG

>MNELE|comb

TKEIMNKYLANRLDKEVVIRHARVAQKSYKNEKRFFCPPPCVYLRGTAWEHRRGILEQSG

LTPNAFIGLSRNHDDMQQLHLEEKGFAAAKTLYISDSDKRKQFDLYLKMLYSNGQNIGTL

KSKRIKVISKPSKKKLSIKNIALSIQSGTHIALFNRLRSQTVSTRFLHVEDENFHASSHQ

WGSFAIHLVDDDEEDSEVFNVQPGHVHYGKTVKIVCSETKVSLPKVVIRKVEKQVAFLDA

EDPVSQLHKVAFYLKDTERMYLCLSQDKIIQFQATPCPTDPKKEMINDGACWTVISTDEA

KYRFCDALTTVKKPIAPVPVVEQMTAEGGSGESYLEISGEGFSPNLKVWLDNHECETVYR

CAETMLAIIPPIQVFKPNWTYVQQPVTCLLVLVRTDGVVYSTDKEYIYEVQSP

>Aqu1.22641

TREAMRQYLKDREDCTVVVLHAKVAQKSYGNEKRFFCPPPSLYLMGKGWKKENEKKKAEG

QRPCCFVGIGNHDQEMQHLSLEEKNFCIAKTLYISDSDKRKHFQLQCKVYLGDGRDLGTF

LSQRIKVISKPSKKKQSLKNPELCIPSGSSVALFNRLRSQTVSTRYLHVEGNNFHASSQQ

WGSFSIHLVDEDAAESEEFTVQEGYIKYGSTVKLVCSTSGMALPRLVIRKVDKQTVLLDA

DDPVSQLHKVAFYMKDTERMYLCLSQDRIIQFQATQCPGEPHREMINDGASWTIISTDKA

EYTFSEGMGPVTAPVTPIPIAKDIRLIGGDDLQMLEIQGEGFTPDLRVWFADVEAETMYR

CAECLMAVVPPIDQFQKSKK--DDSIKVPLLIVRIDGIIYNTHMKFQYISSDV

>Smed_mk4.0

TKEVMRKYLQDPRDHILIILHAKVAQKSYGNEKRFFCPPPCVYLHGKGWEDDQNRLSEQS

KSLKAFMGIGSNEQEMVELNLSHKDYSAAKTLYISDSDKRKHFMLHVKIFHPNGQDVGQF

YSKRIKVISKPSKKKQSLKNTDLCIASGTKVALFNRLRSQTVSTRYLHVEDKNFHASSTK

WGAFSMHLLADDEVEAEEFSVQEGYIHYGHTVKLVCSVTGMALPRLIVRKVDKTTVILDA

DDPISQLHKCAFYLKDTDRMYLCLSQDKIIQYQAVSRPDHENREAINDSACWTIISTDKA

EYRWFQALGPAMGPVSPVPLVSDLKLNGGGDVAMVELNGENFSTNLEVWFGDVPAQTYYR

CSNVLLCVVPDINEFRTAWDHVQEPVEVPVNLVRSDGVVYSTGMTFTYRPEPA

>IXOSC|ISCW

TRDAMDRYLRDRGDMVLVILHAKVAQKSYGNEKRFFCPPPCVYLLGDGWQRKRDQLLRDG

EALCAFIGIGNSDQDMQQLDFAGKSYCAAKTLFISDSDKRKHFMLSVKLFYGNGEDVGVF

QSKRIKVISKPSKKKQSLKNADLCIASGTRVALFNRLRSQTVSTRYLHVDGGNFHASSSQ

WGAFTIHLLDDNESEAEEFTVRDGYIHYGSTVKLVCSVTGMALPRLVIRKVDKQNAFLDA

DDPVSQLHKCAFYMKDTERMYLCLSQEKIIQFQATPCPKEPNREMINDGASWTIISTDKA

EYTFHEGAGPVRVPVTPVPVVNSLHLNGGGDIAMLELTGENFAPNLRVWFGNVEAETMYR

CAECLLCVVPDISAFREGWQWVRQPTQVPVSLVRSDGVIYATGLTFTYTPEPG

>Tca_910833

TREAMERYLRDRSDMVIVILHAKVAQKSYGNEKRFFCPPPCIYLFGDGWRLRQEQMLREG

ESLCAFIGIGNSDQDMQQLDLNGKQYCAAKTLYISDSDKRKHFMLSVKMFYGSGHDIGVF

HSKRIKVISKPSKKKQSLKNADLCIASGTKVALFNRLRSQTVSTRYLHVENGHFHASSTQ

WGAFTIHLLDDNESESEEFQVRDGYIHYGSTVKLVCSVTGMALPRLIIRKVDKQQALLEA

DDPVSQLHKCAFYMKDTERMYLCLSQERIIQFQATPCPKEPNKEMINDGACWTIISTDKA

EYQFYEGMGPVRSPVTPVPIVHSLHLNGGGEVAMLELTGDNFSPSLQVWFGDVEAETMYR

CQESMLCVVPDISQFRGEWLWVRQPTQVPVSLVRNDGIIYATGLTFTYTPEPG

>NEMVE|jgi|

TKAAMDRYLTDNGLQTVVIFHAKVAQKSYGNEKRFFCPPPCVYLFGEGWRNRQRLLQQAG

EDPVAFIGIGNSEQEMQQLIIEEKGYGAAKTLFISDSDKRKHFELSVKLLYPNGDHVGIF

NSKRIKVISKPSKKKQSLKNADLCIQSGSTVALFNRLRSQTVSTRYLHVENGNFHASSQQ

WGAFTIHLLDDEESESEEFTVREGYIHYGSTVKLVCSVTGMALPRLIVRKVDKQTSLLDA

DDPVSQLHKVAFYMKDTERMYLCLSQERIIQFQATPCPKESNKEMINDGASWTIISTDKA

ECNFYEGMGPVRSPVTPVPFVSHLQLNGGGDVAMLELTGENFTANLKVWFGDVEAETMYR

CGESLLCVVPDISAIRGGWRFVRQPTEVAVSLVRCDGVIYPTGLTFTYTPEPV

>Skow_25901

SKDAMRHYLRDRQDMTLVILHAKVAQKSYGNEKRFFCPPPCIYLLGPGWKAKKEQMEKDG

ENVCAFMGIGNSDQDMQQLNLEGKNYCAAKTLYISDSDKRKHFFLSVKMFYGNGEDIGVF

NSKRIKVISKPSKKKQSLKNADLCIQSGSTVALFNRLRSQTVSTRYLHVENGNFHASSQQ

WGAFNIHLLDDDESESEEFTVRDGYIHYGSTVKLVCSVTGMALPRLIIRKVDKQTALLDA

DDPVSQLHKCAFYMKDTERMYLCLSQERIIQFQATPCPKEPVKEMINDGASWTIISTDKA

EYTFYEGMGPVSSPLTPVPVVHSLQLNGGGDVAMLELTGDNFFPNLRVWFGDVEAETMYR

CAESIICVVPDISAFRGGWRWVRQPTQVPVSLVRNDGIIYPTGLTFTYTPEPG

>Pdu_SuH

TREGMRNYLRDRGDQVLVILHAKVAQKSYGNEKRFFCPPPCIYLFGNGWKRKREQMERDG

ASVCAFMGIGNSDQEMVQLNLEGKHYCAAKTLYISDSDKRKHFMLTVKMFYGNGQDIGVF

NSKRIKVISKPSKKKQSLKNADLCIASGTKVALFNRLRSQTVSTRYLHVEGGNFHASSTQ

WGAFTIHLLDDDEGESEEFTVRXGYIHYGMTVKLVCSVTGMALPRLIVRKVDKQTVLLDA

DEPVSQLHKCAFYMKDTERMYLCLSQERIIQFQATPCPKEPNKEMINDGAAWTIISTDKA

EYTFFEGMGPVKAPVTPVPVVSSLQLNGGGDVAMLELSGENFMPSLKVWFGDVEAETMFR

CEVSMLCVVPDISAFRSSWRWVRQPLQVPVTLVRNDGIIYATGLTFTYTPEPG

>CAPTE|Capt

----MREYLKDRHDQILVVLHAKVAQKSYGNEKRFFCPPPCIYLFGSGWKRKKEAMEREG

VTVCAFMGIGNSEQDMQQLQLDGRNYCAAKTLYISDSDKRKHFMLSVKLFYGNGDEIGLF

NSQRIKVISKPSKKKQSLKNADLCIASGSKVALFNRLRSQTVSTRYLHVENGNFHASSTQ

WGAFTIHLLDDNEGESEEFTVRDGYVHYGSTVKLVCAVTGMALPRLVMRKVDKQTVLLDA

DEPVSQLHKCAFYLKDTDRMYLCLSQEKIIQFQATACPKESNKQMINDGASWTIISTDKA

EYTFYEGMGPVKCSVTPVPVVNSLHLNGGGDVAMLELAGENFSPSLKVWFGEVEAETMYR

CAESMLCVVPDISAFRSGWKWVRQPLQVPVSLVRHDGVIYATGLTFTYTPEPG

>CRAGI|oyst

TKEAMRNYLKDRGDQVLVILHAKVAQKSYGNEKRFFCPPPCIYLFGSGWKRKKEAIEAEG

GTTCAFMGIGNSDQEMVQLNLEGKNYCAAKTLYISDSDKRKHFMLTVKMFFGNGQDIGVF

NSKRIKVISKPSKKKQSLKNADLCIASGTKVALFNRLRSQTVSTRYLHVENGNFHASSTQ

WGAFTIHLLDDNESESEEFTVRDGYIHYGSTIKLVCSVTGMALPRLIIRKVDKQTAILDA

DDPVSQLHKCAFYMKDSERMYLCLSQERIIQFQATPCPKEPNKEMINDGASWTIISTDKA

EYTFFEGMGPVKSPVSPVPVVNSLHLNGGGDVAMLELSGEFLAPNLKVWFGEVEAETMFR

CEESMFCVVPDISAFRAGWRWVRQPLQVPVLLVRSDGIIYSTGLTFTYTPEPG

>Aca_524911

------------------------------------MTP---------------------

------------------------KYCAAKTLYISDSDKRKHFMLTVKMFYGNGQDIGVF

QSKRIKVISKPSKKKQSLKNADLCIASGTKVALFNRLRSQTVSTRYLHVENGNFHASSTQ

WGAFTIHLLDDDEGESEEFTVKDGYIHYGSTVKLVCSVTGMALPRLIIRKVDKQTSLLDA

DDPVSQLHKCAFYLKDTERMYLCLSQERIIQFQATPCPKEAKREMINDGASWTIISTDKA

EYTFYEGMGPVNKPVTPVPVVNSLNLNGGGDVAMLELNSENFTAALKVWFGEVEAETMYR

CDDSMLCVVPDISAFRTGGRWVRQPLQVPVSLVRCDGVIYSTGLIFTYTPEPG

>Lotgi1|115

-REAMRKYLRDRSDQILVILHAKVAQKSYGNEKRFFCPPPCIYLFGKGWKRKHDQMEEEG

STVCAFMGIGNSDQEMVQLHLEDKDYCAAKTLYISDSDKRKHFMLSVKMFYGNGQDIGLF

LGKRIKVISKPSKKKQSLKNAELCIASGTKVALFNRLRSQTVSTRYLHVENCNFHASSTQ

WGAFTIHLLDDNEGESEEFTVRDGYIHYGSTVKLVCSVTGMALPRLVIRKVDKQTALLDA

DDPVSQLHKCAFFLKDTERMYLCLSQERIIQFQATPCPKEPNKEMINDGASWTIISTDKA

EYTFYEGMGPVKNSLTPVPVVNSLHLNGGGDVAMLELNGENFNPSLKVWFGDVEAETMFR

AEDSMLCVVPDIAAFRPGWKWVRQPLQAPVSLVRLDGVIYATGLTFTYTPEPG

**Presenilin sequences**

>ENSP00000326366

MTELPAPLSYFQNAQMSEDNHLSNTVRSQNDNRERQEHNDRRSLGHPEPLSNGRPQGNSR

QVVEQDEEEDEELTLKYGAKHVIMLFVPVTLCMVVVVATIKSVSFYTRKDGQLIYTPFTE

DTETVGQRALHSILNAAIMISVIVVMTILLVVLYKYRCYKVIHAWLIISSLLLLFFFSFI

YLGEVFKTYNVAVDYITVALLIWNFGVVGMISIHWKGPLRLQQAYLIMISALMALVFIKY

LPEWTAWLILAVISVYDLVAVLCPKGPLRMLVETAQERNETLFPALIYSSTMVWLVNMAE

GDPEAQRRVSKNSKYNAESTERESQDTVAENDDGGFSEEWEAQRDSHLGPHRSTPESRAA

VQELSSSILAGEDPEERGVKLGLGDFIFYSVLVGKASATASGDWNTTIACFVAILIGLCL

TLLLLAIFKKALPALPISITFGLVFYFATDYLVQPFMDQLAFHQFYI

>ENSP00000375745

MLAGTVRFARHCLKFFPAQKPACVDFGASRGRAMLTFMASDSEEEVCDERTSLMSAESPT

PRSCQEGRQGPEDGENTAQWRSQENEEDGEEDPDRYVCSGVPGRPPGLEEELTLKYGAKH

VIMLFVPVTLCMIVVVATIKSVRFYTEKNGQLIYTPFTEDTPSVGQRLLNSVLNTLIMIS

VIVVMTIFLVVLYKYRCYKFIHGWLIMSSLMLLFLFTYIYLGEVLKTYNVAMDYPTLLLT

VWNFGAVGMVCIHWKGPLVLQQAYLIMISALMALVFIKYLPEWSAWVILGAISVYDLVAV

LCPKGPLRMLVETAQERNEPIFPALIYSSAMVWTVGMAKLDPSSQGALQLPYDPEMEEDS

YDSFGEPSYPEVFEPPLTGYPGEELEEEEERGVKLGLGDFIFYSVLVGKAAATGSGDWNT

TLACFVAILIGLCLTLLLLAVFKKALPALPISITFGLIFYFSTDNLVRPFMDTLASHQLY

I

>S.kowalevskii_gi|291237660|ref|XP_002738746.

MSLSNEEEEDCSERSQLMETVPNVRYSGTGGSVNFSDPPDTEDGGIRTHMYGGFDRTRPT

GVREQNVESDSDEACNRRIQQESSTRRTVQQHDDEDDEELTLKYGAKHVIMLFAPVSLCM

LVVVATVGAVTFYTDDSGGAYLIYTPFHEDKGGAGTKAWNAFANAAIMIGVILVMTIFLV

ILYKKRCYKFIHGWLIVSSLMLLFLFTYIYLQELLSTYNVPMDYITLTIFMWNFGVVGMM

CIHWKGPLHLQQAYLIIISALMALVFIKYLPEWTTWVILGAISIYDLVAVLCPRGPLRVL

VETAQERNEPIFPALIYSSTMVWLVGMADNNPEQRPKRKKKKSNKKTELEQPIREPGKRH

DDALNEDLDEGGFTDWSNRQPIQTPTDPRTQSVESVDARAAASALRRSASNERPRDTTAT

LDLEEEEERGVKLGLGDFIFYSVLVGKASSYGDWTTTLACFVAILIGLCLTLLLLAIFKK

ALPALPISITFGLIFYFTTRYLVSPFTDTLASQQVYI

>Pdu_psn

MAYSAKHVIMLFVPVTLCMLVVVATISSVTFYTERNGYLVYTPFHEDSPDTGTKVWQSFA

NAFILLGAIVVMTIFLILLYRYECYRFIHGWLIVSSLMLLFLFSYLYLGEVLKAYNVPMD

YITVALLMWNFGVVGMVCIHWKGPLRLQQAYLIMVSALMALIFIKYLPDWTTWVVLAVIS

VWDLVAVLCPKGPLRMLVETAQERNEPIFPALIYSSTMVWIVGMADTDGANGGSKKKKKK

KRVDSTEIQLQPNDLEPQLVDGTTNPAISQTPDDEDDGGFREFQNNTGQTDRQTRRQHIA

NNVANSTNSHPTAPRTGADPEVERSRQIRRQREQEMEEEERGIKLGLGDFIFYSVLVGKA

SSYGDWNTTLACFVAILIGLCFTLLLLAIFRRALPALPISITFGLVFYFATSQLVRPFMD

KLSSNQVFI

>CAPTE|CapteP159305 pep:novel supercontig:GCA_000328365.1:CAPTEscaffold_220:12897:14901:1 gene:CapteG159305 transcript:CapteT159305 description:""

MLYGAKHVIMLFVPVSLCMLVVVATISSINFYTTKGGYLIYTPFTEETDDTGTKVWMSLA

NAFIVIIVVVVMTVFLILLYKYRCYKVIHGWLIVSSAMLLFLFSYIYLGEVLRTYNVPMD

YITVALLMWNFGVVGMVCIHWKGPLLLQQGYLIMVSALMALIFIKYLPDWTAWVLLAFIS

IWDLIAVLCPKGPLRILVETAQERNEPMLPGLIYSSTMVWMFGMADVDPVRDAEQSAMMK

GSDTMPSLEEGETNQASDSQDDQDGGFAERDTHSPNREARRRQVASNSSTNSHTARTAVQ

VLGNDVEPNGRARNDNSSGEEEERGVKLGLGDFIFYSVLVGKASAEGDWNTTIACFIAIL

IGLSFTLLLLAIVRKALPALPISIAFGLTFYFTTSQLVQPFMDRCSSRQVFI

>HelroP87358 pep:novel supercontig:GCA_000326865.1:HELROscaffold_57:750979:755224:1 gene:HelroG87358 transcript:HelroT87358 description:""

MVLIEEDLQYGAESIIMLFIPVSICISFVIASVQALDFYKEEGLPLPFTPFVPEEKDEVK

DTLWKSLANSLIIVGVVIIMTILLFLLFKYRCYKVCAFWMFLATLLLLFIFSYIYYSEFC

RYLNTATDFISTGFNLWNFGLMGILAIHWKSPLIVQQFYLIVVSALMALTFIKHLPGWTA

WALLIVISVWDLIAVLTPKGPLKGLVNISSERRENLFPALVYSSTPFSAVVATSSTSQDD

IHTEVYHVSTATAASGDHVYNGNNYDDDSTNLAYMNRFIHSKPDRKDEPKISSTIYNAGI

KLGLGDFIFYSLLVGKVSQLEEWNTTTACFISVLIGLSLTILLLVLYEQALPALPISVFF

GVAFYFGGYYTIDPFVSVCVSRQAFI

>HelroP114139 pep:novel supercontig:GCA_000326865.1:HELROscaffold_44:1448547:1457943:1 gene:HelroG114139 transcript:HelroT114139 description:""

MDEKLDCEKSKLIKSNEILEAMAFDGEVRDLNNENDIEMNNVTSNNGISDAADDGNLSGE

PRSPEQNVTNNDSNNPVNIDEYLDELDLKYGAGHVIQLFIPVSLCMAAVVCGINFIEFYN

VKDVYLMYTPFEQSVTPETSTGTRIWQSLANALIMLSVVIVMTVVLLLLYKYRCYKFIAG

WLFMSSFMLLFFFTFIFYSELCRTMNIPMDYITTAVFLWNFGVLGIMCIHWKGPLLLQQF

FLIVVSALMALTFIKHLPDWTTWVLLAVISIWDLVAVLAPKGPLRSLVKLAQERNDPIFP

ALIYSTTMAWLVEPSSGAYEADNDGGADAEADVDGEGDNVIEIHDDQVPEGQIPEGHVPE

GHVHEGHVPESHVPESHVPEGHVPEGHVPEGHILSSDKAGTSANVDYVNLEHFTTEITIA

AEKKSDEPDQQERPAEEQGEPQAKQKRSKHRQHSKQQNRPKGPSQQQNEQQQIGRLGHPE

SFEQFKQQVARLDKMEDSGVKLGLGDFIFYSVLVGKASLLGDWNTTVACFIAILMGLCFT

LLLLASFRKALPALPVSIAFGLIFYFATSQVVLPFMVMCSLNQVFI

>Acal_gi|46981378|gb|AAT07667.1

MNPQRNSTTSVGVDSSPTERTGLVSNLTGGEWAGESGNFSPVRRYSAADDEATTVPETSV

VTPSTNSTTNQQPQSQRTRRGEGSQNRSDGDGAAATRGGGAQQEEEDDGEETLLYGAKHV

IMLFVPVTLCMVVVVATISSITYYTTKGTYFLYTPFHDKTEDTGTKLWQSMANALILLGA

IIVMTIVLLLLYKYECYKIINGWLVMSSVMLLFFFSYIYLEQILRAYNVPMDYITVAIIM

WNFGVGGLFCIHWKGPLLLQQAYLISISALVALMFIKFLPDWTTWAVLGVMMVWDLVAVL

CPKGPLRMLVETAQNRNEPIFPALIYSSTMVWTITMADGDPNKKKNQKKKKQETTTAEES

ANGAQASGGADEDDGGFQEHIQNGGSRSRGLSSGSESNSARTAVAALGDMSQADSPRQQR

AKVDTVAVDAETTVVVNRNPPRPRAQRAATDQQNRQRNESETDVASSEADEDRGVKLGLG

DFIFYGVLVGKASSNGDWNTTLACFVAILIGLCFTLLLLAIFRKALPALPISLTFGLVFN

FATSALVRPFMDSLASEQVYI

>Lotgi1|207659|estExt_fgenesh2_pm.C_sca_1280010

MSGTIDNSSSSSGGSATERTGLMSNRGSRSGQTPQSPQQQDGGGIFNDSANITPLTERSR

NAENRNDNRSRRSAADTSVNNAERPTHNSQQRPSGGGGDGGEEEEEESFMYGANSVIMLF

VPVTLCMAVVVATISSVTFYTEKTDGYLIYTPFHDKTDDTGTKLWQSFANAFILLGVICV

LTIFLLLLYKFRCYKVIHGWLIVSSLMLLFLFSYIYLGEVLRAYNVPMDYVTVIILMWNF

GVVGMVCIHWKGPLLLQQAYLIMISALMALIFIKYLPDWTTWVVLGVMVVWDLVAVLCPK

GPLRVLVETAQERNEPIFPALIYSSTVMYQTITMADDAEGNKKKRKQKKESNTVEENGAT

PVQTDGAVDDEDDGGFRCNTRYSSPMTASTESTRARNAVQALNTTNNYQTIADPDRPPGT

ATPNQRSSTNSQPQTQDEEERGVKLGLGDFIFYGVLVGKASSYGDWNTTLACFVAILIGL

CFTLLLLAIFKKALPALPISITFGLVFNFATSSLVQPFMDSLAAEQVYL

>CRAGI|oyster|CGI_10019579

MSLNASSSTDSDSLSETKPLMETHVDVNRRQYEDIRSNDRATRESAQAEENHDREVNTQS

RGNRTSAQRRPARSEEEEEDDTLLYGAKHVIMLFVPVSLCMAVVVATISSVTFYTEKGGY

LVYTPFHDGDKADADTGTKVWQSVANALILLGVICVMTIVLLLLYKFRCYKVIHGWLIAS

SLMLLFLFSYIYLGVVLQAYNVAMDYITVALLMWNFGVVGMICIHWKGPLFLQQAYLIVI

SALMALVFIKYLPDWTTWVVLGVMVIWDLVAVLCPKGPLRILVETAQERNEPIFPALIYS

STMIWSVVGMADDGSDKKKKKKKKSKKKPEAATAEPDQVEAELDGGFLEERPTRQLSQST

EETARAVRAARVLGDTENGQRTNSPRNPTREEEEEEEEEERGVKLGLGDFIFYGVLVGKA

SSNGDWNTTLACFVAILIGLCFTLLLLAIFRKALPALPISITFGLIFNFTTSLLVQPFAD

RLSASQVYI

>Smed_mk4.000085.14.01

MVLILSENKFIDYIRATSEIVIAMNIYMDFLTLTLFIWNFGVVGFLMIHWKGPLILQQAY

LIIISAKVALILLKFMPLWTTWVVLGALAIWDLVAVLCPFGPLRILVEIASERNEPIFPA

LIYSTTATMMVKIPDSQGNSNPNPQLTHIQNTDSRDRVQIKDDRSRIRRNIRDIHEDVMD

RGDRGVKLGLGDFVFYSLLMGRATLDSDFNTIMACYVAILVGMFLTILLLTMAGRALPAL

PISMACGILFYFLTSLIISPFTKVLWMKRAFI

>Smed_mk4.000085.15.01

MAEDNTGYLQHNRQDTLEEHIDPKKLLAFGSKQIISLFVPVSICMLMVGFVAKTVSYYAT

TDHYLIYTPFHTKDADVGTTVWQSLANALIMIVLVVVMSIILVLLYKYKCYKAIEHLLLP

IMLKLNFAATIHDLH

>Tca_189239692|ref|XP_967139.2

MSESESEFASVSEKTRLMDGHVETNGSGNSLSGDSRVGPEGSRKRRSRRLPEDDDVPEAQ

IVTGGSSRPPPVDYEVAELRDEEEELKYGAKHVIKLFAPVSLCMVVVVATISAVNFYSVK

DMYLVYTPFHEESSDTSTKVWNAAANSLILMAVIVVMTVLLIVLYKYRCYKTIHGWLILS

SLMLLTVFSYLYLEEILRAYNIPMDYPTIVLLMWNFGVMGMVCIHWQGPLVLQQAYLIFV

AALMALVFIKYLPEWTTWAVLAVISVWDLVAVLMPKGPLRILVETAQERNEQIFPALIYS

STYMYAYTSMATPSNDENPPRGAAARSSADSTEEHGFTQDWVDNHSSRVAQRRLEVHDVP

RGAPRTQIHQEEEERGVKLGLGDFIFYSVLVGKASSYGDWNTTLACFVAILIGLCLTLLL

LAIFKKALPALPISITFGLIFYFATKEIVSPFADSLANEQVFI

>ISCW020583-PA pep:known scaffold:IscaW1:DS846142:70190:86926:-1 gene:ISCW020583 transcript:ISCW020583-RA description:"presenilin, putative"

VAFSDGSASREGTEVDLQPASGGGGRAWGESGTEEEEEELKYGAKHVIRLFVPVSLCMLV

VVATISSISFYRHTNTYLVYTPFTDQTVDTGTKVWQSFANAFILMGVIVVMTILLILLYK

FRCYKVIHAWLIVSSLLLVFLFAYIYLGEVLRAYNVPMDYLTVALVMWNFGVVGMISIHW

KGPLLLQQAYLILVSALMALVFIKYLPDWTAWVVLGVISVWDLIAVLCPKGPLRILVETA

QERNEPIFPALIYSSAMVWNVVTMADRDPFYSSQNQHASQSDASSGTSRRPCSTFPRMRG

SGRSRRRSQRGSMANSGTLAALGSSQGSAPEVDDLPRQEEEERGVKLGLGDFIFYSVLVG

KASSYGDWNTTIACFVAILIGLCLTLLLLAIFKKALPALPISITFGLIFYFATSSLVQPF

ADSLAGEQVFI

>ISCW022390-PA pep:novel scaffold:IscaW1:DS931786:277787:279145:-1 gene:ISCW022390 transcript:ISCW022390-RA description:"presenilin, putative"

KMCHSMTAQYRHIMRQVITLICALSICMASVACLVRILKPSKNDGMSQPYVRYPVEYDAE

PVLLMNSFANAFSFLSMIMIVNCTLVLLYKGGYTNVIKAWLMTGSGVLLFVVTYYYMGRC

VYYFNFPMDHLSCSFIVWNIGMTGMGTLYYKGPFVMHQGFVIYVSILMAVVLEESFPEWT

AWILMILVSLWDMFAVLCVIGPLRMLIETAHERNEPLFPALLFSTSSAWCYDLSAQTPTN

AEKLPRRLLNFRGSTNTEDSFVVSRMKAIQEIRRQEDVLCSHHHHHTHQPRHHRQRRQDA

AAPEDTGMKMGLGDFIFYSILVGKASRHGTVSAVVICYIYVVIGIILTLALLVIAQKPVP

ALPLSISLGMFAYFSTISFVEPFMEETGVLSL

>ISCW003378-PA pep:novel scaffold:IscaW1:DS661556:788:1951:-1 gene:ISCW003378 transcript:ISCW003378-RA description:"seven trans-membrane protein, putative"

EHVVRLLAAVSCCMLFVVTSIQISTTFSDSGGQYLPYTPFAETNVTTATRAVNSVANALI

LIAVIVTMTVLIVSLYYFRFYKVIKGWIFLASCLLLYGSTAAFVHQICLIYNVPMDYISV

ALFIWNFGTLGMVVIQSKGPLVVQQGYLIVESAFMALVFIKYVPEWTLWVLLCVIPIWDL

IAVLCVVGPLKILVETAKERNEGLQPGLIFATVVAGGFAGMASRKGSGPQRGSIDTPREE

EPEGSRDEPRKEAVQDDSLSDSFSDPVEEKSGVKMGLGDFVFYSILVGKVATYGDLNVVA

ACFIAVLVGICVTLLLLSMLRVALPALPVSLALGLLFAFPQELIHEFMQPFLEQQIHV

>Nemve1|217043 + 217044

MSEDSADETSKLMGDESPVRISGRRSNARREERDLRDEDDDEMCDRENDPRNLRRQSSTD

NVLPQVEDDNESEADEEEMLKYGARSVMMLIIPVSTCMLVVVATISSVTYYTENSGQYLV

YTPFHEETGISNAQKAGEAIANALIVIGVVLVLTIILVVLYKFRCYCIISGWLVLSSLML

LFFFGYIYFQELLRVYNVAMDYITLSLILWNFGVVGMICIHWKGPLILQQAYLILVSALM

ALVFIKYLPDWTTWAILAAISLYDLFAVLCPKGPLKILVQTAQERDEPLFPSLIYSSTMM

WTVGMADRDPSSNHPASQENSAGENEEEVERSGEGPTSEEARNAVRNLGEAGQQQPQQNG

EEEEKGVKLGLGDFIFYSVLVGKASSYKDWNTTIACFVAILIGLCLTLLLLAIYRKALPA

LPISITFGLIFNFATKELVKPFMDSLSSKQAFI

>MNELE| ML01594a

MSGIRMSSVAVTAENCENGEPQAPEESHQGQANIPEIVEPEDDDAEEQEMLKYGAKHVMM

LIVPVSLCMIIVIFAIRIITMYDQVSQPQVYLVYTPFNDQTKKTTGSPISDAVLNMLIVL

GLVIFMTCVLVLLYKYRCYKVIEGWLFLSSLILLFMFTGLNLHEIVMRFNFAMDYITISL

IVWNFGVMGIISVHWKGPLIVQQGYLLIISAMMALVFIKYLPEWTLWFILGGIAVYDLFA

VLCPKGPLRILVETAEERNEPIFPSLIYSSTMMWTITMAEGDPVSSYNPLNSSSSSSAQG

SPPARPQPEMEPEEQGVKLGLGDFIFYSVLVGKAATADDWNTVMACFVAILIGLCLTLLI

LAIVKKALPALPISIFVGIIFYVCTRWCIKPMCDQMALSQVYV

>Monbr1|29512|fgenesh2_pg.scaffold_35000054

MMMMNCLSLSLSLSSLSLSLSLSIHSLSLSLLLLSTLLFTSLFSNQFISLLSLSLSSLCF

LTGVVNTMIRNENKRILHTQREREREREKRQRWLASLSLSLSLSLSHSESALLRRDRSIM

AGDGSEAPEVASTNSGDPLIEHDRRTTQDDGRRATRPLDANVHERLLSAGRENSVNYTAE

EREARIRARQEEAARRELELDLKYDAESVLALIKPVSACMIVVIATIRSITYFSQNDTQF

AYTPFESNGGAGESSGERFGGAVLNALIVVGIVIVMTFILVMLYIYEYYKIIYGWLALSA

LLLLYFFSYQYIECVMCTRRQVLIAHNASIDWITMAFIIWNFGTVGFIAIFWRSPLAVQQ

VYLVIVSALMALVLIKNLPDWTTWVLLAAIAIYDLFAVLSPCGPLKCLVEVAQERNQPLF

PSLIYSSTMMWTVTMADVGDSSSNSATIESQPRVADGGASTRGTEMTSMSSQPRARQGSR

ADSEADDGARDISTFAAPRGETRSTNQAAPASQPSFQPDDEDDSGVKLGLGDFIFYSVLV

GKAATAHEWTTILACYVAILIGLACTLLLLSIFKKALPALPISIFFGLCFFFLTSEVLDP

LIDRLNERQIFL

>Aqu1.225250_PAC:15723778

MEEQEEDGITVSPPLQGTREEEEETEQKDDEQSCGMKYVFKKILNVLPSFGDDGDDPTSE

ANEEALRFGAQQMILLIFPVFICMALVVAIQLSVEKNVTSSGTLIYTPFDEDTASNDGFV

LLFALANVAIVITLVVVMTIILVCLYNEVLQVHYLFIDWPSFLVLIWNFGGMGVLVIHWK

GPLRLQQAYLIFCSALTANIFVKYLPNWTAWILLAAISLYDLIAVLCPKGPLRVLVETAR

ERNETIFPSLIYSTTMVWLVGMADRPTNKKKDKKNTDDTSGSSDGSHEEEEEEEGGGERE

GRRDNDDGDDDTDGIEMRSIRAAADTNRERRRERQQQQQQEEEEEEEEQEERQGFKLGLG

DFIFYSILVGKAAHDSTGDWVVISSCFVAILIGLCMTIIILGIVRRALPALPISIFCGLI

FYFSSQYVIAPFAQVLATTQTFI

**Presenilin alignment**

>HroP87358

-MVLIEEDLQYGAESIIMLFIPVSICISFVIASVQALDFYKEEGLPLPFTPFVPEKDEVK

DTLWKSLANSLIIVGVVIIMTILLFLLFKYRCYKVCAFWMFLATLLLLFIFSYIYYSEFC

RYLNTATDFISTGFNLWNFGLMGILAIHWKSPLIVQQFYLIVVSALMALTFIKHLPGWTA

WALLIVISVWDLIAVLTPKGPLKGLVNISSERRENLFPALVYSSTPFSAVVATSSPDRKD

EPKISSTI-YNAGIKLGLGDFIFYSLLVGKVSQLEEWNTTTACFISVLIGLSLTILLLVL

YEQALPALPISVFFGVAFYFGGYYTIDPFVSVCVSRQAFI

>HroP114139

DEYLDELDLKYGAGHVIQLFIPVSLCMAAVVCGINFIEFYNVKDVYLMYTPFEQSETSTG

TRIWQSLANALIMLSVVIVMTVVLLLLYKYRCYKFIAGWLFMSSFMLLFFFTFIFYSELC

RTMNIPMDYITTAVFLWNFGVLGIMCIHWKGPLLLQQFFLIVVSALMALTFIKHLPDWTT

WVLLAVISIWDLVAVLAPKGPLRSLVKLAQERNDPIFPALIYSTTMAW----LVEPKGPS

QQQNEQQQMEDSGVKLGLGDFIFYSVLVGKASLLGDWNTTVACFIAILMGLCFTLLLLAS

FRKALPALPVSIAFGLIFYFATSQVVLPFMVMCSLNQVFI

>ISC22390-P

MCHSMTAQYRHIMRQVITLICALSICMASVACLVRILKPSKNDGMSQPYVRYPVEYDAEP

VLLMNSFANAFSFLSMIMIVNCTLVLLYKGGYTNVIKAWLMTGSGVLLFVVTYYYMGRCV

YYFNFPMDHLSCSFIVWNIGMTGMGTLYYKGPFVMHQGFVIYVSILMAVVLEESFPEWTA

WILMILVSLWDMFAVLCVIGPLRMLIETAHERNEPLFPALLFSTSSAW-CYDLSAPRHHR

QRRQDAAAPEDTGMKMGLGDFIFYSILVGKASRHGTVSAVVICYIYVVIGIILTLALLVI

AQKPVPALPLSISLGMFAYFSTISFVEPFME--ETGVLSL

>ISC03378-P

-------------EHVVRLLAAVSCCMLFVVTSIQISTTFSDGGQYLPYTPFAETNVTTA

TRAVNSVANALILIAVIVTMTVLIVSLYYFRFYKVIKGWIFLASCLLLYGSTAAFVHQIC

LIYNVPMDYISVALFIWNFGTLGMVVIQSKGPLVVQQGYLIVESAFMALVFIKYVPEWTL

WVLLCVIPIWDLIAVLCVVGPLKILVETAKERNEGLQPGLIFATVVAGGFAGMASQDDSL

SDSFSDPVEEKSGVKMGLGDFVFYSILVGKVATYGDLNVVAACFIAVLVGICVTLLLLSM

LRVALPALPVSLALGLLFAFP-QELIHEFMQPFLEQQIHV

>Monbr1|295

ARRELELDLKYDAESVLALIKPVSACMIVVIATIRSITYFSQNDTQFAYTPFESNGESSG

ERFGGAVLNALIVVGIVIVMTFILVMLYIYEYYKIIYGWLALSALLLLYFFSYQYIEQVL

IAHNASIDWITMAFIIWNFGTVGFIAIFWRSPLAVQQVYLVIVSALMALVLIKNLPDWTT

WVLLAAIAIYDLFAVLSPCGPLKCLVEVAQERNQPLFPSLIYSSTMMW-TVTMADAAPAS

QPSFQPDDEDDSGVKLGLGDFIFYSVLVGKAATAHEWTTILACYVAILIGLACTLLLLSI

FKKALPALPISIFFGLCFFFLTSEVLDPLIDRLNERQIFL

>Sme15.01

EHIDPKKLLAFGSKQIISLFVPVSICMLMVGFVAKTVSYYATTDHYLIYTPFHTKDADVG

TTVWQSLANALIMIVLVVVMSIILVLLYKYKCYKAIEHLLL-------------------

-------------------------------PIMLKLNFAATI-----------------

---------HDLH-----------------------------------------------

------------------------------------------------------------

----------------------------------------

>Sme14.01

--------------------------MVLILSENKFIDYIRA------------------

-------------------------------------------------------TSEIV

IAMNIYMDFLTLTLFIWNFGVVGFLMIHWKGPLILQQAYLIIISAKVALILLKFMPLWTT

WVVLGALAIWDLVAVLCPFGPLRILVEIASERNEPIFPALIYSTTATM-MVKIPDRRNIR

DIHEDVMDRGDRGVKLGLGDFVFYSLLMGRATLDSDFNTIMACYVAILVGMFLTILLLTM

AGRALPALPISMACGILFYFLTSLIISPFTKVLWMKRAFI

>MNELE|_ML0

DDAEEQEMLKYGAKHVMMLIVPVSLCMIIVIFAIRIITMYDQPQVYLVYTPFNDQKKTTG

SPISDAVLNMLIVLGLVIFMTCVLVLLYKYRCYKVIEGWLFLSSLILLFMFTGLNLHEIV

MRFNFAMDYITISLIVWNFGVMGIISVHWKGPLIVQQGYLLIISAMMALVFIKYLPEWTL

WFILGGIAVYDLFAVLCPKGPLRILVETAEERNEPIFPSLIYSSTMMW-TITMAEPPARP

QPEME---PEEQGVKLGLGDFIFYSVLVGKAATADDWNTVMACFVAILIGLCLTLLILAI

VKKALPALPISIFVGIIFYVCTRWCIKPMCDQMALSQVYV

>Nemve1|217

SEADEEEMLKYGARSVMMLIIPVSTCMLVVVATISSVTYYTESGQYLVYTPFHEEGISNA

QKAGEAIANALIVIGVVLVLTIILVVLYKFRCYCIISGWLVLSSLMLLFFFGYIYFQELL

RVYNVAMDYITLSLILWNFGVVGMICIHWKGPLILQQAYLILVSALMALVFIKYLPDWTT

WAILAAISLYDLFAVLCPKGPLKILVQTAQERDEPLFPSLIYSSTMMW-TVGMADEAGQQ

QPQQNGEE-EEKGVKLGLGDFIFYSVLVGKASSYKDWNTTIACFVAILIGLCLTLLLLAI

YRKALPALPISITFGLIFNFATKELVKPFMDSLSSKQAFI

>S.kowalevs

DEDDEELTLKYGAKHVIMLFAPVSLCMLVVVATVGAVTFYTDGGAYLIYTPFHEDKGGAG

TKAWNAFANAAIMIGVILVMTIFLVILYKKRCYKFIHGWLIVSSLMLLFLFTYIYLQELL

STYNVPMDYITLTIFMWNFGVVGMMCIHWKGPLHLQQAYLIIISALMALVFIKYLPEWTT

WVILGAISIYDLVAVLCPRGPLRVLVETAQERNEPIFPALIYSSTMVW-LVGMADRPRDT

TATLDLEEEEERGVKLGLGDFIFYSVLVGKASSYGDWTTTLACFVAILIGLCLTLLLLAI

FKKALPALPISITFGLIFYFTTRYLVSPFTDTLASQQVYI

>Acal_gi|46

EEDDGEETLLYGAKHVIMLFVPVTLCMVVVVATISSITYYTTKGTYFLYTPFHDKTEDTG

TKLWQSMANALILLGAIIVMTIVLLLLYKYECYKIINGWLVMSSVMLLFFFSYIYLEQIL

RAYNVPMDYITVAIIMWNFGVGGLFCIHWKGPLLLQQAYLISISALVALMFIKFLPDWTT

WAVLGVMMVWDLVAVLCPKGPLRMLVETAQNRNEPIFPALIYSSTMVW-TITMADRNESE

TDVASSEADEDRGVKLGLGDFIFYGVLVGKASSNGDWNTTLACFVAILIGLCFTLLLLAI

FRKALPALPISLTFGLVFNFATSALVRPFMDSLASEQVYI

>Tca_189239

ELRDEEEELKYGAKHVIKLFAPVSLCMVVVVATISAVNFYSVKDMYLVYTPFHEESSDTS

TKVWNAAANSLILMAVIVVMTVLLIVLYKYRCYKTIHGWLILSSLMLLTVFSYLYLEEIL

RAYNIPMDYPTIVLLMWNFGVMGMVCIHWQGPLVLQQAYLIFVAALMALVFIKYLPEWTT

WAVLAVISVWDLVAVLMPKGPLRILVETAQERNEQIFPALIYSSTYMYAYTSMATPRGAP

RTQIH-QEEEERGVKLGLGDFIFYSVLVGKASSYGDWNTTLACFVAILIGLCLTLLLLAI

FKKALPALPISITFGLIFYFATKEIVSPFADSLANEQVFI

>CAPTE|Capt

--------MLYGAKHVIMLFVPVSLCMLVVVATISSINFYTTKGGYLIYTPFTEETDDTG

TKVWMSLANAFIVIIVVVVMTVFLILLYKYRCYKVIHGWLIVSSAMLLFLFSYIYLGEVL

RTYNVPMDYITVALLMWNFGVVGMVCIHWKGPLLLQQGYLIMVSALMALIFIKYLPDWTA

WVLLAFISIWDLIAVLCPKGPLRILVETAQERNEPMLPGLIYSSTMVW-MFGMADPNGRA

RNDNSSGEEEERGVKLGLGDFIFYSVLVGKASAEGDWNTTIACFIAILIGLSFTLLLLAI

VRKALPALPISIAFGLTFYFTTSQLVQPFMDRCSSRQVFI

>ISCW020583

GTEEEEEELKYGAKHVIRLFVPVSLCMLVVVATISSISFYRHTNTYLVYTPFTDQTVDTG

TKVWQSFANAFILMGVIVVMTILLILLYKFRCYKVIHAWLIVSSLLLVFLFAYIYLGEVL

RAYNVPMDYLTVALVMWNFGVVGMISIHWKGPLLLQQAYLILVSALMALVFIKYLPDWTA

WVVLGVISVWDLIAVLCPKGPLRILVETAQERNEPIFPALIYSSAMVWNVVTMADGSAPE

VDDLPRQEEEERGVKLGLGDFIFYSVLVGKASSYGDWNTTIACFVAILIGLCLTLLLLAI

FKKALPALPISITFGLIFYFATSSLVQPFADSLAGEQVFI

>Lotgi1|207

EE-EEEESFMYGANSVIMLFVPVTLCMAVVVATISSVTFYTEKDGYLIYTPFHDKTDDTG

TKLWQSFANAFILLGVICVLTIFLLLLYKFRCYKVIHGWLIVSSLMLLFLFSYIYLGEVL

RAYNVPMDYVTVIILMWNFGVVGMVCIHWKGPLLLQQAYLIMISALMALIFIKYLPDWTT

WVVLGVMVVWDLVAVLCPKGPLRVLVETAQERNEPIFPALIYSSTVMYQTITMADQRSST

NSQPQTQDEEERGVKLGLGDFIFYGVLVGKASSYGDWNTTLACFVAILIGLCFTLLLLAI

FKKALPALPISITFGLVFNFATSSLVQPFMDSLAAEQVYL

>CRAGI|oyst

EEEEEDDTLLYGAKHVIMLFVPVSLCMAVVVATISSVTFYTEKGGYLVYTPFHDGDADTG

TKVWQSVANALILLGVICVMTIVLLLLYKFRCYKVIHGWLIASSLMLLFLFSYIYLGVVL

QAYNVAMDYITVALLMWNFGVVGMICIHWKGPLFLQQAYLIVISALMALVFIKYLPDWTT

WVVLGVMVIWDLVAVLCPKGPLRILVETAQERNEPIFPALIYSSTMIWSVVGMADPRNPT

REEEEEEEEEERGVKLGLGDFIFYGVLVGKASSNGDWNTTLACFVAILIGLCFTLLLLAI

FRKALPALPISITFGLIFNFTTSLLVQPFADRLSASQVYI

>Pdu_psn

--------MAYSAKHVIMLFVPVTLCMLVVVATISSVTFYTERNGYLVYTPFHEDSPDTG

TKVWQSFANAFILLGAIVVMTIFLILLYRYECYRFIHGWLIVSSLMLLFLFSYLYLGEVL

KAYNVPMDYITVALLMWNFGVVGMVCIHWKGPLRLQQAYLIMVSALMALIFIKYLPDWTT

WVVLAVISVWDLVAVLCPKGPLRMLVETAQERNEPIFPALIYSSTMVW-IVGMADSRQIR

RQREQEMEEEERGIKLGLGDFIFYSVLVGKASSYGDWNTTLACFVAILIGLCFTLLLLAI

FRRALPALPISITFGLVFYFATSQLVRPFMDKLSSNQVFI

>Aqu1.22525

TSEANEEALRFGAQQMILLIFPVFICMALVVAIQLSVEKNVTSSGTLIYTPFDEDASNDG

FVLLFALANVAIVITLVVVMTIILVCLYN----------------------------EVL

QVHYLFIDWPSFLVLIWNFGGMGVLVIHWKGPLRLQQAYLIFCSALTANIFVKYLPNWTA

WILLAAISLYDLIAVLCPKGPLRVLVETARERNETIFPSLIYSTTMVW-LVGMADQQQQE

EEEEEEEQEERQGFKLGLGDFIFYSILVGKAAHTGDWVVISSCFVAILIGLCMTIIILGI

VRRALPALPISIFCGLIFYFSSQYVIAPFAQVLATTQTFI

>ENSP326366

EEEDEELTLKYGAKHVIMLFVPVTLCMVVVVATIKSVSFYTRKDGQLIYTPFTEDTETVG

QRALHSILNAAIMISVIVVMTILLVVLYKYRCYKVIHAWLIISSLLLLFFFSFIYLGEVF

KTYNVAVDYITVALLIWNFGVVGMISIHWKGPLRLQQAYLIMISALMALVFIKYLPEWTA

WLILAVISVYDLVAVLCPKGPLRMLVETAQERNETLFPALIYSSTMVW-LVNMAEVQELS

SSILAGEDPEERGVKLGLGDFIFYSVLVGKASASGDWNTTIACFVAILIGLCLTLLLLAI

FKKALPALPISITFGLVFYFATDYLVQPFMDQLAFHQFYI

>ENSP375745

GL-EEELTLKYGAKHVIMLFVPVTLCMIVVVATIKSVRFYTEKNGQLIYTPFTEDTPSVG

QRLLNSVLNTLIMISVIVVMTIFLVVLYKYRCYKFIHGWLIMSSLMLLFLFTYIYLGEVL

KTYNVAMDYPTLLLTVWNFGAVGMVCIHWKGPLVLQQAYLIMISALMALVFIKYLPEWSA

WVILGAISVYDLVAVLCPKGPLRMLVETAQERNEPIFPALIYSSAMVW-TVGMAKPPLTG

YPGEELEEEEERGVKLGLGDFIFYSVLVGKAAASGDWNTTLACFVAILIGLCLTLLLLAV

FKKALPALPISITFGLIFYFSTDNLVRPFMDTLASHQLYI

**Notch sequences**

>Hsa_364163

MQPPSLLLLLLLLLLLCVSVVRPRGLLCGSFPEPCANGGTCLSLSLGQGTCQCAPGFLGE

TCQFPDPCQNAQLCQNGGSCQALLPAPLGLPSSPSPLTPSFLCTCLPGFTGERCQAKLED

PCPPSFCSKRGRCHIQASGRPQCSCMPGWTGEQCQLRDFCSANPCVNGGVCLATYPQIQC

HCPPGFEGHACERDVNECFQDPGPCPKGTSCHNTLGSFQCLCPVGQEGPRCELRAGPCPP

RGCSNGGTCQLMPEKDSTFHLCLCPPGFIGPDCEVNPDNCVSHQCQNGGTCQDGLDTYTC

LCPETWTGWDCSEDVDECETQGPPHCRNGGTCQNSAGSFHCVCVSGWGGTSCEENLDDCI

AATCAPGSTCIDRVGSFSCLCPPGRTGLLCHLEDMCLSQPCHGDAQCSTNPLTGSTLCLC

QPGYSGPTCHQDLDECLMAQQGPSPCEHGGSCLNTPGSFNCLCPPGYTGSRCEADHNECL

SQPCHPGSTCLDLLATFHCLCPPGLEGQLCEVETNECASAPCLNHADCHDLLNGFQCICL

PGFSGTRCEEDIDECRSSPCANGGQCQDQPGAFHCKCLPGFEGPRCQTEVDECLSDPCPV

GASCLDLPGAFFCLCPSGFTGQLCEVPLCAPNLCQPKQICKDQKDKANCLCPDGSPGCAP

PEDNCTCHHGHCQRSSCVCDVGWTGPECEAELGGCISAPCAHGGTCYPQPSGYNCTCPTG

YTGPTCSEEMTACHSGPCLNGGSCNPSPGGYYCTCPPSHTGPQCQTSTDYCVSAPCFNGG

TCVNRPGTFSCLCAMGFQGPRCEGKLRPSCADSPCRNRATCQDSPQGPRCLCPTGYTGGS

CQTLMDLCAQKPCPRNSHCLQTGPSFHCLCLQGWTGPLCNLPLSSCQKAALSQGIDVSSL

CHNGGLCVDSGPSYFCHCPPGFQGSLCQDHVNPCESRPCQNGATCMAQPSGYLCQCAPGY

DGQNCSKELDACQSQPCHNHGTCTPKPGGFHCACPPGFVGLRCEGDVDECLDQPCHPTGT

AACHSLANAFYCQCLPGHTGQWCEVEIDPCHSQPCFHGGTCEATAGSPLGFICHCPKGFE

GPTCSHRAPSCGFHHCHHGGLCLPSPKPGFPPRCACLSGYGGPDCLTPPAPKGCGPPSPC

LYNGSCSETTGLGGPGFRCSCPHSSPGPRCQKPGAKGCEGRSGDGACDAGCSGPGGNWDG

GDCSLGVPDPWKGCPSHSRCWLLFRDGQCHPQCDSEECLFDGYDCETPPACTPAYDQYCH

DHFHNGHCEKGCNTAECGWDGGDCRPEDGDPEWGPSLALLVVLSPPALDQQLFALARVLS

LTLRVGLWVRKDRDGRDMVYPYPGARAEEKLGGTRDPTYQERAAPQTQPLGKETDSLSAG

FVVVMGVDLSRCGPDHPASRCPWDPGLLLRFLAAMAAVGALEPLLPGPLLAVHPHAGTAP

PANQLPWPVLCSPVAGVILLALGALLVLQLIRRRRREHGALWLPPGFTRRPRTQSAPHRR

RPPLGEDSIGLKALKPKAEVDEDGVVMCSGPEEGEEVGQAEETGPPSTCQLWSLSGGCGA

LPQAAMLTPPQESEMEAPDLDTRGPDGVTPLMSAVCCGEVQSGTFQGAWLGCPEPWEPLL

DGGACPQAHTVGTGETPLHLAARFSRPTAARRLLEAGANPNQPDRAGRTPLHAAVAADAR

EVCQLLLRSRQTAVDARTEDGTTPLMLAARLAVEDLVEELIAAQADVGARDKWGKTALHW

AAAVNNARAARSLLQAGADKDAQDNREQTPLFLAAREGAVEVAQLLLGLGAARELRDQAG

LAPADVAHQRNHWDLLTLLEGAGPPEARHKATPGREAGPFPRARTVSVSVPPHGGGALPR

CRTLSAGAGPRGGGACLQARTWSVDLAARGGGAYSHCRSLSGVGAGGGPTPRGRRFSAGM

RGPRPNPAIMRGRYGVAAGRGGRVSTDDWPCDWVALGACGSASNIPIPPPCLTPSPERGS

PQLDCGPPALQEMPINQGGEGKK

>Hsa_256646

MPALRPALLWALLALWLCCAAPAHALQCRDGYEPCVNEGMCVTYHNGTGYCKCPEGFLGE

YCQHRDPCEKNRCQNGGTCVAQAMLGKATCRCASGFTGEDCQYSTSHPCFVSRPCLNGGT

CHMLSRDTYECTCQVGFTGKECQWTDACLSHPCANGSTCTTVANQFSCKCLTGFTGQKCE

TDVNECDIPGHCQHGGTCLNLPGSYQCQCPQGFTGQYCDSLYVPCAPSPCVNGGTCRQTG

DFTFECNCLPGFEGSTCERNIDDCPNHRCQNGGVCVDGVNTYNCRCPPQWTGQFCTEDVD

ECLLQPNACQNGGTCANRNGGYGCVCVNGWSGDDCSENIDDCAFASCTPGSTCIDRVASF

SCMCPEGKAGLLCHLDDACISNPCHKGALCDTNPLNGQYICTCPQGYKGADCTEDVDECA

MANSNPCEHAGKCVNTDGAFHCECLKGYAGPRCEMDINECHSDPCQNDATCLDKIGGFTC

LCMPGFKGVHCELEINECQSNPCVNNGQCVDKVNRFQCLCPPGFTGPVCQIDIDDCSSTP

CLNGAKCIDHPNGYECQCATGFTGVLCEENIDNCDPDPCHHGQCQDGIDSYTCICNPGYM

GAICSDQIDECYSSPCLNDGRCIDLVNGYQCNCQPGTSGVNCEINFDDCASNPCIHGICM

DGINRYSCVCSPGFTGQRCNIDIDECASNPCRKGATCINGVNGFRCICPEGPHHPSCYSQ

VNECLSNPCIHGNCTGGLSGYKCLCDAGWVGINCEVDKNECLSNPCQNGGTCDNLVNGYR

CTCKKGFKGYNCQVNIDECASNPCLNQGTCFDDISGYTCHCVLPYTGKNCQTVLAPCSPN

PCENAAVCKESPNFESYTCLCAPGWQGQRCTIDIDECISKPCMNHGLCHNTQGSYMCECP

PGFSGMDCEEDIDDCLANPCQNGGSCMDGVNTFSCLCLPGFTGDKCQTDMNECLSEPCKN

GGTCSDYVNSYTCKCQAGFDGVHCENNINECTESSCFNGGTCVDGINSFSCLCPVGFTGS

FCLHEINECSSHPCLNEGTCVDGLGTYRCSCPLGYTGKNCQTLVNLCSRSPCKNKGTCVQ

KKAESQCLCPSGWAGAYCDVPNVSCDIAASRRGVLVEHLCQHSGVCINAGNTHYCQCPLG

YTGSYCEEQLDECASNPCQHGATCSDFIGGYRCECVPGYQGVNCEYEVDECQNQPCQNGG

TCIDLVNHFKCSCPPGTRGLLCEENIDDCARGPHCLNGGQCMDRIGGYSCRCLPGFAGER

CEGDINECLSNPCSSEGSLDCIQLTNDYLCVCRSAFTGRHCETFVDVCPQMPCLNGGTCA

VASNMPDGFICRCPPGFSGARCQSSCGQVKCRKGEQCVHTASGPRCFCPSPRDCESGCAS

SPCQHGGSCHPQRQPPYYSCQCAPPFSGSRCELYTAPPSTPPATCLSQYCADKARDGVCD

EACNSHACQWDGGDCSLTMENPWANCSSPLPCWDYINNQCDELCNTVECLFDNFECQGNS

KTCKYDKYCADHFKDNHCDQGCNSEECGWDGLDCAADQPENLAEGTLVIVVLMPPEQLLQ

DARSFLRALGTLLHTNLRIKRDSQGELMVYPYYGEKSAAMKKQRMTRRSLPGEQEQEVAG

SKVFLEIDNRQCVQDSDHCFKNTDAAAALLASHAIQGTLSYPLVSVVSESLTPERTQLLY

LLAVAVVIILFIILLGVIMAKRKRKHGSLWLPEGFTLRRDASNHKRREPVGQDAVGLKNL

SVQVSEANLIGTGTSEHWVDDEGPQPKKVKAEDEALLSEEDDPIDRRPWTQQHLEAADIR

RTPSLALTPPQAEQEVDVLDVNVRGPDGCTPLMLASLRGGSSDLSDEDEDAEDSSANIIT

DLVYQGASLQAQTDRTGEMALHLAARYSRADAAKRLLDAGADANAQDNMGRCPLHAAVAA

DAQGVFQILIRNRVTDLDARMNDGTTPLILAARLAVEGMVAELINCQADVNAVDDHGKSA

LHWAAAVNNVEATLLLLKNGANRDMQDNKEETPLFLAAREGSYEAAKILLDHFANRDITD

HMDRLPRDVARDRMHHDIVRLLDEYNVTPSPPGTVLTSALSPVICGPNRSFLSLKHTPMG

KKSRRPSAKSTMPTSLPNLAKEAKDAKGSRRKKSLSEKVQLSESSVTLSPVDSLESPHTY

VSDTTSSPMITSPGILQASPNPMLATAAPPAPVHAQHALSFSNLHEMQPLAHGASTVLPS

VSQLLSHHHIVSPGSGSAGSLSRLHPVPVPADWMNRMEVNETQYNEMFGMVLAPAEGTHP

GIAPQSRPPEGKHITTPREPLPPIVTFQLIPKGSIAQPAGAPQPQSTCPPAVAGPLPTMY

QIPEMARLPSVAFPTAMMPQQDGQVAQTILPAYHPFPASVGKYPTPPSQHSYASSNAAER

TPSHSGHLQGEHPYLTPSPESPDQWSSSSPHSASDWSDVTTSPTPGGAGGGQRGPGTHMS

EPPHNNMQVYA

>Hsa_263388

MGPGARGRRRRRRPMSPPPPPPPVRALPLLLLLAGPGAAAPPCLDGSPCANGGRCTQLPS

REAACLCPPGWVGERCQLEDPCHSGPCAGRGVCQSSVVAGTARFSCRCPRGFRGPDCSLP

DPCLSSPCAHGARCSVGPDGRFLCSCPPGYQGRSCRSDVDECRVGEPCRHGGTCLNTPGS

FRCQCPAGYTGPLCENPAVPCAPSPCRNGGTCRQSGDLTYDCACLPGFEGQNCEVNVDDC

PGHRCLNGGTCVDGVNTYNCQCPPEWTGQFCTEDVDECQLQPNACHNGGTCFNTLGGHSC

VCVNGWTGESCSQNIDDCATAVCFHGATCHDRVASFYCACPMGKTGLLCHLDDACVSNPC

HEDAICDTNPVNGRAICTCPPGFTGGACDQDVDECSIGANPCEHLGRCVNTQGSFLCQCG

RGYTGPRCETDVNECLSGPCRNQATCLDRIGQFTCICMAGFTGTYCEVDIDECQSSPCVN

GGVCKDRVNGFSCTCPSGFSGSTCQLDVDECASTPCRNGAKCVDQPDGYECRCAEGFEGT

LCDRNVDDCSPDPCHHGRCVDGIASFSCACAPGYTGTRCESQVDECRSQPCRHGGKCLDL

VDKYLCRCPSGTTGVNCEVNIDDCASNPCTFGVCRDGINRYDCVCQPGFTGPLCNVEINE

CASSPCGEGGSCVDGENGFRCLCPPGSLPPLCLPPSHPCAHEPCSHGICYDAPGGFRCVC

EPGWSGPRCSQSLARDACESQPCRAGGTCSSDGMGFHCTCPPGVQGRQCELLSPCTPNPC

EHGGRCESAPGQLPVCSCPQGWQGPRCQQDVDECAGPAPCGPHGICTNLAGSFSCTCHGG

YTGPSCDQDINDCDPNPCLNGGSCQDGVGSFSCSCLPGFAGPRCARDVDECLSNPCGPGT

CTDHVASFTCTCPPGYGGFHCEQDLPDCSPSSCFNGGTCVDGVNSFSCLCRPGYTGAHCQ

HEADPCLSRPCLHGGVCSAAHPGFRCTCLESFTGPQCQTLVDWCSRQPCQNGGRCVQTGA

YCLCPPGWSGRLCDIRSLPCREAAAQIGVRLEQLCQAGGQCVDEDSSHYCVCPEGRTGSH

CEQEVDPCLAQPCQHGGTCRGYMGGYMCECLPGYNGDNCEDDVDECASQPCQHGGSCIDL

VARYLCSCPPGTLGVLCEINEDDCGPGPPLDSGPRCLHNGTCVDLVGGFRCTCPPGYTGL

RCEADINECRSGACHAAHTRDCLQDPGGGFRCLCHAGFSGPRCQTVLSPCESQPCQHGGQ

CRPSPGPGGGLTFTCHCAQPFWGPRCERVARSCRELQCPVGVPCQQTPRGPRCACPPGLS

GPSCRSFPGSPPGASNASCAAAPCLHGGSCRPAPLAPFFRCACAQGWTGPRCEAPAAAPE

VSEEPRCPRAACQAKRGDQRCDRECNSPGCGWDGGDCSLSVGDPWRQCEALQCWRLFNNS

RCDPACSSPACLYDNFDCHAGGRERTCNPVYEKYCADHFADGRCDQGCNTEECGWDGLDC

ASEVPALLARGVLVLTVLLPPEELLRSSADFLQRLSAILRTSLRFRLDAHGQAMVFPYHR

PSPGSEPRARRELAPEVIGSVVMLEIDNRLCLQSPENDHCFPDAQSAADYLGALSAVERL

DFPYPLRDVRGEPLEPPEPSVPLLPLLVAGAVLLLVILVLGVMVARRKREHSTLWFPEGF

SLHKDVASGHKGRREPVGQDALGMKNMAKGESLMGEVATDWMDTECPEAKRLKVEEPGMG

AEEAVDCRQWTQHHLVAADIRVAPAMALTPPQGDADADGMDVNVRGPDGFTPLMLASFCG

GALEPMPTEEDEADDTSASIISDLICQGAQLGARTDRTGETALHLAARYARADAAKRLLD

AGADTNAQDHSGRTPLHTAVTADAQGVFQILIRNRSTDLDARMADGSTALILAARLAVEG

MVEELIASHADVNAVDELGKSALHWAAAVNNVEATLALLKNGANKDMQDSKEETPLFLAA

REGSYEAAKLLLDHFANREITDHLDRLPRDVAQERLHQDIVRLLDQPSGPRSPPGPHGLG

PLLCPPGAFLPGLKAAQSGSKKSRRPPGKAGLGPQGPRGRGKKLTLACPGPLADSSVTLS

PVDSLDSPRPFGGPPASPGGFPLEGPYAAATATAVSLAQLGGPGRAGLGRQPPGGCVLSL

GLLNPVAVPLDWARLPPPAPPGPSFLLPLAPGPQLLNPGTPVSPQERPPPYLAVPGHGEE

YPAAGAHSSPPKARFLRVPSEHPYLTPSPESPEHWASPSPPSLSDWSESTPSPATATGAM

ATTTGALPAQPLPLSVPSSLAQAQTQLGPQPEVTPKRQVLA

>Hsa_277541

MPPLLAPLLCLALLPALAARGPRCSQPGETCLNGGKCEAANGTEACVCGGAFVGPRCQDP

NPCLSTPCKNAGTCHVVDRRGVADYACSCALGFSGPLCLTPLDNACLTNPCRNGGTCDLL

TLTEYKCRCPPGWSGKSCQQADPCASNPCANGGQCLPFEASYICHCPPSFHGPTCRQDVN

ECGQKPGLCRHGGTCHNEVGSYRCVCRATHTGPNCERPYVPCSPSPCQNGGTCRPTGDVT

HECACLPGFTGQNCEENIDDCPGNNCKNGGACVDGVNTYNCRCPPEWTGQYCTEDVDECQ

LMPNACQNGGTCHNTHGGYNCVCVNGWTGEDCSENIDDCASAACFHGATCHDRVASFYCE

CPHGRTGLLCHLNDACISNPCNEGSNCDTNPVNGKAICTCPSGYTGPACSQDVDECSLGA

NPCEHAGKCINTLGSFECQCLQGYTGPRCEIDVNECVSNPCQNDATCLDQIGEFQCICMP

GYEGVHCEVNTDECASSPCLHNGRCLDKINEFQCECPTGFTGHLCQYDVDECASTPCKNG

AKCLDGPNTYTCVCTEGYTGTHCEVDIDECDPDPCHYGSCKDGVATFTCLCRPGYTGHHC

ETNINECSSQPCRHGGTCQDRDNAYLCFCLKGTTGPNCEINLDDCASSPCDSGTCLDKID

GYECACEPGYTGSMCNINIDECAGNPCHNGGTCEDGINGFTCRCPEGYHDPTCLSEVNEC

NSNPCVHGACRDSLNGYKCDCDPGWSGTNCDINNNECESNPCVNGGTCKDMTSGYVCTCR

EGFSGPNCQTNINECASNPCLNQGTCIDDVAGYKCNCLLPYTGATCEVVLAPCAPSPCRN

GGECRQSEDYESFSCVCPTGWQGQTCEVDINECVLSPCRHGASCQNTHGGYRCHCQAGYS

GRNCETDIDDCRPNPCHNGGSCTDGINTAFCDCLPGFRGTFCEEDINECASDPCRNGANC

TDCVDSYTCTCPAGFSGIHCENNTPDCTESSCFNGGTCVDGINSFTCLCPPGFTGSYCQH

DVNECDSQPCLHGGTCQDGCGSYRCTCPQGYTGPNCQNLVHWCDSSPCKNGGKCWQTHTQ

YRCECPSGWTGLYCDVPSVSCEVAAQRQGVDVARLCQHGGLCVDAGNTHHCRCQAGYTGS

YCEDLVDECSPSPCQNGATCTDYLGGYSCKCVAGYHGVNCSEEIDECLSHPCQNGGTCLD

LPNTYKCSCPRGTQGVHCEINVDDCNPPVDPVSRSPKCFNNGTCVDQVGGYSCTCPPGFV

GERCEGDVNECLSNPCDARGTQNCVQRVNDFHCECRAGHTGRRCESVINGCKGKPCKNGG

TCAVASNTARGFICKCPAGFEGATCENDARTCGSLRCLNGGTCISGPRSPTCLCLGPFTG

PECQFPASSPCLGGNPCYNQGTCEPTSESPFYRCLCPAKFNGLLCHILDYSFGGGAGRDI

PPPLIEEACELPECQEDAGNKVCSLQCNNHACGWDGGDCSLNFNDPWKNCTQSLQCWKYF

SDGHCDSQCNSAGCLFDGFDCQRAEGQCNPLYDQYCKDHFSDGHCDQGCNSAECEWDGLD

CAEHVPERLAAGTLVVVVLMPPEQLRNSSFHFLRELSRVLHTNVVFKRDAHGQQMIFPYY

GREEELRKHPIKRAAEGWAAPDALLGQVKASLLPGGSEGGRRRRELDPMDVRGSIVYLEI

DNRQCVQASSQCFQSATDVAAFLGALASLGSLNIPYKIEAVQSETVEPPPPAQLHFMYVA

AAAFVLLFFVGCGVLLSRKRRRQHGQLWFPEGFKVSEASKKKRREPLGEDSVGLKPLKNA

SDGALMDDNQNEWGDEDLETKKFRFEEPVVLPDLDDQTDHRQWTQQHLDAADLRMSAMAP

TPPQGEVDADCMDVNVRGPDGFTPLMIASCSGGGLETGNSEEEEDAPAVISDFIYQGASL

HNQTDRTGETALHLAARYSRSDAAKRLLEASADANIQDNMGRTPLHAAVSADAQGVFQIL

IRNRATDLDARMHDGTTPLILAARLAVEGMLEDLINSHADVNAVDDLGKSALHWAAAVNN

VDAAVVLLKNGANKDMQNNREETPLFLAAREGSYETAKVLLDHFANRDITDHMDRLPRDI

AQERMHHDIVRLLDEYNLVRSPQLHGAPLGGTPTLSPPLCSPNGYLGSLKPGVQGKKVRK

PSSKGLACGSKEAKDLKARRKKSQDGKGCLLDSSGMLSPVDSLESPHGYLSDVASPPLLP

SPFQQSPSVPLNHLPGMPDTHLGIGHLNVAAKPEMAALGGGGRLAFETGPPRLSHLPVAS

GTSTVLGSSSGGALNFTVGGSTSLNGQCEWLSRLQSGMVPNQYNPLRGSVAPGPLSTQAP

SLQHGMVGPLHSSLAASALSQMMSYQGLPSTRLATQPHLVQTQQVQPQNLQMQQQNLQPA

NIQQQQSLQPPPPPPQPHLGVSSAASGHLGRSFLSGEPSQADVQPLGPSSLAVHTILPQE

SPALPTSLPSSLVPPVTAAQFLTPPSQHSYSSPVDNTPSHQLQVPEHPFLTPSPESPDQW

SSSSPHSNVSDWSEGVSSPPTSMQSQIARIPEAFK

>Cte_P219186

MCPSKLTFGILCWFLGLTVALAIQSCSPNPCQNGVQCVDPAGSPGSHADPFCDCGDLYRG

EYCEYDNPCREALSHCLNGARCTVDDTLSGIEAVCHCALGFEGSMCELQVAESACVSSPC

QHEGRCRLDGALDRFTCDCNAGFRGELCELDDYCASQPCRNAGKCTSTSDGFRCRCLEGY

TGVTCTDDVNECTQSHAPCVNGGTCDNLHGSYRCVCPPEYTGPNCEVPYVACSPSPCQNG

GSCIVVASLQYECKCVTGFTGTNCEVNIDDCANHMCANGATCVDGIQEYACNCPEEWKGR

YCTMDIDECAASHGPCQNGGTCQNKDGGYMCICVNGWTGKDCNINIDDCAVRPCYNGGTC

HDKPGYYYCECPLGKKGNLIALGEYLRLRCHLNDACMSNPCHAGAICDTSPLDGSYICSC

PPGWTGDDCTEDPNECQESWDTPCEHGGSCVNTPGSFKCDCAVGFEGPRCEHNINECASD

PCQNDGTCLDARGHFTCVCMDVVLAVHHALTDAPLFWHRYTGVYCEENIDECEREPCKNG

GVCEDKIGKFSCHCPEGFTGSTCMVNIDDCASLPCQNDGTCIDEINGYQCRCLTGFEGSD

CELNTNDCYGIDCNQGTCRDMLGYFICQCDAGYSGQFCDSQVDECDDEPCGYGGTCRSYP

GGHECQCPPGTSGSNCEHNHNECSSNPCRNGATCEDGLDQFTCNCRAGYTGPICEEDIDE

CMSNPCLNEGRCIDQVNGFLCDCPLGYYDYICASNVNECDSSPCINDGACVDGINQYECL

CPSGYEGARCESQKDECRSNPCQHGGRCEDFLGYYICSCPPGYSGSNCQFNIDECAGNPC

VHGECIDEVADYTCSCYKPFTGKNCSVEMNPCEPNQCRNLANCVPSHNYKDFTCQCQSGF

TGRLCQHDINECISSNPCRNGATCQNTEGSYICQCLPGYEGRHCDVNPDDCIPNPCHNGG

TCIDGIGDFTCSCIQGFGGRHCKNDIDECASNPCQNGATCRSYVNSYTCECRLGFSGVNC

EVNDDDCTASSCLNKGRCIDGVNSYTCQCPAEYTGANCQYHINACDQISCLNGATCIEEA

GSHRCHCPFGFTGPSCENLVDWCNQNPCHNNAKCVPMSNQFQCICEPSNSWTGALCDVPM

VSCQAAAAGKGVPLNAICENGGKCRDIGNYHMCDCQPGFDGSYCATEINECASQPCQNGA

TCQDLIGRYHCECKPGFQGINCEFDIDECVTMPCQNGGTCHDLINAYNCSCPHGTEGFLC

EINKDDCTATSCHHSGTCVDEVGGFSCRCPPGFVGPRCEGDINECLSNPCSELGTRDCVQ

LVNDYRCDCMDGWTGRHCNEQRDYCESEPCMNNGRCENAGSGARCQCKAGFSGDLCQYTS

AHCNSNPCLHAGSCSPTEDGYECHCVPGAAGEHCEFDIVNECLSNPCDNQGTCINRVGYY

DCLCPPLWNGVTCDTFDATFTGGTGQPVTPAPTTPKPLDEQQRECIEKGCEAKANNGVCD

EECNSYACQYDSTECSYNVSVYKDCSAIQQGIHCFNLFRNGICDNACRSEGCLFDGFDCQ

PEMKRCNPFYDAYCINHYGNGYCDEGCNTEECSWDGLDCVAIQSSDSVVPGSLFVVVGIP

PDEFMKVKTSFLRGISQLFRANVLIERDESGNEKIFPFPDSRTRNKRDIKIENGSMIYLK

LDNSNCEGYCFHDTNSAVQYLAQSLKEGWESNVPILKVGETPKTINEPTGELLLIVVSVS

ACIIIVALLLGVLYTMTRKRAHGTTWFPEGFTAARRPQDGQEMKDVQQGVPSQGEEPVEE

GAIAAAPEDWSDLGTPERPSAKRFKHDDEEKIIRKDDPRQWTQIHLEATGKIPNPSILAL

TPPQGDHLDMRDVDVRGPDGKTPLMIASLSLGRCDAESSGSGETDDVADGSAGIITDLLT

QGAEINAKTDSTGETSLHLAARFARADAAKRLLDAGADPNAPDNSGRTPLHAAVASDAMG

VFQILLRNRTTDLNARMNDGSSPLILSVRLAMEEMVEDLLNAKAEVNATDKYGKTALHWA

ASTNNVESLMLLLHHQANQDAQDDKDQTPLYLAAKEGTYEAAKLLLDQFANRDITDHMDH

LPRDIAQERGHMDIVKLLDEYQAESPNLMASGYSPSAMMSTYAGHHMAKGKKKKSKASSS

SYAAAMHAKDTSTISPDISKVPPHVVQLPGPKGKSRKKKSQPPPPPRRTQQNSSRGRTSS

LSTASPSDSIESPPEGLIELPPSYEHACNAGQLMSMHQQQQQHQQQQQQHQQQQQHMSHP

AYPVSHVVVTMPSHQHMPPQHMHPSEDLNWLDSFQHQAMPQQPQQPQQPQQPQQHMMPQQ

QHPEPAVTSPQSGSGASPLSNPSPLTQTTYSSPYSNQSGTSLSSQSPLYPSSQSPQEHGP

PPSISPPKKMGLPLSPTHMQAMQQHAAAQHNKLRPSNTSPAVHHLPTMEEYAGLTCMGGM

QSTHLGPPQPADHSQLHAMSMYQYPTPPSHHSHDATPPQHLFQQPPQHQQQQQPAHQQHQ

PPHHVTDYLTPSPDSPEQWSSSSPCSQSDWSIPSPPGGQLPKQHHQQQTIKKESRGPAYL

>Aca_524905578

MAPGTHCEQDIDDCDPNPCHNEGVCTDKVNDFHCTCPLGYNDKRCMSDVDECESNPCVNG

GRCIDGYNSVHSPYISLRSTGTNCEININECASNPCLHGNCVDLVNGYKCHCEQPYSGKN

CDVKMDPCRPNPCRNQAQCVPQNNYQNLYKCNCPVGYTGQWMGGPCMNGGTCIDGVASYN

CSCVSGFGGRHCQNDIDECASNPCTNGATCEDYVNSYTCTCRPGYSGMHCQVNDNDCTKS

DIRSITCKAAAALKKVLVSDLCENGGTCFDAGNTHTCECRHGYEGSYCEKEIDECASGPC

QNGATCSDRIGKYTCQCAKGFQGLNCELDVDYCANQPCDNGGTCHDLVDNFHCSCPPGTE

GLLCEINHNDCLINSCHNGGTCVDEVGGYQCVCPPGFVGKHCEGDVNECLSNPCDPDGAQ

DCVQLNNSFRCDCRPGWTGHRCSMRIRCDQDPCLNGGSCRDTIQGAVCSCLVGFGGEFCG

VATSSCDPDPCLNGGVCGETATGFECMCGPGTDGVLCEIDLYDDCQVEVCLNDGVCIDKI

GYYECNCPRNWNGVRCELFDKEFLGGIGRPVSPSKETVPAICKQYNCPEKADNGQCDEEC

NLMECNHDNTECSYGTMPWQNCSQMVNGLYCWQVFRDGKCNSECNSPACLYDGFDCQQPL

GKCNPNYDTYCLMHYNNGHCDKGCNTAECDWDGLDCDEGNEALVSGTLFIIVLIEPEEFH

KVKQEFVRQLGHLLRVGVRIKLDVRDNEEMIYPWSGEVADTDESIVSRTRRWVEGVLDGG

HEAALSRHRRAVRNGDKLDKMDANDNHTSPPASNECYHEDDDEGLQIKRARVNGLTPLML

ASIRGGGLGEDDDSNSGSGSAEGGDSDNNTSVDVISSLLMQGAAINAQTERTGETSLHLA

ARYARADAAKVLLDAGAEPNAEDSTGRTPLHTAVAADAQGVFQILLRNRSTNLNARMHCG

GTPLILACRLAIEDTVEELISADADIEATDNNGGWLEETPLFLASREGGYETVKVLLDHY

ANRDMTDHMDRLPRDIAFERRHHDIVELLDSYKVASPASVHLGGGVTSPNHHHHHAAMAG

AAGYMHPGKQSKSKSRKSKNTAVKDTGSVSSPPNGMPGGLPATANGKSKSRKKKADGGDS

KKSQQQQQQQQQQLQQQQQLPSSVSSQSQAMLHHSSSDSSPMSTISPGSYSIDSHHSPPG

YETSPPRYENSGGLVLPPGVGGSDRGGSALGGRHHLEDFQVMAGHYSSPALMEHSSVDSM

LSDWSLFKMQQQQQQQQQQQQQFQQKQQPQQQLAAGCAGGLANGHLGSASGNSPTHVVGP

GGGATVLGSVNHGGPAPPMKVKNLPMSPTHIQALQQHAQQQRAAHGSPHHRGNDFPNSFH

LDGSGSQLPPSAAAPPPPSVAQIYPLQHKMVQQQQQQQHHQQQQQSLQQQRAQQQQQQQQ

QQQQQQQQQQQQQQQQQQSYPQTFDQFPTPPSHNSQHITDSPPQLHSTISSFRQDHLPTP

SPDSPGQWSSSSPHSAHSDWSEGISSPVPPITHGPNPHRNKRMAEHAYF

>Lgi_156225

MYWTTGKKVLRRRCTNENIGEYCQDANPCRGSYCMNNGDCRVVESGGGQTKAVCSCKLGF

SGMMCEFLDPNSACYRNRCQNGGTCITGDSLSDFSCKCMPGYRGTLCESIDYCASMPCRN

GGVCSSGSDGYQCQCPIGYRGRTCMTDIDECAENPGICRNGGRCQNRYGSYEKKVCFVHE

TLLEPVHFELCLFTTPQQTGFRGKNCEEDIDYCINNTCNNGSTCIDGIKSYTCKCPPTMT

GDLCQIDVDECETTPFICQNGGTCMNHNDGFMCICVNGWTGSDCSENIDDCKNNPCFNGG

NCEDRVGYFYCKCPRGKTGMRCHLDDACESNPCNAGASCETSPIDGSPICTCKRGWTGDT

CSRDVDECTESADTPCEHGGTCINTPGSYKCDCAVGFTGPWCEVNINECSSNPCINDGTC

LDEKGAYRCLCMPGFTGRNCEVEYDECGSNPCINGGVCQDLVNKFKCTCPAGFEGPTCAV

NIDECETFPCLNGGTCNDQINTYTCTCKTGYSGVHCQENVDNCRKVDCGHGTCVDGLDTY

TCSCLPGYTGLLCQYDIDECSSNPCKFGGTCSNLDNSYECRCPYGTSGQNCEKNYNECVS

NPCHNGATCIDGINRYNCKCKPGYEGTHCELDIDDCASQPCHNGGKCTDLINKFKCECPA

GYYDAECRSNVNECGSNPCMNYGTCVDGVNKFECLCLDGYGGHRCQNDINDCVSNPCQHG

GTCNDHLNSYSCDCKPGFEGKNCDKNINECASNPCVHGECVDLANEYHCNCNPPYSGKNC

EQELNPCHPSPCQNHAQCVPTNNYKDFECRCPTGFTGKLCNQDVDECASGSPCRNGAQCV

NKNGSFKCICLRGYEGKRCEVNHDDCAPNPCINGGTCVDEIGGYSCRCVDGFGGQHCQND

IDECASNPCMHGSTCTDYVNSYTCTCRLGFSGVHCEVNDNDCTKSSCLNDGKCIDGVNNF

TCQCKGGFTGSICQTRINPCDTNPCMNAGTCFNHNGNYYCNCPYGFQGTHCERVQDWCKN

NPCQNSGTCEQFANNFRCTCTSDWTGEKCDIRRVPCNSAAALKGVDVSKLCRNGGICKNL

GGTTHECICQAGYEGSYCEREINECASAPCKNGATCKDLVNHYTCQCMTGFQGQNCEYNI

DDCANQPCANNGVCHDLVDDFKCSCPAGTLGLLCEINSNDCFDNACYHGGTCVDKIGHYE

CKCPPGFAGPRCEGDVNECSSNPCSALGTQDCVQLDNDYRCQCKAGFSGRLCENRIQCSE

APCKNGGTCTNTDSGPKCSCLKGYSGDYCEFQSADCDSSPCQNDGTCRPTQDGYFCLCPA

GASGINCEIDEMDECLSSPCKNDGTCHDKIGYYECYCPRYYTGIHCQNYDVNAVPGIGRP

VTMKPSLNLNPDKELCEQYGCEAKANNGKCDNECNMHACDYDNKECSYGMKPWKECSGMR

DGVFCWKVFKDGTCNPQCNNEECLYDGFDCQTKLKECNPIYDSYCKKYYGNGHCDQGCDT

EECEWDGLDCDKSPERLAEGTLVIIVLVEPEAFRNMSKEFLRKLGHLLRAVVRVLKDKNG

SPMIYPWQQKEGDDVSDVIRVKRWAESAFGWDTSVREKRALLQGTKVYLEIDNRGCYKYS

TDECFDNTDNAAQFVAAALQRNDADLGMPVGKIESLGKEPTVDSPAITFVYIVVACGAIL

LITLVVIIVISRKRERAVTWFPEGFFNKRGSEKAGVRNRRGPDGEEMKDVQKGSHHSQLD

KMDDRHGDWGEDEEEHPKAKRQRIDEQGDSSDQSLVNFEIQDSRQWTQKHMEAANVTNPS

ILTLTPPQGDDPIDTKDINARGPGGMTPLMLASLRGNTLDTSCDDESGSAEGNEEDNRAA

DVINNLLFQGASINLQTDRTGETSLHLAARYARADAAKVLLDAGADCNALDSTGRTPLHT

AVAADAQGVFQILLRNRSTNLNAKMYDGTTPLILAARLAIEDMVEDLINADADINAFDNN

GKTALHWSAAVNNVEATESLLERNANRDAQDNKDETPLFLAAREGSFEAAKALLNHYANR

EITDHLECLPREVADERHHHDIVQLLDTYKVNSPGGMHLGNGGVPSPSDLPFMQHIHMKQ

KPKARKNNKSAPKENGPISPVNGKTKSKPRKKKTVQNTQNNNIATMQGMIDPSLGVISPV

TSLESQSPQGYDLTPPRYENPMVGLQQANLHVNDMALMSEHYKNGGIHVDNSYWMDNTYG

QQQIPPTSTTPPSQSGGSPLNHGIQSPNKLKNLPTSPTHIQAMQHAAQLNRGVPATHNVG

RQNDYAYLNGGENIPITNLVYTQPVDIRHKMSQQQPQQIQYLDQFPTPPSHHSDSTPPQH

QMATFLPDHFPTPSPDSPGQWSSSSPHSAHSDWSEGISSPVQPATQTHGKRCKCKRCTDA

VYI

>sko_585675458

MWHHWSLKLVLLVFIARPTPSQGGSCTSLNPCQNGALCDDSGGIITCSCVVGFAGEYCDY

QDPCVNANNPNYTPCQNGGTCQVVVSTNGVNFECICPIGFSATLCESPEANICEIEQPCN

NGGACILITLQDYICQCAEGYIGDTCDEPDHCYQSLSPCSNGATCTSTQNGFSCECTTGF

TGSSCDININECILYPGLCQHGGTCQDLTGSYECLCTLEYRGRHCEHVYVPCEPSECENG

GTCHVTGLLSYECRCPSGFSGENCEVNIDDCHNHQCMNGATCIDGLNDYTCDCPSTFTGQ

YCTEDVDECLEQPTFCKNGGTCSNSVGGFTCICVSGWIGRDCGINFDDCGSAVCYNGGTC

VDRVGSFHCDCVEGYTGLLCHLDDACVSDPCHQGALCDTNPSTGSALCTCRNGYTGSDCS

EDIDECDNDNGPCEHFGTCVNEPGTFRCECATGFTGPRCEVNINECQSSPCRNHGSCQDW

NGFFVCICMTGYTGVFCDIDIDECESSPCQNGGYCVDGVNQYYCECTTGFQGKNCEFDIN

ECASAPCRHGATCEDSINGYICRCVPGFAGIHCETDIDDCNPSPCVHGVCQDSINSYTCN

CDGGYQGNNCHIEINECASNPCVFGGTCTDLINSYSCDCVAGLTGPNCEINTNECQSNPC

QNEATCVDKVNMYSCYCTPGYRGKHCEININECSSNPCVNGGTCIDGINGFICNCPLGYY

DAFCLSDVDECSSSPCAHGGSCVDGVNEFMCVCVSGYTGERCQADYNECSSNPCQHGGTC

HNYLDGYECSCLTGYEGINCEFNIDDCAFEPCANGGQCIDLVNDFACACDPPYTGSVCED

RLDPCLNHGCQNEAVCEPTQSYQDYICSCVGGFTGIRCADDVDECVFSPCQNGAQCVNTH

GTYQCLCVRGYDGKNCENNIDDCDPDPCQNGGTCIDEVDDYSCACVPGFTQKNCMQEIDE

CMSNPCLNDATCDDYVDSFTCTCPLGFSGTYCQTNDQDCTDSSCMYGGTCIDDVNSYTCL

CPHGYTGPNCQYHVYECASNPCMNDATCIDHSGFYTCNCLSGYYGELCQTLIDWCSAVNN

PCHNGGTCTQINAQYTCTCMPGWTGLLCDVSMVSCGDAALQQHVQLRDLCRHGGTCVNDR

SSHLCNCAPGYEGSYCQFDTNECLSAPCHNGATCNEMIGSYYCDCSIGFTGTNCEINIDD

CASSPCQNGGVCHDLIDSYKCSCPSGTQGQNCEDNIDDCTIRPGEKCHHGGTCVDQVGGF

RCQCRAGYVGPRCEGDINECLSSPCDPRGTQDCVQLVNEYRCDCKLGFTGKHCALDFDFC

NPDPCQNGGNCVDGVSGYTCDCLPGFTGDECHLLESNCLPNPCQNGGSCEPTVSGYICHC

LLGVTGEQCQVDTYDECGSAPCLYGGACLDLVGYYLCECPPYQNGVQCDVYDPEFTGGIG

KPVTVSPTIAPDEEERGCTIAGCTEKHGDGVCDEECNTYKCAWDGKDCSLGLLPWENCTS

TSVRCWEVFQNDFCDEECNNEKCLFDGFDCEKQVSECNPIYDAYCTNHYANGHCDHGCNN

AECDWDGLDCDEYPPMFAEGTLILIILAPPEVIRNNSKQFLRDVGHKLRTTVVFVKDTKG

DEMILPWSEGDDLATNVAMARHRRSAYSGSSLLDIIIGRHKRAGRTGSKVFLKLDNRKCL

QEENECFATAGEAASFLGANAAQDDLDFAGFPVQGAGSTDEDIITAQLPNKLVWVLVAGV

AIVLVVVVLGVIVSTARKRKAHGTLWYPENFNKFVCKPENPRRRSRRKEPVGEDSVGMRR

MAHMPNGASGSADPNENSVWDDEGPSVEPHRKRIKVHTEQPSPSTSSHSDHERSLPADDE

IDNRLWSKSHYDAADVPIPASLALTPPQGEGEDESDQLEVDVKGPGGFTPLMLVSCRTGY

DESEMEDGSAAIITDLLAQGACPSATTERTGETSLHLAARYARADAAKTLLDAGADANAQ

DYTGRTPLHSAVAADAQGVFQILLRNRATNLDAKNFDGTTPMVLASRLAVENMVEELINA

GADLNSVDSSGKSALHWAAAVNNVESTTTLLNHGANKDIQDEKEETPLFLSAREGSFETA

KILLDHFANREITDHMDRSPRDIAHERLHDDIVKLLDEYNIVQSPPVAVSMSSNGPMTSP

GQLCGNFLPHMTGSKQYKRKGHRSKHAHNQGSSPIVPNGAHMNELSPKMMNSNKDHPPPP

PRKPMKKKRDSMETSAALSPVDSLESPPNYHETSPCMQNMYAKYTIALSHPSIASLDTIA

PINGHNVSSAPQYYKENITAAPRLSHPSPDSMCQASRNGIVLNSSGLGLDLGVIAPVSVP

SDWMSSVHRRNGHVSVPTQNRQSLAPHHSSQPNLDMSTHPPSSTAAPHTSFATSPSQMAA

FQALAGNQASQLAYAAQSEQDQPPPQYSVARPTTNVTQISNGAIPVHMICPQNNQPHHVT

TDQNNSHHSQTQLPQSQIPQHVAMAAEKYPTPPSQHSHMHPSSLENTPNHINNIMPENYL

TPSPESPGQWSSSSPHSANSDWSEGVSSPPQSSHANGHHAPPPKITTTAIYI

>Sko_291233055

MFVQLLLVAVVLISYTDAITCEQKAMDEGRAVSALCFGDLNQGVCVDDGVDYYCNCSYAV

FGSDCQDNFICLDPYNPCLNGANCLLIPDVFCFCQLGYSGTYCEIDDNQVITTVTTTTTV

VTTVASSPCDVMPCLNNGVCEEIDATTYTCTCLTGFLGTNCEVDICDPNPCQNNGNCTYL

NLSYSCDCLLGYIGNECEIPESNCLPNPCLNSGLCQPTETGYICSCVSGYNGTECEYDQR

DECSSSPCVNGGFCLDDVEMYLCDCPIYYNGLHCDIFDINFPGGIGEDVAAAPLAADQRN

CEIPECALVYGDGVCDAKCNTYNCYWDGRDCSLGTLPWENCTVDSVNCWDVFQDGKCDEE

CNINTCIHDGFDCDEPVGECKYDNYCSNYYADGLCDNGCNNAPCSWDGLDCDEYPPNYAE

GTLVMTILLEPEAIRNNSKMFLREVGQILHTIVVFVQDENGNDKIIAWTEEDEDDVIDSS

ELSKRSAYNDTVTQETLTGSKVYLKMDNRKCYQSGGSCLQTADEAASLLGARSSKSTIDL

EVLPLQNIETEADTTTTENSPSSLLWIMAVLLVIIAFPLVGVLISANRRRKAKGTLWFPD

NFVPHTSQGGKRKETSGKESVNLRSIEGTPNGVASVESCDYESHGKSPSTLSGKSHSPDS

ETDIEPSKKRIKFQEPSPSASSQDDRDQMIMIDDETDKQYWSKSHYDAAEVKVPPLLAMT

PPNDVDDADGVQFEVNAQGPGGLTPLMLASAHGCDEDMEERSAAIIVDLLAQGATTSSNT

KKTGETSLHLAARYSRSDAAKTLLMSGADANAKDLTGRTPLHAAVAADAMGVFQNMVEGL

VNAGADLNAADNGGKTALHWAAAVNNGEATSVLLKHGASKDAQDDKEETPLFLASREGSH

EAAKILLDYYGNRDITDHMDRSPRDIAEERFHHDIIKLLDEYNIVHVESPPELLSLVANG

AVVQSCQQGNYLKNDANKPTKKKMSRTKNGFRFGNSIKQNSGQSKAGMQNFPSSQQQSPP

RVLQQFGEDSPAAFSSDNSSMSSPANYPSTSPSSYHSNPASHPNIPVMDNIMVSKNSTIN

CYDYEDDNHIITAPRISHPFAPSSSSSSPPHYQGEVTMNSSLRLDMGVTAPVTVPNDWIK

SIQNNGKGSTTNDCFIDDFLETSIEDVPRFAPSLSQFAAFQALANTPRTTHPGFSNNPCD

VQYSSSNVNHMPGSSGGIPATGPSNTHLKLWD

>Pdu_Notch

MGHLELLIVCLAWILGPQSASALSCSPNPCRNGMLCIMPPGGDEAYCDCGELYAGMYCQF

DNPCRADKCMNGGTCTVDIRTSGLRAICRCPIGYNGTMCELMDPKNICATSPCGYGGTCK

LDGSLSRFKCECPPGRIGSICELEDYCASRPCRNGGDCTSLSGTYSCRCLSGYTGRNCTE

DIDECQTMTPCKHGGTCQNLFGTYRCTCPVTYTGENCETTYLPCSPSPCQNGGSCIVMGS

LSYECRCANGFHGTNCHINIDDCKNNLCKHGATCVDGIESYSCSCPPTFKGQYCDEDVDE

CSMGVNPCKNGATCQNQIGGYQCICVNGWTGKDCSENVDDCAIRPCYNGARCIDKVGYYY

CDCPPGKTGLRCHLDDACVSNPCKSGANCETSPIDGKFLCSCQPGWNGDDCNLDNNECQE

SWRSPCEHGGTCVNTPGSYRCDCPIGFDGPRCEVNINECLSNPCFNDGTCLDESGRFQCI

CMPGYQGKRCEEDVDECRDQPCLNGGVCEDKIAKFTCSCPKGFTGPTCAINVNECQSRPC

INGGTCQDEIDGYQCICQKGYYGKNCEQDIDDCQGVNCNNGGCIDLQDSFQCRCWEGYTG

KYCDSEIDECSSKPCQHGGTCTNLVNGYECQCPKGAYGPNCEHNINECASNPCRNGASCE

DGLNQYVCNCRPGFTGTLCEIDINECESNPCQNGGTCLDQINSFLCRCPRGYYDYMCASN

INECESSPCMHGGRCIDGINRFDCECPIGYEGYRCQSEENLCDNNPCQHGSTCEPGLAEY

RCACKNGYTGRDCETNIDDCNPNPCVHGNCLDHINGYECICDVAYSGVNCSKEMNPCEPN

RCQNGAHCIPENNYEDFMCQCPIGFTGRLCAQDIDECAASNPCYNGATCENTDGSYVCWC

AKGFTGRHCQTNVDDCNPDPCQNGATCHDGDADYTCHCVVGFGGRNCQQDIDECASDPCQ

NGASCHDYVNSYTCTCQLGFSGVNCDINDNDCTSSSCLNGGSCIDEVNSYRCACSAGYTG

ANCQHRINPCDSRPCLNDGSCNNQDGSFECTCRFGFTGPRCDHFVNWCTQNPCRNGGRCF

QRANQFECECPPNWTGPLCDVGMVNCQVAAMSKSVSLQELCQHGGTCHDSENSHFCRCQR

GFDGSYCEVEIDECQSAPCKNGATCDNHQGYYTCLCPDGFQGPDCEYDIDECATSPCGYG

GVCHDLVNSFVCSCPPGTMGSLCEINVNECFEGACHNNGLCKDGIGTFTCECPSGFIGPR

CEGDINECLSDPCSTPGTHSCVQLINDYRCDCYPGWGGRHCNEKVDFCNSQPCKNGGTCS

PGPSGPLCACRDGYTGDKCQYTNTVCTSAPCQNNGVCVPHGDDYTCECPPGVAGRNCQHD

VQDECVSNPCQHGGHCQNRMGYYECMCPRLWNGFNCDVYDEFFTGGLGSPVTTPPTTPAS

IDKQQKMCIDNNCAEKSGNGRCDEECNLYACKFDNHECTFYVGPWQNCSAIRQGIHCYDL

FKNGRCDKACNSKECLYDGFDCEDPVQECNPIYNEYCNSHYGNGHCDKGCDNAACGWDGL

DCTEDTPEIAVGTLVFILKIDPMEFSKIRVGFLRDVGRLLHTVLKIKQNEGMDMIYPWPD

DSYPVASELRTKRAASSKGTQVFTQLDNQKCHQECFDDTRNAAEYLAAGAQIDGLAKAYQ

VHSLYSADGPKPHAENPIWIILSSTLFIIIVFLLLGVLGTAVRRKRAHGTTWFPEGFRLP

LGNSAPKQERDGPVGEERVVQNGQIPQSLDTTSSSDNLASGIEWDDEGPDHPQSKRFKHD

PDQDDDKRQWTREHLAAACVPQPSIAALTPPQGDDQYDTSCVDVRGPGGYTPLLLASCYG

GRYGSGEDENDNDVSADFITTLLSQGASINARTDQSGETSLHLAARYARADAAKRLLDVG

SDPNAQDYSGRTPLHAAVAADAQGVFQILLRHRQTNLNMRMNDGTTALHLAARLAIEGTV

NDLIAADADVNSVDEYGKTALHWAAAVNNLEASLVLLQHGANRDAQDSKDETPLFLAAKE

GSYEVAKVLLDHFANRDITDQMDRLPRDVAVEKRHHDIVKLLDEYRVQSPGMCSAYATSP

NLMPNFMPQKQTKKGRQRKTNAMKENEAMSLHHQPNKVNLPPAKKSRKKRQGPPPSAANG

SPPKEDLTDAVTDLPPSYESATGLYQQHPAPMDIAHHISQSLQELHQDMRNVDELNTWMD

PINGHMHQMLPPVSSPSSISSPQGPGSSTSSPLCHPATSPHINEHSPSASASSMGPSPLN

NPVQSPHQHQMALSPKKSFPLSPTHLQALQQHVAIAQRAQGSPPNFMNPMNQDYNNMGQV

QQQQQQQQQHHHQPSPPQQMHTYIGEQFPTPPSQHSLPTPPSQHSLPETTPQHMGYFPGD

QTLTPSPEPEGWSSSNSPHSAHSGDWSDAVESPHQARPHPQAHPQTHYHIPPKPQPQPPR

DTAAYL

>Hro_72015

MNANATGEICEENIDDCINNKCMSNSTCVDQADGYRCRCLPGSGYTGKFCDEDVDECLKS

PCHNQGTCNNQPGSYTCSCLNGYSGRHCEVDVDDCESNPCLNGGTCRDSVGGFHCECPPH

KSVLCQYDNPCTRTMNICKHGTCVTDADTAGWLCRCDSGYQGKDCSEDVDECMMKQNPCD

NDGKCQNEYGTFSCKCKTGYTGTRCETNIDECLQVQCHNGGACLDGIGTFKCACTSGWFW

TFCILYHVFCVGCPTPPIFKPTPPHAGYTGKFCTEVVDNCPSINCNHGRCVNEEGGYRCI

CDPGFKGLADHHCLKRCLNGGKCQQQLQQPRCLCHPGYAGEFCQIEVNECSFHPCLNDAI

CFDFMDSFVCECKPGYSGQRCQTDDDDCSERSCLNGGTCVDQVNSYTCKCPVGYHGDRCQ

WEMTPCSSAPCLNDGTCVPSENVFERFCSDGFTGPRCETLKDMCAVNNGGCHQQSTCKQE

SNQIICVCSAGWTGLKCDMPAGSCQEAAIKKNVPAIALCQNGGQCRHSHRGHSCKCMPGY

EGSYCEFQVHPCSPNPCKNNGVCLDRNFFNKNLKTSKFAGGNLENKNSSSSTSGSKGSSN

NIDFDIDGFTCKCPVEYEGKLCEKPVDHCRDGPCLNGGSCYSGSAGFLCSCPVGTHGDRC

EITINACEISTTATTLTTNNHTTTTPNKCHNGGTCIPNYKGAPGYRCLCPPGFTGRNCQG

KLSGCRLKPCKNGGKCWKVRSNDVASHGNNNDGSGGNNAAQCEDNYYIIANHHQHHHQHH

QHHNHHSLTSSSPPPSSLPSPGNLYCIFNGCHWKAGDGVCDEECNNKNCLNDGGDCESTD

PFQSCNVSTCSQTFNDSVCNQECNNIDCLFDGFDCLDRDRRQTCFHEDYCSYHYADGNC

>Hro_75318

MEFVNSFACKCISGFSGTLCQTNDDDCTDSSCLFGGTCVDQVNGFKCVCAPGYTGSNCQH

RLDSCQQHPDLCLNSGVCVSVLDINSNEDRTLNQVVKSFAKCYCSKGFTGPRCEEVVDWC

ARAPCWNGATCLSFVNQFECKCRSGFSGALCDVRMVSCHAAATAKKTTVEELCQNGGTCS

DVGNTHHCACRPGFEGSYCQIETDECLSMPCRNGATCLDRVRAYECLCPAGFQGTQCEMN

IDDCENNPCQNFGTCHDMVNTFHCSCPHGTEGLLCEINVNDCHKDTCLNGGSCRDKVGGF

ECICPPGFSGPRCEGDLNECTSNPCSLHGSFDCVQLPGDYKCLCRIGWSGKQCERRVSIC

SLPKIAHMCENGGTCVESFNNSYYCMCQKGFSGDRCQYKHTQCNTNPCLNGGTCQSNKQA

FQCECPIGIGGERCQYDVVNECFSNPCQHGGTCQDRLSHYTCVCAQFWNGLNCEVFDSDF

KGGVGYMEDVVMASSKQIQHPDCAINNCSAKAGNGICDKECDYAECNYDGYDCSYKSHIY

HSCSAVRSHVVDCNKLFQNGVCDEACMDESCLYDGMDCLPEPKRTCDLLYESYCSNHYAD

GFCDQGKKTNNFLLIKYKNEVKYYSNEKI

>Cgi_23623

MVKNIHKLVCEFHRNVVVCRDILCTGKVFQLARNRITKITVTIRAPLRLTFPILFVRSLF

EWGTVSNKWKYQLLYIEGRHFAWKPKIVSKLEYPYATAPDMCCAISCKIRDFVRGEGDRC

GENYVGEFCEDDNPCRPDAHFCENNGTCRIINTADGFKGRCDCRLGYSGTLCEILEPTSV

CYHNPCQYGGTCYNTVSLQDYACSCPVGYRGVNCSKVDHCAPKPCRNQGECHALDDGYQC

NCLRGYKGDTCMEDVNECVENPDICQNGGTCDNRPGSYMCICPREYTGRHCEELYVPCQP

SPCRNGGTCIPSGGLSYQCICQSGFQGMHCETNVDDCVNSRCANGSTCVDQVNSYTCRCP

PTLTGQFCEKDVDECRLYPNICKNGATCLNHPVGNYTCICVNGWTGRDCSINIDDCKDNP

CYNGGTCHDKVGYYYCDCPHGKTGLRCHLEDACTSSPCHEGASCETSPINGDPICSCKKG

WTGNDCSMDVNECHEKNEIPDGDVNLSQNRDNDCRCVNGWNGTDCLSDIDECYSGSVSPC

EHGGTCVNTPGSFKCDCVNGFTGPRCETNINECQSNPCQNQGTCLDLSGMFRCICMPGYA

GTVCEEDIDECQSSPCQNGGVCEDLTNKFKCTCPPGFEGATCQINIDECASNPCQNGATC

KDKINAYTCSCAHGYQGDRCETNIDDCRGVTCSNGGTCRDLLGDYQCVCPEGYTGTHCET

DIDECESQPCKYGGTCHTIANAAGYECRCPRGTTGKNCEVNFDDCKDNPCINGATCEDGL

NNFVCRCKPGYTGDRCDVNVDDCNPNPCHNGGTCRDLENGFLCDCPYGYHDATCLSNVDE

CASNPCLNGGQCKDGVNKYTCSCPAGYEGIRCETKTNECASNPCQHQGVCHDLDGSYRCD

CVPGFTGVNCEINIDNCATNPCAHGSCTDLANDYKCHCEVQWTGKNCDTKLDPCNPNPCH

NSATCSASADFTDFSCYCPQGLTGRYCSEDYDECKTTQCHSEGTCVNTYGSYKCMCSRGY

TGRFCESNINDCESSPCQNGGTCYDKVANYTCICPPGFSGYHCQNDINECASNPCEHGAV

CNDYVNSYTCTCKPGYSGTNCHVNDNDCTESSCLNGGTCHDLVNSFRCECALGFEGMNCE

TRILPCDSNPCLEGGTCVNDKSLTSFHCVCPYGFTGSRCEEFHDWCVEGVSVAQLCQNGG

TCHNIGNSHNCTCHRGYQGSYCEIDVDECQSAPCQNGATCMDRIGDYLCQCKKGFEGDNC

ERNVDDCAHNPCTNGGKCHDLDDNFTCSCLPGTKGLLCELDEQDCFAGACFHGGTCVEKI

NGYECNCQPGYVGPRCEGDINECLSNPCDPLGTHSCIQMVNDYRCECNPGYRGHNCSEKI

NFCASQPCKNGGKCTSGDSKPICTCMEPYSGDLCETEITACSSGPCLNEGVCHPMGKNQF

QCICPPGVGGSICDQDILDECDKAYNPCRNGGSCRNRMGKYECICPRNYVGLNCERYDAS

NTGSPQTTAVIDICEINGCAAKAQNRICEEECNRAKCQYDGGDCSFGVPDLYEKCFDPEY

CAIKYNNSECDHRCNNQQCLYDGGDCREHKDCNPFGNFYCDRLYNNGLCDETCNVKECNW

DGSDCKGKSNAKSQVPGSMIVFVKVSPTEFLYKRALFLRQLGLLLNVLVEIKRDANNSFM

VYPWEPTAMQGRAKRYADLALEGMLNRFRRAPAPSTETNGTKVYLTLDASSCVSVNAELD

CITNLDVATQVFAVAVKNGYDTGVKVERIEMKDCMGKECDGGTTQPPGTPNMAYIYVAVS

IAAVLIMIVVVIFVLSRRKTAHGTTWFPEGFFMSNSGPKSTGPPRTAKRRVPDGEEMKNV

KGPGCDDLNTQVTSPPWDTDDDMPKAKHAKMETQSKHLEQVLSPPPSDTDQRQWTSRHLN

AANIPNDQMALTPPYDNEHTHVNDVDVRGPDGLTPLMLASYRGNGLDNGCDNESSGSGSG

ESSNSGDSDDKSVEVIQALLVQGAEINAQTDRTGETSLHLAARYARADAAKVLLDAGADC

NAEDITGRTPLHAAIAADALGVFQILLRNRSTNLNAKSQDGTTPLILAVRMAVEGMVEDL

IKSDADINMTDEWGKTALHWAAAVNNSKAAQTLLQNGANRDAQDTKASTAYLSKTDETPL

FLAAREGSSHTAQILLDHYANRDITDHMDRLPRDIAHERQHRDILRLLDEYRMSPAGMTL

SNGMPASPGHMHMMQQKNSKQKRRKNNSNTPISPNGLPNGVHPKKPKTKKKSPKHGTSPN

CEGSASSMETVSPGNSIESPLRYDQTPSSYDMYARSMSQQPVYHIDNVTLSHSMTDDQAI

LSYEYNNSPGMQPQWTQPHHNPPPTYTTSITPPSQPPINSPMGHGKMSPVKPTKNSLPTS

PTHIQAMHQRARQEQRAAHGSPHSRQNDHYVYSNSETHLPITTMPNMYTNDSYMHQSQAI

DYPQKMYIEKYPTPQSQSSMDSPQVRSGVPLPEHYLTPSPDSPGQWSSSSPHSAHSDWSE

AISSPDQPIRNKPVFL

>Tca_642937796

MWITNWKDYDLRIIVSSAVLLALANVATAFVSCSPSPCKNGGTCVSSLKGGDSYCNCTSK

YVGEYCQHLNPCHTGPGPRCQNGGSCTVRISAGGSPSFTCACPLGFSASLCEIPIANSCD

KKPCLNGGTCSLRSLKAYQCSCAPGYTGKHCEKEDHCASQPCRNGAKCTSVGDSFECTCA

PGFTGPACKDDKDECRHKPCVHGKCHNTHGSYTCTCDEGYTGQNCESEYIPCDPSPCLNG

GQCRQRDKHTYTCDCPTGFRGTNCEENIDDCPGHQCRNGATCIDGINSYTCQCPATYTGR

FCTQDVDECSLRPSVCQNGATCTNSIGGFSCICVNGWTGADCSVNIDDCAGAACFNGATC

IDRVGSFYCRCTPGKTGLLCHLDDACTSNPCHADAICDTSPINGSYTCSCASGYKGIDCS

EDIDECEQGSPCEHDGICVNTPGSFACNCTQGFTGPRCETNVNECESHPCHNDGSCLDDP

GTFRCVCMPGFTGTQCEIDIDECKDQPCLNGGVCHDLINSFKCSCAIGFTGSRCQINIDD

CVSSPCRNGGTCHDSIAGYTCECPPGFTGLSCETNINDCQSSPCQHGECLDGENSFSCTC

HPGYTGYLCQYQINECESNPCQYGGLCQDLVNGYQCLCKAGTSGPNCEINVNECYSNPCR

NNARCVDGINSYNCECIPGFTGKHCENDIDECASNPCANGGVCIDMINGFRCECPRGYYD

ARCLSDVDECNSNPCKHGGSCEDGVNQFICHCLPGYSGKQCEINIDECASNPCQHGGICH

DHLASYTCECLPGYTGINCETNIDDCAINPCKNRGTCIDQVNDYKCVCELPFTGRKCEDK

LDPCTPNRCRHNAKCTPSSNYKDFACTCSGGYTGRFCDQDVDECVVSKPCRNGATCKNTN

GSYHCICALGYEGKDCSINTDDCASHPCQNGATCLDDIGDYTCLCVNGFEGKQCDIDIDE

CLSNPCQNGATCNQYVDSYTCTCPLGFSGINCQTNDEDCTETSCMNGGTCIDGINSYTCT

CKPGFTGSNCQNRINLCDSSPCLNGATCQDHTTHYTCHCPYGYTGKDCGEYVDWCSTNPC

ENQATCVQNKNQYQCLCGVGWTGKVCDVEMVSCKDAALRKVVPLKKLCNNGTCEDIGNSH

RCHCSDGYSGSYCQTEINECDSAPCQNGATCLDLIGSYSCKCPKGFQGQNCELNVDDCKP

NPCQNGGTCHDLVNSFSCSCPSGTLGYICEINVDDCRPGACHNNGTCLDKVGGFECKCPP

GFVGPRCEGDINECLSNPCSNAGTLDCVQLVNDYHCNCKLGFMGRHCESKVNFCATSPCQ

NGGVCTTIHAGHKCTCPEGFYGKNCEFSGYDCDSNPCQNGGVCRISDGGGYICDCPSGTN

GTNCEIDTINECDSNPCKHLDAICQDKLGDYACYCPPKHTGKNCEIYDRGFPGGLGRAVV

PTLDATSYYAKDLEKQRQQCNQNKCPVKRGNGRCDEECNTYACEFDGNDCSLGINPWANC

TASTRCWEVFMDGICNEDCNNPECLFDGRDCEKSLQPCNPIYDAYCQKHYANGLCDYGCN

NAECNWDGLDCEKAPPELAEGVISMIILMDMQGFRKNLVAFLRDTSHQLRTNVRVKKDQE

GRDMIYPWLGSEPSNFDSYYTRKHHVKYTEQTQSGVIVYLEIDNRKCTITDSSDSCFQTA

SAAAEFLAAKASKHTLSQSFPIYQVNGSSGIIDDLDTPTNAKYVVLGFIIVFAFSAAIAV

IVTTQRKRASGITWFPEGWSLANTGPRRRSRRRGPEGQELRNMGKHPSVPLEIDNHGQGH

VPQWSDDDSDMPQPKRFRGLETGYASDHTGITDYEEAEPRVWSQQHLEAADVKPISMLTP

PQGDLNDVNARGPCGMTPIMVASTRGGGMDTGEDDEDDSAGAVIQDLVAQGAELNATMDK

TGETSLHLAARYARADAAKRLLDAGADANAQDNTGRTPLHAAVAADALGVFQILLRNRAT

NLNARMHEGTTPLILAARLAIEGMVQDLISAEADINAADNSGKTALHWAAAVNNVEAVNV

LLAHGANRDAQDDKDETPLFLAAREGSYQACKALLEAYANREITDHMDRLPRDVASERLH

HDIVRLLDEHVPRSPQMVNVIPNSSMMTSPSGQLQLQQPTVIVPKRQKSKRPSKNSNNNN

NASLDPTSPEMETINTGSIRRKPSVKKSKKPTPQQEIPQSVDSITSTLSPVESPMTTNLP

SPYDTASIYSNMGLTPQGLESLINKQPPSYEDVVKNNLHGLVGLENYSFNLGNFHDTMMM

QQRQLQQANTLSPPYSNQSPPHSVQSNMSLSPQGYNGSPSPAKTRPSLPTSPTHIAAMRA

ATQQKHGGVPQAQNLQMGFDFSQTGLELPMATYPGLVSGNLQFQQQNYYLTPPSQHSDLL

PDNFPTPSPESPGHWSSSSPRSCNSDWSENVASPNNTYGGGHQTNKGSDAIYI

>Nve_215245

MWSVLNIFWFLTIFVNAKAAGFYYQRLKTSEGKTCRPRLEAKLYPKYCGSHTPCENIKDC

TKPNDQCICDEDCGNICTDPTTNCTTGWCTNGGVCSEAGGVVTCSCPKNFTGARCEIDVD

ECTTLSQPCQNGGTCSNVYGGYMCRCITGWDGADCSVNIDECKQNDPFPRCQHGGTCVDK

IGSYTCICPPGKTGLVCNFDDECASNPCSANATCVTSFSGKASCICNSGWTGKNCDVDIK

ECEGDSSPCYHGGTCRDIPGSFVCDCVPGFAGARCEDNINECESNPCVHGLCLDYQNKFE

CACSKGYTGRLCDVEINECDSNPCLNGGQCHDGLGNYSCTCQVGYVGEICQTNYDDCASN

PCQNDGTCQDGIAQYTCLCPLGFTGKSCETNTNECAGNPCMNMGTCIDGINSFRCQCPLG

FTGNRCETEIDECESSPCQNGGTCKDKINGYVCICPPGASGTHCENDPNDCPANACQNGG

VCIDGMNTYSCKCHPGFTGFSCGVPVNECASSPCRNGGTCEDGVAQYYCRCRDGYTGKNC

EVRIDSCIDHTCQNGASCVSSTPYAYSCQCKPGYTGQYCETDVDECAARPCVNGACVDGV

NGFICRCDPGFTGDRCQINVDDCQSSPCVHGGSCIDSINTYTCQCPKGFTGPRCEIHINE

CSSDPCQHGGTCSDRIGSYSCYCRPGYTGSNCQHPLDRCANDPCRNGATCRRTGEDLSDF

HCECPLGYKGTICDVKEVSCAVAGTICANGGTCFDSNGVQSCTCKPGFTGSYCRTNIDEC

AKGPCKYGATCHDAVANYTCTCTAGFTGKNCDININECASNPCQRGSCLDLVNGYLCSCP

KGYIGKHCEVNADDCFVNACFNGGSCVDGIAEFKCTCPLGFSGSRCEVDVDECASSPCSA

LGTEKCINNIGAYHCQCKQGFLGRHCDLNINECLSYPCRNGGECKDGAGEYTCLCPHGFS

GDDCERRVYTCSDQPCLNSGTCRTVHNTYNCSCRPGVYGKQCEWNKDECLSAPCRNGGTC

IDGYGSYSCKCPLGFAGANCEESINECLSQPCKNGGSCRDIVNGYKCDCPSKMIGKNCET

VVKDQCPVPNCAKKFDGGKCNPKCNTHECNWDGTTCSLGIEPWSNCTTVTKSGKACYQVF

ANGVCDRECNTGGCLFDGFDCKPSVPKCGADKYCAARFANAECDAICNNVACQNDGLDCS

FKKPEIVEGTLVLVLLVVPEAFMNGSRVFMRELSRTLNTIAFIKKDSEGKELVKVYPLPP

SAPVPAERRKRSAEKIWFIDSDLTESHRFRRAAVQKNGVQVQINLDNRGCETDCFQSTEQ

AAKYLGAQQSTGKLNLPYPVYSVKTEEKPTVEPETGFQPEPLWIAILCVGVPLFIVGVLA

GGKRVYTKLWLPEGFVRRPVHQRRSLRRDPVGQEHSMRSMNKSSDLEEEGAVGGDLTPPQ

EARDAKRVKLEEVDESQRVKVSEKEKDTRQWTRLHREAADVTVRNCTALALTPPQEGESE

KPGIDTGVDARGPGGFTPLHLASCRGTLVDGCSIDDDKESDDSGGAMVSDLLALGASYGA

RTDIEKETPLHLAARHSRADAAKRLLHAGADPNARDKLGRTPLHLAVGADAQGVFQILLR

NRTTDLEAAMEDGTTPLILAARLDLLDIVKDLIKASCKVNNVDAQGKSALHWAAAVNSHE

VTSELCKNGAKKDMQDDKGQTPLFLGAREGSLEAVRILLLSYANRMIADNMDKTPEEVAR

QRAHNDIVELLSDWSIGCNSPKAAPAPTSPPDQRSPLNGTASPPSMDQISGNKVVTHFPV

SAGTTRPKTNSTSTRGARSKVQNENGAKRKRKRRKDDDLPHGNRKASQGGASSTYSPYGK

ELSPCSSGANFSPPAFSVVNGLSPQGSSATGISPSNSTTLSPQQHSSLDANSPQFPLSDS

PFDDGYLDLDIFEDLTDQDLHGDFASPACGLRGSTSDLHDSISMLPETTIGPVPQDSSVP

TTSACMYLPRHSVAPFGHTAPRLYSAHSSPNLCADNESRQLIVAHSINGYPSNQSSIQAC

KLSNRLEEHVMMETTLHHNNQSMCVPVESGMFREKHHTPPSAHSYGTSSYDSSPQKLPIS

YLTPSPESPKDWSSSPSSHSDWSNC

>Mle_128617a

MTRLDATSCSFTKLIGSPFRGRNQISVCKEYTYNRDQRNIHCQCGVGLTGKYCERTVDMC

SRNPCRGNGNCTDLGGGEFRCDCFPGYTAVNCEDDIDECKEDPCQNGGECRNTIGDFYCE

CRPGYGGNFCEKNIDNCANDPCKNGGTCNDGENSYTCTCQPGFTGENCETEINECENNTC

PSYSVCVDKINRYRCICNSADENRGNCEIDENECTANLCKNGGTCVDLPGDFRCECPEGY

SGDTCQFDDTACGVKPCRHGTCKLLDIDNHYCVCDPGYAGPVCSIDIDDCQPNPCQNKAT

CIDQLSDFFCECPAVFTGKKCDLDAKHCANNPCHNGGSCAPASSHPFYKCTCERGYTGNR

CQEEILCAPTKCGNKGKCIMLDESTDTYRCECDQDFEGPLCETELESCGLDFCHNGGTCN

SLLNRCDCAPGWKGRYCQDRLACSDRPCRNGGSCIPEPYGFQCRCPDGYTGAHCEIPEAM

VCSPNPCQNGGICSADGGTHTCKCPATYTGRNCETRDFCLDNPCKHGYVCENFPPKYKVP

MKYICCEEKSWRCYNDDDNCPNECNALKNDSICQEECYNTECQLDAADCAQNLQTPHDAW

SECPNRRCSGVFNDGECNSECNNPECNYDGMDCIRKTETCEYEKYCRTYFNNNLCDQGCN

NTACMWDGRDCLQDNSHYKAKDGIKMTLDVTCERVTDFKSQLTLTLDEWSRAITVIDSAV

PVINEENRCDLYLSFDNRYCGSDCRTANVTTLKSYLWACLQSNHSASPFPFRVMAMITSA

ESDKTTLQPEQVSTAIIATIAVIGLLLLIAAVMVGRNVYRARARLWEPDGFKTSRKRNIK

DISKMGDPVHLKTIPKLSEQHNFNYYMSENMSQVMEVDLEPMYIMSGRTHGRGVFDDQDE

TAPMITQAAYELRQRDETASPVTQAEEQVAIGRPSGRIGLGRGETQLHVVCRNKKADELK

RILAQGNVDVNVQDAFGRTPLYAAVGSDAGEIVDILLEVENIDLEKPIVQKGSTPLILAS

RMVYNSIVEKLLKARAQVNAVDSSGRTALHWAAAVCNMQAMELLLNHGANKDAESIKKET

PLFLAAREGKLEAVKFLVYHNAQRNLADSMDQTPIDVARDHLHTDIEEVLLTWNTDGNTV

LKQQHVQLPGSLTPPQSDDAATGSPNSIPTLSPCEKEASDRDEKKNSKRKKKKTEHNPQS

HQTAYNQTSYWAKNQLHSPPNWMSTQMLSPESLTGSPTSTSLPTSCDPNVPSSDSHQRLT

HYPLHAGYPIDTPQSYIPHDHLLSQHRPNNNRQYSHHPAHQLDYHSSPDSPEETWQITSP

PNSDWSHTSP

>Aqu_525314

MTLARTLFLLLACMVVYSSCTCSQTCVHGTCDSANTCICDQNWFGTNCTEFACNPNNNPC

LNGATCSRGEGNDFICHCAEGYSGTLCDGNDGRIPITLCNYTSSTSCGNFGTCIVDPYTG

NETCVCEFGRSGPNCNEVFNYCHAYPCYNGGTCVLTSGFFTCLCPPAFNLSSTCRERNPC

NGTSPCLNEGTCYSVNGANACLCPPGFSGDICNVSLSNCDPSPCVNGGMCNNTSDFTDYT

CSCPPSYSGKNCQNYTDPCDGFECLNGGVCDSSQLSDDGSTYATSCNCSAGFEGERCERD

IDECTRFSPCLHGATCINQYDGYQCSCAPGYNGTRCENNINDCSPVNPCQNGGKCTDGLN

SFTCDCSGTGFTGLTCNVSTNTTCSSLGCFFGGNCLFDSFSNRDKCSCPEGYYGELCLFK

VQTCFLTKSCSVSGTSHCSQNNDCICKTGYTGPTCNTTDPCATNQTSDPSCSCPPKLTPG

ECCSDCANGGTCLYLDGRKTCICPLGWGGPDCTLDVDECSTNPCQNGVCINTDGNYTCTC

ASGYIGRNCEEYNGSCTNSTCDNGGVCRETGEGPKCVCPHANCSSLANNSICDLHCFTPA

CGYDSSDCTFNGNRTTSIWSQCPFTDCRSSYKDGVCDQKCNNAPCLYDGNDCLFSLPSCP

QQNITRCSGLVGNGVCDLYCNTTQCPFDTVDCAKQVYLPGTLIMILFSLPSVQFDHSHIE

PFRRSLGRLINAEVEVLSYSNISKEEASMYIDNTRIDAPLSYWKVTMLINIQYCVQACPT

SIEQVIRIVRASSGSVKLDDFGIVDAIEGSPPLPTSSPTTPPNNPASATSIIVIIVSVSV

SILIVVLVVGVLGKRVRDNGGVGKVLKKVRSRGIWHVDGRDQTNNSGSNTQDTTDLDRRS

SSPECVADSFSQTDRRGYYIWQPIKVSVDGEESGSVAVIGERGKDLRRWTPLHCEAVRFS

GDNTLQDILMRDHSQLDAQGPGGFTPLMIAIVSQEKKNRHKLITVRTDSSSSSSDQSERD

ILMTNCAPSYSHPGAVDGMILPGHYPGHHPPHISPYSPVGILVSHCANVNITNDYGQTAL

HLAAKLGREDYIHILLSAKADPNIQDMWGQTALHVAIGAATPGAFKALLAYPKTSMELKS

MGGVTPLIMCVKMANHPMLQQLITKNVDIAATDNEGRTAVHWAAMINNIEALKMLIKQGP

DNIKDAPNGRGETALYLACREGATECVRYLLFECFANNTLMDMLDKSPLQIAYERQHADV

VELLKQANQASCAMPIPPPPSSYNHKVHVSSANPMFSRANTIKMEPGLPSPHIPPSYQQS

LASNLPIGMPYPLPPRNGLYPLRLLPPTTTNQGVPPMQHSDQEGGGGGPGIPRVSSIEAE

LEQLVSLAFTYPSPISHTTSPATQGISSFEQTSPGHSTPGEGVSPGLSHHQDYSPASSNS

PSVYPVSNQTTPPASHVTTPPASHLGGAGSQPLKHLPLSTGYSQDYQPNSVSSVDQLSYN

TALQSITHAHLPGGHSPPIAPMTSPDPLSLPLASSSQPNGLSPGGQGTSFTTKENHSPST

GYQCFPSPPKEVGEYCYPKPHTVDPTADIINSLTPSPEDYSSQQTSVETSSPSQYLPQHQ

STVLRHYAYDYSAATYNPAQLCEGEYHQQHLPGISVSYNAHESTV

>Isc_7213

MASVIDNQIYAPLGLYSNCPPLFVGEYCQHMNPCQAVSGKRCQNGGTCNVEMSVTSGPSF

SCTCPVGYSASMCEIAVPSACDDRPCRNGGTCSLVTLDNYTCACATGFRGKHCDKKDHCA

SQPCLNYGSCLSRADSYACVCAQGYSGPTCAVDVDECKAKPCEQGTCINTQGSFSCVCEA

GFTGQLCESRYIPCEPSPCLNGGTCRPIDSLNYQCSCPPGFTGTHCEANVDDCPGNLCQN

GATCLDGVNSYTCHCPPTYTGPYCSRDVDECSFRPTVCKNGATCTNTVGGYSCICVNGWT

GTDCSENIDDCAVAACFNGATCHDRVGSFYCQCAPGKTGLLCHLDDACASNPCHEGAICD

TSPIDGTYLCSCPNGFQGVDCTEDVDEEATQGEEEEGYKGIHCEVDIDECLPNPCLNGGI

CNDLINGFKCLCPVGFSGKKCEANEDDCSSFPCRNGGSCHDGIASYTCHCPPGFTGATCE

TNINDCQSSPCHHGVCHDGINSFSCNCHAGYTGLLCQTQINECLSSPCRHGGTCEDLVNG

YQCRCRPGTSGVDCEYNVNECFSNPCRHGAKCIDGIDSYTCDCLAGFTGIHCETNINECA

SNPCSNGGVCTDLVNGFKCDCPRGYFDARCLSDVNECASNPCQNGGTCEDEVNRFVCHCP

PGYGGHRCEQDIDECQSNPCQHGGSCHDTLNAYSCTCIDGYSGRNCETNLDDCSPNPCLN

GGSCIDLVGTFRCVCEVPFSGPTCDVELDPCSPNKCRNGAQCSPSSNYLDFACSCKLGFT

GRLCDEDIDECAVSSPCRNEATCVNVNGSYECLCTRGYEGRDCLTNTDDCASYPCQNGGT

CLDGIGEYTCLCVDGFGGEHCADDMNECASNPCQNGATCNDYVNSYACACLPGFSGTNCQ

TNDEDCTPSSCMNGGTCVDGVNNYTCQCSPGYTGSNCQYHINECDSQPCAHGATCVHHIG

YHTCHCPFGYMGPRCETFVDWCATNPCLNGASCKQSNNTYRCTCQPGWTGLLCDVSMVSC

EDAALQKGTKVADLCKHGGVCEDFGNSHHCLCPEGYEGSYCQREVNECLSNPCQNGATCH

DLLGQYACDCPEGFQGLNCEYDVNDCDPSPCRNGGTCHDLVNKFVCSCPHGTLGTLCEID

VNECFESACHHGGTCLDRVGRYECQCPPGYVGSRCEGDVNECLSSPCDPLGTLDCVQLVN

DYRCDCRPGHAGRRCELKVDPCSTDPCLNGGVCHPGPRGPTCVCQEGFWGETCANRSCGD

ASPCRNGGQCRGQSCVCPRGTSGSFCERLEELGAGGACPLGCRNGGACRKGASGRFECEC

LVGWKGLRCEEYDSSFRGALAPSVEGVYDLFLKSLEEEKKLCAARRCEKKAGNRYCDEEC

NTYACNFDGGDCSLGINPWMNCTAAIKCWDVFRDGKCNVECNNIDCLFDGFDCQKDLQPC

NEHYDTYCMSNYGNGYCDYGCNNEECNWDGLDCEMDPPYLAEGSIVIMISIEPEVFRNNT

VPFLRDIGHALRTNVLFKRDPTGSPMIYPWRATDGQKPNHAALPVPRTGTLVHLEIDNRR

CLKSQGSECFQSAAKAAEFLAAAQARHSLETPFHIQEVQGAGREQGGDGAGDGGRASALH

VVIGLIGVLLAGLLVGVLFTTQKKRARGITWFPEGFLRTGSGQRRHSRRRGPDGQEMRAA

HANGEGPPEAWSDDEGERPPAKRARGSPDSSCGDTVVSDCEEERDPRPWTQQHLEAADVH

HPELLLALTPPQGNDLDQHNVDVRGPGGLTPLMLASFRGNGLDTGEDVGEDDDGSASMIQ

DLLMQGAQLASTTEKTGETPLHLAARYARADAAKRLLDAGAEANAQDSSGRTPLHAAIAA

DAVGVFQILLRNRATNLNAKMNNGMTPLILSIRLAMEGMAEDLINAEADVNAADCDARTP

LHWAAAVNDVTSVRILLAHGANRDAQDIHEETPLFLAAREGSLNAVRALLDHAANRDITN

HMDRRPRDVARDRMHLDIVDLLDKYVPSSQIPGLGSPPMLLSNPTVIGSTKSAKGKRRQQ

PKNNGNASIALQHLDASATSPGAEVNHVKRRPSIKKRREPPMPLPEVPHPLDSPHESPGG

PLFLQQGGVATLTKQPPCYEDCVGPVYTAVYDPQAYANAPMAQQQQHPRQNSVPSMGGYT

MASSPAKQRPSLPTSPTHMAAMRAAHQHKLQQGAPTYDYPVQPAYYHYPTPPSQHSHGGG

PEATPQHYLHPAETYLTPSPESPGQWSSSSPHSAQSDWSEGISSPLGQQALGGCELKQQQ

QQQTVQPAGLTHPDPVFI

**Notch alignment**

>Pdu_Notchb

DKQQKMCIDNNCAEKSGNGRCDEECNLYACKFDNHECTFYVGPWQNCSRQGIHCYDLFKN

GRCDKACNSKECLYDGFDCEDPVQECNPIYNEYCNSHYGNGHCDKGCDNAACGWDGLDCT

EDTPEIAVGTLVFILKIDPMEFSKIRVGFLRDVGRLLHTVLKIKQN-EGMDMIYPWPDDS

YPVASELRTKRAAKGTQVFTQLDNQKCHQE---CFDDTRNAAEYLAAGAQIDGLAKAYQV

HSLYSADGPKPHAENPIWIILSSTLFIIIVFLLLGVLGTAVRRKRAHGTTWFPEGFR-NS

APKQERDGPVGEERVVQNGQWDDEG-PDHPQSKRFKHDPDQDDDKRQWTREHLAAACVPQ

PSIAALTPPQGDDQYDTSC--VDVRGPGGYTPLLLASCYGGRYGSGEDENDNDVSDFITT

LLSQGASINARTDQSGETSLHLAARYARADAAKRLLDVG-SDPNAQDYSGRTPLHAAVAA

DAQGVFQILLRHRQTNLNMRM-NDGTTALHLAARLAIEGTVNDLIAADADVNSVDEYGKT

ALHWAAAVNNLEASLVLLQHGANRDAQDSKDETPLFLAAKEGSYEVAKVLLDHFANRDIT

DQMDRLPRDVAVEKRHHDIVKLLDEYRVQSPGMCSAYATSPNLMPNFMPQKQTKKGRQRK

TNAMKENEAMSLHHFPTPPSQHLTPSPEPEG-WSSSNPHSAHGDWSDAVESAAYL

>sko_585675

DEEERGCTIAGCTEKHGDGVCDEECNTYKCAWDGKDCSLGLLPWENCTSTSVRCWEVFQN

DFCDEECNNEKCLFDGFDCEKQVSECNPIYDAYCTNHYANGHCDHGCNNAECDWDGLDCD

EYPPMFAEGTLILIILAPPEVIRNNSKQFLRDVGHKLRTTVVFVKDTKGDEMILPWSEGD

DLATNVAMARHRRTGSKVFLKLDNRKCLQEENECFATAGEAASFLGANAAQDDLDAGFPV

QGAGSTIITAQLPNKLVWVLVAGVA-IVLVVVVLGVIVSTARKRKAHGTLWYPENFN-NP

RRRSRRKEPVGEDSVGMRRMWDDEGPSVEPHRKRIKVPADDEIDNRLWSKSHYDAADVPI

PASLALTPPQGEGEDESDQLEVDVKGPGGFTPLMLVSCRTGYDE---SEMEDGSAAIITD

LLAQGACPSATTERTGETSLHLAARYARADAAKTLLDAG-ADANAQDYTGRTPLHSAVAA

DAQGVFQILLRNRATNLDAKN-FDGTTPMVLASRLAVENMVEELINAGADLNSVDSSGKS

ALHWAAAVNNVESTTTLLNHGANKDIQDEKEETPLFLSAREGSFETAKILLDHFANREIT

DHMDRSPRDIAHERLHDDIVKLLDEYNMTSPGQLCGNFLPHMTGSKQYKRKGHRSKHAHN

QGSSPIVPNGAHMNYPTPPSQHLTPSPESPGQWSSSSPHSANSDWSEGVSSAIYI

>Cte_P21918

DEQQRECIEKGCEAKANNGVCDEECNSYACQYDSTECSYNVSVYKDCSQQGIHCFNLFRN

GICDNACRSEGCLFDGFDCQPEMKRCNPFYDAYCINHYGNGYCDEGCNTEECSWDGLDCI

QSSDSVVPGSLFVVVGIPPDEFMKVKTSFLRGISQLFRANVLIERDESGNEKIFPFPDSR

TRNKRDIKIE---NGSMIYLKLDNSNCEGY---CFHDTNSAVQYLAQSLKEGWES-NVPI

LKVGETKTINEPTGELLLIVVSVSACIIIVALLLGV-LYTMTRKRAHGTTWFPEGFT---

---AARRPQDGQEMKDVQQGWSDLGTPERPSAKRFKHRKDDP---RQWTQIHLEATGKIP

NPSLALTPPQ---GDHLDMRDVDVRGPDGKTPLMIASLRCDAESSGSDDVADGSAGIITD

LLTQGAEINAKTDSTGETSLHLAARFARADAAKRLLDAG-ADPNAPDNSGRTPLHAAVAS

DAMGVFQILLRNRTTDLNARM-NDGSSPLILSVRLAMEEMVEDLLNAKAEVNATDKYGKT

ALHWAASTNNVESLMLLLHHQANQDAQDDKDQTPLYLAAKEGTYEAAKLLLDQFANRDIT

DHMDHLPRDIAQERGHMDIVKLLDEYQAESPNLMASGYSPSAMMSTYAGHHMAKGKKKKS

KASSSSYAAAMHAKYPTPPSHHLTPSPDSPEQWSSSSPCSQ-SDWSIPSP-PAYL

>Tca_642937

EKQRQQCNQNKCPVKRGNGRCDEECNTYACEFDGNDCSLGINPWANC-TASTRCWEVFMD

GICNEDCNNPECLFDGRDCEKSLQPCNPIYDAYCQKHYANGLCDYGCNNAECNWDGLDCE

KAPPELAEGVISMIILMDMQGFRKNLVAFLRDTSHQLRTNVRVKKDQEGRDMIYPWLGSE

PSNFDSYYTRKHHSGVIVYLEIDNRKCTITDSSCFQTASAAAEFLAAKASKHTLSQSFPI

YQVNGSIDDLDTPTNAKYVVLGF--IIVFAFSAAIAVIVTTQRKRASGITWFPEGWS-GP

RRRSRRRGPEGQELRNMGKHWSDDD-SDMPQPKRFRGTDYEEAEPRVWSQQHLEAADVKP

I--SMLTPPQ------GDLNDVNARGPCGMTPIMVASTRGGGMDTG-EDDEDDSAAVIQD

LVAQGAELNATMDKTGETSLHLAARYARADAAKRLLDAG-ADANAQDNTGRTPLHAAVAA

DALGVFQILLRNRATNLNARM-HEGTTPLILAARLAIEGMVQDLISAEADINAADNSGKT

ALHWAAAVNNVEAVNVLLAHGANRDAQDDKDETPLFLAAREGSYQACKALLEAYANREIT

DHMDRLPRDVASERLHHDIVRLLDEHVMTSPSGQLQLQQPTVIVPKRQKSKRPSKNSNNN

NNASLDPTSPEMETYLTPPSQHPTPSPESPGHWSSSSPRSCNSDWSENVASAIYI

>Mle_128617

-NDDDNCP-NECNALKNDSICQEECYNTECQLDAADCAQNLQTWSEC---PRRCSGVFND

GECNSECNNPECNYDGMDCIRKTETCE--YEKYCRTYFNNNLCDQGCNNTACMWDGRDCQ

DNSHYKAKDGIKMTLDVTCERVT----DFKSQLTLTLD----------------EWSRAI

TVIDSAVPVINEENRCDLYLSFDNRYCGSDCRT--ANVTTLKSYLWACLQSNHSAFPFRV

MAMITSTTLQPEQVSTAIIATIAVILLLIAAVMVG-----RNVYRARARLWEPDGFK-TS

RKRNIKDISKMGDPVHLKTI-----------------KLSEQHNFNYYMSENMSQV----

---------------------MEV----DLEPMYIMSGRTHGRGVFDQRDETASPTQAEE

QVAIGRPSGRIGLGRGETQLHVVCRNKKADELKRILAQGNVDVNVQDAFGRTPLYAAVGS

DAGEIVDILLEVENIDLEKPIVQKGSTPLILASRMVYNSIVEKLLKARAQVNAVDSSGRT

ALHWAAAVCNMQAMELLLNHGANKDAESIKKETPLFLAAREGKLEAVKFLVYHNAQRNLA

DSMDQTPIDVARDHLHTDIEEVLLTWNDGNTVLKQQHVQLPGSLTPPQSDDAATGSPNSI

PTLSPCEKEASDRDYSHHPAHQYHSSPDSPEEWQITSPPN--SDWSHTSP-----

>Aqu_525314

------CPHANCSSLANNSICDLHCFTPACGYDSSDCTFNGNRWSQC---PTDCRSSYKD

GVCDQKCNNAPCLYDGNDCLFSLPSCPQQNITRCSGLVGNGVCDLYCNTTQCPFDTVDC-

--KQVYLPGTLIMILFSLPSVQFDHSHPFRRSLGRLINAEVEV--------LSYSNISKE

EASMYIDNTRIDA---KVTMLINIQYCVQA---CPTSIEQVIRIVRASSGSVKLDDFGIV

DAIEGSTPPNNPASATSIIVIIVSVSILIVVLVVGV---LGKRVRDNG------GVGKGI

WHVDGRDQTNNSGSNTQDTTFSQTDRRGYYIWQPIKVIGERGKDLRRWTPLHCEAVRFSG

DNTDILMRDHSQ---------LDAQGPGGFTPLMIAIVRHKLITVRTNCAPSYSHGAVDG

MILPGHYPGHHTNDYGQTALHLAAKLGREDYIHILLSAK-ADPNIQDMWGQTALHVAIGA

ATPGAFKALLAYPKTSMELKS-MGGVTPLIMCVKMANHPMLQQLITKNVDIAATDNEGRT

AVHWAAMINNIEALKMLIKQGPDKDAPNGRGETALYLACREGATECVRLLFECFANNTLM

DMLDKSPLQIAYERQHADVVELLKQANPPPPSSYNHKVHVSSANPMFSRANTIKMEPGLP

SPHIPPSYQQSLASFPSPPKEVSSPSQYLPQH-QSTVRHYA-YDYSAATYN----

>Nve_215245

TVVKDQCPVPNCAKKFDGGKCNPKCNTHECNWDGTTCSLGIEPWSNCTTKSKACYQVFAN

GVCDRECNTGGCLFDGFDCKPSVPKCG--ADKYCAARFANAECDAICNNVACQNDGLDCS

FKKPEIVEGTLVLVLLVVPEAFMNGSRVFMRELSRTLNTIAFIKKDSEGKELVKVYPLPP

SAPVPAERRKRSANGVQVQINLDNRGCETD---CFQSTEQAAKYLGAQQSTGKLNLPYPV

YSVKTEPTVEPETGFQPEPLWIAILCVGVPLFIVGV---LAGGKRVYTKLWLPEGFV-VH

QRRSLRRDPVGQE-HSMRSM---TPPQEARDAKRVKLVSEKEKDTRQWTRLHREAADVTV

RNCLALTPPQ-EGESEKPGIDVDARGPGGFTPLHLASCRGTLVDGCSDKESDDSGAMVSD

LLALGASYGARTDIEKETPLHLAARHSRADAAKRLLHAG-ADPNARDKLGRTPLHLAVGA

DAQGVFQILLRNRTTDLEAAM-EDGTTPLILAARLDLLDIVKDLIKASCKVNNVDAQGKS

ALHWAAAVNSHEVTSELCKNGAKKDMQDDKGQTPLFLGAREGSLEAVRILLLSYANRMIA

DNMDKTPEEVARQRAHNDIVELLSDWSCNSPKAAPAPTSPPDQRSPLNGTASPPSMDQIS

GNKVVTHFPVSAGTHHTPPSAHLTPSPESPKDWSSSPSH---SDWSNC-------

>Hsa_364163

----------GCEGRSGDGACDAGCSGPGGNWDGGDCSLGVPDWKGC-PSHSRCWLLFRD

GQCHPQCDSEECLFDGYDC-ETPPACTPAYDQYCHDHFHNGHCEKGCNTAECGWDGGDCE

DGDPEWGP-SLALLVVLSPPALDQQLFALARVLSLTLRVGLWVRKDRDGRDMVYPYPGAR

AEEKLGGTRDPTYAGFVVVMGVDLSRCGPDHPACPWDPGLLLRFLAAMAAVGALELPGPL

LAVHPHAGTAPPANQLPWPVLCSPVVILLALGALLVLQLIRRRRREHGALWLPPGFT-TQ

SAPHRRRPPLGEDSIGLKALVMCSGPEEGEEVGQAEE-TGPPSTCQLWS---LSGGCGAL

PQAAMLTPPQ---ESEMEAPDLDTRGPDGVTPLMSAVCCGEVQSGTFQGAWLGCPEPWEP

LLDGGACPQAHTVGTGETPLHLAARFSRPTAARRLLEAG-ANPNQPDRAGRTPLHAAVAA

DAREVCQLLLRSRQTAVDART-EDGTTPLMLAARLAVEDLVEELIAAQADVGARDKWGKT

ALHWAAAVNNARAARSLLQAGADKDAQDNREQTPLFLAAREGAVEVAQLLLGLGAARELR

DQAGLAPADVAHQRNHWDLLTLLE---GAGPPEARHKATPGREAGPFPRARTVSVSVPPH

GGGALPRCRTLSAGIPIPPPC-LTPSPERGSPLDCG-PPALQ-------------

>Hsa_263388

VSEEPRCPRAACQAKRGDQRCDRECNSPGCGWDGGDCSLSVGDWRQC--EALQCWRLFNN

SRCDPACSSPACLYDNFDCHGRERTCNPVYEKYCADHFADGRCDQGCNTEECGWDGLDCS

EVPALLARGVLVLTVLLPPEELLRSSADFLQRLSAILRTSLRFRLDAHGQAMVFPYHRPS

PGSEPRARRELAPIGSVVMLEIDNRLCLQSPENCFPDAQSAADYLGALSAVERLDFPYPL

RDVRGEPLEPPEPSVPLLPLLVAGAVLLLVILVLGV--MVARRKREHSTLWFPEGFS-VA

SGHKGRREPVGQDALGMKNMWMDTE---CPEAKRLKVGAEEAVDCRQWTQHHLVAADIRV

APAMALTPPQ--GDADADGMDVNVRGPDGFTPLMLASFCGGALEPMPDEADDTSASIISD

LICQGAQLGARTDRTGETALHLAARYARADAAKRLLDAG-ADTNAQDHSGRTPLHTAVTA

DAQGVFQILIRNRSTDLDARM-ADGSTALILAARLAVEGMVEELIASHADVNAVDELGKS

ALHWAAAVNNVEATLALLKNGANKDMQDSKEETPLFLAAREGSYEAAKLLLDHFANREIT

DHLDRLPRDVAQERLHQDIVRLLDQPSGLGPLLCPPGAFLPGLKAAQSGSKKSRRPPGKA

GLGPQ---------YPAAGAH-LTPSPESPEHWASPSPPSL-SDWSESTPSQVLA

>Hsa_256646

---PATCLSQYCADKARDGVCDEACNSHACQWDGGDCSLTMENWANC-SSPLPCWDYINN

-QCDELCNTVECLFDNFECQGNSKTCK--YDKYCADHFKDNHCDQGCNSEECGWDGLDCA

DQPENLAEGTLVIVVLMPPEQLLQDARSFLRALGTLLHTNLRIKRDSQGELMVYPYYGEK

SAAMKKQRMTRRSAGSKVFLEIDNRQCVQDSDHCFKNTDAAAALLASHAIQGTLS--YPL

VSVVSE--SLTPERTQLLYLLAVAVVIILFIILLGV--IMAKRKRKHGSLWLPEGFT-RD

ASNHKRREPVGQDAVGLKNLWVDDE---GPQPKKVKAEEDDPIDRRPWTQQHLEAADIRR

TPSLALTPPQ--AEQEVDVLDVNVRGPDGCTPLMLASLRGGSSDLSDEDAEDSSANIITD

LVYQGASLQAQTDRTGEMALHLAARYSRADAAKRLLDAG-ADANAQDNMGRCPLHAAVAA

DAQGVFQILIRNRVTDLDARM-NDGTTPLILAARLAVEGMVAELINCQADVNAVDDHGKS

ALHWAAAVNNVEATLLLLKNGANRDMQDNKEETPLFLAAREGSYEAAKILLDHFANRDIT

DHMDRLPRDVARDRMHHDIVRLLDEYNALSPVICGPNRSFLSLKHTPMGKKSRRPSAKST

MPTSLPNLAKEAKDYPTPPSQHLTPSPESPDQWSSSSPHSA-SDWSDVTTSQVYA

>Hsa_277541

---EEACELPECQEDAGNKVCSLQCNNHACGWDGGDCSLNFNDWKNC-TQSLQCWKYFSD

GHCDSQCNSAGCLFDGFDCQRAEGQCNPLYDQYCKDHFSDGHCDQGCNSAECEWDGLDCE

HVPERLAAGTLVVVVLMPPEQLRNSSFHFLRELSRVLHTNVVFKRDAHGQQMIFPYYG-R

EEELRKHPIKRAARGSIVYLEIDNRQCVQASSQCFQSATDVAAFLGALASLGSLNIPYKI

EAVQSETVEPPPPAQLHFMYVAAAAFVLLFFVGCGV-LLSRKRRRQHGQLWFPEGFK-SE

ASKKKRREPLGEDSVGLKPLWGDED----LETKKFRFDLDDQTDHRQWTQQHLDAADLRM

SA-MAPTPPQ--GEVDADCMDVNVRGPDGFTPLMIASCSGGGLETGNSEEEEDAPAVISD

FIYQGASLHNQTDRTGETALHLAARYSRSDAAKRLLEAS-ADANIQDNMGRTPLHAAVSA

DAQGVFQILIRNRATDLDARM-HDGTTPLILAARLAVEGMLEDLINSHADVNAVDDLGKS

ALHWAAAVNNVDAAVVLLKNGANKDMQNNREETPLFLAAREGSYETAKVLLDHFANRDIT

DHMDRLPRDIAQERMHHDIVRLLDEYNTLSPPLCSPNGYLGSLKPGVQGKKVRKPSSKGL

ACGSKEAKDLKARRFLTPPSQHLTPSPESPDQWSSSSPHSNVSDWSEGVSS----

>Isc_7213

EEEKKLCAARRCEKKAGNRYCDEECNTYACNFDGGDCSLGINPWMNC-TAAIKCWDVFRD

GKCNVECNNIDCLFDGFDCQKDLQPCNEHYDTYCMSNYGNGYCDYGCNNEECNWDGLDCE

MDPPYLAEGSIVIMISIEPEVFRNNTVPFLRDIGHALRTNVLFKRDPTGSPMIYPWRATD

GQKPNHAALPVPRTGTLVHLEIDNRRCLKSQGSCFQSAAKAAEFLAAAQARHSLETPFHI

QEVQGADGAGDGGRASALHVVIGLIGVLLAGLLVGV-LFTTQKKRARGITWFPEGFL-GQ

RRHSRRRGPDGQEMR-----WSDDE-GERPPAKRARGDCEEERDPRPWTQQHLEAADVHH

PELLALTPPQ---GNDLDQHNVDVRGPGGLTPLMLASFRGNGLDTGEGEDDDGSASMIQD

LLMQGAQLASTTEKTGETPLHLAARYARADAAKRLLDAG-AEANAQDSSGRTPLHAAIAA

DAVGVFQILLRNRATNLNAKM-NNGMTPLILSIRLAMEGMAEDLINAEADVNAADCDART

PLHWAAAVNDVTSVRILLAHGANRDAQDIHEETPLFLAAREGSLNAVRALLDHAANRDIT

NHMDRRPRDVARDRMHLDIVDLLDKY-LGSPPMLLSNPTVIGSTKSAKGKRRQQPKNNGN

ASIALQHLDASATSYPTPPSQHLTPSPESPGQWSSSSPHSAQSDWSEGISSPVFI

>Cgi_23623

-AVIDICEINGCAAKAQNRICEEECNRAKCQYDGGDCSFGVPDYEKC-FDPEYCAIKYNN

SECDHRCNNQQCLYDGGDC-REHKDCNPFGNFYCDRLYNNGLCDETCNVKECNWDGSDCK

SNAKSQVPGSMIVFVKVSPTEFLYKRALFLRQLGLLLNVLVEIKRDANNSFMVYPWEPTA

MQGRAKRYADLALNGTKVYLTLDASSCVSVNAECITNLDVATQVFAVAVKNGYDTKVERI

EMKDCMTTQPPGTPNMAYIYVAVSIVLIMIVVVIFV---LSRRKTAHGTTWFPEGFFMGP

PRTAKRRVPDGEEMKNVKGPWDTDD--DMPKAKHAKMPPPSDTDQRQWTSRHLNAANIPN

DQ-MALTPPY--DNEHTHVNDVDVRGPDGLTPLMLASYRGNGLDNGCSGDSDDKSEVIQA

LLVQGAEINAQTDRTGETSLHLAARYARADAAKVLLDAG-ADCNAEDITGRTPLHAAIAA

DALGVFQILLRNRSTNLNAKS-QDGTTPLILAVRMAVEGMVEDLIKSDADINMTDEWGKT

ALHWAAAVNNSKAAQTLLQNGANRDAQDTKDETPLFLAAREGSSHTAQILLDHYANRDIT

DHMDRLPRDIAHERQHRDILRLLDEYRPAGMTLSNGMPASPGHMHMMQQKNSKQKRRKNN

SNTPISPNGLPNGVYPTPQSQSLTPSPDSPGQWSSSSPHSAHSDWSEAISSPVFL

>Lgi_156225

NPDKELCEQYGCEAKANNGKCDNECNMHACDYDNKECSYGMKPWKECSRDGVFCWKVFKD

GTCNPQCNNEECLYDGFDCQTKLKECNPIYDSYCKKYYGNGHCDQGCDTEECEWDGLDCD

KSPERLAEGTLVIIVLVEPEAFRNMSKEFLRKLGHLLRAVVRVLKDKNGSPMIYPWQQKE

GDDVSDVIRVKRWQGTKVYLEIDNRGCYKYSTDCFDNTDNAAQFVAAALQRNDADLGMPV

GKIESLKEPTVDSPAITFVYIVVACILLITLVVIIV----ISRKRERAVTWFPEGFF-EK

AGVRNRRGPDGEEMKDVQKGWGEDE-EEHPKAKRQRIVNFEIQDSRQWTQKHMEAANVTN

PSILTLTPPQ--GDDPIDTKDINARGPGGMTPLMLASLRGNTLDTSCGNEEDNRADVINN

LLFQGASINLQTDRTGETSLHLAARYARADAAKVLLDAG-ADCNALDSTGRTPLHTAVAA

DAQGVFQILLRNRSTNLNAKM-YDGTTPLILAARLAIEDMVEDLINADADINAFDNNGKT

ALHWSAAVNNVEATESLLERNANRDAQDNKDETPLFLAAREGSFEAAKALLNHYANREIT

DHLECLPREVADERHHHDIVQLLDTYKGVPSPSDLPFMQHIHMKQKPKARKNNKSAPKEN

GPISPVNGKTKSKPFPTPPSHHPTPSPDSPGQWSSSSPHSAHSDWSEGISSAVYI

>Sko_291233

--DQRNCEIPECALVYGDGVCDAKCNTYNCYWDGRDCSLGTLPWENCTVDSVNCWDVFQD

GKCDEECNINTCIHDGFDCDEPVGECK--YDNYCSNYYADGLCDNGCNNAPCSWDGLDCD

EYPPNYAEGTLVMTILLEPEAIRNNSKMFLREVGQILHTIVVFVQDENGNDKIIAWTEED

EDDVIDSSELSKRTGSKVYLKMDNRKCYQSGGSCLQTADEAASLLGARSSKSTIDEVLPL

QNIETETENSP----SSLLWIMAVLLVIIAFPLVGVLISANRRRKAKGTLWFPDNFV-HT

SQGGKRKETSGKESVNLRSIPDSET-DIEPSKKRIKFMIDDETDKQYWSKSHYDAAEVKV

PPLLAMTPPNDVDDADGVQFEVNAQGPGGLTPLMLAS--------AHEDMEERSAAIIVD

LLAQGATTSSNTKKTGETSLHLAARYSRSDAAKTLLMSG-ADANAKDLTGRTPLHAAVAA

DAMGVFQ-------------------------------NMVEGLVNAGADLNAADNGGKT

ALHWAAAVNNGEATSVLLKHGASKDAQDDKEETPLFLASREGSHEAAKILLDYYGNRDIT

DHMDRSPRDIAEERFHHDIIKLLDEYNVVQS--CQQGNYLKNDANKPTKKKMSRTKNGFR

FGNSIKQNSGQSKA-----------MPGSSGGIPATGPSNTHKLWD---------

>Aca_524905

-TVPAICKQYNCPEKADNGQCDEECNLMECNHDNTECSYGTMPWQNCSVNGLYCWQVFRD

GKCNSECNSPACLYDGFDCQQPLGKCNPNYDTYCLMHYNNGHCDKGCNTAECDWDGLDCD

EGNEALVSGTLFIIVLIEPEEFHKVKQEFVRQLGHLLRVGVRIKLDRDNEEMIYPWSGEV

ADTDESIVSRTRR----------------------WVEGVLDGGHEAALSRHRRANGDKL

DKMDANNHTSPPASNECYH-----------------------------------------

-----------------------EDDDEGLQIKRARV-----------------------

---------------------------NGLTPLMLASIRGGGLGEDDGDSDNNTSDVISS

LLMQGAAINAQTERTGETSLHLAARYARADAAKVLLDAG-AEPNAEDSTGRTPLHTAVAA

DAQGVFQILLRNRSTNLNARM-HCGGTPLILACRLAIEDTVEELISADADIEATDNNGG-

---WL-------------------------EETPLFLASREGGYETVKVLLDHYANRDMT

DHMDRLPRDIAFERRHHDIVELLDSYKVTSPNHHHHHAAMAGAAGYMHPGKQSKSKSRKS

KNTAVKDTGSVSSPFPTPPSHNPTPSPDSPGQWSSSSPHSAHSDWSEGISSHAYF

>Hro_75318

QIQHPDCAINNCSAKAGNGICDKECDYAECNYDGYDCSYKSHIYHSCSRSHVDCNKLFQN

GVCDEACMDESCLYDGMDCLEPKRTCDLLYESYCSNHYADGFCDQGKKTN----------

---------------------------NFL------------------------------

------------------------------------------------------------

------------------------------------------------------------

------------------------------------------------------------

------------------------------------------------------------

------------------------------------------------------------

------------------------------------------------------------

------------------------------------------------------------

------------------------------------------------------------

-------------------------------------------------------

>Hro_72015

SPGNLYCIFNGCHWKAGDGVCDEECNNKNCLNDGGDCE-STDPFQSC-NV-STCSQTFND

SVCNQECNNIDCLFDGFDCLDRRQTC--FHEDYCSYHYADGNC-----------------

------------------------------------------------------------

------------------------------------------------------------

------------------------------------------------------------

------------------------------------------------------------

------------------------------------------------------------

------------------------------------------------------------

------------------------------------------------------------

------------------------------------------------------------

------------------------------------------------------------

-------------------------------------------------------

**Delta / *Platynereis* Delta-like / Jagged sequences**

>HELRO|HelroP77153/1-510 pep:novel supercontig:GCA_000326865.1:HELROscaffold_19:2875816:2885491:-1 gene:HelroG77153 transcript:HelroT77153 description:""

FQTSQPPSSSSSSSSSSSSSSSSSSSSSPPPCSPSNRCNVFFKICLTHQQPVMKLKNFPE

AGPGGYDCTFGSTVTDVLWRNNSSNMHHTVRLPISYKWTHKFALIIEAYHMDDDNNHNIQ

QGLLLKLILLKNVTSEQGWITESFHNGSTKLDYRYRLVCLHNIYGPFCSNSCVARDDSLG

HYRCLRNGTKSCLQGWTGEWC

>HELRO|leech|jgi|Helro1|190982/1-1248

MAMHFKSISFWHSKETCEFYVGTDADSYLIERAHYSGVILPRSEWQTMSFAGHTAILKYK

IRVQCDLHHFNVTCTKFCRPRNDTFGHYTCDSNGDKVCLNGWIGTNC

>CRAGI|EKC18324/1-590 pep:novel supercontig:GCA_000297895.1:scaffold43868:156077:171415:1 gene:CGI_10013656 transcript:EKC18324 description:"Delta-like protein "

TGRFEVSILAYFNESGRMAGESCCSGSRDNDTCLSLCPTFLTVCLQHHSAFIPEFPACTY

GHAVTPVFNRSLTDDVKPTKNMFFIKIPFQFSWPESFSLVIDAWQEPQLNQSAKDLSNRI

LRDVVRDKLTPPSLWVKRRTKTAQLELIYEYRVVCDEFYYSNTCEQICRHRNDNFGHYVC

DETGSKVCLPGWQGEFC

>CRAGI|EKC19039/1-787 pep:novel supercontig:GCA_000297895.1:scaffold43344:54596:61707:1 gene:CGI_10009832 transcript:EKC19039 description:"Delta-like protein "

TGIFELNLKRFENENGVNADGNCCAGVRTGARCTSWCRTFFRVCLTHYQATITDDVHCNF

AEKFTPVLTHGNNSVDFSNLPVKFDIPIKFPFQFAWPGTFTLIIEAWHDSTQQGPTTGST

RELIYRLQKQRSVEVGSSWKQFTRTENTMTLEYAYKVECGENYYGPGCVNYCRPRDDSFG

HYVCDEYGQKVCMQGWKGDYC

>CRAGI|EKC42676/1-1136 pep:novel supercontig:GCA_000297895.1:scaffold48:380506:412698:-1 gene:CGI_10023897 transcript:EKC42676 description:"Delta-like protein "

MVIRGSRGVLEINRSYTLILEAWDQDTMAYGGQLIERAAHSGIILPGQDWHTITHNGPTA

SLIYRIRVVCDDHYYNTTCTKFCRPRNDHFGHYTCDRNGDKVCMQGWMGNNC

>HOMSA|gi|110735443|ref|NP_005609.3|/1-723 delta-like protein 1 precursor [Homo sapiens]

SGVFELKLQEFVNKKGLLGNRNCCRGGAGPPPCACRTFFRVCLKHYQASVSPEPPCTYGS

AVTPVLGVDSFSLPDGGGADSAFSNPIRFPFGFTWPGTFSLIIEALHTDSPDDLATENPE

RLISRLATQRHLTVGEEWSQDLHSSGRTDLKYSYRFVCDEHYYGEGCSVFCRPRDDAFGH

FTCGERGEKVCNPGWKGPYC

>HOMSA|gi|21704277|ref|NP_002217.3|/1-1238 protein jagged-2 isoform a precursor [Homo sapiens]

MGYFELQLSALRNVNGELLSGACCDGDGRTTRAGGCGHDECDTYVRVCLKEYQAKVTPTG

PCSYGHGATPVLGGNSFYLPPAGAAGDRARARARAGGDQDPGLVVIPFQFAWPRSFTLIV

EAWDWDNDTTPNEELLIERVSHAGMINPEDRWKSLHFSGHVAHLELQIRVRCDENYYSAT

CNKFCRPRNDFFGHYTCDQYGNKACMDGWMGKEC

>HOMSA|gi|4557679|ref|NP_000205.1|/1-1218 protein jagged-1 precursor [Homo sapiens]

SGQFELEILSMQNVNGELQNGNCCGGARNPGDRKCTRDECDTYFKVCLKEYQSRVTAGGP

CSFGSGSTPVIGGNTFNLKASRGNDRNRIVLPFSFAWPRSYTLLVEAWDSSNDTVQPDSI

IEKASHSGMINPSRQWQTLKQNTGVAHFEYQIRVTCDDYYYGFGCNKFCRPRDDFFGHYA

CDQNGNKTCMEGWMGPEC

>HOMSA|gi|9506545|ref|NP_061947.1|/1-685 delta-like protein 4 precursor [Homo sapiens]

SGVFQLQLQEFINERGVLASGRPCEPGCRTFFRVCLKHFQAVVSPGPCTFGTVSTPVLGT

NSFAVRDDSSGGGRNPLQLPFNFTWPGTFSLIIEAWHAPGDDLRPEALPPDALISKIAIQ

GSLAVGQNWLLDEQTSTLTRLRYSYRVICSDNYYGDNCSRLCKKRNDHFGHYVCQPDGNL

SCLPGWTGEYC

>IXOSC|ISCW013622-PA/1-821 pep:novel scaffold:IscaW1:DS940907:305687:325744:-1 gene:ISCW013622 transcript:ISCW013622-RA description:"neurogenic locus protein delta, putative"

SGVFELQLRAFSNLLSQDSRGSCCSTGLPPSPGAPCPGTCRTWFRVCLKHYQKTVDTNSP

CTYGEVQTPVVASSGPGSTLNLTAPVRFSFDFSWPGTFSLIVEAWHENSNGGEGEAAFAA

GNSVGELVCLGGSERACVVWRRRQQPSDPGVASDDAALAQRGPGLDLGYFAVGADVAVFS

FRVVCEEHYFGADCARLCRPRDDKFGHYACNAKGDVVCLPGWRGDYC

>A.cali_gi|524885246|ref|XP_005099232.1| PREDICTED: delta-like protein D-like

HATPEPPGSSAPDVPRSRGPQWRCLFGYTDHLWPISCVPSVGQCGPSRRLVNTSVFRPKV

KYEEREADEAAFAHHSRKMAQFLGPQTLIVPFLCFCSQGDFSLIIEAWHDTSLDSPNQEY

RSTLISRLAEIRSAASGPDWYTYVHTFNYTELKYAFRFTCDSNYFGAKCADLCRARDDKF

GHYSCAENGTKVCLDGWDGEYC

>jgi|Lotgi1|71205|gw1.30.148.1

SGYFHLQVNSVSNPRGEIADGSCCNGGRHDYRGTCSEKCNTFFRVCLKEYQERVTTTGTC

TFGNQSTVALGGNSFTYRSGNSRAMLKLPFEFAWTRSYTLMVEAWDHSTNNGMDSLIERA

AQTGMILPGQDWHTIIHTGPTASLVYKIQVVCDEHYYNTTCTKFCRPHNDRFGHYTCDSN

GDKVCLHGWMGQEC

>jgi|Lotgi1|224870|estExt_Genewise1Plus.C_sca_1270022

SGVFEVKIRSLFNKHGRTVSGRCCAGPEVDDACTATCRTFFTLCLLHYQKPVPVYPACTF

GETKTQIVGANNIGIGIGAEPAFSVAIPFSFSWPGNFAFILDAWHDQHRNKSTSDTRHII

LRASRAKRLKPSPLWVEDERLTRTSRLEFSYRVTCDENYHGLGCDKWCKERDDQFGHYRC

TQGGVKVCSQGWQGELC

>jgi|Lotgi1|234999|estExt_fgenesh2_pg.C_sca_570141

LGTFELKLTSFLNAHGLNSDGNCCNGLRTAGVCSSSCKTFFRVCLTHYQSEISNNPECTF

GSKTTSVLGNNTIQFNKDTASQEFKNPVQFEFQFSWPGSFSLIIEAWHDSTLHGPESGSP

RELIARVAVQRSAEVGKDWYTFKHDTPYSEINYSYRIICGEYYYGAGCSEFCRPRDDQFG

HYKCSTNGTKICLDGWSGDMC

>gi|315660402|gb|ADU54207.1| Delta2 [Amphimedon queenslandica]

DYELLFRFRRYSNPTDRDNNDNCCDNLLICVSHCDTLFRSLCLRNRDSNSEPQCIPGTVY

DPGTVGDYNGPDNSITFGESVGSISNPIIYERPGSIPESGFQLYMEVFDYDSRNDNDYID

SIVLNIPAQPTTERQSTVVGEDGKISLELSYSLSCSQNYYGSDCSQQCIPRNDNTNGHYT

CNTTTGGIICREGWQNITTNC

>gi|315660404|gb|ADU54208.1| Delta3 [Amphimedon queenslandica]

RYTLLVKAVQYHNPDNQDWNDGCCDWPCSNNCDLYFKFCIRNKGLSASNINNCWDSVQTY

GDVSTNNYYFPNYGELYPGARIWNDLTFNRNEPWPGSVQVLVESLDADDNADDLIDRNAF

NLDLSPNGQWSNELYANGYYDRAQFKIRVRLFCQQNYYGSNCNVYCVQQNDDTNGHYTCG

SDGAKICNNGYTNPSGNC

>gi|315660408|gb|ADU54210.1| Delta5 [Amphimedon queenslandica]

DFTLSFQFHRYSNPSDRDVNGNCCDTAPGVICGSCDTHFKSLCLRNGGTSHSQTGQCIPG

TVLSPGGVGGSSVNFSAHIGVISNPFGYNKSGNILQSGFQLYLEVWDDDYAFNDDLIDRI

VFDILNRTSSQISHTAIGVFNRVSLLSSYRLLCSVNYYGFDCSVLCIPYNDDTNGHYTCN

STTGAKKCREGWQNVTNNC

>gi|340376769|ref|XP_003386904.1| PREDICTED: protein jagged-2-like [Amphimedon queenslandica]

DFGLQFQFYRYSNPSDRNFNGNCCDFWCDSCDTLFKSLCLRNGGTSHSETGHCIQSTVAN

PGRIGGSSTSVNFRGRIGSTPNPFDYTRSGSIPQAGFQLYLEVWDDDTFSSDDLIDRIVL

DVPIHPLSGVSRTVSGVFNRVSLVLSYTLSCRLNYYGSDCSVLCIPYNDDTNGHYTCNST

TGARICRDGWQNVDNYC

>SB_16910

AGNFEVQFVSIQNVAGELRNGKCCDGSRTIYGSRSHCLDACETFFTICLKPSDGQAGSNS

GTCTFGQYTTKVLGGNSFTVTDSSEQIVSLRFTFSWLKAKWLSFFEPPLLYLATMTLKPQ

IINPKYMRSDRSLATTYLLVLEARDSDNTTSQLIDETSLRGILFPNKTWESHSYNGPTAS

IKYNVRVVCDEHYYGRTCTILCKPRDDIFGHYTCDEQGHKICLPGWRGDHC

>Pdu_delta

SGVFELQLLSFMNEKGLNADGNCCHGYRTGSGCSESCKTFFKICLKHYQANISPGPPCTF

GSITTGVVGDNTFEFPDPHPSFSNPISFPFDFAWPGTFSLIIEAWNSETPNGPTQDSPRE

LITRLATQKSLNVGDPWENYTHHTNSSELRMKYRVRCETDYYGRGCTELCRSRDDRFGHY

TCSRNGSKVCLDGWTGSYC

>Pdu_jagged

SGQFQMQLVSLQNVKGELASGYCCRGETRTKQGLCLAPCRTFIRFCLREYQTMLRNLSAT

SCAFGNATSRVLGGNVITSRTSEEIFVIPFSFAWTRAFTLVFEILHMADGEEEQVIARGS

HSGVVLPSPDWHLMTFSASSGVSMTYSLRVSCDSNYYNTTCTKLCRPRHDAFGHYRCDSN

GDKVCLQGWMGTNC

>S.kow_gi|585706379|ref|XP_002740519.2| PREDICTED: protein jagged-1, partial

SGYFELQILSVWNAAGELENGDCCDGVPDPDGSCTQDSCDTQVSVCLKEFQSSVTRDGPC

TFGKAHSQILGGNSFTLPAEDAAARLKLSFEFRWMTFCTLILKILDNDNTSTSAREELID

TATHSGSLKPGSGWQTLNSPGTVAQVAYKIRIMCDEYYYSTDCMTFCKPRDDDFGHYTCD

SNGNKLCNTGWSGTNC

>S.kow_gi|259013422|ref|NP_001158418.1| delta protein precursor

SGIFQLRLASFSNDQGRNVSGQCCSVSSTSSDTCGSPCRTFFRVCLKHYQAHVSTDDDCI

YGEVETPILGNNTFEIPLSDMETFINPIRLPFTFRWPGTFSLVIEAFHSVEVGPNGFPYP

GSPRSLIARLATQRFAAVGFEWNTEVYSSEDTDSELHYSYRIVCDEHYFGEECSDFCRPR

DDNLGHYTCDAFGNKVCLPGWGGDSV

>TRICA|gi|189239617|ref|XP_969486.2|/1-1303 PREDICTED: serrate [Tribolium castaneum]

SGFFELQVLEMANPRGELSTGECCGGGARSPIMGRCSTPCNTFFRLCLKEYQSNVTSTGS

CSFGNTSSVVLGRDSFTLADPERGKLVLPFTFRWTRSFTLILQAVDHNNFTLPATNDIIE

EATYSGIIDPSPEWHTLNHRGPKATLTYRVRVKCDSHYYNATCTKFCRPRDDKFGHYICD

ANGDKECIEGWKGATC

>TRICA|TCOGS2:TC004114-PA/1-775 pep:novel scaffold:Tcas3:ChLGX:2622828:2629701:-1 gene:TCOGS2:TC004114 transcript:TCOGS2:TC004114-RA description:"Delta-like protein "

SGVFELRLISFDNEAGKDDKGKCCSGVSGPNSECEGMCRPRFRICLKEYQVKIDTTSPCT

FGDVITTEFETNPGSDNSQSGFDNSIALPFPFTWPGTFSLIVEAWHGNQTSHPAVLVSRL

TRQRWLDVSDKWTEDVHSSNYSTLKFEYRVTCKSHYYGKGCENLCRPRDDQFGHYSCSPT

GERVCLAGWTGDYC

>jgi|Capca1|97577|e_gw1.300.37.1

SGVFELSLERFANGQGVNSDGNCCSGIREQGLCKESCRTFFRVCLNHYQSTISQNPKCTF

GEVTTPIVGYNSFKLEDSSHGFSNPIQLPFDFAWPGTFTLIIEAWHSEINAGPSNDSPRE

LITQISVQRSAQISDEWGHFTFTSETTELEYKYRVVCDANYFGAGCQDLCKPRDDRFGHY

TCSANGTMQCREGWTGKYC

>CAPTE|polychaete|jgi|Capca1|101809|e_gw1.317.47.1/1-1204

GGVFQAQILSYYNKRGELANGQCCQQQVTEPLTNKCSGSCNVTFALCLREHQTRVTYDGA

CTFGSAASGVFRGNRLSFSENDQRSLLELDLEFAWTRTFTMLLEMTHNGSSRWNSRNDTV

FNSSSEVIEHVSYSSVLLPGDAWHVVTHPGPTAAIVYRVRVRCAPHYYNTTCTRLCRPRH

DKFGHYNCNENGDKVCLEGWMGSNC

>Smed_jagged c82477

WNLIILISLKMLSFFPKMIFKLEIIFCFLEIFHGFVESKSQFSLKITKYIKYSFQRLDGK

CCGDHGSVIQHTCTVPCIQRFSICLEYYHASVSPNDPTPYTGHCAYGKRLTEIWGDSSMK

FNKESTHSYKFTIKSSWPGSITLIIEAYDTSETNPYRKAVIDRVVYKNFLKPSYNSSMWN

DVVLSNDRSVYQFSMNLICKPDYYGEFCDRYCKNNGGHYKCDINGEKVCDYGWMGSDC

>Smed_delta c72486

MLQLRMYSKLLILFLINLLYFSQPVQCSKGVFEIHIKKYTLFPTLNLNKCCPTRKNSTNS

CSEDCNIFMEICLSIYQKNPHRYACDIGETYIKTDILGNYALNDSVKRDYSILVRERWKR

NFNIIVKIKHYDLITKKNVVIVTLSSSEVDVKPSSSWITKQLSTPKDHFQLLVSFRYTCS

QNYHGEECDKLCKPSANERGFYNCSSNGDRICHTGYSGSYC

>Pdu_dlike1

DGMIKVRFKEFENKEGKAANGHCCDGRGGGCDHVFKICLDKPNGEKSVENCAYGMKQAEP

IRNVNRIPFGSKINNLDNPLTFVFFEPLPAKVSVKVRIDDHDRFTEDDFVDFLEQRVRTP

TKYTVSNRTTLEFEVFRECTHNFYGHDCSAFCQPAPRFKNQYLCDPETGNKICTSDWGGV

DC

>Pdu_dlike2

SGIASVKFVNYIGDGKMADGKCCVPNIGGRCDLFFTVCLNDPKVTSSDSSPCSVGTFTTE

ITQDSNEIAFTDKIGSAPNPLKINFQSWATAIDLTVTVNHLSAGTNETKLVEELKKSVSI

TLTNTEQEIELSDRTSLFMSISVGCDEYYFGPGCGVYCKSSAGDNYECGKDGEKICKAGW

TGADC

>Pdu_dlike3

SGEVRIKFIKYENDKGKGANGQCCDGKYKNGCDHMFSVCLDAPGKANDITRCQFGKYQST

AIKNRNFNIFDSSIANLKNPLVFKIDTVFPAEFDFKVVVFDHDHGVFARGNDHVDTLTQR

MQPEQMYTTKHRTKLVFEASMVCSNGYYGTNCGTHCPAPSDTSHYECDSTGQKVCLQGWT

GADC

**Delta / *Platynereis* Delta-like / Jagged alignment**

>Pdu_dlike2

SGIASVKFVNYIG-DGKMADGKCCVPNIGGRCDLFFTVCLNDPKVTSSDSSPCSVGT-FT

TEIDSNEIAFPNPLKINF-QSWATAIDLTVTVNHLSAGTETKLVEELKKSVSITLTNTEQ

EIELSDRTS-LFMSISVGCDEYYFGPGCGVYCKSSAGD--NYECGKDGEKICKAGWTGAD

C

>Pdu_dlike1

DGMIKVRFKEFENKEGKAANGHCCDGR-GGGCDHVFKICLDKPNGSVE---NCAYGMKAE

PIRNVNRIPFDNPLTFVFFEPLPAKVSVKVRIDDHDRFTEDDFVDFLEQRVR-TP----T

KYTVSNRTT-LEFEVFRECTHNFYGHDCSAFCQPAPRFKNQYLCDPTGNKICTSDWGGVD

C

>Pdu_dlike3

SGEVRIKFIKYENDKGKGANGQCCDGKYKNGCDHMFSVCLDAPGKDIT---RCQFGKYQS

TAIKNRNFNIKNPLVFKIDTVFPAEFDFKVVVFDHDHGVGNDHVDTLTQR--MQPEQMYT

TKHRTK----LVFEASMVCSNGYYGTNCGTHC-PAPSDTSHYECDSTGQKVCLQGWTGAD

C

>Pdu_delta

SGVFELQLLSFMNEKGLNADGNCCHGYCSESCKTFFKICLKHYQANISPGPPCTFGSITT

GVVGDNTFEFSNPISFPFDFAWPGTFSLIIEAWNSETPNPRELITRLATQKSLNVGDPWE

NYTHHTNSSELRMKYRVRCETDYYGRGCTELCRSRDDRFGHYTCSRNGSKVCLDGWTGSY

C

>Pdu_jagged

SGQFQMQLVSLQNVKGELASGYCCRGECLAPCRTFIRFCLREYQTRNLSATSCAFGNATS

RVLGGNVIT-EEIFVIPFSFAWTRAFTLVFEILHMADGEEEQVIARGSHSGVVLPSPDWH

LMTFSASSGSMTYSLRVSCDSNYYNTTCTKLCRPRHDAFGHYRCDSNGDKVCLQGWMGTN

C

>Sme_d_c724

KGVFEIHIKKYT-LFPTLNLNKCCPTRCSEDCNIFMEICLSIYQKNPHRY-ACDIGETKT

DILGNYALNDKRDYSILVRERWKRNFNIIVKIKHYDLITKNVVIVTLSSSEVVKPSSSWI

TKQLSTPKDHLLVSFRYTCSQNYHGEECDKLCKPSANERGFYNCSSNGDRICHTGYSGSY

C

>Sme_j_c824

KSQFSLKITKYIKYSFQRLDGKCCGDHCTVPCIQRFSICLEYYHASVSPNDHCAYGKRLT

EIWGDSSMKFTHSYKFTIKSSWPGSITLIIEAYDTSETNRKAVIDRVVYKNFLKPSSMWN

DVVLSNDRSVYQFSMNLICKPDYYGEFCDRYCKNNG---GHYKCDINGEKVCDYGWMGSD

C

>Hro_P77153

---FQTSQPPSSSSSSSSSSSSSSSSSPSNRCNVFFKICLTHQQPKLKNFPDCTFGSTVT

DVLRNNSSNMHHTVRLPISYKWTHKFALIIEAYHMDDDNQQGLLLKLILLKNVTSEQGWI

TESFHNGSTKLDYRYRLVCLHNIYGPFCSNSCVARDDSLGHYRCLRNGTKSCLQGWTGEW

C

>Hro_190982

-------------------------------MAMHFKISFWHSKE------TC-------

------------------------EFYVGTDA-------DSYLIERAHYSGVILPRSEWQ

TMSFAGHTAILKYKIRVQCDLHHFNVTCTKFCRPRNDTFGHYTCDSNGDKVCLNGWIGTN

C

>Aca_524885

HATPEPPGSSAPDPRSRGPQWRCLFGYWPISCVPSVGQCGPSRRLTSVFRPKVKYEEREA

DEAAHHSRKMPQTLIVPFCFCSQGDFSLIIEAWHDTSLDRSTLISRLAEIRSAASGPDWY

TYVHTFNYTELKYAFRFTCDSNYFGAKCADLCRARDDKFGHYSCAENGTKVCLDGWDGEY

C

>Nve_16910

AGNFEVQFVSIQNVAGELRNGKCCDGSCLDACETFFTICLKPSDGAGSNSGTCTFGQYTT

KVLGGNSFTVEQIVSLRFTFSWLKTYLLVLEARDSDNTT-SQLIDETSLRGILFPNKTWE

SHSYNGPTASIKYNVRVVCDEHYYGRTCTILCKPRDDIFGHYTCDEQGHKICLPGWRGDH

C

>Sko_259013

SGIFQLRLASFSNDQGRNVSGQCCSVSCGSPCRTFFRVCLKHYQAHVSTDDDCIYGEVET

PILGNNTFEIINPIRLPFTFRWPGTFSLVIEAFHSVEVGPRSLIARLATQRFAAVGFEWN

TEVYSSEDTDLHYSYRIVCDEHYFGEECSDFCRPRDDNLGHYTCDAFGNKVCLPGWGGDS

V

>Sko_585706

SGYFELQILSVWNAAGELENGDCCDGVTQDSCDTQVSVCLKEFQSSVTRDGPCTFGKAHS

QILGGNSFTLAARLKLSFEFRWMTFCTLILKILDNDNTSREELIDTATHSGSLKPGSGWQ

TLNSPGTVAQVAYKIRIMCDEYYYSTDCMTFCKPRDDDFGHYTCDSNGNKLCNTGWSGTN

C

>Lgi_224870

SGVFEVKIRSLFNKHGRTVSGRCCAG-CTATCRTFFTLCLLHYQKPVPVYPACTFGETKT

QIVGANNIGIAFSVAIPFSFSWPGNFAFILDAWHDQHRNTRHIILRASRAKRLKPSPLWV

EDERLTRTSRLEFSYRVTCDENYHGLGCDKWCKERDDQFGHYRCTQGGVKVCSQGWQGEL

C

>Lgi_71205

SGYFHLQVNSVSNPRGEIADGSCCNGGCSEKCNTFFRVCLKEYQERVTTTGTCTFGNQST

VALGGNSFTYRAMLKLPFEFAWTRSYTLMVEAWDHSTNNMDSLIERAAQTGMILPGQDWH

TIIHTGPTASLVYKIQVVCDEHYYNTTCTKFCRPHNDRFGHYTCDSNGDKVCLHGWMGQE

C

>Lgi_234999

LGTFELKLTSFLNAHGLNSDGNCCNGLCSSSCKTFFRVCLTHYQSEISNNPECTFGSKTT

SVLGNNTIQFKNPVQFEFQFSWPGSFSLIIEAWHDSTLHPRELIARVAVQRSAEVGKDWY

TFKHDTPYSEINYSYRIICGEYYYGAGCSEFCRPRDDQFGHYKCSTNGTKICLDGWSGDM

C

>Cgi_42676

---------------------------------------------------MVIRGSR--

---GVLEINR--------------SYTLILEAWDQDTMAGGQLIERAAHSGIILPGQDWH

TITHNGPTASLIYRIRVVCDDHYYNTTCTKFCRPRNDHFGHYTCDRNGDKVCMQGWMGNN

C

>Cgi_19039

TGIFELNLKRFENENGVNADGNCCAGVCTSWCRTFFRVCLTHYQATITDDVHCNFAEKFT

PVLGNNSVDFDIPIKFPFQFAWPGTFTLIIEAWHDSTQQTRELIYRLQKQRSVEVGSSWK

QFTRTENTMTLEYAYKVECGENYYGPGCVNYCRPRDDSFGHYVCDEYGQKVCMQGWKGDY

C

>Cgi_18324

TGRFEVSILAYFNESGRMAGESCCSGSCLSLCPTFLTVCLQHHSAFIPEFPACTYGHAVT

PVFSLTDDVKMFFIKIPFQFSWPESFSLVIDAWQEPQLNSNRILRDVVRDK-LTPPSLWV

KRRTKTAQLELIYEYRVVCDEFYYSNTCEQICRHRNDNFGHYVCDETGSKVCLPGWQGEF

C

>Isc_13622

SGVFELQLRAFSNLLSQDSRGSCCSTGCPGTCRTWFRVCLKHYQKTVDTNSPCTYGEVQT

PVVSGPGSTLTAPVRFSFDFSWPGTFSLIVEAWHENSNGGNSVGELVCLGGSERACVVWR

RRQQPSDPGAAVFSFRVVCEEHYFGADCARLCRPRDDKFGHYACNAKGDVVCLPGWRGDY

C

>Cte_101809

GGVFQAQILSYYNKRGELANGQCCQQQCSGSCNVTFALCLREHQTRVTYDGACTFGSAAS

GVFRGNRLSFRSLLELDLEFAWTRTFTMLLEMWNSRNDTSSEVIEHVSYSSVLLPGDAWH

VVTHPGPTAAIVYRVRVRCAPHYYNTTCTRLCRPRHDKFGHYNCNENGDKVCLEGWMGSN

C

>Cte_97577

SGVFELSLERFANGQGVNSDGNCCSGICKESCRTFFRVCLNHYQSTISQNPKCTFGEVTT

PIVGYNSFKLSNPIQLPFDFAWPGTFTLIIEAWHSEINAPRELITQISVQRSAQISDEWG

HFTFTSETTELEYKYRVVCDANYFGAGCQDLCKPRDDRFGHYTCSANGTMQCREGWTGKY

C

>Tca_892396

SGFFELQVLEMANPRGELSTGECCGGGCSTPCNTFFRLCLKEYQSNVTSTGSCSFGNTSS

VVLGRDSFTLRGKLVLPFTFRWTRSFTLILQAVDHNNFTTNDIIEEATYSGIIDPSPEWH

TLNHRGPKATLTYRVRVKCDSHYYNATCTKFCRPRDDKFGHYICDANGDKECIEGWKGAT

C

>Tca_004114

SGVFELRLISFDNEAGKDDKGKCCSGVCEGMCRPRFRICLKEYQVKIDTTSPCTFGDVIT

TEFNPGSDNSDNSIALPFPFTWPGTFSLIVEAWHGNQTSPAVLVSRLTRQRWLDVSDKWT

EDVHSSNYSTLKFEYRVTCKSHYYGKGCENLCRPRDDQFGHYSCSPTGERVCLAGWTGDY

C

>Hsa_217042

MGYFELQLSALRNVNGELLSGACCDGDGHDECDTYVRVCLKEYQAKVTPTGPCSYGHGAT

PVLGGNSFYLPGLVVIPFQFAWPRSFTLIVEAWDWDNDTEELLIERVSHAGMINPEDRWK

SLHFSGHVAHLELQIRVRCDENYYSATCNKFCRPRNDFFGHYTCDQYGNKACMDGWMGKE

C

>Hsa_455767

SGQFELEILSMQNVNGELQNGNCCGGATRDECDTYFKVCLKEYQSRVTAGGPCSFGSGST

PVIGGNTFNLRNRIVLPFSFAWPRSYTLLVEAWDSSNDTPDSIIEKASHSGMINPSRQWQ

TLKQNTGVAHFEYQIRVTCDDYYYGFGCNKFCRPRDDFFGHYACDQNGNKTCMEGWMGPE

C

>Hsa_110735

SGVFELKLQEFVNKKGLLGNRNCCRGGPPCACRTFFRVCLKHYQASVSPEPPCTYGSAVT

PVLGVDSFSLSNPIRFPFGFTWPGTFSLIIEALHTDSPDPERLISRLATQRHLTVGEEWS

QDLHSSGRTDLKYSYRFVCDEHYYGEGCSVFCRPRDDAFGHFTCGERGEKVCNPGWKGPY

C

>Hsa_950654

SGVFQLQLQEFINERGVLASGRPCEPG----CRTFFRVCLKHFQAVVSPG-PCTFGTVST

PVLGTNSFAVRNPLQLPFNFTWPGTFSLIIEAWHAPGDDPDALISKIAIQGSLAVGQNWL

LDEQTSTLTRLRYSYRVICSDNYYGDNCSRLCKKRNDHFGHYVCQPDGNLSCLPGWTGEY

C

>Aqu_54208

RYTLLVKAVQYHNPDNQDWNDGCCDWPCSNNCDLYFKFCIRNKGLSASNINNCTYGDVST

NNYPNYGELYWNDLTFNRNEPWPGSVQVLVESLDADDN-ADDLIDRNAFNLDLSPNGQWS

NELYANGYYRFKIRVRLFCQQNYYGSNCNVYCVQQNDDTGHYTCGSDGAKICNNGYTNGN

C

>Aqu_54207

DYELLFRFRRYSNPTDRDNNDNCCDNLCVSHCDTLFRLCLRNRDS--NSEPQCIPGTVPG

TVGNGPDNSIVGSISNPIIYERPGGFQLYMEVFDYDSRNDNDYIDSIVLNIPAQPTTERQ

STVVGEDGKSLELSYSLSCSQNYYGSDCSQQCIPRNDNTGHYTCNTTGGIICREGWQNTN

C

>Aqu_54210

DFTLSFQFHRYSNPSDRDVNGNCCDTAICGSCDTHFKLCLRNGGTSHSQTGQCIPGTVLS

PGGGGSSVNFIGVISNPFGYNKSGGFQLYLEVWDDDYAFNDDLIDRIVFDILRTSSQISH

TAIGVFNRVSLLSSYRLLCSVNYYGFDCSVLCIPYNDDTGHYTCNSTGAKKCREGWQNNN

C

>Aqu_340376

DFGLQFQFYRYSNPSDRNFNGNCCD--WCDSCDTLFKLCLRNGGTSHSETGHCIQSTVAN

PGRGSSTSVNIGSTPNPFDYTRSGGFQLYLEVWDDDTFSSDDLIDRIVLDVPIHPSGVSR

TVSGVFNRVSLVLSYTLSCRLNYYGSDCSVLCIPYNDDTGHYTCNSTGARICRDGWQNNY

C

***CAML***

>Pdu_CAML_EF hand domain

NKDELSEFRESFEMFDVNRDGYITMEELRGMMESLDQKCSDIELQRIMKAADLDKNGKIDFNEFVIIMDKFCPENFENFEDQLREAFQLIDKDNSGKISSTELKQVLRRIGENDITDDEIEDLVREIDLDGDGEVDYEEFVKIMVSK
